# Why is thiol unexpectedly less reactive but more selective than alcohol in phenanthroline-catalyzed 1,2-*cis O*- and *S*-furanosylations?†

**DOI:** 10.1039/d4ob01593b

**Published:** 2024-11-18

**Authors:** Boddu S. Ramakrishna, Neha Rani, Hengfu Xu, Cyrus Alan-Lee, H. Bernhard Schlegel, Hien M. Nguyen

**Affiliations:** a Department of Chemistry, Wayne State University Detroit Michigan 48202 USA hbs@chem.wayne.edu hmnguyen@wayne.edu

## Abstract

The lack of catalytic stereoselective approaches for producing 1,2-*cis S*-furanosides emphasizes the critical need for further research in this area. Herein, we present a stereoselective *S*-furanosylation method, utilizing a 4,7-dipiperidine-substituted phenanthroline catalyst. This developed protocol fills a gap in the field, enabling the coupling of cysteine residues and thiols with furanosyl bromide electrophiles. The process allows for stereoselective access to 1,2-*cis S*-furanosides. Through computational and experimental investigations, thiol is found to be less reactive than alcohol but exhibits greater stereoselectivity. The 1,2-*cis* stereoselectivity of *O*-products depends on the nature of the electrophile, while *S*-products are obtained with excellent 1,2-*cis* stereoselectivity, irrespective of the furanose structure. The displaced bromide ion from the glycosyl electrophile influences the reaction's reactivity and stereoselectivity. Alcohol-OH forms a stronger hydrogen bond with bromide ion than thiol-SH, contributing to the difference in their reactivity. The energy difference between forming *S*-furanoside and *O*-furanoside transition states is 3.7 kcal mol^−1^, supporting the increased reactivity of alcohol over thiol. The difference in transition state energies between the major and minor *S*-product is greater than that for the major and minor *O*-product. This is consistent with experimental data showing how thiol is more stereoselective than alcohol. The catalyst and reaction conditions utilized for the generation of 1,2-*cis O*-furanosides in our prior studies are found to be unsuitable for the synthesis of 1,2-*cis S*-furanosides. In the present study, a highly reactive phenanthroline catalyst and specific reaction conditions have been developed to achieve stereoselective *S*-linked product formation.

## Abstract 2

Thiol is less reactive but more selective than alcohol. Alcohol-OH forms a stronger hydrogen bond with displaced bromide anion than thiol-SH, contributing to the difference in their reactivity and selectivity.

## Graphical abstract

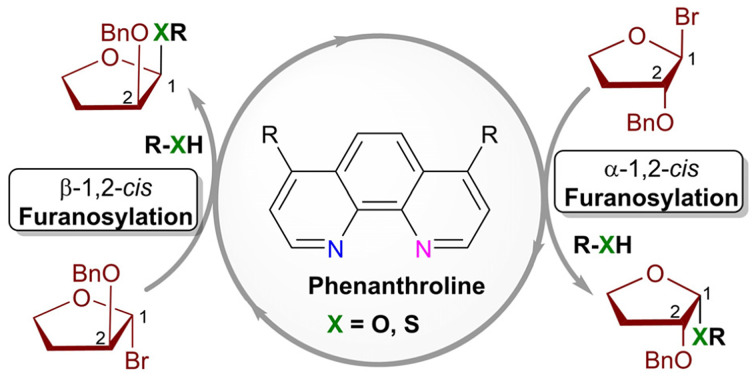

## Introduction

The substitution of a sulfur atom for the anomeric oxygen atom in oligosaccharides has attracted significant attention due to the important applications of *S*-oligosaccharides in biochemical research. *S*-Oligosaccharides can act as competitive inhibitors for glycoside hydrolase enzymes.^1^ Synthetic antigens have been created using *S*-linked oligosaccharides, which elicit immune responses similar to native *O*-linked epitopes.^2^ The choice to replace oxygen with sulfur was based on several factors. *S*-Glycosides are well-tolerated by most biological systems and exhibit activities comparable to or better than their native *O*-glycoside counterparts.^3^ They also preserve the natural conformation of *O*-linked substrates when in solution and complexed with proteins.^4^ While the C–S bond is longer and more flexible than the C–O bond, the C–S–C angle is smaller than the C–O–C angle, which leads to slight differences in the glycosidic bond.^5^ Additionally, *S*-glycosides are less prone to hydrolysis by acid/base or enzymes.^3,6^ It is worth noting that the discovery of *S*-glycosylation of cysteine residues as a new post-translation modification found in prokaryotes is both novel and intriguing.^7,8^

**Fig. 1:**
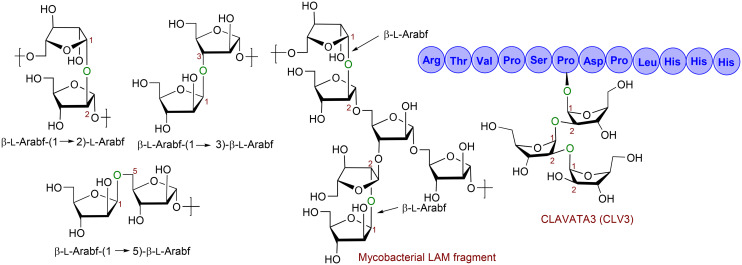
Biologically important oligo l-arabinofuranosides bearing the β-l-Arabf-(1,2)-l-Arabf, β-l-Arabf-(1,3)-l-Arabf, or β-l-Arabf-(1,5)-l-Arabf motif. Arabf = arabinofuranose.

The *S*-furanosides could also potentially be used as inhibitors of β-l-arabinofuranosidases to prevent hydrolysis of β-(1 → 2), β-(1 → 3), and β-(1 → 5) linkages of β-l-Arabf disaccharides (Fig. 1).^13,14^ Additionally, *S*-linked furanosides could serve as mimetics of furanosides containing the β-l-arabinofuranoside (β-l-Arabf) motifs,^13,15,16^ offering a promising strategy for studying and manipulating these motifs. Notably, oligoarabinosides containing β-l-Arabf-(1 → 2)-l-Arabf motifs are present in the cell-wall polysaccharides of lipoarbinomannan (LAM), which are critical for the growth, survival, and virulence of *M. tuberculosis* (Fig. 1).^17^ The β-l-Arabf-(1 → 2)-l-Arabf unit has also been discovered to glycosylate hydroxyproline residues during post-translational modification in the hydroxyproline-rich proteins, CLAVATA3, (Fig. 1)^18,19^ and arabinogalactan proteins,^20^ highlighting the potential applications of *S*-linked furanosides in various biological processes.

**Scheme 1:**
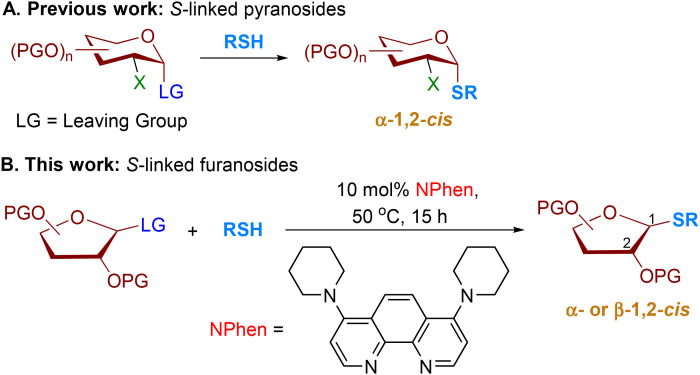
Stereoselective construction of *S*-linked pyranosides and furanosides.

Similar to *O*-linked pyranosides, the vulnerability of β-l-arabinofuranosides to acid/base and enzymatic hydrolysis has been reported.^13^ As the interest in arabinofuranosides continues to grow, *S*-arabinofuranosides could potentially offer a solution to the challenges associated with native *O*-arabinofuranosides.^6^ Efficient methods have been developed for the stereoselective formation of α-1,2-*cis S*-linked pyranosides and pyranosyl peptides (Scheme 1).^6,9,10^ In contrast, the catalytic stereoselective synthesis of β-arabinofuranosides is underdeveloped due to challenges arising from the conformational flexibility and electronic properties of furanose, the steric hindrance of the C2-substituent of arabinose, and the absence of available anchimeric assistance.^21^ Several groups have reported the highly controlled formation of 1,2-*cis* β-*O*-arabinofuranosidic linkages.^22,23^ However, the catalytic, stereoselective method for the synthesis of *S*-linked furanosides remains elusive.^11,12^

In the context of our research interest in phenanthroline-catalyzed stereoselective 1,2-*cis O*-furanosylation,^24^ we postulated that the system could potentially be applied to achieve stereoselective 1,2-*cis S*-furanosylation. However, challenges must be addressed as the phenanthroline system provided 1,2-*cis O*-arabinofuranosides in moderate stereoselectivity.^24^ Thiol competition with phenanthroline catalysts can affect product stereoselectivity. As a result, a more reactive phenanthroline catalyst is developed for the stereoselective synthesis of 1,2-*cis S*-furanosides. Our findings show that less reactive thiol exhibits higher stereoselectivity than more reactive alcohol under phenanthroline-catalyzed conditions. In the present study, a 4,7-piperidine substituted phenanthroline (NPhen) catalyst has been developed in conjunction with specific conditions to achieve the stereoselective formation of 1,2-*cis S*-furanosides (Scheme 1). Our developed protocol exhibits a high 1,2-*cis* stereoselectivity for coupling a variety of peptides containing cysteine residue and thiol nucleophiles with diverse furanosyl electrophiles. This developed protocol results in the production of 1,2-*cis S*-furanoside products in good yields with excellent levels of diastereoselectivity (*cis* : *trans* = 15 : 1–25 : 1). Unlike *O*-furanosylation, the selectivity of the *S*-products is not influenced by the stereochemical nature of furanosyl bromide donors. Our findings suggest that the displacement of the bromide ion from furanosyl donors influences the stereoselectivity and reactivity of the reaction, as evidenced by our density functional theory calculations.

## Results and discussion

### Reaction development

**Scheme 2:**
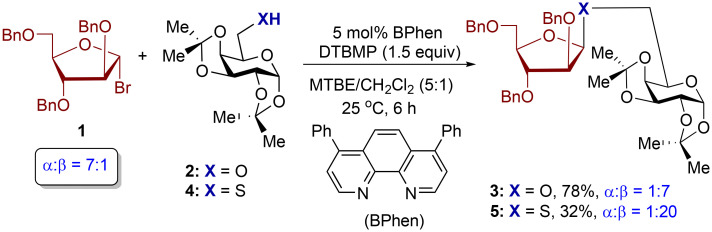
Preliminary studies with readily available 4,7-diphenyl-1,10-phenanthroline (BPhen) catalyst.

In our study, we initiated our investigation by examining the potential of thiogalactoside bis-acetonide 4 as a nucleophile in its reaction with tribenzyl arabinofuranosyl bromide 1 (Scheme 2). The reaction was conducted under previously optimized conditions for stereoselective *O*-furanosylations, utilizing 5 mol% 4,7-diphenyl-1,10-phenanthroline (BPhen) as a catalyst and di-*tert*-butylmethylpyridine (DTBMP) as an acid scavenger of HBr in a 5 : 1 mixture of MTBE and CH_2_Cl_2_ (0.2 M) at 25 °C for 6 hours. Our findings revealed that thiogalactoside 4 displayed high levels of 1,2-*cis* stereoselectivity (*α* : *β* = 1 : 20) in comparison to galactoside alcohol 2 (*α* : *β* = 1 : 7). This stereoselectivity of the *S*-product 5 was not dependent on the anomeric composition of furanosyl bromide 1 (*α* : *β* = 7 : 1). However, we observed that thiol 4 exhibits lower reactivity than alcohol 2 under phenanthroline-catalyzed glycosylation. The lower reactivity of thiol 4 led to the formation of 32% of *S*-product 5 (Scheme 2), alongside unreacted donor 1 remaining in the reaction mixture. In contrast, alcohol 2 reacted with donor 1 to yield *O*-product 3 with a higher yield (78%, Scheme 2).

In the following study, a series of experiments were performed to optimize the *S*-furanosylation reaction by adjusting various parameters, including solvent, temperature, catalyst loading, and reaction concentration (Table S1†). The reaction of thiogalactoside 4 with arabinosyl bromide 1 proceeded smoothly without catalyst deactivation, enabling the approximation of the reaction rate and conversion using the yield of *S*-product 5. By testing different conditions, it was determined that utilizing 10 mol% of BPhen in MTBE at a concentration of 0.5 M (ref. 25 and 26) and 50 °C for 15 h significantly increased the yield of *S*-product 5 from 32% to 69% (Table S1†), in comparison to the optimized conditions for *O*-product 3 (Scheme 2). Furthermore, the use of 4,7-piperidine substituted phenanthroline, NPhen, as the catalyst further enhanced the yield of product 5 (from 69% to 79%) and improved the 1,2-*cis* stereoselectivity (*α* : *β* = 1 : 20 → 1 : 25, Table 1). However, modifying the electronic properties of the 4,7-substituents on the phenanthroline framework, as observed in catalysts like MeOPhen, BrPhen, and Phen, did not yield significant improvements (Table 1). Similar trends were observed when introducing 2,9-substituents onto the phenanthroline framework to form hindered catalysts such as MePhen, *n*-BuPhen, and PhPhen, reducing yield and stereoselectivity. In the absence of a phenanthroline catalyst, the reaction proceeded sluggishly, resulting in a 13% yield of product 5 with *α* : *β* = 1 : 10. It was also observed that the optimized conditions for forming *S*-product 5 were unsuitable for forming *O*-product 3 (*α* : *β* = 1 : 2, Scheme S1†).

**Table 1:** Evaluation of phenanthroline catalysts for stereoselective *S*-furanosylation^a^

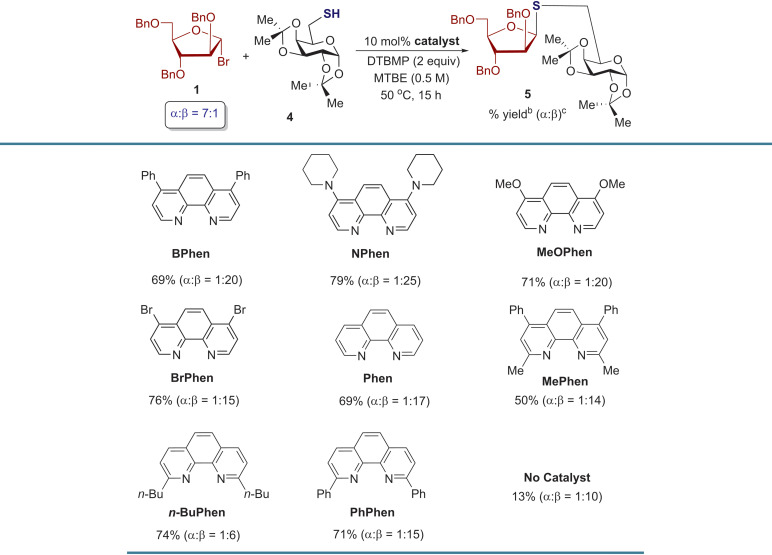

a All reactions were performed with 0.3 mmol of bromide donor 1, 0.1 mmol of thiol 4, and 10 mol% of catalysts with respect to donor 1.

b Isolated yields.

c The *α* : *β* ratio was determined by ^1^H NMR analysis.

### Substrate scope

**Scheme 3:**
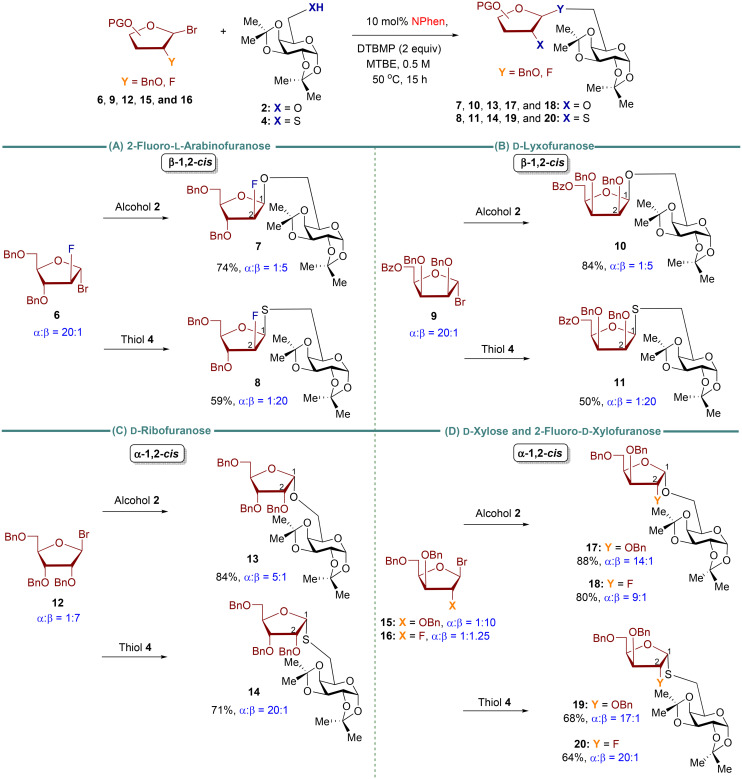
Reactivity and stereoselectivity differences between *O*-furanosides and *S*-furanosides. All reactions were conducted with furanosyl bromide (0.2 mmol), thiol or alcohol acceptors (0.1 mmol), and 10 mol% of NPhen with respect to donor at 50 °C for 15 h. Isolated yield was calculated. The *α*/*β* ratio was determined by ^1^H NMR.

Our findings indicate that alcohol 2 is less 1,2-*cis* stereoselective than thiol 4 when reacting with arabinofuranosyl bromide 1. As such, we question whether a similar trend applies to other furanosyl donor substrates (Scheme 3). In previously optimized *O*-furanosylation studies, furanosyl bromide donors 6, 9, and 12 exhibited moderate 1,2-*cis* stereoselectivity (*cis*/*trans* = 2 : 1–7 : 1).^24^ To directly compare thiol 4 and alcohol 2, the optimized conditions for the thiol are applied to the corresponding alcohol (Scheme 3). These donors exhibited high diastereoselectivity upon reaction with thiol 4, leading to the formation of *S*-furanoside products 8, 11, and 14 (*cis*/*trans* = 20 : 1). This level of diastereoselectivity surpasses that observed in the *O*-furanoside counterparts 7, 10, and 13 (*cis*/*trans* = 5 : 1). Xylosyl bromide 15 and 2-fluoro-xylosyl bromide 16 were highly stereoselective for both *O*- and *S*-furanosylation. It is generally observed that *S*-products were obtained at lower yields than *O*-products for all furanosyl donors tested. In certain instances, unreacted starting donors could be isolated. However, the high reaction temperatures and longer reaction times led to the decomposition of the donor and acceptor into unknown compounds that proved challenging to identify.

Next, we examine the thiol scope with donor 1 (Table 2). We found that all *S*-arabinofuranoside products were formed with excellent levels of β-stereoselectivity (*α* : *β* = 1 : 15–1 : 25). At the outset, we assessed multiple furanoside acceptors featuring thiol functionality at the C5, C3, and C2-positions. The reactions of these furanosyl thiols produced β-1,5-, β-1,3-, and β-1,2-*S*-disaccharides 21, 23, and 24 in synthetically useful yield with excellent levels of 1,2-*cis* β-diastereoselectivity (*α* : *β* = 1 : 15–1 : 20). By comparison, moderate β-stereoselectivity was observed with *O*-product 22 (*α* : *β* = 1 : 5).^24^ The established protocol was also applied to farnesyl thiol and tetrazole-5-thiol, resulting in the formation of *S*-furanoside products 25 and 26, respectively, exhibiting high 1,2-*cis* diastereoselectivity (*α* : *β* = 1 : 20).

**Table 2:** Reaction of thiol nucleophiles with l-arabinofuranosyl bromide^a^

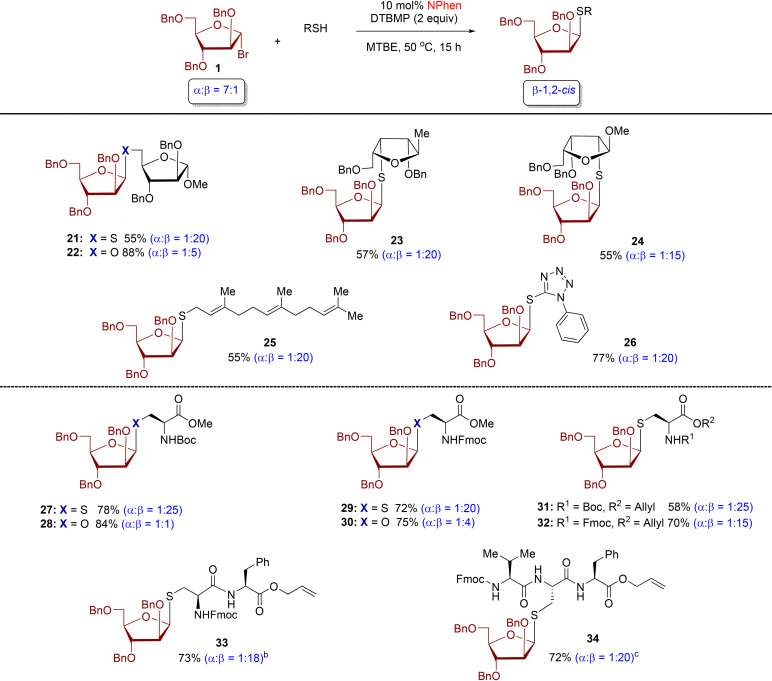

a All reactions were conducted with arabinofuranosyl bromide 1 (0.2 mmol), thiol acceptors (0.1 mmol), and 10 mol% of NPhen to donor 1 at 50 °C for 15 h. Isolated yield was calculated. The *α*/*β* ratio was determined by ^1^H NMR.

b The reaction was conducted with 10 mol% NPhen in CH_2_Cl_2_ at 25 °C.

c The reaction was conducted with 10 mol% NPhen in CH_2_Cl_2_/MeCN (5 : 1) at 25 °C.

Next, our attention turned to arabinofuranosylation of cysteine residues and cysteine-containing peptides (Table 2). The furanosylation reaction of cysteine residues with high stereoselectivity remains underdeveloped. We conducted testing using a combination of *N*-Boc- and *N*-Fmoc-protected cysteine residues with l-arabinofuranosyl bromide 1. The resulting *S*-glycoconjugate products 27, 29, 31, and 32 exhibited significant levels of β-1,2-*cis* diastereoselectivity (*α* : *β* = 1 : 15–1 : 25). The phenanthroline-catalyzed conditions tolerated both *N*-Fmoc- and *N*-Boc-protected cysteine residues, commonly used in solid-phase peptide synthesis. The *N*-Fmoc-protected products 29 and 32 obtained higher yields than their *N*-Boc counterparts 27 and 31 but with slightly lower stereoselectivity. The coupling of *N*-Boc- and *N*-Fmoc-protected serine residues afforded *O*-furanoside products 28 and 30, respectively, with *α* : *β* = 1 : 1–1 : 4. Furthermore, dipeptide l-Cys-l-Phe and tripeptide l-Val-l-Cys-l-Phe were effectively engaged as thiol nucleophiles, resulting in the production of highly yielding and β-stereoselective glycopeptides 33 (73%, *α* : *β* = 1 : 18) and 34 (72%, *α* : *β* = 1 : 20), respectively.

A recent discovery has confirmed the existence of natural *S*-glycoproteins, which are sugars linked to the sulfur atom of cysteine on bacterial peptides during post-translational modification.^7,27^ This exciting development has sparked interest in bacterial glycoproteins and their potential therapeutic applications.^28^ Currently, methods for the synthesis of *S*-linked glycopeptides are restricted to pyranose substrates.^10,29^ In addition, only a few catalytic stereoselective methods have been reported.^30^ Building on the efficient and stereoselective phenanthroline-catalyzed reactions of diverse thiols with l-arabinofuranosyl bromide donor 1, an investigation was conducted to explore the potential application of this developed protocol with other furanosyl and pyranosyl bromide donors (Table 3). This catalysis method can be applied to other furanosyl donors, producing *S*-furanosylated cysteine products (35–42) with outstanding 1,2-*cis* diastereoselectivity (*cis*/*trans* = 15 : 1–25 : 1), regardless of the anomeric composition of furanosyl bromide donors and their stereochemically distinct structures (Table 3). The 2-fluoro-furanosyl bromide donors and cysteine residues exhibited low reactivity, resulting in moderate yields for *S*-products 35, 36, and 42. In addition, the procedure has been effectively utilized for the coupling with a variety of highly pure α-pyranosyl bromide donors, leading to *S*-pyranoside products 44–49 with highly diastereoselective purity and net retention of anomeric configuration. These findings underscore the significant potential of these methods in the synthesis of glycoproteins with diverse structures.

**Table 3:** Reaction of furanosyl and pyranosyl bromide donor with cysteine residues^a^

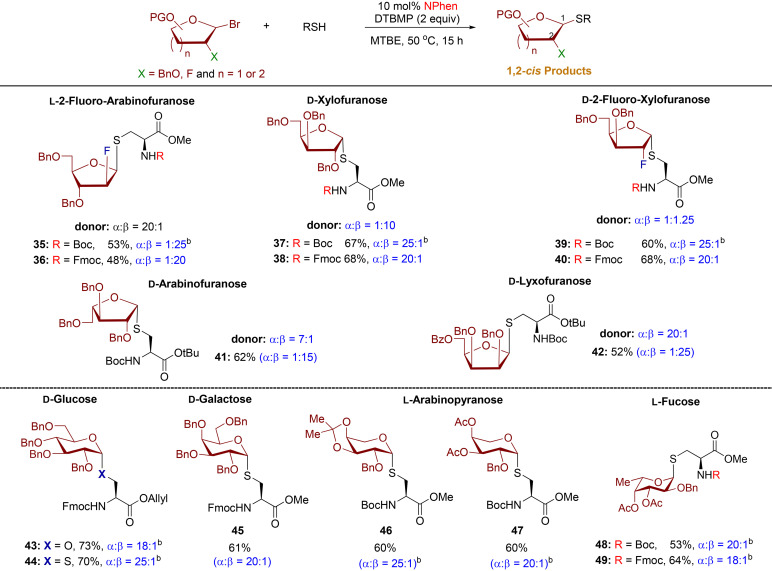

a All furanosylations and pyranosylations were conducted with donors (0.2 mmol), cysteine residues (0.1 mmol), and 10 mol% of NPhen with respect to the donor at 50 °C for 15 h. Isolated yield was calculated. The *α*/*β* ratio was determined by ^1^H NMR. In the case of pyranosyl substrates, α-bromide donors were used.

b The reactions were conducted in MTBE/DCE (5 : 1).

### Mechanistic and computational studies

**Fig. 2:**
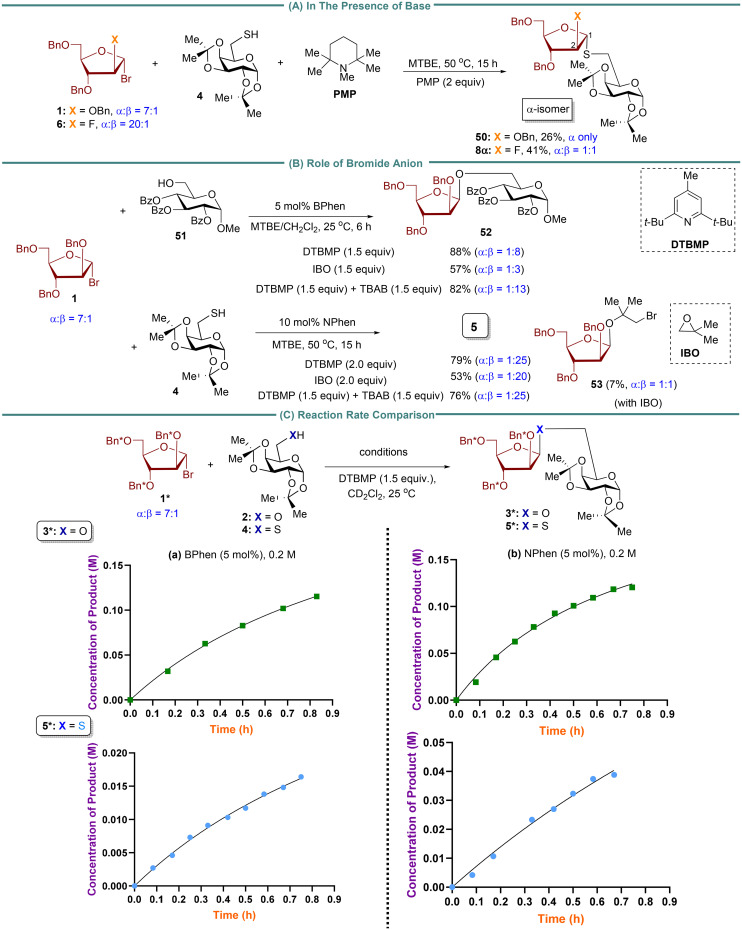
(A) Control experiment with the bulk base, penta-methylpiperidine (PMP). (B) Studies the role of bromide ion in influencing reaction selectivity difference between alcohol and thiol. (C) Reaction rate comparison between alcohol and thiol.

To understand how β-1,2-*cis S*-furanosides are formed, we conducted a control experiment using a strong base, penta-methylpiperidine (PMP), with a p*K*_a_ value of 11.5 (Fig. 2A) and excluding phenanthroline. We hypothesized that PMP could deprotonate thiol, producing thiolate *in situ*. Thiolate is expected to be more nucleophilic than thiol. The unexpected product, 1,2-*trans S*-arabinofuranoside 50 (Fig. 2A), predominantly as a single α-isomer, was observed with the use of arabinosyl donor 1. Conversely, the use of 2-fluoro-arabinosyl donor 6 resulted in a 1 : 1 mixture of α- and β-isomers of product 8. These findings suggest that direct S_N_2 substitution is unlikely in the reaction with thiol. Instead, it is postulated that the reaction may proceed *via* an oxocarbenium ion,^21^ and the stereoselectivity of the *S*-linked product appears to be influenced by the nature of the donor. The data presented also demonstrate the influence of the phenanthroline catalyst in facilitating the formation of 1,2-*cis S*-furanosides (Tables 1–3 and Scheme 3).

Next, we assessed the impact of the bromide ion on the reactivity and stereoselectivity of the reaction, considering that chloride anion has been reported to activate thiol and thiol radical through hydrogen bonding.^31^ We conducted control experiments in the presence of isobutyl oxide (IBO) as acid scavenger (Fig. 2B). We hypothesized that the displaced bromide anion from arabinosyl bromide 1 could be captured by IBO, leaving no bromide ion present in the reaction. In both examples, the yield and stereoselectivity of *O*-product 52 (88 → 57%, *α* : *β* = 1 : 8 → 1 : 3) and *S*-product 5 (79 → 53%, *α* : *β* = 1 : 25 → 1 : 20) were reduced compared to the results obtained in the presence of DTBMP acid scavenger. First, these findings suggest that the bromide ion may influence stereoselectivity. DTBMP can preserve bromide anion, which helps establish a rapid equilibrium between α- and β-arabinofuranosyl bromide donors.^37^ The more reactive α-arabinofuranosyl bromide then undergoes invertive substitution to produce the corresponding β-isomer products 5 and 52.^24^ This bromide-mediated equilibration pathway^37^ can compete with the major operative phenanthroline-catalyzed furanosylation pathway.^24^ The lower yields of products 5 and 52 could be attributed to the instability of donor 1, which is prone to decomposition under IBO-reaction conditions. In the case of thiol acceptor 4, a 7% yield of side product 53 was isolated in the reaction. This result indicates that IBO traps the bromide generated in the reaction to form 1-bromo-2-hydroxyisobutane, which then reacts with donor 1 to produce side product 53. This finding is consistent with our previous studies with 2-fluoro donors,^26^ demonstrating that 1-bromo-2-hydroxyisobutane can compete with sterically hindered or low reactive nucleophiles. However, this nucleophilic competition does not occur with reactive nucleophiles, such as primary alcohols 2 or 51. To investigate the effect of the bromide ion on hydrogen bonding interactions with alcohol compared to thiol,^31^ NMR titration experiments were carried out using TBAB (tetrabutylammonium bromide) with alcohol/thiol (Fig. S5 and S6†). The results showed a downfield shift of the alcohol proton with increasing TBAB concentration, while no significant change was observed in the thiol proton. To further explore the role of the bromide ion, we conducted two control experiments in the presence of an excess amount of TBAB (Fig. 2A). When alcohol 51 was used as the acceptor, the addition of 1.5 equivalents of TBAB markedly enhanced the stereoselectivity (*α* : *β* = 1 : 8 → 1 : 13). In contrast, when thiol 4 was used as the acceptor, the addition of 2.0 equivalents of TBAB resulted in only a slight improvement in the stereoselectivity (*α* : *β* = 1 : 20 → 1 : 25). These findings provide evidence that the bromide ion not only forms a stronger hydrogen bond with alcohol than with thiol. In addition, the *in situ* anomerization of furanosyl bromide, facilitated by external bromide, significantly influences the stereoselectivity of the reaction. Further details regarding the impact of tetrabutylammonium bromide (TBAB) on the stereoselectivity of *O*-furanoside products will be reported in due course.

Previous NMR experiments, kinetic profile, and Density Functional Theory (DFT) calculations on *O*-furanosylation showed that the rate-determining step involves an inverted displacement of the faster-reacting phenanthrolinium ion intermediate with alcohol nucleophile.^24^ The rapid equilibration between the phenanthrolinium ion intermediates is critical to achieving the 1,2-*cis* stereoselectivity.^24^ The present study examines the reaction rate using BPhen and NPhen as catalysts for *O*- and *S*-furanosylation reactions with alcohol 2 and thiol 4. To compare the rates of nucleophilic substitution reactions of alcohol and thiol, the reaction rates between arabinosyl bromide 1 with alcohol 2 and between 1 with thiol 4 were measured using BPhen and NPhen as catalysts (Fig. 2C). We used 2,3,5-tri-benzyl-*d*_7_-arabinofuranosyl bromide 1* as an electrophile to obtain a clear view of the aromatic region in ^1^H NMR. Based on kinetic studies, the product concentration of *O*-furanoside 3* and *S*-furanoside 5*, using either BPhen or NPhen, indicates that the reaction rate with alcohol 2 was approximately five times faster than that of thiol 4. As anticipated, the NPhen-catalyzed furanosylation reaction was more rapid than the BPhen-catalyzed furanosylation reaction for both alcohol and thiol nucleophiles.

In our previous kinetic and DFT studies of *O*-furanosylation, we selected a 2-fluoro-arabinosyl bromide, obtained in a high 1,2-*trans* configuration (20 : 1), as a model donor.^24^ This choice enabled us to conveniently monitor the reaction progress using ^19^F NMR so that we could study the potential impact of the anomeric composition of this donor on the stereochemical outcome in phenanthroline-catalyzed *O*-furanosylation. Additionally, it facilitates an investigation of whether the reaction operates *via* associative pathways.^24^ In our current DFT studies, the 2-fluoro-arabinosyl bromide donor was chosen to maintain consistency with our earlier *O*-furanosylation studies.^24^ Furthermore, the utilization of 2-fluoro-arabinosyl bromide allows for the investigation of the impact of the C2-fluorine atom on reaction stereoselectivity, as the role of fluorine at C2 of pyranosyl donors on 1,2-*trans* glycosylation has been documented.^36^ Our present study involving the 2-fluoro-arabinosyl bromide 6 (*cis* : *trans* = 1 : 20) revealed that both 1,2-*cis O*-products 7 (*cis* : *trans* = 5 : 1) and *S*-product 8 (*cis* : *trans* = 20 : 1) were formed as the major products (Scheme 3A). These findings imply that the reaction is unlikely to proceed *via* the S_N_1 pathway. In the context of the S_N_2 pathway, although we cannot completely rule out the possibility of a direct S_N_2 displacement with thiol in the reaction with 2-fluoro arabinosyl donor 6 based on the outcome for product 8, the result obtained with product 20 (*cis* : *trans* = 20 : 1) from 2-fluoro-xylofuranosyl donor 18 (*cis* : *trans* = 1 : 1.25) (Scheme 3D) suggests that the reaction does not proceed through a direct S_N_2 displacement. Furthermore, the results in Fig. 2A indicate that direct S_N_2 substitution with thiol is not feasible.

**Fig. 3:**
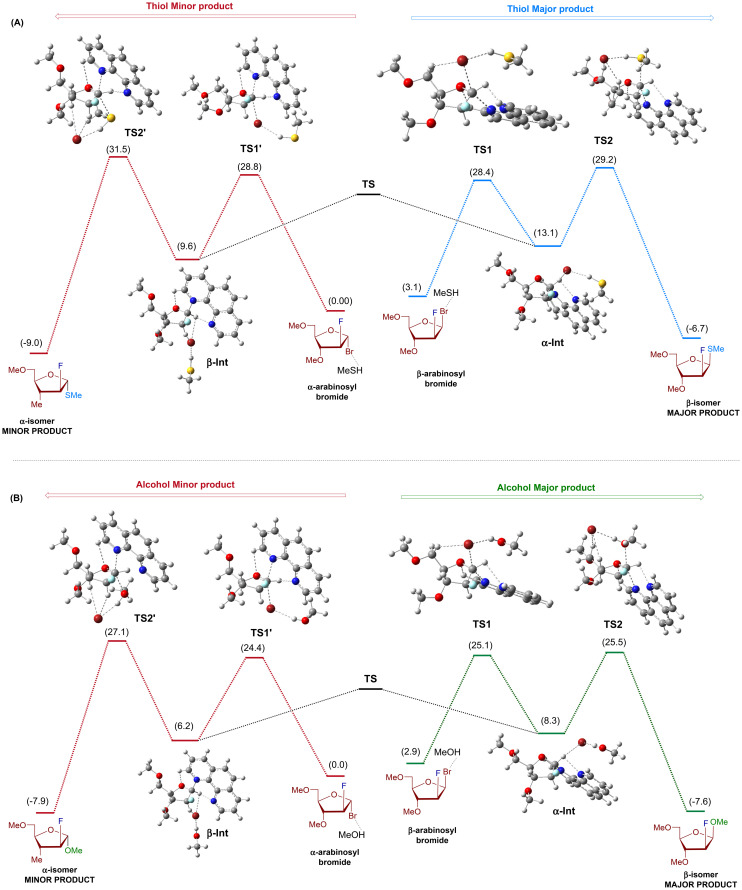
Computed free energy profile diagrams for the formation of α/β-*S*-linked (A) furanoside products from arabinosyl bromide at 50 °C and (B) α/β-*O*-linked furanoside products from arabinosyl bromide at 50 °C. Relative free energy changes (Δ*G*) are in kcal mol^−1^ and are computed with the Gaussian 16 program package^32^ at the M06-2X/def2-TZVPP//M06-2X/def2-SVP level of theory^33^ using diethyl ether with SMD implicit solvation.^34^

By utilizing 2-fluoro-arabinosyl bromide donor, we aim to examine reactivity and stereoselectivity differences between alcohol and thiol using DFT calculations. To reduce the computational cost, we chose tri-methoxy-2-fluoro-arabinosyl bromide, methanol, and methanethiol as model coupling partners. Methanol or methanethiol, forming hydrogen bonds with bromide,^31^ was present in all complexes to maintain consistency across reactants, intermediates, and transition states. As illustrated in Fig. 3, the free energy profile for forming both α- and β-furanosides begins with the arabinosyl bromide donor. Initially, the phenanthroline catalyst displaces the bromide leaving group through transition states TS1 and TS1′, forming the intermediates α-Int and β-Int, respectively (Fig. S7†). Subsequently, the nucleophile (alcohol or thiol) attacks through transition states TS2 and TS2′, forming the final product. Examination of the key intermediates and transition states emphasizes the pivotal role of transition state TS2 in determining the differences in reactivity and stereoselectivity between thiol and alcohol. In the instance of the thiol nucleophile (Fig. 3A), the formation of the β-*S*-product (major) through TS2 requires 29.2 kcal mol^−1^, whereas the α-*S*-product (minor) requires 31.5 kcal mol^−1^. The 2.3 kcal mol^−1^ energy difference between the two transition states (TS2 and TS2′) is consistent with our experimental results and supports the prevalence of the β-*S*-isomer. Similarly, when alcohol attacks on α-Int, it encounters a transition state, TS2, with a barrier of 25.5 kcal mol^−1^ (Fig. 3B), while alcohol attack on β-Int occurs through TS2′, which exhibits a barrier of 27.1 kcal mol^−1^. The 1.6 kcal mol^−1^ energy difference between the two transition states (TS2 and TS2′) also supports the prevalence of the β-isomer as the reaction's major product. Special attention was given to the conformation of all structures, ensuring that the optimized geometries for both major and minor pathways for methanol and methanethiol reactions closely resembled each other. This approach ensures that conformational changes in remote parts of the molecule do not influence small energy differences. The free energy of the transition state for the formation of the major β-*S*-product is lower than that of the minor α-*S*-product by 2.3 kcal mol^−1^. However, this difference in the free energy of activation between the alcohol transition states decreases to 1.6 kcal mol^−1^. The larger energy gap between thiol transition states provides additional support for the increased stereoselectivity observed with thiol.

**Fig. 4:**
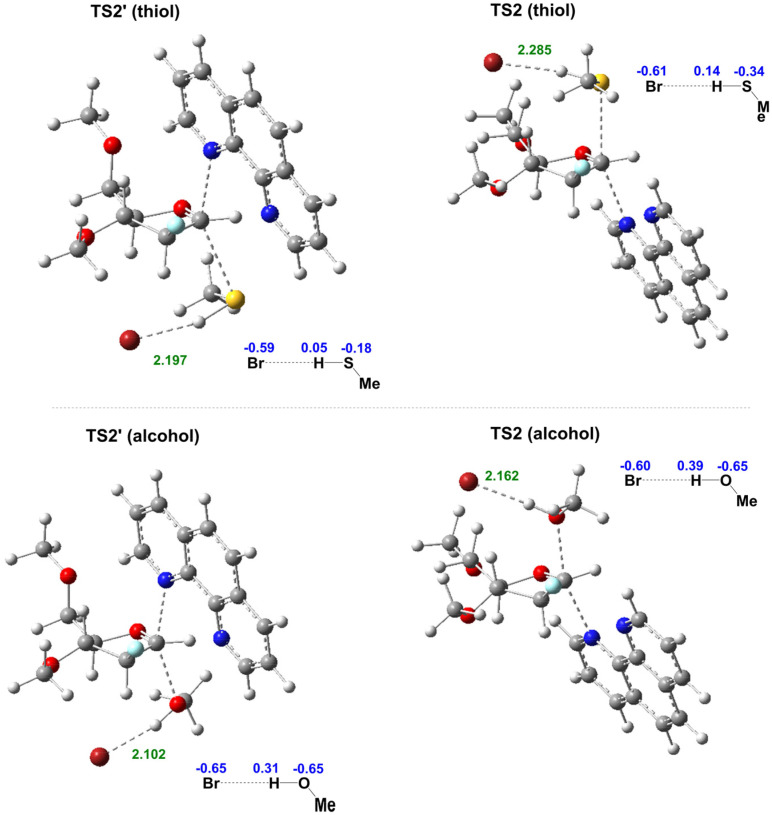
Optimized transition state structure of TS2 and TS2′ with thiol and alcohol nucleophiles. Crucial hydrogen bonding distances are reported in Å (green), and the electrostatic charges on Br^−^ and nucleophiles are shown in blue.

A more detailed analysis of transition states TS2 for alcohol and thiol reveals numerous hydrogen bonding interactions (Fig. S8†). These interactions involve (a) phenanthroline nitrogen and the sugar C1-anomeric proton, (b) bromide ion and MeOH/MeSH, (c) bromide ion and the sugar's hydrogens, and (d) phenanthroline H and sugar ring oxygen atom. The interaction between the bromide ion and the proton from MeOH/MeSH influences the reactivity of alcohol and thiol acceptors. Although alcohols are generally less acidic than thiols, MeOH forms a stronger hydrogen bond with Br^−^ than MeSH (MeSH⋯Br^−^ + MeOH → MeSH + MeOH⋯Br^−^, Δ*G* = −4.2 kcal mol^−1^). Because oxygen is more electronegative than sulfur, the proton attached to alcohol is more electropositive than the one attached to thiol^35^ (electrostatic charge calculations show a charge of 0.39 on the alcohol's H atom, compared to 0.14 on the thiol's H atom in TS2, Fig. 4). Consequently, the interaction between alcohol and bromide ion forms a stronger hydrogen bond than between thiol and bromide ion. The calculated Br^−^⋯H–OMe distance in TS2 (2.162 Å) is shorter than in Br⋯H–SMe distance (2.285 Å). These findings are consistent with the experimental NMR titration results (Fig. S5 and S6†). Cumulative analysis underscores a stabilized transition state in the case of alcohol, contributing to enhanced reactivity. This is further manifested in the transition state (TS2) barriers for the major product formations, β-*O*-furanoside and β-*S*-furanoside. The kinetic barriers for forming β-*O*-furanoside and β-*S*-furanoside are 25.5 and 29.2 kcal mol^−1^, respectively (Fig. 3). The observed difference of 3.7 kcal mol^−1^ between these two transition states provides support for the increased reactivity of alcohol. We also calculated the energy profiles without the bromide ion to better understand the impact of hydrogen bonding on the higher reactivity of alcohols compared to thiols (Fig. S9†). We observed that the TS2 barriers for both thiol and alcohol become unfavorable. This highlights the role of bromide in accepting the proton from the nucleophile in this model for the reaction mechanism. Additionally, the energy gap between the transition states TS2 for the formation of β-*S*-furanoside and β-*O*-furanoside decreased from 3.7 kcal mol^−1^ (with bromide) to 0.04 kcal mol^−1^ (without bromide). This reduction highlights the difference in bromide hydrogen bonding to MeOH *versus* MeSH, which accounts for the higher reactivity observed with alcohol compared to thiol.

## Conclusion

Our study presents a catalytic stereoselective furanosylation method producing *S*-furanosides and *S*-furanosyl peptides in good yields with high levels of 1,2-*cis* stereoselectivity. A significant finding is that thiols exhibit lower reactivity than alcohols under phenanthroline-catalyzed conditions. Despite the lower reactivity, thiol nucleophiles display high levels of stereoselectivity, forming 1,2-*cis S*-furanoside products, regardless of the anomeric composition of furanosyl bromide donors and their stereochemical structure. Conversely, alcohols exhibit higher reactivity but lower stereoselectivity, yielding *O*-furanoside products with reduced stereoselectivity. Moreover, the 1,2-*cis* stereoselectivity of *O*-furanoside products highly depends on their structures. The bromide ion generated through the displacement of an activated electrophilic bromide with the phenanthroline catalyst influences the reactivity and selectivity differences between thiol and alcohol. Our computational studies provide insights into how the bromide anion forms a stronger hydrogen bond interaction with alcohol-OH compared to thiol-SH, leading to lower kinetic energy barriers in the case of *O*-furanoside and, therefore, higher reactivity with alcohol nucleophiles. The kinetic profiles and DFT calculations indicate that the reaction rate with alcohol is faster than that of thiol. Additionally, computational studies, NMR titration studies with TBAB, and control experiments with acid scavengers DTBMP and IBO suggest that the bromide ion enhances the stereoselectivity of thiol over alcohol. Our research advances our understanding of stereoselective glycosylation reactions and provides insights for the design of catalysts and reaction conditions for the stereoselective synthesis of 1,2-*cis S*-linked furanosides.

## Conflicts of interest

The authors declare no financial interest.

## Supplementary Material

OB-023-D4OB01593B-s001

OB-023-D4OB01593B-s002

## Data Availability

The data that support the findings of this study are available in the ESI† of this article.
