# Supplementary material for: Why is thiol unexpectedly less reactive but more selective than alcohol in phenanthroline-catalyzed 1,2-cis O- and S-furanosylations?
Source: Org Biomol Chem. 2024 Nov 18;23(2):328–42. doi: 10.1039/d4ob01593b (PMC11582804; doi:10.1039/d4ob01593b)
Supplement: OB-023-D4OB01593B-s001 [file OB-023-D4OB01593B-s001.pdf]

Supplementary Information (SI) for Organic & Biomolecular Chemistry.
This journal is © The Royal Society of Chemistry 2024

Scheme 1. Stereoselective construction of β-*S*-linked pyranosides and furanosides

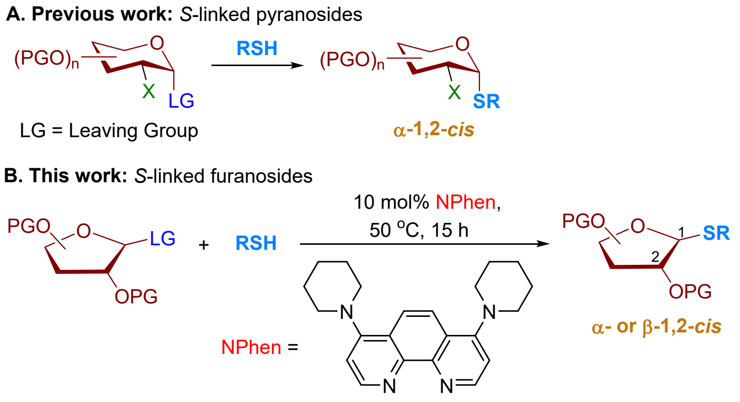

Scheme 2. Preliminary studies with readily available 4,7-diphenyl-1,10-phenanthroline (BPhen) catalyst

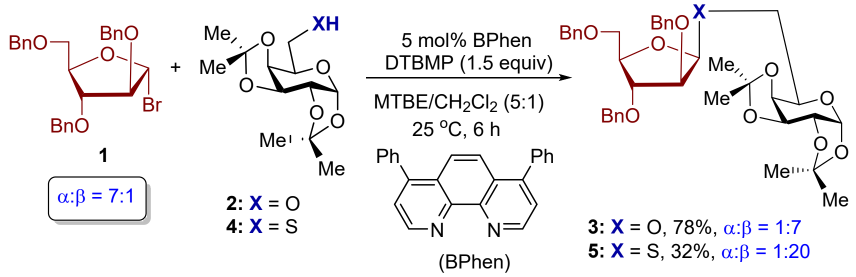

Table 1. Evaluation of Phenanthroline Catalysts for Stereoselective *S*-Furanosylation.

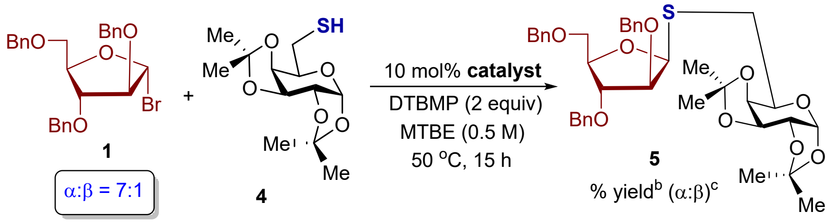

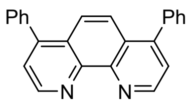

BPhen

69% (α:β = 1:20)

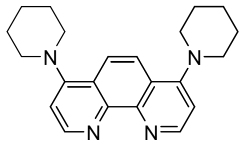

NPhen

79% (α:β = 1:25)

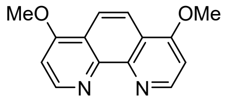

MeOPhen

71% (α:β = 1:20)

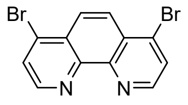

BrPhen

76% (α:β = 1:15)

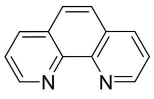

Phen

69% (α:β = 1:17)

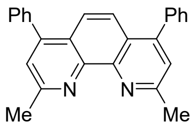

MePhen

50% (α:β = 1:14)

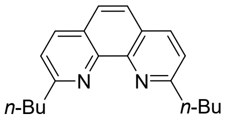

***n*-BuPhen**

74% (α:β = 1:6)

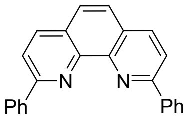

PhPhen

71% (α:β = 1:15)

Scheme 3. Reactivity and stereoselectivity differences between *O*-furanosides and *S*-furanosides

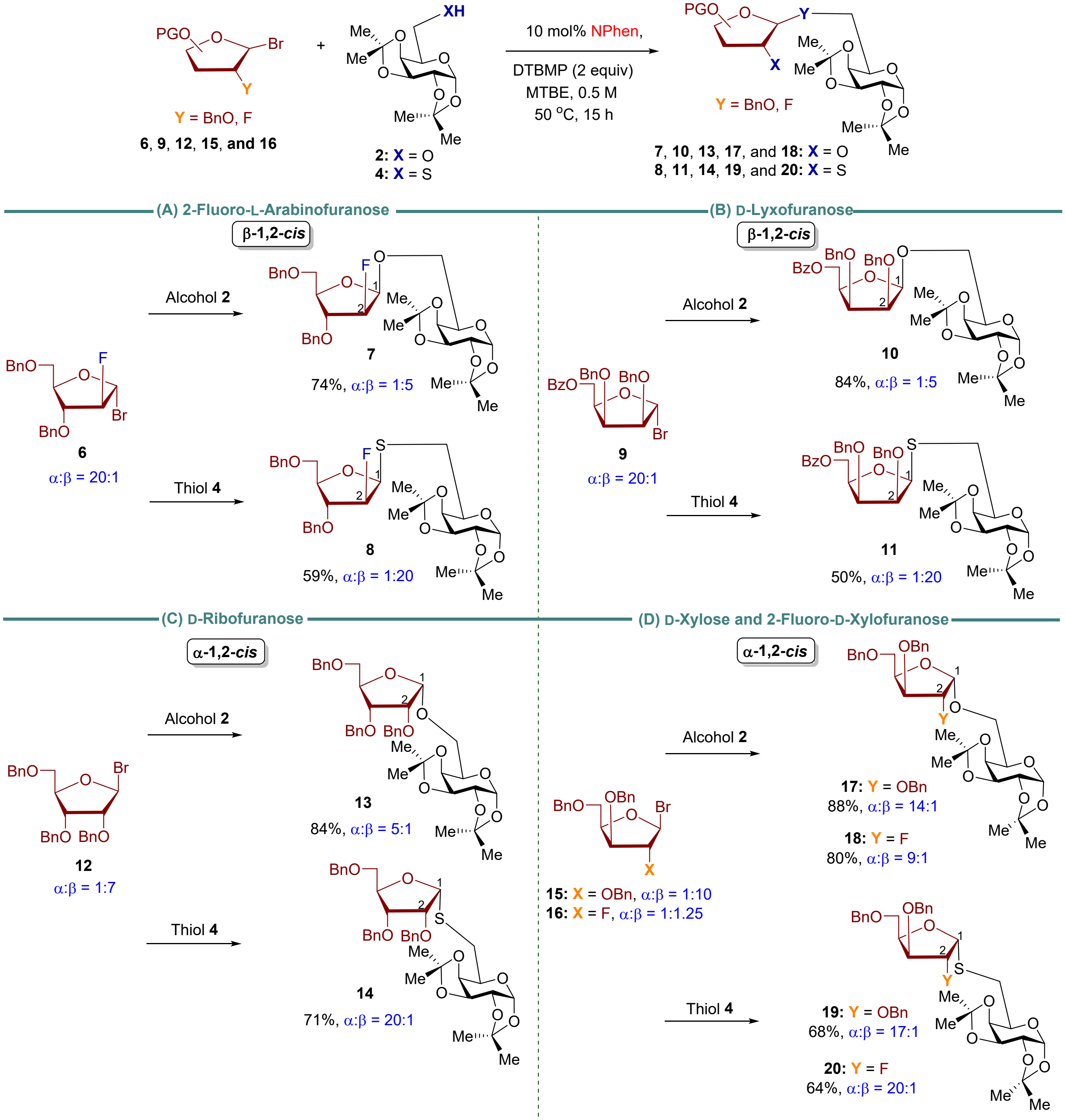

Table 2. Reaction of thiol nucleophiles with L-arabinofuranosyl bromide

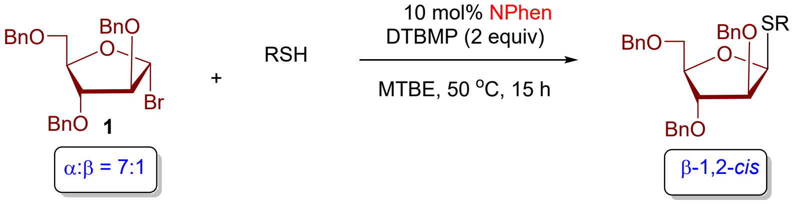

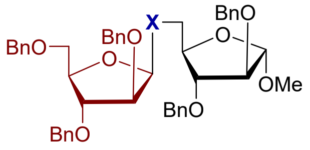

21: **X** = S 55% (α:β = 1:20)
22: **X** = O 88% (α:β = 1:5)

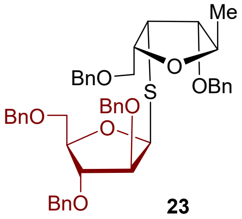

23
57% (α:β = 1:20)

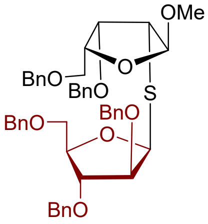

24
55% (α:β = 1:15)

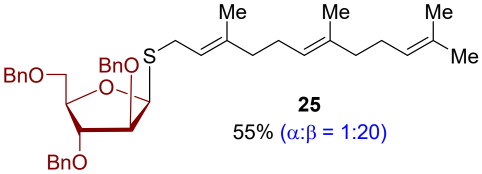

25
55% (α:β = 1:20)

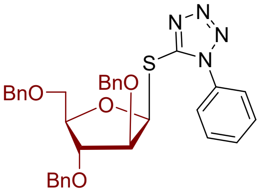

26
77% (α:β = 1:20)

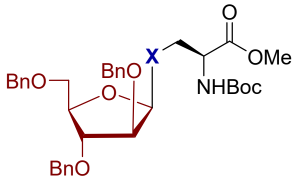

27: **X** = S 78% (α:β = 1:25)
28: **X** = O 84% (α:β = 1:1)

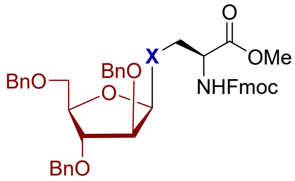

29: **X** = S 72% (α:β = 1:20)
30: **X** = O 75% (α:β = 1:4)

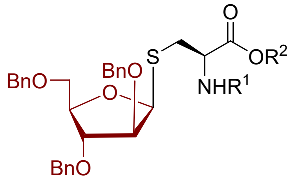

31: R^1 = Boc, R^2 = Allyl 58% (α:β = 1:25)
32: R^1 = Fmoc, R^2 = Allyl 70% (α:β = 1:15)

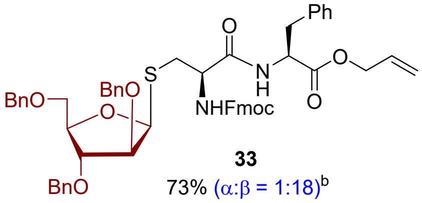

33
73% (α:β = 1:18)[b]

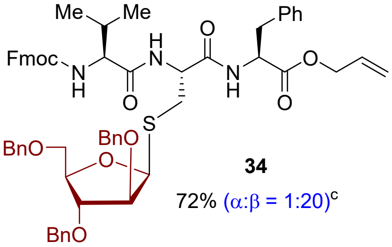

34
72% (α:β = 1:20)[c]

Table 3. Reaction of furanosyl and pyranosyl bromide donor with cysteine residues

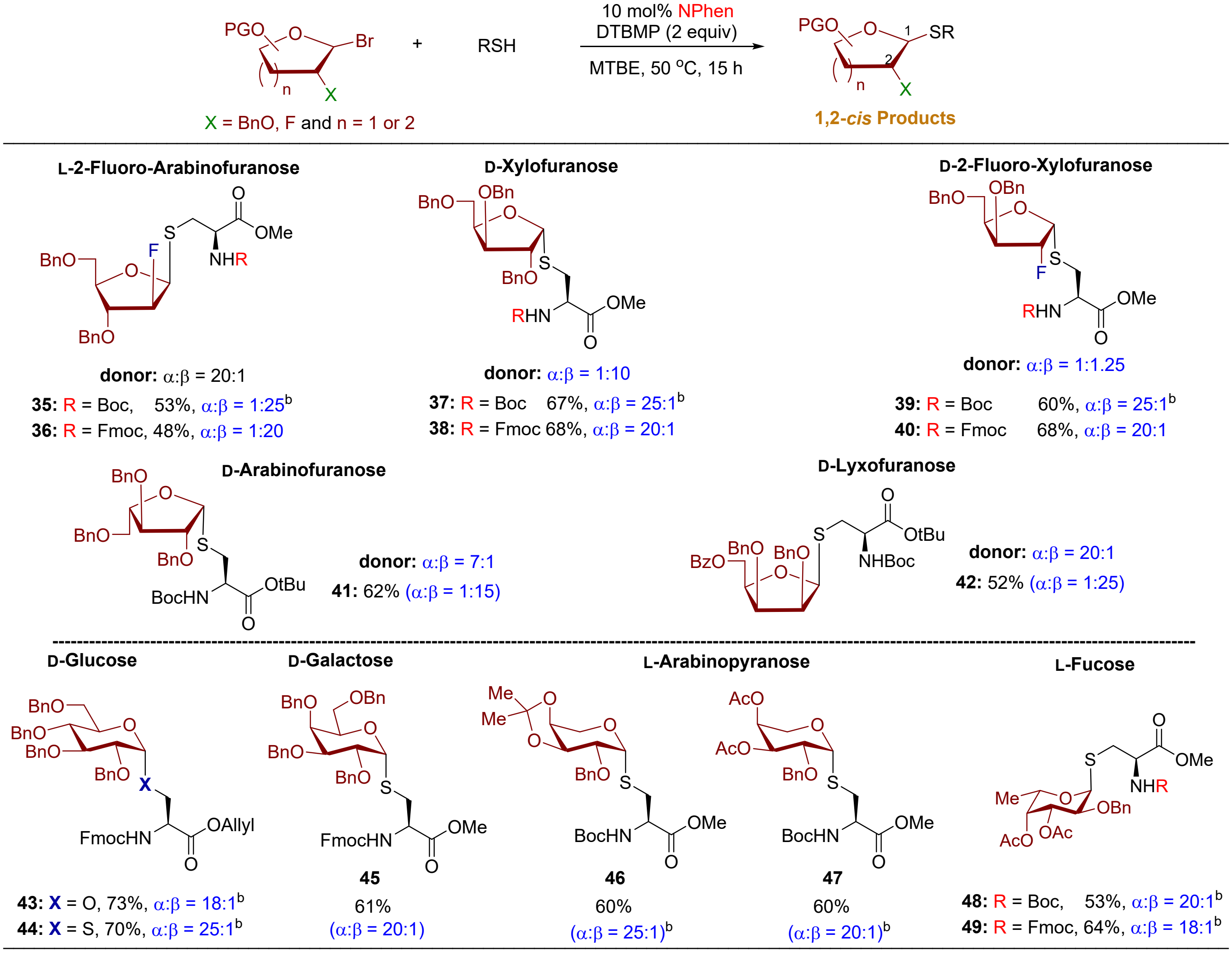

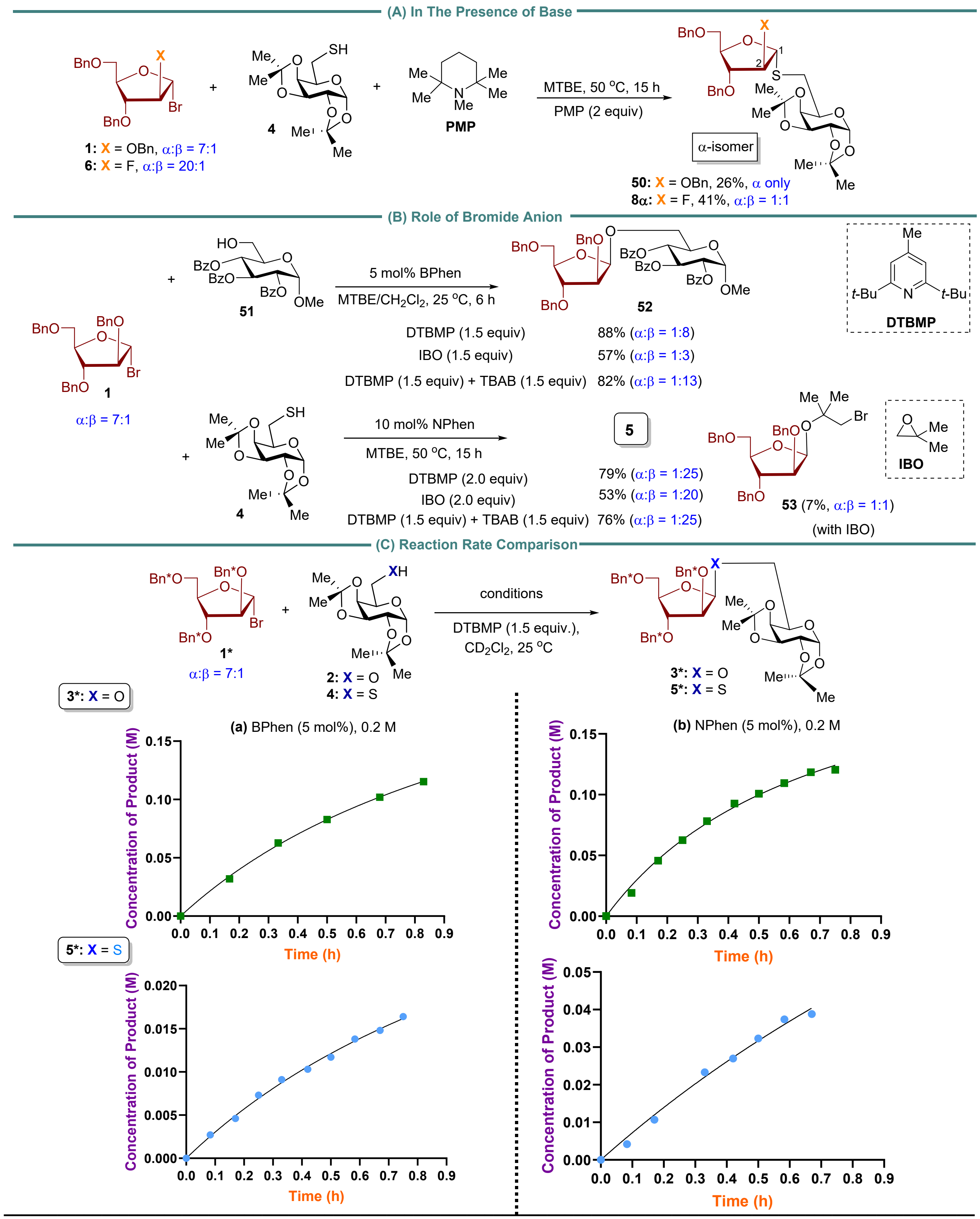

Figure 2. (A) Control experiment with the bulk base, penta-methylpiperidine (PMP). (**B**) Studies the role of bromide ion in influencing reaction selectivity difference between alcohol and thiol. (**C**) Reaction rate comparison between alcohol and thiol
