# Supplementary material for: Why is thiol unexpectedly less reactive but more selective than alcohol in phenanthroline-catalyzed 1,2-cis O- and S-furanosylations?
Source: Org Biomol Chem. 2024 Nov 18;23(2):328–42. doi: 10.1039/d4ob01593b (PMC11582804; doi:10.1039/d4ob01593b)
Supplement: OB-023-D4OB01593B-s002 [file OB-023-D4OB01593B-s002.pdf]

Supplementary Information (SI) for Organic & Biomolecular Chemistry.
This journal is © The Royal Society of Chemistry 2024

Supporting Information

Why Is Thiol Unexpectedly Less Reactive but More Selective Than Alcohol in Phenanthroline-Catalyzed 1,2-*cis* *O*- and *S*-Furanosylations?

Boddu S. Ramakrishna,[†] *Neha Rani,*[†] *Hengfu Xu, Cyrus Alan-Lee, H. Bernhard Schlegel,* and Hien M. Nguyen**

Department of Chemistry, Wayne State University, Detroit, Michigan 48202, United States.
Email: hbs@chem.wayne.edu (H. B. Schlegel) and hmnguyen@wayne.edu (H. M. Nguyen)

Contents

1. General Information S2
2. Experimental details and data S3
   2.1 General Procedure for Preparation of Glycosyl Bromide Donors S3
   2.2 Preparation of Thiol Acceptors S4
   2.3 Optimization S9
   2.4 General Procedure for Phenanthroline-Catalyzed Furanosylation Reaction S10
3. Kinetic Experiments S40
   3.1 Preparation of 4-methylphenyl 1-thio-, 2,3,5-tri-*O*-benzyl-d_7-a-D-arabinofuranoside S40
   3.2 Kinetic Experiment Setup and Data Acquisition S41
   3.3 Initial Rate Comparison Between Alcohol and Thiol Acceptor S43
   3.4 ^{1}H NMR Data for Titration Experiments S46
4. Computational Study S48
5. References S82
6. Spectral Data S83

1. General Information

Methods and Reagents

All reactions were conducted in oven-dried Schlenk flasks equipped with glass stoppers under a positive pressure of argon/nitrogen. Organic solutions were concentrated via rotary evaporation below 40 °C at 25 torr. Progress of the reactions was routinely monitored using analytical thin-layer chromatography (TLC) on pre-coated glass plates with silica gel 60 F254 from Sigma Aldrich. Visualization was achieved using UV light, ceric ammonium molybdate, or 5% sulfuric acid in methanol. Flash chromatography was carried out on silica gel flash chromatography columns or a Biotage Isolera One system utilizing normal phase pre-column cartridges and a Sfär Silica HC D 10 g column. The α:β ratio was determined by ^{1}H NMR. Dry solvents were obtained from an SG Waters solvent system employing activated alumina columns under argon pressure or purchased from Sigma-Aldrich in sealed bottles. Unless specified otherwise, all other reagents were utilized as received from suppliers such as Sigma Aldrich, Alfa Aesar, Acros Organics, TCI, Ambeed, and AA Blocks.

Instrumentation

All ^{1}H NMR spectra were recorded on a 400 MHz, 500 MHz, or 600 MHz spectrometer. All ^{13}C NMR spectra were recorded on a 101 MHz or 126 MHz NMR spectrometer. All proton-decoupled fluorine (19F) NMR spectra were recorded on either a (471 MHz) NMR spectrometer or a DRX-400 (376 MHz) spectrometer. Chemical shifts are expressed in parts per million (δ scale) and are referenced to residual $CHCl_3$ (^{1}H: δ 7.26 ppm, ^{13}C: δ 77.16 ppm), CD_2Cl_2 (^{1}H: δ 5.32 ppm, ^{13}C: δ 53.84 ppm) in the NMR solvent. Data are presented: chemical shift, multiplicity (s = singlet, d = doublet, t = triplet, q = quartet, m = multiplet, and bs = broad singlet), integration, and coupling constant in hertz (Hz). High-resolution mass spectrometry utilizing electrospray ionization (TOF ESI+) was performed to confirm the identity of the compounds at the Wayne State University Lumigen Center.

2. Experimental Details and Data

2.1 General Procedure for Preparation of Glycosyl Bromide Donors

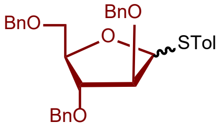

1A

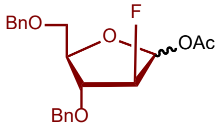

1B

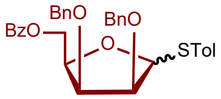

1C

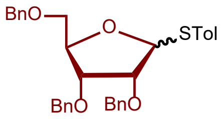

1D

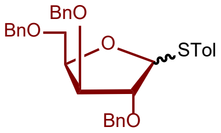

1E

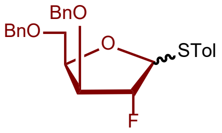

1F

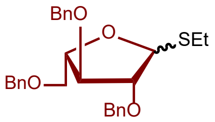

1G

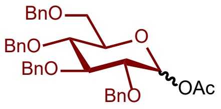

1H

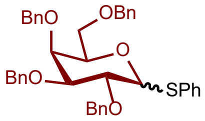

1I

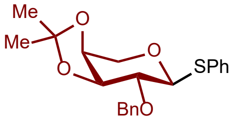

1J

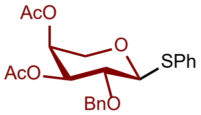

1K

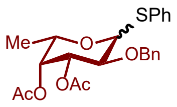

1L

General Procedure 1: A 10 mL oven-dried Schlenk flask was charged with thioglycoside (0.2 mmol, 1 equiv) and dried dichloromethane (DCM) (2 mL, 0.1 M). The solution was cooled to 0 °C in an ice bath, and Br_2 (20 µL, 0.4 mmol, 2 equiv) was added. The resulting solution was stirred in an ice bath and monitored by TLC. Upon completion (~30 min), the reaction mixture was quenched with cyclohexene and then concentrated in *vacuo* to give glycosyl bromide as pale-yellow syrup. The crude glycosyl bromide donor was used without further purification.[1]

General Procedure 2: A 25 mL round bottle flask was charged with glycosyl acetate (0.2 mmol, 1 equiv) and dichloromethane (DCM) (2 mL, 0.1 M). The solution was cooled to 0 °C in an ice bath, and 33% HBr/acetic acid (105 µL, 0.6 mmol, 3 equiv) was added to the flask. The resulting solution was stirred at 0 °C for 1 h under an argon balloon. The reaction was monitored by TLC. Upon completion, the reaction mixture was diluted with ethyl acetate, washed with water, saturated with aqueous $NaHCO_3$ (3x), brine, and dried over Na_2SO_4. The organic layer was concentrated in *vacuo*, and the crude glycosyl bromide donor was used for the next step without further purification.[2]

2.2 Preparation of Thiol Acceptors

2.2.1 Preparation of Glycosyl Primary Thiols

The literature procedure[3] was adopted for the synthesis of glycosyl primary thiols from the corresponding alcohols, and the characterization data is as follows.

((3a*R*,5*S*,5a*R*,8a*S*,8b*R*)-2,2,7,7-tetramethyltetrahydro-5*H*-bis([1,3]dioxolo)[4,5-*b*:4',5'-*d*]pyran-5-yl)methanethiol

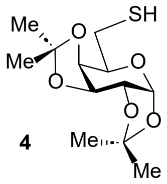

4

The desired product **4** was obtained as a light-yellow oil (562 mg, 70%). The ^{1}H NMR of compound **4** matches with the literature report.[4]

^{1}H NMR (500 MHz, $CDCl_3$) δ 5.52 (d, *J* = 5.0 Hz, 1H), 4.62 (dd, *J* = 7.9, 2.6 Hz, 1H), 4.35 (dd, *J* = 7.9, 2.4 Hz, 1H), 4.31 (dd, *J* = 5.2, 2.6 Hz, 1H), 3.78 (t, *J* = 6.9 Hz, 1H), 2.76 (dt, *J* = 14.0, 7.3 Hz, 1H), 2.67 (ddd, *J* = 13.5, 9.9, 6.7 Hz, 1H), 1.61 (dd, *J* = 9.8, 7.3 Hz, 1H), 1.54 (s, 3H), 1.44 (s, 3H), 1.34 (d, *J* = 7.1 Hz, 6H).

((2*S*,3*S*,4*S*,5*S*)-3,4-bis(benzyloxy)-5-methoxytetrahydrofuran-2-yl)methanethiol

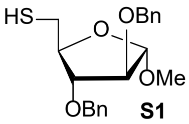

S1

The desired product **S1** was obtained as a colorless oil (82 mg, 91%).

^{1}H NMR (500 MHz, $CDCl_3$) δ 7.39 – 7.27 (m, 10H), 4.93 – 4.90 (m, 1H), 4.63 – 4.47 (m, 4H), 4.13 (td, *J* = 6.2, 4.8 Hz, 1H), 4.00 (dd, *J* = 3.0, 1.2 Hz, 1H), 3.89 (ddd, *J* = 6.4, 3.0, 0.6 Hz, 1H), 3.38 (s, 3H), 2.79 (ddd, *J* = 13.8, 8.2, 4.8 Hz, 1H), 2.69 (ddd, *J* = 13.9, 8.2, 6.0 Hz, 1H), 1.54 (t, *J* = 8.2 Hz, 1H).

^{13}C NMR (126 MHz, $CDCl_3$) δ 137.86, 137.59, 128.63, 128.60, 128.12, 128.09, 128.07, 128.05, 107.34, 88.46, 85.12, 82.02, 72.39, 72.21, 55.03, 27.23.

HRMS (ESI): calc. for $C_{20}H_{24}O_4NaS$ $[M+Na]^+$: 383.1288; found: 383.1281.

2.2.2 Preparation of Glycosyl Secondary Thiols

(2*S*,3*S*,4*S*,5*R*)-4-(benzyloxy)-5-((benzyloxy)methyl)-2-methoxytetrahydrofuran-3-thiol

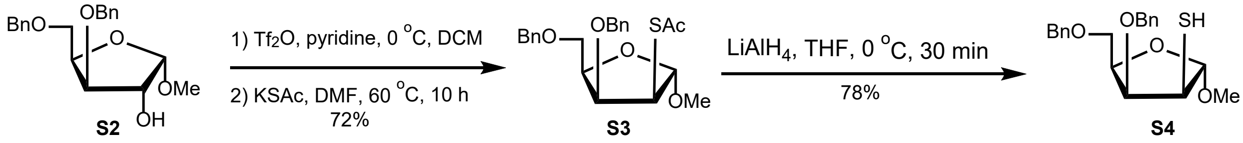

To a 250 mL oven-dried flask was charged with compound **S2**[5] (1.8 g, 5.2 mmol, 1 equiv) in dried dichloromethane (36 mL). The solution was cooled to 0 ºC, and triflic anhydride (1.08 mL, 6.3 mmol, 1.2 equiv) and pyridine (0.85 mL, 10.4 mmol, 2 equiv) were added. The reaction mixture was allowed to reach room temperature and stirred for 1 h. The reaction was monitored by TLC. Upon completion, the mixture was quenched with cold aqueous 1M HCl solution, extracted with DCM, washed with saturated aqueous $NaHCO_3$ and brine, and dried over anhydrous sodium sulfate. The crude intermediate was concentrated to dryness and used for the next step without further purification.

The crude intermediate was then dissolved in DMF (60 mL), and potassium thioacetate (0.9 g, 7.8 mmol, 1.5 equiv) was added. The resulting mixture was stirred at 60 ºC for 10 h. The reaction mixture was extracted with ethyl acetate (200 mL), washed with brine, and evaporated to dryness. The crude was subjected to silica gel column chromatography (1:3, EtOAc/hexanes) to afford compound **S3** (1.5 g, 72%) as a syrup. R_f 0.4 (1:2, EtOAc/hexanes); **^{1}H NMR** (500 MHz, $CDCl_3$) δ 7.35 – 7.26 (m, 10H), 4.94 (d, J = 3.2 Hz, 1H), 4.59 (t, J = 11.5 Hz, 2H), 4.51 (d, J = 12.0 Hz, 1H), 4.44 (d, J = 11.5 Hz, 1H), 4.43 – 4.37 (m, 2H), 4.18 (dd, J = 5.5, 3.3 Hz, 1H), 3.71 (dd, J = 10.0, 5.2 Hz, 1H), 3.68 – 3.63 (m, 1H), 3.38 (s, 3H), 2.35 (s, 3H). **^{13}C NMR** (126 MHz, $CDCl_3$) δ 194.85, 138.19, 137.65, 128.51, 128.49, 127.97, 127.95, 127.79, 108.44, 79.09, 78.75, 74.06, 73.67, 68.99, 55.88, 51.88, 30.65. **HRMS (ESI)**: calc. for $C_{22}H_{26}O_5NaS$ $[M+Na]^+$: 425.1393; found: 425.1388.

To a solution of **S3** (500 mg, 1.24 mmol, 1 equiv) in dried THF (12 mL) was added $LiAlH_4$ (377 mg, 9.94 mmol, 8 equiv) at 0 ºC. After the resulting mixture was stirred at rt for 30 min, it was diluted with ethyl acetate. The mixture was diluted with DCM, washed with 1 N HCl, and then brine. The organic layer was dried over Na_2SO_4, filtered, and concentrated. The residue was subjected to flash silica gel column chromatography (1:7, EtOAc/hexanes) to give **S4** (350 mg, 78%) as a syrup. R_f 0.33 (1:4, EtOAc/hexanes); **^{1}H NMR** (500 MHz, $CDCl_3$) δ 7.36 – 7.27 (m,

10H), 4.92 (d, J = 3.8 Hz, 1H), 4.72 (d, J = 11.2 Hz, 1H), 4.62 – 4.50 (m, 3H), 4.35 (td, J = 6.1, 4.5 Hz, 1H), 4.19 (t, J = 5.0 Hz, 1H), 3.73 (qd, J = 9.9, 6.1 Hz, 2H), 3.40 (s, 3H), 3.33 (ddd, J = 9.5, 5.5, 3.8 Hz, 1H), 2.01 (d, J = 9.8 Hz, 1H). **^{13}C NMR** (126 MHz, $CDCl_3$) δ 138.13, 137.68, 128.56, 128.54, 128.16, 128.05, 128.00, 127.85, 111.82, 79.76, 79.42, 74.33, 73.72, 68.90, 56.23, 47.57. **HRMS (ESI)**: calc. for $C_{20}H_{24}O_4NaS$ $[M+Na]^+$: 383.1288; found: 383.1283.

(2*R*,3*S*,4*R*,5*R*)-4-(benzyloxy)-2-((benzyloxy)methyl)-5-methyltetrahydrofuran-3-thiol

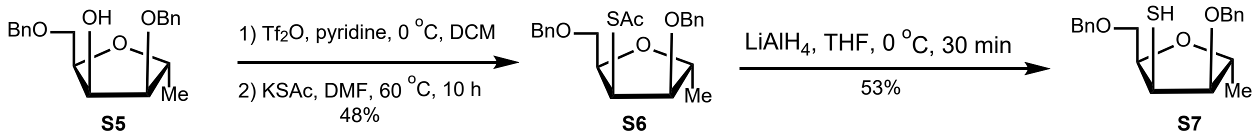

The procedure described above for synthesizing glycosyl acceptor **S4** was adopted with compound **S5**[6] to afford compounds **S6** and **S7**. The characterization data for compounds **S6** and **S7** were reported as follows:

Compound **S6** (0.11 g, 48%) was obtained as a syrup. R_f 0.3 (1:4, EtOAc/hexanes); **^{1}H NMR** (500 MHz, $CDCl_3$) δ 7.37 – 7.27 (m, 10H), 4.70 (d, J = 11.8 Hz, 1H), 4.61 – 4.52 (m, 3H), 4.19 – 4.11 (m, 2H), 3.98 (dd, J = 5.7, 4.6 Hz, 1H), 3.70 – 3.59 (m, 3H), 2.34 (s, 3H), 1.27 (d, J = 6.4 Hz, 3H). **^{13}C NMR** (126 MHz, $CDCl_3$) δ 194.42, 138.33, 138.00, 128.53, 128.46, 127.89, 127.86, 127.68, 89.37, 82.83, 79.45, 73.57, 72.23, 71.06, 47.87, 30.65, 19.32. **HRMS (ESI)**: calc. for $C_{22}H_{26}O_4NaS$ $[M+Na]^+$: 409.1444; found: 409.1438.

Compound **S7** (48 mg, 53%) was obtained as a colorless oil. R_f 0.25 (1:2, EtOAc/hexanes); **^{1}H NMR** (500 MHz, $CDCl_3$) δ 7.38 – 7.27 (m, 10H), 4.78 (d, J = 11.7 Hz, 1H), 4.68 (d, J = 11.7 Hz, 1H), 4.65 – 4.55 (m, 2H), 4.08 – 4.03 (m, 1H), 3.98 (ddd, J = 8.5, 4.8, 3.9 Hz, 1H), 3.66 – 3.58 (m, 3H), 3.31 (td, J = 8.0, 6.9 Hz, 1H), 1.77 (d, J = 7.6 Hz, 1H), 1.30 (d, J = 6.3 Hz, 3H). **^{13}C NMR** (126 MHz, $CDCl_3$) δ 138.23, 138.00, 128.61, 128.53, 128.04, 127.96, 127.84, 127.79, 92.54, 84.43, 78.65, 73.67, 72.95, 70.03, 43.79, 19.65. **HRMS (ESI)**: calc. for $C_{20}H_{24}O_3NaS$ $[M+Na]^+$: 367.1338; found: 367.1334.

2.2.3 Preparation of Cysteine Containing Acceptors

Allyl (((9*H*-fluoren-9-yl)methoxy)carbonyl)-L-cysteinyl-L-phenylalaninate

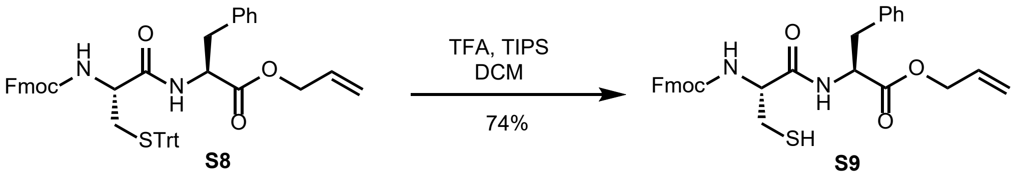

The compound **S8**[7] (350 mg, 0.46 mmol, 1 equiv) was dissolved in dry dichloromethane (2 mL). TIPS (0.31 mL, 1.5 mmol, 3.3 equiv) was then added to the solution, followed by dropwise addition of TFA (2 mL) under argon atmosphere at room temperature. The reaction mixture was allowed to stir at room temperature for 1-2 h. The reaction mixture was monitored by TLC. Upon completion, the mixture was concentrated to give a gummy crude product. Diethyl ether (10 vol) was then added, and the solids precipitated were filtered using sintered funnel and washed again with diethyl ether to get pure compound **S9** (180 mg, 74%) as colorless solid; R_f 0.26 (1:4, EtOAc/hexanes); **^{1}H NMR** (500 MHz, $CDCl_3$) δ 7.78 (d, J = 7.6 Hz, 2H), 7.58 (d, J = 7.6 Hz, 2H), 7.41 (t, J = 7.5 Hz, 2H), 7.33 (t, J = 7.4 Hz, 2H), 7.29 – 7.18 (m, 3H), 7.11 (d, J = 7.0 Hz, 2H), 6.67 – 6.50 (m, 1H), 5.87 (td, J = 10.7, 5.2 Hz, 1H), 5.66 – 5.57 (m, 1H), 5.29 (dd, J = 26.5, 13.8 Hz, 2H), 4.88 (q, J = 7.4, 6.8 Hz, 1H), 4.62 (d, J = 6.1 Hz, 2H), 4.47 (dd, J = 10.6, 6.9 Hz, 1H), 4.37 (d, J = 7.1 Hz, 2H), 4.22 (t, J = 6.9 Hz, 1H), 3.20 (dd, J = 14.0, 5.6 Hz, 1H), 3.14 – 2.94 (m, 2H), 2.67 (s, 1H), 1.52 (d, J = 11.6 Hz, 1H). **^{13}C NMR** (126 MHz, $CDCl_3$) δ 170.86, 169.32, 156.00, 143.78, 141.49, 135.61, 131.41, 129.59, 129.40, 128.84, 128.43, 127.96, 127.46, 127.26, 126.43, 125.12, 120.21, 119.46, 67.43, 66.41, 56.01, 53.51, 47.28, 37.93, 27.02. **HRMS (ESI)**: calc. for $C_{30}H_{31}O_5N_2S$ $[M+H]^+$: 531.1948; found: 531.1958.

Allyl (((9*H*-fluoren-9-yl)methoxy)carbonyl)-L-valyl-L-cysteinyl-L-phenylalaninate

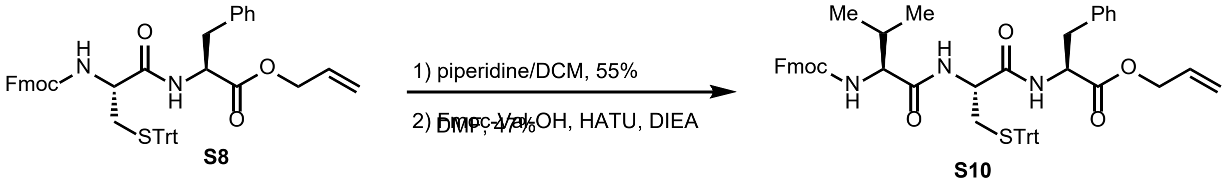

The compound **S8**[7] (2.0 g, 2.59 mmol, 1 equiv) was dissolved in dried dichloromethane (21 mL). Piperidine (5.2 mL, 52.6 mmol, 20 equiv) was added to the solution. The reaction mixture was allowed to stir at room temperature for 3 h. The reaction mixture was monitored by TLC. Upon completion, the mixture was concentrated. The crude product was subjected to flash silica gel column chromatography (1:30, MeOH/DCM) and was used in the next step. To a stirred solution of Fmoc-Val-OH (475 mg, 1.4 mmol, 1.1 equiv) in dried DMF (8 mL) was added HATU (726 mg,

1.91 mmol, 1.5 equiv), DIPEA (0.33 mL, 1.91 mmol, 1.5 equiv) followed by H-Cys(Trt)-Phe-OAllyl (700 mg, 1.27 mmol, 1 equiv) at room temperature under nitrogen atmosphere. The reaction mixture was stirred for 3 to 6 h (monitored by TLC). Upon completion, the reaction mixture was quenched with water and extracted with EtOAc (3× 50 mL). The combined organic layer was dried over anhydrous Na_2SO_4 and evaporated to dryness. The obtained crude product was purified by flash silica gel chromatography to get pure compound **S10** (920 mg, 83%) as colorless solid; R_f 0.34 (1:2, EtOAc/hexanes); **^{1}H NMR** (500 MHz, $CDCl_3$) δ ^{1}H NMR (500 MHz, $CDCl_3$) δ 7.86 (d, J = 7.5 Hz, 2H), 7.67 (t, J = 6.4 Hz, 2H), 7.50 (d, J = 7.9 Hz, 8H), 7.38 – 7.27 (m, 14H), 7.17 (d, J = 7.5 Hz, 2H), 6.45 (d, J = 8.0 Hz, 1H), 6.00 (d, J = 7.1 Hz, 1H), 5.90 (td, J = 10.8, 5.3 Hz, 1H), 5.40 – 5.25 (m, 3H), 4.87 (d, J = 6.8 Hz, 1H), 4.65 (d, J = 6.5 Hz, 2H), 4.52 (dd, J = 10.8, 7.0 Hz, 1H), 4.42 (t, J = 8.8 Hz, 1H), 4.29 (t, J = 6.9 Hz, 1H), 4.11 – 3.97 (m, 2H), 3.17 (d, J = 6.1 Hz, 2H), 2.86 (dd, J = 13.6, 7.4 Hz, 1H), 2.64 (dd, J = 13.3, 5.6 Hz, 1H), 2.08 (q, J = 7.3, 6.8 Hz, 1H), 0.98 (d, J = 6.9 Hz, 3H), 0.89 (d, J = 6.9 Hz, 3H). **^{13}C NMR** (126 MHz, $CDCl_3$) δ 171.18, 170.58, 169.36, 156.44, 144.43, 144.04, 141.47, 135.70, 131.50, 129.66, 129.52, 128.68, 128.24, 127.87, 127.27, 127.24, 127.09, 125.23, 120.13, 120.12, 119.27, 67.49, 67.18, 66.21, 53.58, 52.39, 47.35, 37.90, 33.44, 31.43, 17.55. **HRMS (ESI)**: calc. for $C_{54}H_{53}O_6N_3S$ $[M+H]^+$: 872.3728; found: 872.3751.

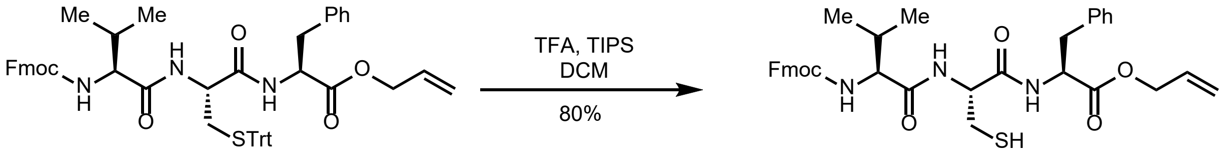

S10 S11

The compound **S10** (350 mg, 0.4 mmol, 1 equiv) was dissolved in dry dichloromethane (4 mL). TIPS (0.16 mL, 0.8 mmol, 2 equiv) was added to the solution, followed by the dropwise addition of TFA (2 mL) under an argon atmosphere at room temperature. The reaction mixture was allowed to stir for 1-2 h. The reaction mixture was monitored by TLC. Upon completion, the mixture was concentrated to give a gummy crude product. Diethyl ether (10 vol) was then, and the solids precipitated were filtered using sintered funnel and washed again with diethyl ether to get pure compound **S11** (202 mg, 80%) as colorless solid; R_f 0.12 (1:4, EtOAc/hexanes); **^{1}H NMR** (500 MHz, $CDCl_3$) δ 7.79 (d, J = 7.6 Hz, 2H), 7.60 (d, J = 7.4 Hz, 2H), 7.42 (t, J = 7.6 Hz, 2H), 7.35 – 7.24 (m, 5H), 7.12 (d, J = 7.2 Hz, 2H), 6.85 (dd, J = 44.1, 7.8 Hz, 2H), 5.87 (tt, J = 11.1,

5.5 Hz, 1H), 5.43 (s, 1H), 5.30 (dd, J = 25.0, 13.8 Hz, 2H), 4.89 (d, J = 7.0 Hz, 1H), 4.70 – 4.56 (m, 3H), 4.53 – 4.31 (m, 2H), 4.24 (t, J = 7.2 Hz, 1H), 4.06 (s, 1H), 3.25 – 2.92 (m, 3H), 2.68 (s, 1H), 2.12 (s, 1H), 1.59 (t, J = 9.3 Hz, 1H), 0.94 (s, 6H). **^{13}C NMR** (126 MHz, $CDCl_3$) δ 173.61, 170.72, 170.24, 157.49, 143.48, 141.49, 134.98, 131.02, 129.28, 128.99, 128.07, 127.71, 127.31, 125.11, 120.25, 119.93, 68.21, 66.94, 66.34, 61.11, 55.40, 54.05, 47.07, 37.62, 30.84, 26.29, 19.22, 18.13. **HRMS (ESI)**: calc. for $C_{35}H_{40}O_6N_3S$ $[M+H]^+$: 630.2632; found: 630.2643.

2.3 Optimization Studies

Table **S1**. Optimization of reaction conditions[a]

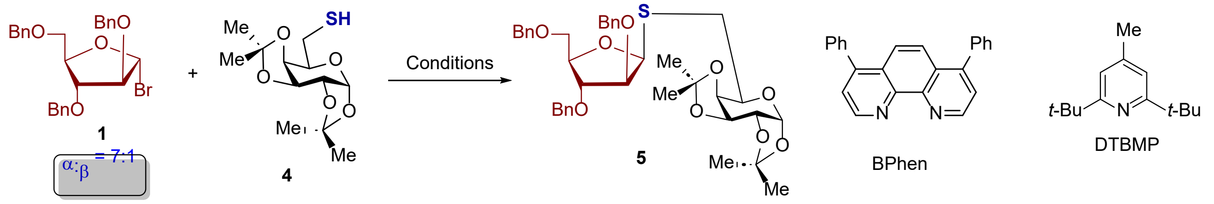

Entry	Solvent	Conc (M)	Catalyst	Catalyst (mol%)	DTMBP (equiv)	Temp (°C)	Time (h)	Yield[b] (%)	α:β[c] ratio
1	MTBE/CH_2Cl_2 (5:1)	0.2	BPhen	5	1.5	25	6	32	1:20
2	MTBE/CH_2Cl_2 (5:1)	0.2	BPhen	5	1.5	25	15	49	1:20
3	MTBE	0.2	BPhen	5	1.5	25	15	50	1:20
4	MTBE	0.2	BPhen	10	1.5	25	15	53	1:20
5	MTBE	0.2	BPhen	10	1.5	50	15	66	1:20
6	MTBE	0.2	BPhen	10	2.0	50	15	63	1:20
7	MTBE	0.5	BPhen	10	2.0	50	15	69	1:20

[a]All reactions were performed with 0.3 mmol of donor **1**, 0.1 mmol of thiol **4**, and 10 mol% of BPhen with respect to donor **1**. [b]Isolated yield. [c]The α:β ratio was determined by 1H NMR analysis.

Scheme S1. Furanosylation of alcohol **2** under optimized conditions for thiol **4**

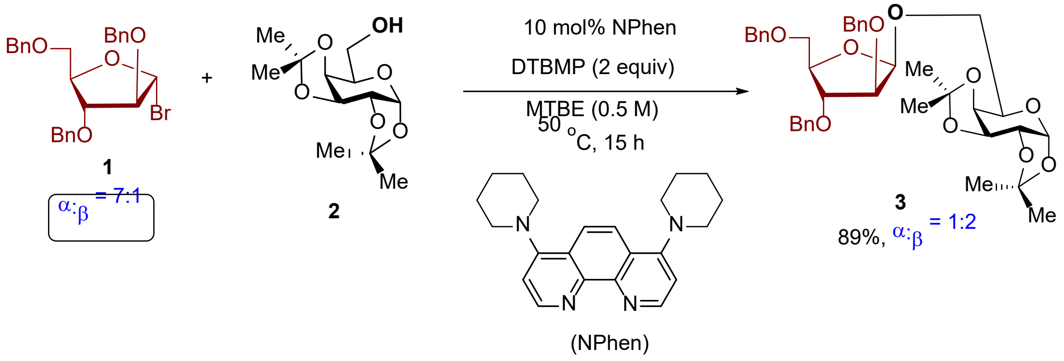

2.4 General Procedure for Phenanthroline-Catalyzed Furanosylation Reaction

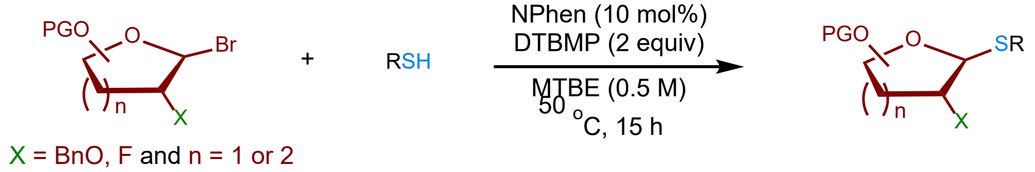

General Procedure A: To an oven-dried 10 mL Schlenk flask was charged with furanosyl bromide (0.2 mmol, 2.0 equiv), thiol (0.1 mmol, 1.0 equiv), 4,7-dipiperidinyl-1,10-phenanthroline (NPhen) (0.02 mmol, 10 mol%), 2,6-di-*tert*-butyl-4-methylpyridine (0.2 mmol, 2.0 equiv) and 0.2 mL (0.5 M) of methyl *tert*-butyl ether (MTBE). The solution was stirred at 50 ºC for 15 h and purified by silica gel flash chromatography to give the desired product. The anomeric configuration of the furanoside products was determined by 1H NMR analysis with 1,2-*cis* products observed coupling constant in the range of 4-4.5 Hz. Whereas, the coupling constant of 1,2-*trans* products was observed in the range of 1-2 Hz.

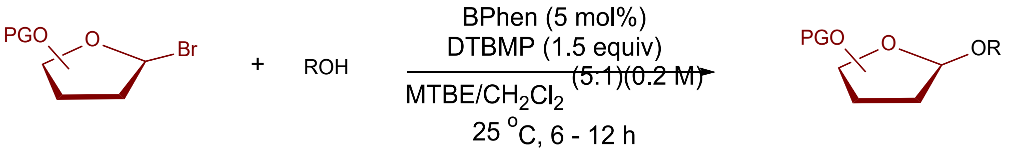

General Procedure B: To 5 mL dried Schlenk flask was charged with furanosyl bromide (0.3 mmol, 3.0 equiv), alcohol (0.1 mmol, 1.0 equiv), bathophenanthroline (BPhen) (0.015 mmol, 5 mol%) with respect to the furanosyl bromide, 2,6-di-*tert*-butyl-4-methylpyridine (0.15 mmol, 1.5 equiv) and 0.5 mL (0.2 M) of a mixture of methyl *tert*-butyl ether and methylene chloride (5:1). The resulting solution was stirred at room temperature for 6 h, and purified by silica gel flash chromatography to give the desired product.

Note: The anomeric configuration of the furanoside products was determined by ^{1}H NMR analysis. The coupling constant of 1,2-*cis* products was observed in the range of 4-4.5 Hz, whereas that of 1,2-*trans* products was observed in the range of 1-2 Hz.

(3a*R*,5*R*,5a*S*,8a*S*,8b*R*)-5-((((2*R*,3*S*,4*R*,5*R*)-3,4-bis(benzyloxy)-5-((benzyloxy)methyl)tetra-hydrofuran-2-yl)oxy)methyl)-2,2,7,7-tetramethyltetrahydro-5*H*-bis([1,3]dioxolo)[4,5-*b*:4',5'-*d*]pyran

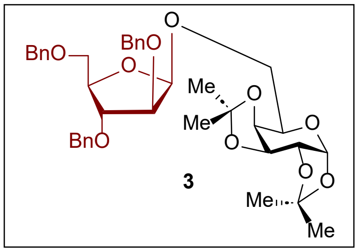

Prepared according to General Procedure **B** at room temperature for 6 h. The desired product **3** was obtained as a colorless oil (51 mg, 78%, α:β 1:7). The ^{1}H NMR of compound **3** matches the literature report.[8]

^{1}H NMR (600 MHz, $CDCl_3$) δ 7.41 – 7.37 (m, 2H), 7.35 – 7.29 (m, 6H), 7.28-7.22 (m, 5H), 5.54 (d, *J* = 5.1 Hz, 1H), 5.21 (d, *J* = 4.2 Hz, 1H), 4.75 (d, *J* = 12.1 Hz, 1H), 4.67 (d, *J* = 11.9 Hz, 1H), 4.61 – 4.49 (m, 5H), 4.29 (dd, *J* = 5.0, 2.4 Hz, 1H), 4.17 (dd, *J* = 7.9, 1.9 Hz, 1H), 4.12 – 4.09 (m, 2H), 4.04 (dd, *J* = 6.4, 4.4 Hz, 1H), 4.00 (td, *J* = 5.9, 1.9 Hz, 1H), 3.75 (d, *J* = 5.9 Hz, 2H), 3.60 (dd, *J* = 10.0, 6.1 Hz, 1H), 3.55 (dd, *J* = 10.0, 4.8 Hz, 1H), 1.43 (s, 3H), 1.41 (s, 3H), 1.30 (s, 3H), 1.29 (s, 3H).

(3a*R*,5*S*,5a*R*,8a*S*,8b*R*)-5-((((2*S*,3*S*,4*R*,5*R*)-3,4-bis(benzyloxy)-5-((benzyloxy)methyl)tetra-hydrofuran-2-yl)thio)methyl)-2,2,7,7-tetramethyltetrahydro-5*H*-bis([1,3]dioxolo)[4,5-*b*:4',5'-*d*]pyran

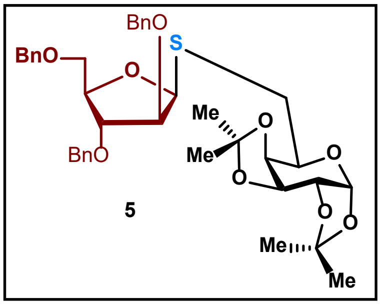

Prepared according to General Procedure **A** at 50 °C for 15 h, the desired product **5** was obtained as a colorless oil (54 mg, 79%, α:β 1:25).

^{1}H NMR (500 MHz, $CDCl_3$) δ 7.38 – 7.22 (m, 15H), 5.56 (d, *J* = 4.9 Hz, 1H), 5.53 (d, *J* = 5.0 Hz, 1H), 4.68 (d, *J* = 11.8 Hz, 1H), 4.61 – 4.56 (m, 2H), 4.55 – 4.49 (m, 4H), 4.31 – 4.27 (m, 2H), 4.18 (t, *J* = 4.5 Hz, 1H), 4.13 (td, *J* = 6.4, 4.3 Hz, 1H), 4.06 (t, *J* = 4.1 Hz, 1H), 3.95 (ddd, *J* = 7.5, 5.7, 1.8 Hz, 1H), 3.73 (dd, *J* = 9.8, 6.3 Hz, 1H), 3.66 (dd, *J* = 10.0, 6.6 Hz, 1H), 3.00 (dd, *J* = 13.8, 7.7 Hz, 1H), 2.82 (dd, *J* = 13.9, 5.5 Hz, 1H), 1.50 (s, 3H), 1.45 (s, 3H), 1.33 (s, 3H), 1.32 (s, 3H).

^{13}**C NMR** (126 MHz, $CDCl_3$) δ 138.39, 138.06, 137.80, 128.51, 128.49, 128.47, 128.07, 127.90, 127.86, 127.83, 127.70, 109.37, 108.73, 96.65, 87.70, 84.39, 84.02, 82.10, 73.44, 72.50, 72.04, 72.01, 71.51, 71.00, 70.72, 69.34, 30.55, 26.26, 26.15, 25.15, 24.54.
HRMS (ESI): calc. for $C_{38}H_{50}O_9NS$ $[M+NH_4]^+$: 696.3206; found: 696.3171.

(3a*R*,5*R*,5a*S*,8a*S*,8b*R*)-5-((((2*R*,3*S*,4*R*,5*R*)-4-(benzyloxy)-5-((benzyloxy)methyl)-3-fluorotetrahydrofuran-2-yl)oxy)methyl)-2,2,7,7-tetramethyltetrahydro-5*H*-bis([1,3]dioxolo)[4,5-*b*:4',5'-*d*]pyran

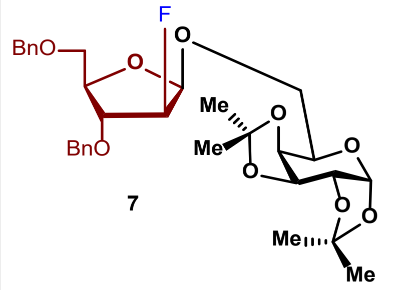

7

Prepared according to General Procedure **A** at 50 °C for 15 h. The desired product **7** was obtained as a colorless oil (43 mg, 74%, α:β 1:5). The ^{1}H NMR of compound **7** matches the literature report.[8]
^{1}**H NMR** (400 MHz, $CDCl_3$) δ 7.37 – 7.24 (m, 10H), 5.52 (d, J = 5.0 Hz, 1H), 5.19 (d, J = 4.3 Hz, 1H), 5.01 (ddd, J = 52.6, 5.9, 4.3 Hz, 1H), 4.69 (d, J = 11.8 Hz, 1H), 4.63 – 4.51 (m, 4H), 4.30 (dd, J = 5.0, 2.4 Hz, 1H), 4.25 – 4.17 (m, 2H), 4.14 (q, J = 5.9 Hz, 1H), 3.98 (ddd, J = 7.1, 5.8, 1.8 Hz, 1H), 3.88 (dd, J = 10.9, 5.6 Hz, 1H), 3.70 (dd, J = 10.9, 6.6 Hz, 1H), 3.65 (dd, J = 10.2, 6.4 Hz, 1H), 3.60 (dd, J = 10.1, 5.8 Hz, 1H), 1.53 (s, 3H), 1.43 (s, 3H), 1.33 (s, 3H), 1.32 (s, 3H).

(3a*R*,5*S*,5a*R*,8a*S*,8b*R*)-5-((((2*S*,3*S*,4*R*,5*R*)-4-(benzyloxy)-5-((benzyloxy)methyl)-3-fluorotetrahydrofuran-2-yl)thio)methyl)-2,2,7,7-tetramethyltetrahydro-5*H*-bis([1,3]dioxolo)[4,5-*b*:4',5'-*d*]pyran

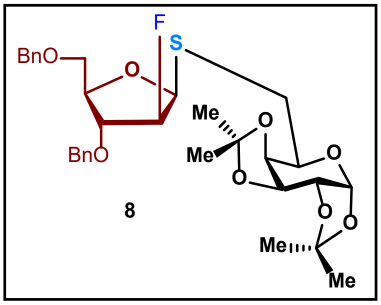

8

Prepared according to General Procedure **A** at 50 °C for 15 h, the desired product **8** was obtained as a colorless oil (35 mg, 59%, α:β 1:20).

^{1}H NMR (500 MHz, $CDCl_3$) δ 7.36 – 7.27 (m, 10H), 5.51 (d, *J* = 5.0 Hz, 1H), 5.31 (dd, *J* = 27.1, 3.3 Hz, 1H), 5.12 – 4.96 (m, 1H), 4.60 (q, *J* = 4.0, 3.5 Hz, 3H), 4.56 (s, 2H), 4.32 (dd, *J* = 8.0, 1.8 Hz, 1H), 4.29 (dd, *J* = 5.0, 2.4 Hz, 1H), 4.14 – 4.06 (m, 2H), 3.94 (ddd, *J* = 7.6, 5.7, 1.8 Hz, 1H), 3.68 (dd, *J* = 10.1, 5.7 Hz, 1H), 3.57 (dd, *J* = 10.0, 6.5 Hz, 1H), 2.99 (dd, *J* = 13.9, 7.6 Hz, 1H), 2.90 (dd, *J* = 13.9, 5.8 Hz, 1H), 1.52 (s, 3H), 1.44 (s, 3H), 1.33 (s, 3H), 1.32 (s, 3H).

^{13}C NMR (126 MHz, $CDCl_3$) δ 138.15, 137.26, 128.64, 128.51, 128.16, 127.98, 127.88, 127.81, 109.41, 108.80, 96.71 (d, J_{C-F} = 186.6 Hz), 96.63, 86.74 (d, J_{C-F} = 19.3 Hz), 83.68 (d, J_{C-F} = 26.4 Hz), 82.69 (d, J_{C-F} = 1.7 Hz), 73.58, 72.24, 71.85, 70.99, 70.68, 70.13 (d, J_{C-F} = 1.7 Hz), 68.96, 31.76, 26.22, 26.12, 25.12, 24.51.

HRMS (ESI): calc. for $C_{31}H_{39}FO_8SNa$ $[M+Na]^+$: 613.2242; found: 613.2228.

^{19}F NMR (471 MHz, $CDCl_3$) δ -191.75.

(3a*R*,5*S*,5a*R*,8a*S*,8b*R*)-5-((((2*R*,3*S*,4*R*,5*R*)-4-(benzyloxy)-5-((benzyloxy)methyl)-3-fluoro-tetrahydrofuran-2-yl)thio)methyl)-2,2,7,7-tetramethyltetrahydro-5*H*-bis([1,3]dioxolo)[4,5-*b*:4',5'-*d*]pyran

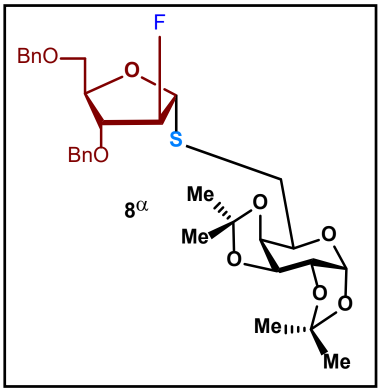

Prepared according to General Procedure **A** with PMP base (2 equiv.) at 50 °C for 15 h. The desired product **8α** was obtained as a colorless oil (24 mg, 41%, α:β 1:1).

Note: To eliminate impurities, we performed multiple purifications and weren't successful. This experiment was solely designed to establish mechanistic insight into the reactivity of substituted furanosyl bromide with nucleophilic acceptor. Thus, we reported the data even though it was impure.

^{1}H NMR (500 MHz, $CDCl_3$) δ 7.35 – 7.27 (m, 10H), 5.53 (d, *J* = 5.1 Hz, 1H), 5.52 (d, *J* = 20.0 Hz, 1H), 4.97 (dt, *J* = 52.9, 1.8 Hz, 1H), 4.67 (d, *J* = 11.9 Hz, 1H), 4.61 (dd, *J* = 7.9, 2.4 Hz, 1H), 4.52 (dd, *J* = 19.6, 12.4 Hz, 3H), 4.35 – 4.29 (m, 3H), 4.14 – 4.06 (m, 1H), 3.99 (td, *J* = 7.3, 1.9 Hz, 1H), 3.67 – 3.58 (m, 2H), 2.95 (dd, *J* = 13.7, 7.6 Hz, 1H), 2.86 (dd, *J* = 13.6, 7.1 Hz, 1H), 1.51 (s, 3H), 1.44 (s, 3H), 1.34 (s, 3H), 1.32 (s, 3H).

^{13}C NMR (126 MHz, $CDCl_3$) δ 138.08, 137.31, 129.92, 128.62, 128.53, 128.13, 128.04, 127.84, 109.47, 108.79, 100.63 (d, $J_{C\text{-}F}$ = 190.7 Hz), 96.85, 88.26 (d, $J_{C\text{-}F}$ = 29.2 Hz), 83.14 (d, $J_{C\text{-}F}$ = 25.3 Hz), 81.04 (d, $J_{C\text{-}F}$ = 4.0 Hz), 73.61, 72.66, 71.53, 71.11, 70.69, 68.81, 66.76, 31.36, 26.16, 26.13, 25.08, 24.63.

HRMS (ESI): calc. for $C_{31}H_{39}FO_8SNa$ $[M+Na]^+$: 613.2242; found: 613.2235.

^{19}F NMR (471 MHz, $CDCl_3$) δ -175.96.

((2*R*,3*S*,4*S*,5*R*)-3,4-bis(benzyloxy)-5-(((3a*R*,5*R*,5a*S*,8a*S*,8b*R*)-2,2,7,7-tetramethyltetrahydro-5*H*-bis([1,3]dioxolo)[4,5-*b*:4',5'-*d*]pyran-5-yl)methoxy)tetrahydrofuran-2-yl)methyl benzoate

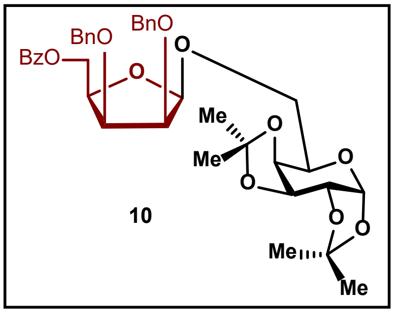

Prepared according to General Procedure **A** at 50 °C for 15 h. The desired product **10** was obtained as a colorless oil (57 mg, 84%, α:β 1:5).

^{1}H NMR (500 MHz, $CDCl_3$) δ 8.05 – 8.01 (m, 2H), 7.57 – 7.52 (m, 1H), 7.42 (dd, *J* = 8.6, 7.2 Hz, 4H), 7.37 – 7.32 (m, 4H), 7.32 – 7.26 (m, 4H), 5.54 (d, *J* = 5.0 Hz, 1H), 5.23 (d, *J* = 4.3 Hz, 1H), 4.91 (d, *J* = 12.0 Hz, 1H), 4.86 (d, *J* = 12.2 Hz, 1H), 4.69 (dd, *J* = 11.6, 4.7 Hz, 1H), 4.63 (t, *J* = 12.2 Hz, 2H), 4.57 (dd, *J* = 11.6, 7.7 Hz, 1H), 4.49 (dd, *J* = 8.0, 2.3 Hz, 1H), 4.38 (ddd, *J* = 7.7, 5.9, 4.6 Hz, 1H), 4.27 (dd, *J* = 5.0, 2.3 Hz, 1H), 4.24 (dd, *J* = 8.0, 1.8 Hz, 1H), 4.17 (t, *J* = 5.8 Hz, 1H), 4.10 (ddd, *J* = 7.0, 5.4, 1.8 Hz, 1H), 4.01 (dd, *J* = 10.6, 5.5 Hz, 1H), 3.87 (dd, *J* = 5.7, 4.3 Hz, 1H), 3.71 (dd, *J* = 10.6, 6.8 Hz, 1H), 1.46 (s, 3H), 1.40 (s, 3H), 1.31 (s, 3H), 1.25 (s, 3H).

^{13}C NMR (126 MHz, $CDCl_3$) δ 166.58, 138.54, 138.27, 132.98, 130.47, 129.82, 128.50, 128.43, 128.36, 128.09, 127.97, 127.86, 127.68, 109.25, 108.61, 100.52, 96.41, 79.21, 77.95, 75.84, 73.83, 72.38, 71.00, 70.84, 70.67, 67.96, 66.08, 65.58, 26.19, 26.04, 25.10, 24.20.
HRMS (ESI): calc. for $C_{38}H_{44}O_{11}Na$ $[M+Na]^+$: 699.2776; found: 699.2763.

((2*R*,3*S*,4*S*,5*S*)-3,4-bis(benzyloxy)-5-((((3a*R*,5*S*,5a*R*,8a*S*,8b*R*)-2,2,7,7-tetramethyltetrahydro-5*H*-bis([1,3]dioxolo)[4,5-*b*:4',5'-*d*]pyran-5-yl)methyl)thio)tetrahydrofuran-2-yl)methyl benzoate

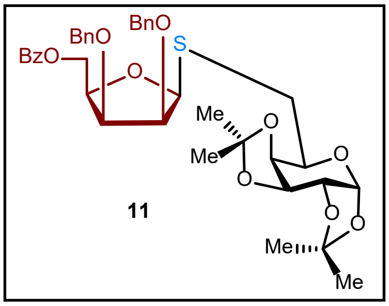

Prepared according to General Procedure **A** at 50 °C for 15 h, the desired product **11** was obtained as a colorless oil (35 mg, 50%, α:β 1:20).
^{1}H NMR (500 MHz, $CDCl_3$) δ 8.01 (d, *J* = 6.9 Hz, 2H), 7.54 (t, *J* = 7.4 Hz, 1H), 7.42 (q, *J* = 7.5 Hz, 4H), 7.37 – 7.32 (m, 4H), 7.29 (t, *J* = 7.2 Hz, 4H), 5.66 (d, *J* = 5.5 Hz, 1H), 5.53 (d, *J* = 4.9 Hz, 1H), 4.89 (t, *J* = 12.3 Hz, 2H), 4.74 (dd, *J* = 11.6, 4.8 Hz, 1H), 4.66 (dd, *J* = 11.9, 3.8 Hz, 2H), 4.61 (dd, *J* = 11.7, 7.3 Hz, 1H), 4.56 (dd, *J* = 7.9, 2.4 Hz, 1H), 4.36 (dt, *J* = 7.4, 5.0 Hz, 1H), 4.31 (dd, *J* = 8.0, 1.9 Hz, 1H), 4.27 (dd, *J* = 4.9, 2.3 Hz, 1H), 4.19 (dq, *J* = 10.0, 4.6 Hz, 2H), 3.97 (ddd, *J* = 7.5, 5.3, 1.9 Hz, 1H), 3.04 (dd, *J* = 14.3, 7.8 Hz, 1H), 2.83 (dd, *J* = 14.3, 5.3 Hz, 1H), 1.46 (s, 3H), 1.44 (s, 3H), 1.31 (s, 3H), 1.30 (s, 3H).
^{13}C NMR (126 MHz, $CDCl_3$) δ 166.53, 138.26, 138.17, 132.97, 130.42, 129.85, 128.46, 128.39, 127.89, 127.83, 127.78, 127.72, 109.30, 108.73, 96.62, 87.87, 79.99, 77.91, 77.49, 73.49, 73.07, 72.11, 70.98, 70.74, 70.23, 65.09, 31.41, 26.24, 26.15, 25.15, 24.46.
HRMS (ESI): calc. for $C_{38}H_{44}O_{10}SNa$ $[M+Na]^+$: 715.2547; found: 715.2533.

(3a*R*,5*R*,5a*S*,8a*S*,8b*R*)-5-((((2*S*,3*R*,4*R*,5*R*)-3,4-bis(benzyloxy)-5-((benzyloxy)methyl)tetra-hydrofuran-2-yl)oxy)methyl)-2,2,7,7-tetramethyltetrahydro-5*H*-bis([1,3]dioxolo)[4,5-*b*:4',5'-*d*]pyran

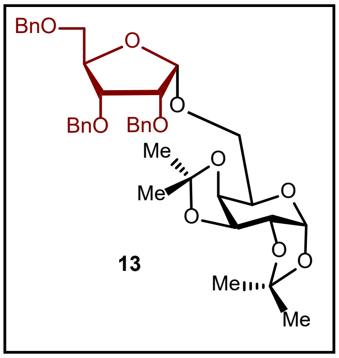

13

Prepared according to General Procedure **A** at 50 °C for 15 h. The desired product **13** was obtained as a colorless oil (56 mg, 84%, α:β 5:1). The ^{1}H NMR of compound **13** matches the literature report.[8]

^{1}**H NMR** (500 MHz, $CDCl_3$) δ 7.41 – 7.26 (m, 13H), 7.23 (d, J = 7.3 Hz, 2H), 5.53 (d, J = 5.0 Hz, 1H), 5.15 (d, J = 3.7 Hz, 1H), 4.76 – 4.73 (m, 2H), 4.64 (d, J = 2.2 Hz, 1H), 4.60 – 4.55 (m, 2H), 4.50 (d, J = 12.1 Hz, 1H), 4.45 – 4.42 (m, 1H), 4.41 (d, J = 7.9 Hz, 1H), 4.31 (dd, J = 4.7, 2.2 Hz, 1H), 4.26 (d, J = 3.9 Hz, 1H), 4.15 (dd, J = 8.5, 6.0 Hz, 1H), 3.93 – 3.86 (m, 2H), 3.84 – 3.76 (m, 2H), 3.50 – 3.44 (m, 1H), 3.40 (dd, J = 10.5, 4.0 Hz, 1H), 1.54 (s, 3H), 1.46 (s, 3H), 1.34 (s, 3H), 1.31 (s, 3H).

(3a*R*,5*S*,5a*R*,8a*S*,8b*R*)-5-((((2*R*,3*R*,4*R*,5*R*)-3,4-bis(benzyloxy)-5-((benzyloxy)methyl)tetra-hydrofuran-2-yl)thio)methyl)-2,2,7,7-tetramethyltetrahydro-5*H*-bis([1,3]dioxolo)[4,5-*b*:4',5'-*d*]pyran

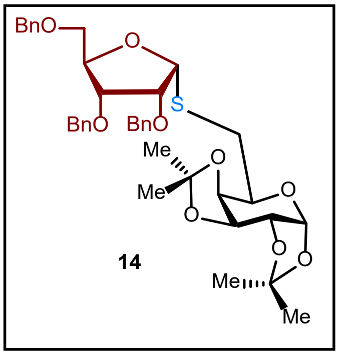

14

Prepared according to General Procedure **A** at 50 °C for 15 h. The desired product **14** was obtained as a colorless oil (48 mg, 71%, α:β 20:1).

^{1}**H NMR** (500 MHz, $CDCl_3$) δ 7.37 (d, J = 6.9 Hz, 2H), 7.31 – 7.20 (m, 13H), 5.53 (d, J = 5.3 Hz, 1H), 5.50 (d, J = 5.1 Hz, 1H), 4.76 (d, J = 11.8 Hz, 1H), 4.63 (dd, J = 22.2, 12.0 Hz, 2H), 4.59 –

4.53 (m, 1H), 4.50 – 4.44 (m, 3H), 4.39 (d, *J* = 12.1 Hz, 1H), 4.26 (td, *J* = 6.6, 5.8, 2.8 Hz, 2H), 4.08 (t, *J* = 5.5 Hz, 1H), 4.02 (ddd, *J* = 9.8, 5.8, 2.1 Hz, 1H), 3.93 (t, *J* = 5.8 Hz, 1H), 3.54 (dd, *J* = 10.8, 3.3 Hz, 1H), 3.47 (dd, *J* = 10.9, 3.5 Hz, 1H), 2.97 (dd, *J* = 13.5, 9.5 Hz, 1H), 2.80 (dd, *J* = 13.5, 5.6 Hz, 1H), 1.47 (s, 3H), 1.42 (s, 3H), 1.31 (s, 3H), 1.29 (s, 3H).

^{13}C NMR (126 MHz, $CDCl_3$) δ 138.28, 138.15, 137.94, 128.48, 128.45, 128.44, 128.43, 128.11, 127.99, 127.86, 127.76, 127.75, 109.16, 108.66, 96.88, 89.91, 80.48, 78.43, 73.55, 73.33, 72.71, 71.00, 70.99, 70.82, 69.28, 67.44, 31.78, 26.16, 26.14, 25.15, 24.63.

HRMS (ESI): calc. for $C_{38}H_{46}O_9SNa$ $[M+Na]^+$: 701.2755; found: 701.2747.

(3a*R*,5*R*,5a*S*,8a*S*,8b*R*)-5-((((2*S*,3*R*,4*S*,5*R*)-3,4-bis(benzyloxy)-5-((benzyloxy)methyl)tetra-hydrofuran-2-yl)oxy)methyl)-2,2,7,7-tetramethyltetrahydro-5*H*-bis([1,3]dioxolo)[4,5-*b*:4',5'-*d*]pyran

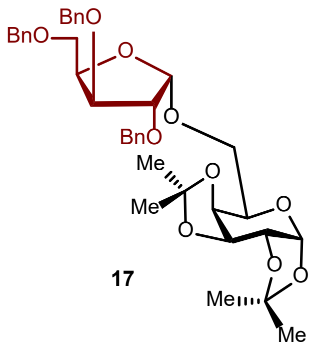

17

Prepared according to General Procedure **A** at 50 °C for 15 h. The desired product **17** was obtained as a colorless oil (58 mg, 88%, α:β 14:1). The ^{1}H NMR of compound **17** matches the literature report.[8]

^{1}H NMR (600 MHz, $CDCl_3$) δ 7.37 – 7.23 (m, 15H), 5.51 (d, *J* = 5.0 Hz, 1H), 5.10 (d, *J* = 4.3 Hz, 1H), 4.70 (d, *J* = 11.8 Hz, 1H), 4.64 (d, *J* = 11.9 Hz, 1H), 4.60 – 4.48 (m, 5H), 4.44 – 4.40 (m, 1H), 4.32 – 4.26 (m, 3H), 4.06 - 4.01 (m, 2H), 3.88 (dd, *J* = 9.9, 6.0 Hz, 1H), 3.74 – 3.66 (m, 2H), 3.59 (dd, *J* = 10.7, 6.6 Hz, 1H), 1.51 (s, 3H), 1.44 (s, 3H), 1.31 (s, 3H), 1.29 (s, 3H).

(3a*R*,5*R*,5a*S*,8a*S*,8b*R*)-5-((((2*S*,3*R*,4*S*,5*R*)-4-(benzyloxy)-5-((benzyloxy)methyl)-3-fluoro-tetrahydrofuran-2-yl)oxy)methyl)-2,2,7,7-tetramethyltetrahydro-5*H*-bis([1,3]dioxolo)[4,5-*b*:4',5'-*d*]pyran

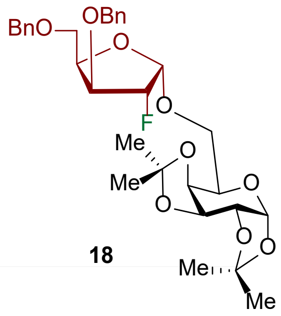

18

Prepared according to General Procedure **A** at 50 °C for 15 h. The desired product **18** was obtained as a colorless oil (46 mg, 80%, α:β 9:1). The ^{1}H NMR of compound **18** matches the literature report.[1]

^{1}H NMR (600 MHz, $CDCl_3$) δ 7.35 – 7.27 (m, 10H), 5.51 (d, *J* = 5.0 Hz, 1H), 5.18 (dd, *J* = 4.4, 1.2 Hz, 1H), 5.01 (dt, *J* = 53.0, 4.8 Hz, 1H), 4.71 (d, *J* = 11.8 Hz, 1H), 4.62 – 4.57 (m, 2H), 4.56 (d, *J* = 12.1 Hz, 1H), 4.53 (d, *J* = 12.0 Hz, 1H), 4.50 -4.45 (m, 1H), 4.44 – 4.35 (m, 1H), 4.30 (dd, *J* = 5.1, 2.4 Hz, 1H), 4.28 (dd, *J* = 8.0, 1.9 Hz, 1H), 4.02 (ddd, *J* = 7.7, 5.9, 1.9 Hz, 1H), 3.88 (dd, *J* = 10.4, 6.0 Hz, 1H), 3.78 (dd, *J* = 10.4, 7.6 Hz, 1H), 3.71 (dd, *J* = 10.6, 4.2 Hz, 1H), 3.61 (dd, *J* = 10.6, 5.8 Hz, 1H), 1.52 (s, 3H), 1.44 (s, 3H), 1.34 (s, 3H), 1.32 (s, 3H).

^{19}F NMR (564 MHz, $CDCl_3$) δ -204.19 (dd, *J* = 53.0, 16.4 Hz).

(3a*R*,5*S*,5a*R*,8a*S*,8b*R*)-5-((((2*R*,3*R*,4*S*,5*R*)-3,4-bis(benzyloxy)-5-((benzyloxy)methyl)tetra-hydrofuran-2-yl)thio)methyl)-2,2,7,7-tetramethyltetrahydro-5*H*-bis([1,3]dioxolo)[4,5-*b*:4',5'-*d*]pyran

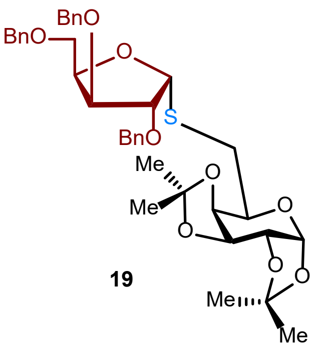

19

Prepared according to General Procedure **A** at 50 °C for 15 h. The desired product **19** was obtained as a colorless oil (46 mg, 68%, α:β 17:1).

^{1}H NMR (500 MHz, $CDCl_3$) δ 7.36 – 7.27 (m, 13H), 7.24 – 7.20 (m, 2H), 5.57 (d, *J* = 5.0 Hz, 1H), 5.53 (d, *J* = 5.0 Hz, 1H), 4.62 (d, *J* = 11.8 Hz, 1H), 4.59 (dd, *J* = 7.9, 2.4 Hz, 1H), 4.56 (d, *J* = 11.9 Hz, 1H), 4.52 – 4.43 (m, 6H), 4.29 (dd, *J* = 5.1, 2.4 Hz, 1H), 4.12 (dd, *J* = 5.0, 2.5 Hz, 1H), 4.06 (dt, *J* = 4.7, 2.3 Hz, 2H), 3.73 (dd, *J* = 9.9, 6.5 Hz, 1H), 3.62 (dd, *J* = 9.9, 5.6 Hz, 1H), 2.97 (dd, *J* = 13.5, 9.0 Hz, 1H), 2.83 (dd, *J* = 13.5, 6.0 Hz, 1H), 1.48 (s, 3H), 1.44 (s, 3H), 1.34 (s, 3H), 1.31 (s, 3H).

^{13}C NMR (126 MHz, $CDCl_3$) δ 138.36, 138.01, 137.57, 128.56, 128.55, 128.48, 128.14, 128.06, 127.92, 127.87, 127.75, 127.72, 109.21, 108.73, 96.90, 88.94, 83.85, 81.82, 78.04, 73.56, 72.94, 72.37, 71.14, 71.05, 70.81, 67.97, 67.18, 31.42, 26.19, 26.15, 25.17, 24.63.

HRMS (ESI): calc. for $C_{38}H_{47}O_9SNa$ $[M+H]^+$: 679.2935; found: 679.2913.

(3a*R*,5*S*,5a*R*,8a*S*,8b*R*)-5-((((2*R*,3*R*,4*S*,5*R*)-4-(Benzyloxy)-5-((benzyloxy)methyl)-3-fluoro-tetrahydrofuran-2-yl)thio)methyl)-2,2,7,7-tetramethyltetrahydro-5*H*-bis([1,3]dioxolo)[4,5-*b*:4',5'-*d*]pyran

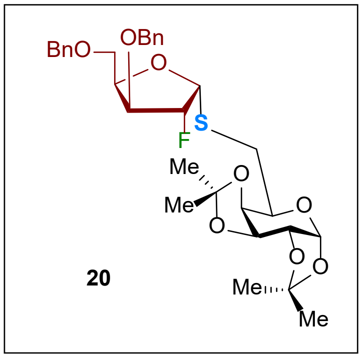

Prepared according to General Procedure **A** at 50 °C for 15 h. The desired product **20** was obtained as a colorless oil (38 mg, 64%, α:β 20:1).

^{1}H NMR (500 MHz, $CDCl_3$) δ 7.36 – 7.26 (m, 10H), 5.52 (d, *J* = 5.0 Hz, 1H), 5.51 (dd, *J* = 26.3, 4.0 Hz, 1H), 5.08 (ddd, *J* = 51.5, 3.8, 1.7 Hz, 1H), 4.66 – 4.54 (m, 4H), 4.51 (d, *J* = 11.9 Hz, 1H), 4.48 – 4.41 (m, 2H), 4.30 (dd, *J* = 5.1, 2.4 Hz, 1H), 4.18 (ddd, *J* = 11.1, 4.2, 1.8 Hz, 1H), 4.02 (ddd, *J* = 8.4, 6.1, 1.8 Hz, 1H), 3.73 (dd, *J* = 9.8, 6.6 Hz, 1H), 3.64 (dd, *J* = 9.8, 5.4 Hz, 1H), 2.98 (dd, *J* = 13.5, 8.7 Hz, 1H), 2.88 (dd, *J* = 13.4, 6.2 Hz, 1H), 1.48 (s, 3H), 1.44 (s, 3H), 1.33 (s, 3H), 1.31 (s, 3H).

^{13}C NMR (126 MHz, $CDCl_3$) δ 138.17, 137.41, 128.66, 128.52, 128.18, 127.86, 127.82, 127.78, 109.32, 108.77, 96.88, 95.60 (d, J_{C-F} = 188.6 Hz), 87.34 (d, J_{C-F} = 17.5 Hz), 81.16 (d, J_{C-F} = 25.0

Hz), 78.82, 73.63, 72.88, 71.08, 71.04, 70.73, 67.48, 67.25, 31.49 (d, $J_{C\text{-}F}$ = 2.1 Hz), 26.13, 26.12, 25.12, 24.64.

HRMS (ESI): calc. for $C_{31}H_{39}O_8FSNa$ $[M+Na]^+$: 613.2242; found: 613.2232.

^{19}**F NMR** (471 MHz, $CDCl_3$) δ -191.17.

(2*R*,3*R*,4*S*,5*S*)-3,4-bis(benzyloxy)-2-((benzyloxy)methyl)-5-((((2*S*,3*S*,4*S*,5*S*)-3,4-bis(benzyloxy)-5-methoxytetrahydrofuran-2-yl)methyl)thio)tetrahydrofuran

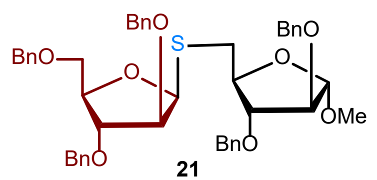

21

Prepared according to General Procedure **A** at 50 °C for 15 h. The desired product **21** was obtained as a colorless oil (42 mg, 55%, α:β 1:20).

^{1}**H NMR** (500 MHz, $CDCl_3$) δ 7.34 – 7.25 (m, 25H), 5.55 (d, *J* = 5.1 Hz, 1H), 4.92 (s, 1H), 4.62 (d, *J* = 11.8 Hz, 1H), 4.58 – 4.49 (m, 7H), 4.46 (dd, *J* = 11.8, 2.3 Hz, 2H), 4.26 – 4.20 (m, 1H), 4.20 – 4.16 (m, 1H), 4.16 – 4.12 (m, 1H), 4.07 (t, *J* = 4.3 Hz, 1H), 3.98 – 3.95 (m, 1H), 3.81 (dd, *J* = 6.4, 2.9 Hz, 1H), 3.71 (dd, *J* = 9.9, 6.3 Hz, 1H), 3.65 (dd, *J* = 10.0, 6.4 Hz, 1H), 3.35 (s, 3H), 2.96 (dd, *J* = 13.7, 7.5 Hz, 1H), 2.86 (dd, *J* = 13.7, 5.0 Hz, 1H).

^{13}**C NMR** (126 MHz, $CDCl_3$) δ 138.32, 138.02, 137.94, 137.65, 137.64, 128.59, 128.53, 128.52, 128.48, 128.05, 128.03, 128.02, 127.96, 127.90, 127.88, 127.86, 127.85, 127.72, 107.25, 88.35, 87.07, 86.63, 84.42, 83.96, 82.10, 81.90, 77.36, 73.46, 72.52, 72.31, 72.16, 72.07, 71.55, 54.94, 32.86.

HRMS (ESI): calc. for $C_{46}H_{50}O_8SNa$ $[M+Na]^+$: 785.3119; found: 785.3107.

(2*R*,3*R*,4*S*,5*R*)-3,4-bis(benzyloxy)-2-((benzyloxy)methyl)-5-(((2*R*,3*R*,4*S*,5*S*)-3,4-bis(benzyloxy)-5-methoxytetrahydrofuran-2-yl)methoxy)tetrahydrofuran

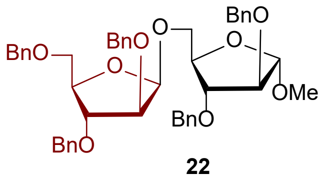

22

Prepared according to General Procedure **A** at 50 °C for 15 h. The desired product **22** was obtained as a colorless oil (66 mg, 88%, α:β 1:5). The ^{1}H NMR of compound **22** matches the literature report.[9]

^{1}H NMR (400 MHz, $CDCl_3$) δ 7.40 – 7.20 (m, 25H), 5.07 (d, *J* = 4.1 Hz, 1H), 4.92 (s, 1H), 4.66 - 4.44 (m, 10H), 4.25 (td, *J* = 6.7, 3.5 Hz, 1H), 4.14 – 4.05 (m, 3H), 3.99 (dd, *J* = 2.9, 1.2 Hz, 1H), 3.84 – 3.77 (m, 2H), 3.61 – 3.52 (m, 3H), 3.35 (s, 3H).

(2*S*,3*S*,4*R*,5*R*)-3,4-bis(benzyloxy)-2-(((2*S*,3*R*,4*S*,5*S*)-4-(benzyloxy)-2-((benzyloxy)methyl)-5-methyltetrahydrofuran-3-yl)thio)-5-((benzyloxy)methyl)tetrahydrofuran

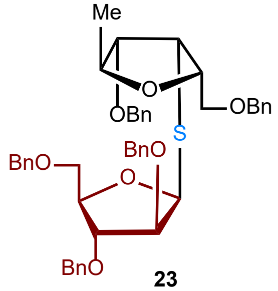

23

Prepared according to General Procedure **A** at 50 °C for 15 h. The desired product **23** was obtained as a colorless oil (43 mg, 57%, α:β 1:20).

^{1}H NMR (600 MHz, $CDCl_3$) δ 7.35 – 7.24 (m, 25H), 5.45 (d, *J* = 4.9 Hz, 1H), 4.73 (d, *J* = 11.6 Hz, 1H), 4.59 – 4.49 (m, 7H), 4.45 (dd, *J* = 11.9, 9.0 Hz, 2H), 4.19 (td, *J* = 6.1, 4.5 Hz, 1H), 4.11 (dt, *J* = 5.0, 2.6 Hz, 3H), 4.05 (t, *J* = 3.8 Hz, 1H), 3.81 (t, *J* = 4.9 Hz, 1H), 3.69 – 3.59 (m, 4H), 3.43 (dd, *J* = 6.4, 4.9 Hz, 1H), 1.27 (d, *J* = 6.5 Hz, 3H).

^{13}C NMR (126 MHz, $CDCl_3$) δ 138.33, 138.31, 138.28, 137.92, 137.46, 128.58, 128.55, 128.47, 128.46, 128.10, 128.08, 127.92, 127.85, 127.80, 127.75, 127.74, 127.70, 90.40, 87.15, 84.45, 83.71, 83.56, 82.39, 79.27, 73.59, 73.40, 72.57, 72.48, 71.99, 71.19, 71.14, 49.60, 19.30.

HRMS (ESI): calc. for $C_{46}H_{50}O_7SNa$ $[M+Na]^+$: 769.3169; found: 769.3158.

(2*S*,3*S*,4*R*,5*R*)-3,4-bis(benzyloxy)-2-(((2*S*,3*S*,4*S*,5*R*)-4-(benzyloxy)-5-((benzyloxy)methyl)-2-methoxytetrahydrofuran-3-yl)thio)-5-((benzyloxy)methyl)tetrahydrofuran

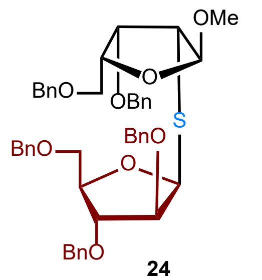

24

Prepared according to General Procedure **A** at 50 °C for 15 h. The desired product **24** was obtained as a colorless oil (42 mg, 55%, α:β 1:15).

^{1}H NMR (500 MHz, $CDCl_3$) δ 7.33 – 7.30 (m, 8H), 7.27 (tdd, *J* = 11.8, 6.4, 4.1 Hz, 17H), 5.52 (d, *J* = 4.9 Hz, 1H), 5.11 (d, *J* = 4.5 Hz, 1H), 4.69 (d, *J* = 11.2 Hz, 1H), 4.61 (d, *J* = 9.9 Hz, 1H), 4.59 – 4.53 (m, 2H), 4.53 – 4.47 (m, 5H), 4.47 – 4.43 (m, 2H), 4.31 (td, *J* = 6.2, 3.4 Hz, 1H), 4.15 (td, *J* = 5.4, 3.8 Hz, 1H), 4.12 – 4.08 (m, 1H), 4.05 (t, *J* = 3.9 Hz, 1H), 3.68 (d, *J* = 6.3 Hz, 2H), 3.66 – 3.60 (m, 2H), 3.56 (t, *J* = 4.9 Hz, 1H), 3.39 (s, 3H).

^{13}C NMR (126 MHz, $CDCl_3$) δ 138.25, 138.19, 138.07, 137.94, 137.57, 128.53, 128.52, 128.50, 128.48, 128.35, 128.19, 128.07, 127.99, 127.98, 127.96, 127.89, 127.88, 127.83, 127.81, 127.79, 127.75, 111.14, 86.05, 84.39, 83.87, 82.26, 80.79, 79.86, 74.35, 73.64, 73.38, 72.62, 72.01, 71.23, 68.70, 56.13, 52.31.

HRMS (ESI): calc. for $C_{46}H_{50}O_8SNa$ $[M+Na]^+$: 785.3119; found: 785.3106.

(2*R*,3*R*,4*S*,5*S*)-3,4-bis(benzyloxy)-2-((benzyloxy)methyl)-5-(((2*E*,6*E*)-3,7,11-trimethyldodeca-2,6,10-trien-1-yl)thio)tetrahydrofuran

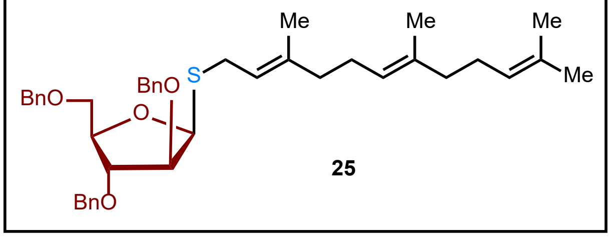

25

Prepared according to General Procedure **A** at 50 °C for 15 h. The desired product **25** was obtained as a colorless oil (35 mg, 55%, α:β 1:20).

^{1}H NMR (500 MHz, $CDCl_3$) δ 7.36 – 7.23 (m, 15H), 5.35 (d, *J* = 5.0 Hz, 1H), 5.27 (t, *J* = 7.8 Hz, 1H), 5.15 – 5.05 (m, 2H), 4.64 – 4.46 (m, 6H), 4.18 (dd, *J* = 5.0, 3.9 Hz, 1H), 4.13 (td, *J* = 6.3, 4.2 Hz, 1H), 4.06 (td, *J* = 4.1, 1.9 Hz, 1H), 3.78 – 3.71 (m, 1H), 3.67 (ddd, *J* = 9.5, 6.5, 2.7 Hz, 1H),

3.41 (ddd, J = 14.0, 9.0, 5.1 Hz, 1H), 3.19 (dd, J = 13.1, 6.7 Hz, 1H), 2.08 (ddt, J = 21.2, 9.6, 6.0 Hz, 6H), 1.98 (dd, J = 9.7, 5.8 Hz, 2H), 1.69 – 1.64 (m, 6H), 1.60 (d, J = 1.6 Hz, 6H).

^{13}C NMR (126 MHz, $CDCl_3$) δ 139.67, 138.39, 138.06, 137.74, 135.42, 131.45, 128.53, 128.52, 128.48, 128.00, 127.93, 127.88, 127.87, 127.86, 127.85, 127.73, 124.51, 124.04, 120.03, 85.98, 84.53, 84.20, 82.11, 73.48, 72.45, 72.03, 71.65, 39.88, 39.83, 28.05, 26.90, 26.67, 25.84, 17.85, 16.18, 16.17.

HRMS (ESI): calc. for $C_{41}H_{52}O_4SNa$ $[M+Na]^+$: 663.3479; found: 663.3470.

5-(((2*S*,3*S*,4*R*,5*R*)-3,4-bis(benzyloxy)-5-((benzyloxy)methyl)tetrahydrofuran-2-yl)thio)-1-phenyl-1*H*-tetrazole

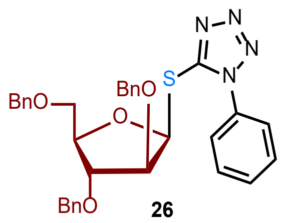

26

Prepared according to General Procedure **A** at 50 °C for 15 h. The desired product **26** was obtained as a colorless oil (45 mg, 77%, α:β 1:20).

^{1}H NMR (500 MHz, $CDCl_3$) δ 7.77 – 7.71 (m, 2H), 7.50 – 7.40 (m, 3H), 7.28 – 7.19 (m, 13H), 7.12 (dd, J = 6.8, 2.8 Hz, 2H), 6.78 (d, J = 6.2 Hz, 1H), 4.68 (d, J = 11.6 Hz, 1H), 4.60 – 4.42 (m, 7H), 4.24 (td, J = 7.3, 3.8 Hz, 1H), 3.77 (dd, J = 10.7, 7.1 Hz, 1H), 3.69 (dd, J = 10.7, 3.8 Hz, 1H).

^{13}C NMR (126 MHz, $CDCl_3$) δ 163.91, 138.00, 137.84, 136.80, 134.58, 129.84, 129.37, 128.62, 128.58, 128.48, 128.22, 128.04, 127.91, 127.89, 127.85, 127.78, 124.28, 85.06, 83.30, 81.45, 81.28, 73.49, 73.45, 72.98, 71.05.

HRMS (ESI): calc. for $C_{33}H_{32}N_4O_4SNa$ $[M+Na]^+$: 603.2042; found: 603.2018.

Methyl *S*-((2*S*,3*S*,4*R*,5*R*)-3,4-bis(benzyloxy)-5-((benzyloxy)methyl)tetrahydrofuran-2-yl)-*N*-(*tert*-butoxycarbonyl)-L-cysteinate

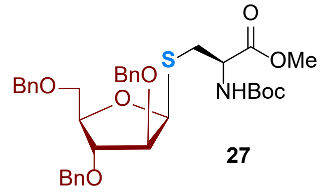

27

Prepared according to General Procedure **A** at 50 °C for 15 h. The desired product **27** was obtained as a colorless oil (50 mg, 78%, α:β 1:25).

^{1}H NMR (500 MHz, $CDCl_3$) δ 7.36 – 7.28 (m, 13H), 7.27 – 7.24 (m, 2H), 5.59 (d, *J* = 7.8 Hz, 1H), 5.42 (d, *J* = 5.0 Hz, 1H), 4.63 – 4.48 (m, 7H), 4.19 (dd, *J* = 5.0, 3.8 Hz, 1H), 4.16 – 4.10 (m, 1H), 4.08 (t, *J* = 4.1 Hz, 1H), 3.73 (s, 3H), 3.70 (dd, *J* = 10.0, 5.9 Hz, 1H), 3.63 (dd, *J* = 9.9, 6.3 Hz, 1H), 3.22 (dd, *J* = 14.1, 4.7 Hz, 1H), 2.98 (dd, *J* = 14.1, 6.7 Hz, 1H), 1.43 (s, 9H).

^{13}C NMR (126 MHz, $CDCl_3$) δ 171.67, 155.50, 138.22, 137.87, 137.41, 128.56, 128.52, 128.48, 128.09, 128.07, 127.90, 127.87, 127.85, 127.77, 87.32, 84.25, 83.42, 82.24, 80.03, 73.43, 73.42, 72.54, 72.03, 70.83, 53.31, 52.59, 33.20, 28.44.

HRMS (ESI): calc. for $C_{35}H_{43}O_8NSNa$ $[M+Na]^+$: 660.2602; found: 660.2592.

Methyl *O*-((2*R*,3*S*,4*R*,5*R*)-3,4-bis(benzyloxy)-5-((benzyloxy)methyl)tetrahydrofuran-2-yl)-*N*-(*tert*-butoxycarbonyl)-L-serinate

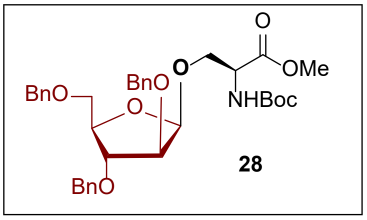

Prepared according to General Procedure **A** at 50 °C for 15 h. The desired product **28** was obtained as a colorless oil (53 mg, 84%, α:β 1:1).

^{1}H NMR (500 MHz, $CDCl_3$) δ 7.38 – 7.21 (m, 25H), 6.32 (d, *J* = 4.9 Hz, 1.7H), 5.40 (d, *J* = 8.4 Hz, 0.6H), 5.33 (d, *J* = 8.5 Hz, 1H), 4.71 – 4.37 (m, 16H), 4.23 (dd, *J* = 7.0, 4.0 Hz, 1H), 4.19 (t, *J* = 5.7 Hz, 1H), 4.17 – 4.10 (m, 2H), 4.06 (d, *J* = 2.1 Hz, 0.7H), 3.99 – 3.95 (m, 0.7H), 3.81 – 3.76 (m, 0.65H), 3.70 (dd, *J* = 9.5, 3.2 Hz, 1H), 3.64 (dd, *J* = 5.0, 3.2 Hz, 2H), 3.58 (d, *J* = 5.5 Hz, 3H), 3.52 (dd, *J* = 9.6, 3.3 Hz, 1H), 3.24 (s, 3H), 3.21 (s, 2H), 1.46 (s, 17H).

^{13}C NMR (126 MHz, $CDCl_3$) δ 169.82, 169.76, 155.47, 155.40, 138.07, 138.06, 138.05, 137.65, 137.42, 137.36, 128.61, 128.56, 128.52, 128.50, 128.49, 128.48, 128.46, 128.44, 128.43, 128.21, 128.15, 128.12, 128.06, 127.95, 127.90, 127.88, 127.86, 127.83, 127.82, 127.78, 101.95, 95.65, 87.28, 83.88, 83.74, 82.10, 81.55, 80.07, 79.96, 73.59, 73.48, 73.21, 72.58, 72.20, 72.19, 72.15, 71.49, 69.65, 59.26, 59.24, 54.12, 28.46, 28.44.

HRMS (ESI): calc. for $C_{35}H_{42}O_9NNa_2$ $[M+2Na-H]^+$: 666.2649; found: 666.2918.

Methyl *N*-(((9*H*-fluoren-9-yl)methoxy)carbonyl)-*S*-((2*S*,3*S*,4*R*,5*R*)-3,4-bis(benzyloxy)-5-((benzyloxy)methyl)tetrahydrofuran-2-yl)-L-cysteinate

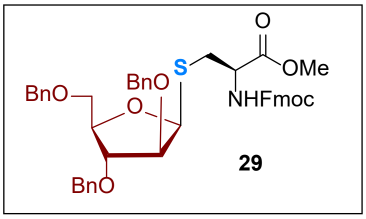

29

Prepared according to General Procedure **A** at 50 °C for 15 h. The desired product **29** was obtained as a colorless oil (55 mg, 72%, α:β 1:20).

^{1}H NMR (500 MHz, $CDCl_3$) δ 7.75 (d, *J* = 7.5 Hz, 2H), 7.62 – 7.55 (m, 2H), 7.38 (t, *J* = 7.5 Hz, 2H), 7.33 – 7.23 (m, 17H), 5.96 (d, *J* = 7.8 Hz, 1H), 5.42 (d, *J* = 5.1 Hz, 1H), 4.65 – 4.45 (m, 7H), 4.37 (dd, *J* = 10.6, 7.3 Hz, 1H), 4.32 (dd, *J* = 10.6, 7.1 Hz, 1H), 4.24 – 4.17 (m, 2H), 4.13 (td, *J* = 6.1, 4.4 Hz, 1H), 4.08 (t, *J* = 4.2 Hz, 1H), 3.74 (s, 3H), 3.67 (dd, *J* = 10.0, 5.9 Hz, 1H), 3.60 (dd, *J* = 10.0, 6.3 Hz, 1H), 3.25 (dd, *J* = 14.2, 4.8 Hz, 1H), 3.05 (dd, *J* = 14.2, 6.6 Hz, 1H).

^{13}C NMR (126 MHz, $CDCl_3$) δ 171.36, 156.16, 144.03, 143.98, 141.44, 141.43, 138.18, 137.87, 137.40, 128.60, 128.57, 128.48, 128.12, 128.10, 127.96, 127.90, 127.89, 127.83, 127.79, 127.23, 125.33, 120.10, 87.43, 84.32, 83.30, 82.28, 73.38, 72.62, 72.11, 70.67, 67.28, 53.80, 52.76, 47.28, 33.22.

HRMS (ESI): calc. for $C_{45}H_{45}O_8NSNa$ $[M+Na]^+$: 782.2758; found: 782.2746.

Methyl *N*-(((9*H*-fluoren-9-yl)methoxy)carbonyl)-*O*-((2*R*,3*S*,4*R*,5*R*)-3,4-bis(benzyloxy)-5-((benzyloxy)methyl)tetrahydrofuran-2-yl)-L-serinate

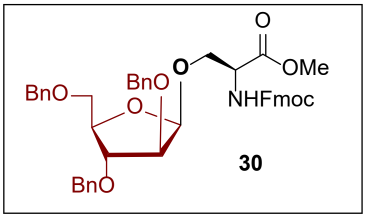

30

Prepared according to General Procedure **A** at 50 °C for 15 h. The desired product **30** was obtained as a colorless oil (56 mg, 75%, α:β 1:4).

^{1}H NMR (600 MHz, $CDCl_3$) δ 7.76 (dd, *J* = 7.6, 2.3 Hz, 0.6H), 7.72 (dd, *J* = 7.6, 5.1 Hz, 2H), 7.63 (d, *J* = 8.7 Hz, 0.5H), 7.59 (dd, *J* = 7.7, 4.2 Hz, 2H), 7.38-7.27 (m, 24H), 6.14 (t, *J* = 9.2 Hz,

1.21H), 5.09 (s, 0.2H), 4.80 (d, J = 4.4 Hz, 1H), 4.68 (d, J = 11.7 Hz, 1.3H), 4.64 – 4.46 (m, 10H), 4.41 (dd, J = 10.8, 7.0 Hz, 1.3H), 4.35 – 4.26 (m, 0.6H), 4.21 (t, J = 6.9 Hz, 1.3H), 4.17 (t, J = 7.0 Hz, 1.3H), 4.11 (dd, J = 10.6, 3.2 Hz, 1.1H), 4.06 (dd, J = 7.4, 4.3 Hz, 1.2H), 4.04 – 4.00 (m, 1.6H), 4.00 – 3.95 (m, 0.6H), 3.78 (s, 0.7H), 3.74 (dd, J = 10.6, 3.8 Hz, 1H), 3.69 (s, 3H), 3.63 (d, J = 5.0 Hz, 0.9H), 3.50 (dd, J = 10.4, 4.5 Hz, 1.2H), 3.43 (dd, J = 10.4, 5.6 Hz, 1.2H).

^{13}C NMR (151 MHz, $CDCl_3$) δ 170.98, 170.69, 156.30, 144.10, 144.00, 143.89, 143.81, 141.37, 141.36, 141.34, 141.31, 138.22, 138.08, 137.94, 137.71, 137.67, 137.43, 128.61, 128.58, 128.49, 128.46, 128.44, 128.43, 128.13, 128.12, 128.04, 128.02, 127.93, 127.86, 127.83, 127.81, 127.78, 127.75, 127.73, 127.16, 127.14, 125.38, 125.24, 125.12, 125.08, 120.05, 120.02, 120.00, 106.10, 101.28, 87.28, 84.18, 83.15, 81.90, 80.04, 73.52, 73.18, 72.69, 72.60, 72.34, 72.01, 70.82, 69.85, 68.45, 67.31, 67.26, 67.02, 54.43, 54.34, 52.64, 52.53, 47.27, 47.19.

HRMS (ESI): calc. for $C_{45}H_{44}O_9NNa_2$ $[M+2Na-H]^+$: 788.2806; found: 788.3071.

Allyl *S*-((*2S*,*3S*,*4R*,*5R*)-3,4-bis(benzyloxy)-5-((benzyloxy)methyl)tetrahydrofuran-2-yl)-*N*-(*tert*-butoxycarbonyl)-L-cysteinate

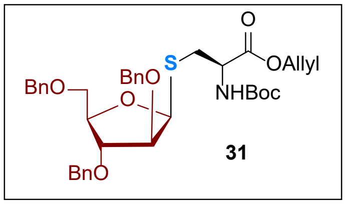

Prepared according to General Procedure **A** at 50 °C for 15 h. The desired product **31** was obtained as a colorless oil (58 mg, 58%, α:β 1:25).

^{1}H NMR (500 MHz, $CDCl_3$) δ 7.38 – 7.21 (m, 15H), 5.89 (ddt, J = 16.3, 10.4, 5.8 Hz, 1H), 5.60 (d, J = 7.6 Hz, 1H), 5.41 (d, J = 5.0 Hz, 1H), 5.32 (dd, J = 17.2, 1.6 Hz, 1H), 5.22 (dd, J = 10.5, 1.3 Hz, 1H), 4.65 – 4.61 (m, 2H), 4.61 – 4.47 (m, 7H), 4.20 – 4.16 (m, 1H), 4.12 (t, J = 4.9 Hz, 1H), 4.07 (t, J = 4.1 Hz, 1H), 3.69 (dd, J = 9.9, 5.8 Hz, 1H), 3.62 (dd, J = 10.0, 6.4 Hz, 1H), 3.23 (dd, J = 14.1, 4.7 Hz, 1H), 3.00 (dd, J = 14.1, 6.7 Hz, 1H), 1.43 (s, 9H).

^{13}C NMR (126 MHz, $CDCl_3$) δ 170.89, 155.51, 138.24, 137.88, 137.42, 131.80, 128.58, 128.53, 128.49, 128.10, 128.08, 127.91, 127.89, 127.86, 127.78, 118.82, 87.40, 84.27, 83.41, 82.27, 80.03, 73.43, 72.57, 72.04, 70.81, 66.26, 53.49, 33.19, 28.46.

HRMS (ESI): calc. for $C_{37}H_{46}O_8NS$ $[M+H]^+$: 664.2939; found: 664.2916.

Allyl *N*-(((9*H*-fluoren-9-yl)methoxy)carbonyl)-*S*-((2*S*,3*S*,4*R*,5*R*)-3,4-bis(benzyloxy)-5-((benzyloxy)methyl)tetrahydrofuran-2-yl)-L-cysteinate

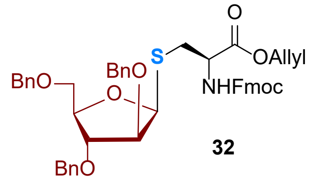

32

Prepared according to General Procedure **A** at 50 °C for 15 h. The desired product **32** was obtained as a colorless oil (55 mg, 70%, α:β 1:15).

^{1}H NMR (500 MHz, $CDCl_3$) δ 7.75 (d, *J* = 7.5 Hz, 2H), 7.59 (t, *J* = 6.6 Hz, 2H), 7.39 (t, *J* = 7.5 Hz, 2H), 7.33 – 7.24 (m, 17H), 5.99 (d, *J* = 7.8 Hz, 1H), 5.90 (ddt, *J* = 16.5, 11.0, 5.8 Hz, 1H), 5.43 (d, *J* = 5.0 Hz, 1H), 5.36 – 5.29 (m, 1H), 5.23 (d, *J* = 10.4 Hz, 1H), 4.67 – 4.62 (m, 2H), 4.62 – 4.54 (m, 2H), 4.54 – 4.46 (m, 4H), 4.38 (dd, *J* = 11.4, 8.1 Hz, 1H), 4.31 (td, *J* = 7.3, 3.5 Hz, 1H), 4.23 – 4.16 (m, 2H), 4.13 (td, *J* = 5.8, 3.5 Hz, 2H), 4.08 (t, *J* = 4.1 Hz, 1H), 3.70 – 3.65 (m, 1H), 3.64 – 3.58 (m, 1H), 3.28 (dd, *J* = 14.2, 4.8 Hz, 1H), 3.07 (dd, *J* = 14.2, 6.6 Hz, 1H).

^{13}C NMR (126 MHz, $CDCl_3$) δ 170.57, 156.15, 143.97, 141.43, 138.17, 137.86, 137.40, 131.72, 128.60, 128.56, 128.48, 128.46, 128.11, 128.09, 127.95, 127.90, 127.89, 127.88, 127.82, 127.77, 127.23, 125.32, 120.09, 119.01, 87.44, 84.31, 83.26, 82.27, 73.37, 72.62, 72.09, 70.62, 67.29, 66.45, 53.91, 47.27, 33.17.

HRMS (ESI): calc. for $C_{47}H_{47}O_8NSNa$ $[M+Na]^+$: 808.2915; found: 808.2904.

Allyl *N*-(((9*H*-fluoren-9-yl)methoxy)carbonyl)-*S*-((2*S*,3*S*,4*R*,5*R*)-3,4-bis(benzyloxy)-5-((benzyloxy)methyl)tetrahydrofuran-2-yl)-L-cysteinyl-L-phenylalaninate

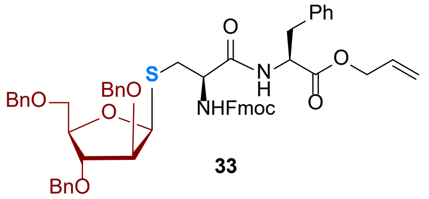

33

Prepared according to General Procedure **A** with DCM as solvent at room temperature for 15 h. The desired product **33** was obtained as a colorless oil (68 mg, 73%, α:β 1:18).

^{1}H NMR (500 MHz, $CDCl_3$) δ 7.77 (dd, *J* = 7.6, 3.2 Hz, 2H), 7.58 (d, *J* = 7.4 Hz, 2H), 7.40 (t, *J* = 7.3 Hz, 2H), 7.35 – 7.17 (m, 20H), 7.12 – 7.08 (m, 2H), 7.02 (d, *J* = 6.2 Hz, 1H), 5.99 (d, *J* =

7.1 Hz, 1H), 5.85 – 5.73 (m, 1H), 5.48 – 5.39 (m, 1H), 5.27 – 5.13 (m, 2H), 4.82 (dt, *J* = 8.0, 6.4 Hz, 1H), 4.59 – 4.39 (m, 9H), 4.35 (d, *J* = 7.2 Hz, 2H), 4.22 – 4.10 (m, 3H), 4.02 (t, *J* = 3.3 Hz, 1H), 3.59 (dd, *J* = 10.1, 6.2 Hz, 1H), 3.53 (dd, *J* = 10.1, 6.8 Hz, 1H), 3.11 (dd, *J* = 13.9, 6.1 Hz, 1H), 3.08 – 3.00 (m, 2H), 2.95 (dd, *J* = 14.2, 7.8 Hz, 1H).

^{13}C NMR (126 MHz, $CDCl_3$) δ 170.64, 170.06, 156.18, 143.91, 141.41, 138.08, 137.73, 137.39, 136.07, 131.64, 129.46, 128.68, 128.59, 128.56, 128.55, 128.47, 128.07, 128.02, 127.95, 127.92, 127.86, 127.84, 127.77, 127.24, 127.12, 125.28, 120.10, 118.97, 88.46, 84.26, 83.13, 82.42, 73.11, 72.52, 71.89, 70.36, 67.31, 66.06, 54.69, 53.82, 47.22, 38.01, 33.69.

HRMS (ESI): calc. for $C_{56}H_{56}O_9N_2SNa$ $[M+Na]^+$: 955.3599; found: 955.3593.

Allyl *N*-((((9*H*-fluoren-9-yl)methoxy)carbonyl)-L-valyl)-*S*-((2*S*,3*S*,4*R*,5*R*)-3,4-bis(benzyloxy)-5-((benzyloxy)methyl)tetrahydrofuran-2-yl)-L-cysteinyl-L-phenylalaninate

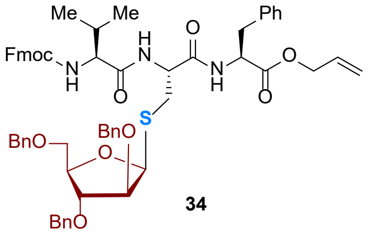

34

Prepared according to General Procedure **A** with DCM/MeCN (5:1) as solvent at room temperature for 15 h. The desired product **34** was obtained as a colorless oil (75 mg, 72%, α:β 1:20).

^{1}H NMR (500 MHz, $CDCl_3$) δ 7.75 (dd, *J* = 7.7, 2.0 Hz, 2H), 7.59 (dd, *J* = 8.2, 3.6 Hz, 2H), 7.39 (td, *J* = 7.5, 2.5 Hz, 2H), 7.31 – 7.20 (m, 20H), 7.15 (d, *J* = 7.6 Hz, 1H), 7.11 (d, *J* = 7.3 Hz, 2H), 6.93 (d, *J* = 6.7 Hz, 1H), 5.80 (ddt, *J* = 16.6, 11.5, 5.8 Hz, 1H), 5.54 (d, *J* = 8.0 Hz, 1H), 5.38 (d, *J* = 4.6 Hz, 1H), 5.26 – 5.13 (m, 2H), 4.80 (q, *J* = 6.4 Hz, 1H), 4.63 – 4.49 (m, 5H), 4.49 – 4.41 (m, 7H), 4.37 (t, *J* = 8.8 Hz, 1H), 4.21 (t, *J* = 6.9 Hz, 1H), 4.17 (td, *J* = 6.3, 3.4 Hz, 1H), 4.08 (dd, *J* = 4.5, 2.4 Hz, 2H), 3.93 (t, *J* = 3.0 Hz, 1H), 3.59 (dd, *J* = 10.3, 6.4 Hz, 1H), 3.52 (dd, *J* = 10.4, 6.2 Hz, 1H), 3.11 (dd, *J* = 14.3, 6.2 Hz, 1H), 3.06 – 2.97 (m, 1H), 2.97 – 2.87 (m, 1H), 0.91 (d, *J* = 6.5 Hz, 3H), 0.87 (d, *J* = 6.5 Hz, 4H).

^{13}C NMR (126 MHz, $CDCl_3$) δ 171.47, 170.60, 169.80, 156.53, 144.09, 143.95, 141.46, 138.00, 137.64, 137.42, 136.14, 131.72, 129.47, 128.61, 128.58, 128.52, 128.06, 128.01, 127.98, 127.96, 127.86, 127.85, 127.83, 127.25, 127.11, 125.21, 120.13, 120.11, 118.93, 88.68, 84.17, 83.19,

82.61, 73.06, 72.51, 71.88, 70.36, 67.04, 66.06, 60.41, 53.94, 53.29, 47.38, 37.99, 33.50, 31.38, 19.33, 17.86.

HRMS (ESI): calc. for $C_{56}H_{56}O_9N_2SNa$ $[M+Na]^+$: 955.3599; found: 955.3593.

Methyl *S*-((2*S*,3*S*,4*R*,5*R*)-4-(benzyloxy)-5-((benzyloxy)methyl)-3-fluorotetrahydrofuran-2-yl)-*N*-(*tert*-butoxycarbonyl)-L-cysteinate

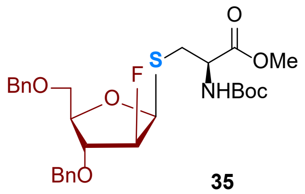

35

Prepared according to General Procedure **A** with MTBE/DCE (5:1) as solvent at 50 °C for 15 h. The desired product **35** was obtained as a colorless oil (29 mg, 53%, α:β 1:25).

^{1}H NMR (500 MHz, $CDCl_3$) δ 7.34 – 7.27 (m, 10H), 5.55 (d, J = 7.8 Hz, 1H), 5.19 (dd, J = 26.1, 3.5 Hz, 1H), 5.02 (ddd, J = 51.4, 3.5, 1.5 Hz, 1H), 4.59 (d, J = 3.5 Hz, 2H), 4.56 – 4.52 (m, 3H), 4.15 – 4.08 (m, 2H), 3.73 (s, 3H), 3.70 – 3.65 (m, 1H), 3.57 (dd, J = 10.4, 6.4 Hz, 1H), 3.24 (dd, J = 14.2, 4.7 Hz, 1H), 3.10 (dd, J = 14.3, 5.9 Hz, 1H), 1.43 (s, 9H).

^{13}C NMR (126 MHz, $CDCl_3$) δ 171.35, 155.43, 138.08, 137.12, 128.69, 128.56, 128.24, 128.05, 127.87, 96.82 (d, $J_{C\text{-}F}$ = 187.5 Hz), 86.17 (d, $J_{C\text{-}F}$ = 19.2 Hz), 83.41 (d, $J_{C\text{-}F}$ = 26.1 Hz), 82.87 (d, $J_{C\text{-}F}$ = 2.0 Hz), 80.22, 73.63, 72.32, 69.88 (d, $J_{C\text{-}F}$ = 1.5 Hz), 53.58, 52.73, 33.80, 28.46.

HRMS (ESI): calc. for $C_{28}H_{36}O_7FNSNa$ $[M+Na]^+$: 572.2089; found: 572.2080.

^{19}F NMR (471 MHz, $CDCl_3$) δ -191.52.

Methyl *N*-(((9*H*-fluoren-9-yl)methoxy)carbonyl)-*S*-((2*S*,3*S*,4*R*,5*R*)-4-(benzyloxy)-5-((benzyloxy)methyl)-3-fluorotetrahydrofuran-2-yl)-L-cysteinate

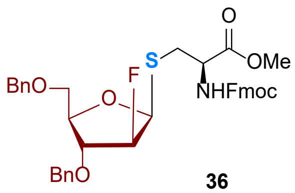

36

Prepared according to General Procedure **A** at 50 °C for 15 h. The desired product **36** was obtained as a colorless oil (32 mg, 48%, α:β 1:20).

^{1}**H NMR** (500 MHz, $CDCl_3$) δ 7.76 (d, J = 7.5 Hz, 2H), 7.60 (dd, J = 14.1, 7.5 Hz, 2H), 7.42 – 7.17 (m, 14H), 6.56 (d, J = 9.0 Hz, 1H), 5.44 (dd, J = 25.4, 4.0 Hz, 1H), 5.11 (dd, J = 50.4, 4.8 Hz, 1H), 4.76 (dt, J = 9.1, 4.4 Hz, 1H), 4.66 (d, J = 11.8 Hz, 1H), 4.60 (d, J = 11.7 Hz, 1H), 4.55 – 4.50 (m, 1H), 4.46 (q, J = 11.9 Hz, 2H), 4.31 (t, J = 7.6 Hz, 2H), 4.25 – 4.20 (m, 1H), 4.16 (t, J = 7.3 Hz, 1H), 3.79 – 3.70 (m, 5H), 3.41 (dd, J = 14.6, 5.0 Hz, 1H), 3.03 (dd, J = 14.6, 3.9 Hz, 1H).
^{13}**C NMR** (126 MHz, $CDCl_3$) δ 170.94, 156.27, 144.18, 143.97, 141.43, 141.39, 137.97, 137.22, 128.73, 128.45, 128.32, 127.92, 127.81, 127.77, 127.22, 125.45, 125.37, 120.07, 95.40 (d, $J_{C\text{-}F}$ = 189.4 Hz), 87.65 (d, $J_{C\text{-}F}$ = 17.6 Hz), 81.14 (d, $J_{C\text{-}F}$ = 24.9 Hz), 79.00, 73.62, 73.07, 67.30, 67.14, 54.28, 52.72, 47.23, 35.20.
HRMS (ESI): calc. for $C_{38}H_{38}O_7FNSNa$ $[M+Na]^+$: 694.2245; found: 694.2235.
^{19}**F NMR** (471 MHz, $CDCl_3$) δ -190.76.

Methyl *S*-((2*R*,3*R*,4*S*,5*R*)-3,4-bis(benzyloxy)-5-((benzyloxy)methyl)tetrahydrofuran-2-yl)-*N*-(*tert*-butoxycarbonyl)-L-cysteinate

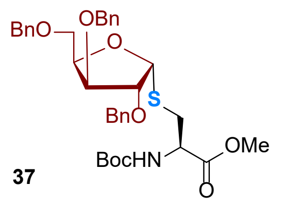

37

Prepared according to General Procedure **A** with MTBE/DCE (5:1) as solvent at 50 ºC for 15 h. The desired product **37** was obtained as a colorless oil (43 mg, 67%, α:β 25:1).
^{1}**H NMR** (400 MHz, $CDCl_3$) δ 7.33 – 7.26 (m, 12H), 7.26 – 7.24 (m, 3H), 5.57 (d, J = 8.3 Hz, 1H), 5.40 (d, J = 5.1 Hz, 1H), 4.59 – 4.47 (m, 7H), 4.20 – 4.15 (m, 1H), 4.12 (q, J = 5.9 Hz, 1H), 4.07 (td, J = 4.1, 1.5 Hz, 1H), 3.72 (s, 3H), 3.72-3.61 (m, 1H), 3.62 (dd, J = 10.4, 4.9 Hz, 1H), 3.21 (dd, J = 13.9, 5.6 Hz, 1H), 3.01 – 2.92 (m, 1H), 1.43 (s, 9H).
^{13}**C NMR** (126 MHz, $CDCl_3$) δ 171.70, 155.52, 138.24, 137.89, 137.43, 128.58, 128.54, 128.50, 128.11, 128.09, 127.92, 127.90, 127.87, 127.79, 87.35, 84.27, 83.45, 82.27, 73.44, 72.57, 72.06, 70.86, 53.33, 52.61, 33.23, 28.46.
HRMS (ESI): calc. for $C_{35}H_{43}O_8NSNa$ $[M+Na]^+$: 660.2602; found: 660.2589.

Methyl N-(((9H-fluoren-9-yl)methoxy)carbonyl)-S-((2R,3R,4S,5R)-3,4-bis(benzyloxy)-5-((benzyloxy)methyl)tetrahydrofuran-2-yl)-L-cysteinate

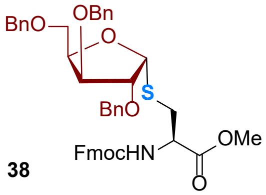

38

Prepared according to General Procedure **A** at 50 °C for 15 h. The desired product **38** was obtained as a colorless oil (52 mg, 68%, α:β 20:1).

^{1}H NMR (500 MHz, $CDCl_3$) δ 7.75 (d, *J* = 7.5 Hz, 2H), 7.59 (dd, *J* = 7.7, 4.8 Hz, 2H), 7.38 (t, *J* = 7.5 Hz, 2H), 7.34 – 7.22 (m, 17H), 5.96 (d, *J* = 7.8 Hz, 1H), 5.42 (d, *J* = 5.1 Hz, 1H), 4.64 – 4.45 (m, 7H), 4.37 (dd, *J* = 10.6, 7.3 Hz, 1H), 4.35 – 4.29 (m, 1H), 4.23 – 4.16 (m, 2H), 4.16 – 4.10 (m, 1H), 4.08 (t, *J* = 4.2 Hz, 1H), 3.74 (s, 3H), 3.67 (dd, *J* = 10.0, 5.9 Hz, 1H), 3.60 (dd, *J* = 10.0, 6.4 Hz, 1H), 3.25 (dd, *J* = 14.3, 4.8 Hz, 1H), 3.05 (dd, *J* = 14.3, 6.5 Hz, 1H).

^{13}C NMR (126 MHz, $CDCl_3$) δ 171.35, 156.15, 144.03, 143.97, 141.44, 138.17, 137.86, 137.40, 128.60, 128.56, 128.48, 128.12, 128.09, 127.96, 127.89, 127.88, 127.83, 127.78, 127.23, 125.33, 120.10, 87.43, 84.31, 83.29, 82.27, 73.37, 72.61, 72.10, 70.67, 67.27, 53.79, 52.75, 47.28, 33.21.

HRMS (ESI): calc. for $C_{45}H_{45}O_8NSNa$ $[M+Na]^+$: 782.2758; found: 782.2746.

Methyl S-((2R,3R,4S,5R)-4-(benzyloxy)-5-((benzyloxy)methyl)-3-fluorotetrahydrofuran-2-yl)-N-(*tert*-butoxycarbonyl)-L-cysteinate

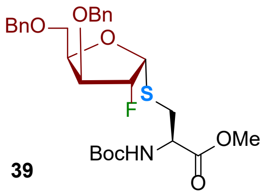

39

Prepared according to General Procedure **A** with MTBE/DCE (5:1) as solvent at 50 °C for 15 h. The desired product **39** was obtained as a colorless oil (33 mg, 60%, α:β 25:1).

^{1}H NMR (500 MHz, $CDCl_3$) δ 7.36 – 7.27 (m, 10H), 6.05 (d, *J* = 8.9 Hz, 1H), 5.37 (dd, *J* = 26.2, 3.8 Hz, 1H), 5.05 (ddd, *J* = 51.3, 4.0, 1.7 Hz, 1H), 4.66 – 4.52 (m, 5H), 4.51 – 4.46 (m, 1H), 4.20 (ddd, *J* = 10.7, 4.1, 1.7 Hz, 1H), 3.82 – 3.76 (m, 1H), 3.74 – 3.71 (m, 4H), 3.32 (dd, *J* = 14.3, 4.9 Hz, 1H), 2.99 (dd, *J* = 14.4, 4.0 Hz, 1H), 1.41 (s, 9H).

^{13}**C NMR** (126 MHz, $CDCl_3$) δ 171.31, 155.65, 138.12, 137.28, 128.71, 128.56, 128.28, 127.88, 127.84, 95.42 (d, $J_{C\text{-}F}$ = 192.4 Hz), 87.40 (d, $J_{C\text{-}F}$ = 17.5 Hz), 81.08 (d, $J_{C\text{-}F}$ = 24.9 Hz), 79.91, 78.97, 73.71, 73.06, 67.17, 53.85, 52.59, 35.00, 28.46.
HRMS (ESI): calc. for $C_{28}H_{36}O_7FNSNa$ $[M+Na]^+$: 572.2089; found: 572.2078.
^{19}**F NMR** (471 MHz, $CDCl_3$) δ -191.51.

Methyl *N*-(((9*H*-fluoren-9-yl)methoxy)carbonyl)-*S*-((2*R*,3*R*,4*S*,5*R*)-4-(benzyloxy)-5-((benzyloxy)methyl)-3-fluorotetrahydrofuran-2-yl)-L-cysteinate

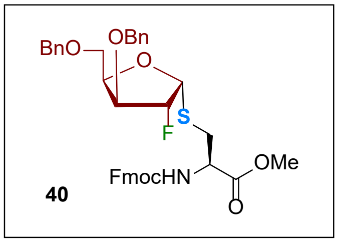

Prepared according to General Procedure **A** at 50 °C for 15 h. The desired product **40** was obtained as a colorless oil (46 mg, 68%, α:β 20:1).
^{1}**H NMR** (500 MHz, $CDCl_3$) δ 7.77 (d, *J* = 7.5 Hz, 2H), 7.61 (dd, *J* = 7.5, 5.1 Hz, 2H), 7.40 (t, *J* = 7.4 Hz, 2H), 7.36 – 7.23 (m, 12H), 5.95 (d, *J* = 7.7 Hz, 1H), 5.21 (dd, *J* = 25.9, 3.6 Hz, 1H), 5.04 (dd, *J* = 51.6, 4.6 Hz, 1H), 4.60 (dd, *J* = 16.2, 4.4 Hz, 3H), 4.50 (s, 2H), 4.47 – 4.26 (m, 2H), 4.23 (t, *J* = 7.1 Hz, 1H), 4.17 – 4.08 (m, 2H), 3.76 (s, 3H), 3.66 (dd, *J* = 10.6, 5.6 Hz, 1H), 3.59 – 3.52 (m, 1H), 3.29 (dd, *J* = 14.2, 4.8 Hz, 1H), 3.17 (dd, *J* = 14.3, 6.0 Hz, 1H).
^{13}**C NMR** (126 MHz, $CDCl_3$) δ 171.02, 156.04, 144.01, 143.90, 141.43, 141.42, 137.97, 137.05, 128.66, 128.49, 128.23, 128.01, 127.84, 127.82, 127.80, 127.22, 125.30, 120.10, 96.80 (d, $J_{C\text{-}F}$ = 188.7 Hz), 86.00 (d, $J_{C\text{-}F}$ = 18.7 Hz), 83.27 (d, $J_{C\text{-}F}$ = 26.2 Hz), 82.85, 73.52, 72.30, 69.74, 67.32, 53.95, 52.83, 47.25, 33.60.
HRMS (ESI): calc. for $C_{38}H_{38}O_7FNSNa$ $[M+Na]^+$: 694.2245; found: 694.2233.
^{19}**F NMR** (471 MHz, $CDCl_3$) δ -191.42.

***tert*-Butyl *S*-((2*R*,3*R*,4*S*,5*S*)-3,4-bis(benzyloxy)-5-((benzyloxy)methyl)tetrahydrofuran-2-yl)-*N*-(*tert*-butoxycarbonyl)-L-cysteinate**

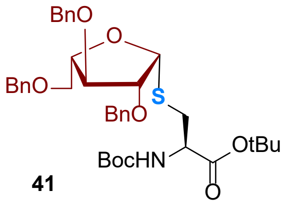

41

Prepared according to General Procedure **A** at 50 °C for 15 h. The desired product **41** was obtained as a colorless oil (42 mg, 62%, α:β 1:15).

^{1}H NMR (500 MHz, $CDCl_3$) δ 7.34 – 7.27 (m, 13H), 7.25 – 7.23 (m, 2H), 5.91 (d, *J* = 8.9 Hz, 1H), 5.34 (d, *J* = 5.0 Hz, 1H), 4.63 – 4.55 (m, 3H), 4.55 – 4.46 (m, 4H), 4.16 (t, *J* = 4.4 Hz, 2H), 4.07 (t, *J* = 4.0 Hz, 1H), 3.72 (dd, *J* = 10.0, 5.8 Hz, 1H), 3.65 (dd, *J* = 10.0, 6.8 Hz, 1H), 3.26 (dd, *J* = 14.1, 4.9 Hz, 1H), 2.95 (dd, *J* = 14.1, 4.4 Hz, 1H), 1.46 (s, 9H), 1.44 (s, 9H).

^{13}C NMR (126 MHz, $CDCl_3$) δ 170.00, 155.63, 138.31, 137.89, 137.47, 128.55, 128.53, 128.48, 128.04, 128.02, 127.95, 127.90, 127.84, 127.75, 88.37, 84.21, 83.61, 82.32, 82.20, 79.60, 73.45, 72.53, 72.05, 70.78, 54.28, 34.52, 28.51, 28.16.

HRMS (ESI): calc. for $C_{38}H_{49}O_8NSNa$ $[M+Na]^+$: 702.3071; found: 702.3052.

((2*R*,3*S*,4*S*,5*S*)-3,4-bis(benzyloxy)-5-(((*R*)-3-(*tert*-butoxy)-2-((*tert*-butoxycarbonyl)amino)-3-oxopropyl)thio)tetrahydrofuran-2-yl)methyl benzoate

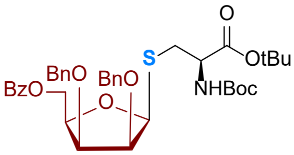

42

Prepared according to General Procedure **A** at 50 °C for 15 h. The desired product **42** was obtained as a colorless oil (36 mg, 52%, α:β 1:25).

^{1}H NMR (500 MHz, $CDCl_3$) δ 8.03 (dd, *J* = 8.2, 1.5 Hz, 2H), 7.57 – 7.51 (m, 1H), 7.45 – 7.38 (m, 4H), 7.37 – 7.26 (m, 8H), 5.49 (d, *J* = 7.8 Hz, 1H), 5.45 (d, *J* = 5.8 Hz, 1H), 4.84 (dd, *J* = 15.7, 11.8 Hz, 2H), 4.74 (dd, *J* = 11.6, 4.6 Hz, 1H), 4.68 – 4.58 (m, 3H), 4.44 (q, *J* = 6.2 Hz, 1H), 4.35 (dt, *J* = 7.3, 4.9 Hz, 1H), 4.21 (dd, *J* = 5.8, 4.5 Hz, 1H), 4.16 (t, *J* = 5.0 Hz, 1H), 3.22 (dd, *J* = 13.9, 4.7 Hz, 1H), 3.03 (dd, *J* = 14.0, 6.3 Hz, 1H), 1.44 (s, 9H), 1.42 (s, 9H).

^{13}C NMR (126 MHz, $CDCl_3$) δ 170.34, 166.50, 155.54, 138.07, 137.74, 133.72, 133.03, 130.32, 130.30, 129.87, 128.59, 128.55, 128.47, 128.42, 127.97, 127.90, 127.85, 127.78, 87.29, 82.41, 79.97, 78.12, 77.33, 73.57, 73.33, 64.77, 53.90, 34.10, 28.47, 28.09.

HRMS (ESI): calc. for $C_{38}H_{47}O_9NSNa$ $[M+Na]^+$: 716.2864; found: 716.2852.

Allyl *N*-(((9*H*-fluoren-9-yl)methoxy)carbonyl)-*O*-((2*S*,3*R*,4*S*,5*R*,6*R*)-3,4,5-tris(benzyloxy)-6-((benzyloxy)methyl)tetrahydro-2*H*-pyran-2-yl)-L-serinate

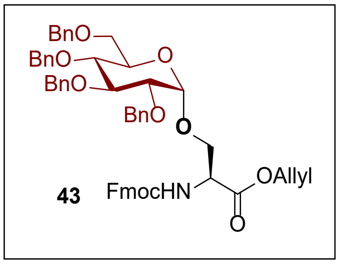

Prepared according to General Procedure **A** with MTBE/DCE (5:1) as solvent at 50 ºC for 15 h. The desired product **43** was obtained as a colorless oil (65 mg, 73%, α:β 18:1).

^{1}H NMR (500 MHz, $CDCl_3$) δ 7.68 (d, *J* = 7.5 Hz, 2H), 7.53 (d, *J* = 7.5 Hz, 2H), 7.29 – 7.17 (m, 22H), 7.07 (dd, *J* = 6.8, 2.8 Hz, 2H), 5.99 (d, *J* = 8.6 Hz, 1H), 5.81 (ddt, *J* = 16.5, 11.1, 5.8 Hz, 1H), 5.23 (d, *J* = 17.4 Hz, 1H), 5.13 (d, *J* = 10.5 Hz, 1H), 4.89 (d, *J* = 10.9 Hz, 1H), 4.76 (dd, *J* = 10.8, 4.3 Hz, 2H), 4.70 (d, *J* = 3.7 Hz, 1H), 4.64 (d, *J* = 11.9 Hz, 1H), 4.59 – 4.47 (m, 5H), 4.40 (dd, *J* = 16.7, 11.5 Hz, 2H), 4.28 (qd, *J* = 10.6, 7.3 Hz, 2H), 4.15 (t, *J* = 7.2 Hz, 1H), 4.08 (dd, *J* = 10.8, 3.7 Hz, 1H), 3.89 – 3.79 (m, 2H), 3.73 (dd, *J* = 9.9, 3.0 Hz, 1H), 3.64 (dd, *J* = 10.6, 3.8 Hz, 1H), 3.61 – 3.54 (m, 2H), 3.49 (dd, *J* = 9.7, 3.7 Hz, 1H).

^{13}C NMR (126 MHz, $CDCl_3$) δ 169.84, 156.21, 143.96, 141.39, 138.88, 138.32, 137.95, 131.67, 128.61, 128.52, 128.50, 128.48, 128.07, 128.05, 128.02, 128.01, 127.98, 127.92, 127.85, 127.81, 127.74, 127.24, 125.36, 125.30, 120.07, 118.93, 98.83, 81.89, 80.03, 77.64, 75.80, 75.29, 73.64, 73.20, 71.07, 70.13, 68.54, 67.46, 66.44, 54.81, 47.23.

HRMS (ESI): calc. for $C_{55}H_{59}O_{10}N_2$ $[M+NH_4]^+$: 907.4164; found: 907.4139.

Allyl *N*-(((9*H*-fluoren-9-yl)methoxy)carbonyl)-*S*-((2*R*,3*R*,4*S*,5*R*,6*R*)-3,4,5-tris(benzyloxy)-6-((benzyloxy)methyl)tetrahydro-2*H*-pyran-2-yl)-L-cysteinate

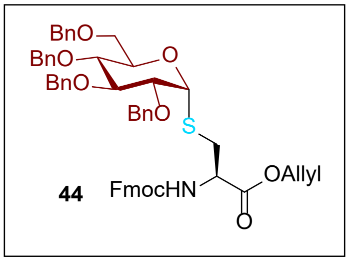

Prepared according to General Procedure **A** with MTBE/DCE (5:1) as solvent at 50 °C for 15 h. The desired product **44** was obtained as a colorless oil (65 mg, 70%, α:β 25:1).

^{1}H NMR (500 MHz, $CDCl_3$) δ 7.76 (dd, *J* = 7.7, 2.2 Hz, 2H), 7.62 (dd, *J* = 16.4, 7.5 Hz, 2H), 7.41 – 7.34 (m, 4H), 7.32 – 7.23 (m, 18H), 7.14 (dd, *J* = 6.8, 2.8 Hz, 2H), 6.33 (d, *J* = 8.7 Hz, 1H), 5.91 (ddt, *J* = 16.5, 11.0, 5.8 Hz, 1H), 5.38 – 5.30 (m, 1H), 5.27 – 5.22 (m, 2H), 4.94 (d, *J* = 11.0 Hz, 1H), 4.82 (d, *J* = 10.7 Hz, 1H), 4.77 (d, *J* = 10.9 Hz, 1H), 4.72 – 4.63 (m, 5H), 4.53 (d, *J* = 12.3 Hz, 1H), 4.48 (d, *J* = 10.9 Hz, 1H), 4.45 – 4.35 (m, 2H), 4.29 (dd, *J* = 10.5, 7.6 Hz, 1H), 4.23 (t, *J* = 7.3 Hz, 1H), 4.17 (ddd, *J* = 10.3, 4.6, 2.1 Hz, 1H), 3.82 (dd, *J* = 9.7, 5.3 Hz, 1H), 3.79 – 3.70 (m, 3H), 3.60 (dd, *J* = 10.3, 8.3 Hz, 1H), 3.32 (dd, *J* = 14.4, 5.7 Hz, 1H), 2.92 (dd, *J* = 14.4, 3.9 Hz, 1H).

^{13}C NMR (126 MHz, $CDCl_3$) δ 170.31, 156.05, 144.08, 143.95, 141.44, 141.42, 138.71, 138.23, 137.91, 137.79, 131.67, 128.64, 128.54, 128.53, 128.47, 128.23, 128.16, 128.14, 128.08, 128.01, 127.90, 127.83, 127.79, 127.26, 125.52, 125.31, 120.09, 119.16, 85.93, 82.41, 72.77, 71.61, 68.74, 67.35, 66.43, 54.47, 47.27, 34.54.

HRMS (ESI): calc. for $C_{55}H_{55}O_9NSNa$ $[M+Na]^+$: 928.3490; found: 928.3484.

Methyl *N*-(((9*H*-fluoren-9-yl)methoxy)carbonyl)-*S*-((2*R*,3*R*,4*S*,5*S*,6*R*)-3,4,5-tris(benzyloxy)-6-((benzyloxy)methyl)tetrahydro-2*H*-pyran-2-yl)-L-cysteinate

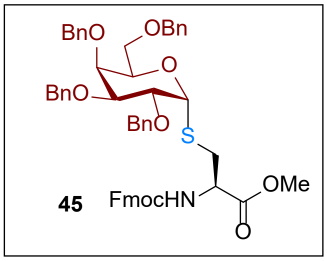

Prepared according to General Procedure **A** at 50 °C for 15 h. The desired product **45** was obtained as a colorless oil (54 mg, 61%, α:β 20:1).

^{1}H NMR (500 MHz, $CDCl_3$) δ 7.73 (dd, *J* = 7.6, 4.3 Hz, 2H), 7.59 (t, *J* = 7.8 Hz, 2H), 7.39 – 7.28 (m, 24H), 6.36 (d, *J* = 8.6 Hz, 1H), 5.32 (d, *J* = 5.5 Hz, 1H), 4.95 (d, *J* = 11.3 Hz, 1H), 4.84 (d, *J* = 11.7 Hz, 1H), 4.71 (d, *J* = 9.0 Hz, 3H), 4.61 (dt, *J* = 9.4, 4.8 Hz, 1H), 4.57 (d, *J* = 11.3 Hz, 1H), 4.46 (d, *J* = 11.9 Hz, 1H), 4.40 – 4.26 (m, 4H), 4.24 (t, *J* = 6.3 Hz, 1H), 4.18 (t, *J* = 7.2 Hz, 1H), 3.90 (d, *J* = 3.0 Hz, 1H), 3.75 – 3.67 (m, 4H), 3.61 (dd, *J* = 9.5, 6.6 Hz, 1H), 3.47 (dd, *J* = 9.4, 5.8 Hz, 1H), 3.29 (dd, *J* = 14.6, 6.0 Hz, 1H), 2.90 (dd, *J* = 14.6, 4.1 Hz, 1H).

^{13}C NMR (126 MHz, $CDCl_3$) δ 171.19, 156.01, 144.07, 144.01, 141.44, 141.44, 138.75, 138.59, 138.18, 137.92, 128.53, 128.52, 128.50, 128.42, 128.09, 127.96, 127.90, 127.85, 127.83, 127.77, 127.72, 127.64, 127.24, 127.21, 125.38, 125.34, 120.06, 120.05, 120.04, 86.26, 79.30, 76.29, 75.20, 75.03, 73.58, 72.83, 70.70, 69.19, 67.04, 54.35, 52.62, 47.29, 34.01.

HRMS (ESI): calc. for $C_{53}H_{57}O_9N_2S$ $[M+NH_4]^+$: 897.3779; found: 897.3754.

Methyl *S*-((3a*S*,6*R*,7*R*,7a*S*)-7-(benzyloxy)-2,2-dimethyltetrahydro-4*H*-[1,3]dioxolo[4,5-c]pyran-6-yl)-*N*-(*tert*-butoxycarbonyl)-L-cysteinate

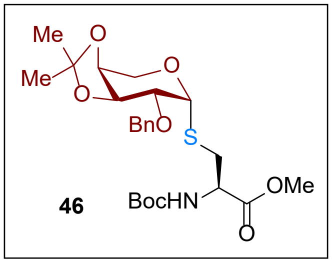

Prepared according to General Procedure **A** with MTBE/DCE (5:1) as solvent at 50 ºC for 15 h. The desired product **46** was obtained as a colorless oil (30 mg, 60%, α:β 25:1).

^{1}H NMR (500 MHz, $CDCl_3$) δ 7.39 – 7.32 (m, 4H), 7.31 – 7.28 (m, 1H), 5.75 (d, *J* = 8.7 Hz, 1H), 5.17 (d, *J* = 4.9 Hz, 1H), 4.76 – 4.64 (m, 2H), 4.65 – 4.58 (m, 1H), 4.26 – 4.16 (m, 3H), 3.87 (d, *J* = 12.3 Hz, 1H), 3.80 (dd, *J* = 6.0, 4.9 Hz, 1H), 3.75 (s, 3H), 3.20 (dd, *J* = 14.1, 4.9 Hz, 1H), 2.91 (dd, *J* = 14.2, 4.3 Hz, 1H), 1.44 (s, 9H), 1.42 (s, 3H), 1.34 (s, 3H).

^{13}C NMR (126 MHz, $CDCl_3$) δ 171.55, 155.51, 137.67, 128.56, 128.11, 128.08, 109.39, 83.86, 80.13, 75.84, 74.24, 72.96, 72.55, 60.93, 53.63, 52.63, 33.92, 28.49, 27.91, 26.17.

HRMS (ESI): calc. for $C_{24}H_{39}O_8N_2S$ $[M+NH_4]^+$: 515.2422; found: 515.2425.

(3*S*,4*S*,5*R*,6*R*)-5-(benzyloxy)-6-(((*R*)-2-((*tert*-butoxycarbonyl)amino)-3-methoxy-3-oxopropyl)thio)tetrahydro-2*H*-pyran-3,4-diyl diacetate

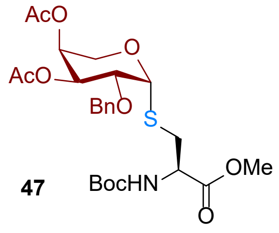

47

Prepared according to General Procedure **A** with MTBE/DCE (5:1) as solvent at 50 ºC for 15 h. The desired product **47** was obtained as a colorless oil (32 mg, 60%, α:β 20:1).

^{1}H NMR (600 MHz, C_6D_6) δ 7.29 (d, *J* = 7.3 Hz, 2H), 7.13 (t, *J* = 7.6 Hz, 2H), 7.05 (t, *J* = 7.3 Hz, 1H), 5.74 (d, *J* = 9.1 Hz, 1H), 5.49 (dd, *J* = 9.5, 3.4 Hz, 1H), 5.38 (dd, *J* = 3.4, 1.8 Hz, 2H), 4.74 (dt, *J* = 9.2, 4.8 Hz, 1H), 4.49 (d, *J* = 11.9 Hz, 1H), 4.28 (d, *J* = 11.7 Hz, 1H), 4.08 (dd, *J* = 9.5, 4.9 Hz, 1H), 3.91 (dd, *J* = 12.9, 2.1 Hz, 1H), 3.47 (dd, *J* = 12.8, 3.3 Hz, 1H), 3.22 (s, 3H), 2.93 (dd, *J* = 14.2, 4.9 Hz, 1H), 2.67 (dd, *J* = 14.2, 4.9 Hz, 1H), 1.68 (s, 3H), 1.67 (s, 3H), 1.42 (s, 9H).

^{13}C NMR (101 MHz, $CDCl_3$) δ 171.50, 169.45, 169.36, 155.57, 138.19, 128.60, 127.98, 127.92, 84.90, 79.90, 74.42, 72.00, 69.34, 69.26, 61.67, 54.01, 51.90, 32.99, 28.40, 20.43.

HRMS (ESI): calc. for $C_{25}H_{35}O_{10}NSNa$ $[M+Na]^+$: 564.1874; found: 564.1870.

(2*S*,3*R*,4*R*,5*S*,6*S*)-5-(benzyloxy)-6-(((*R*)-2-((*tert*-butoxycarbonyl)amino)-3-methoxy-3-oxopropyl)thio)-2-methyltetrahydro-2*H*-pyran-3,4-diyl diacetate

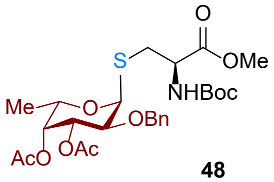

48

Prepared according to General Procedure **A** with MTBE/DCE (5:1) as solvent at 50 ºC for 15 h. The desired product **48** was obtained as a colorless oil (39 mg, 53%, α:β 20:1).

^{1}H NMR (500 MHz, $CDCl_3$) δ 7.36 – 7.27 (m, 5H), 5.53 (d, *J* = 5.5 Hz, 1H), 5.33 – 5.27 (m, 1H), 5.26 (dd, *J* = 3.4, 1.2 Hz, 1H), 5.14 (dd, *J* = 10.5, 3.4 Hz, 1H), 4.69 (d, *J* = 12.0 Hz, 1H), 4.62 – 4.53 (m, 2H), 4.40 – 4.35 (m, 1H), 4.05 (dd, *J* = 10.4, 5.5 Hz, 1H), 3.76 (s, 3H), 3.06 (dd, *J* = 13.8, 4.8 Hz, 1H), 2.82 (dd, *J* = 13.8, 6.4 Hz, 1H), 2.12 (s, 3H), 1.97 (s, 3H), 1.43 (s, 9H), 1.14 (d, *J* = 6.5 Hz, 3H).

^{13}C NMR (126 MHz, $CDCl_3$) δ 171.54, 170.56, 170.07, 155.30, 137.76, 128.52, 128.02, 127.88, 84.06, 80.34, 72.84, 72.25, 71.27, 70.53, 65.35, 53.19, 52.78, 32.33, 28.45, 20.78, 16.03.

HRMS (ESI): calc. for $C_{26}H_{38}O_{10}NS$ $[M+H]^+$: 556.2211; found: 556.2190.

(2*S*,3*R*,4*R*,5*S*,6*S*)-6-(((*R*)-2-((((9*H*-fluoren-9-yl)methoxy)carbonyl)amino)-3-methoxy-3-oxopropyl)thio)-5-(benzyloxy)-2-methyltetrahydro-2*H*-pyran-3,4-diyl diacetate

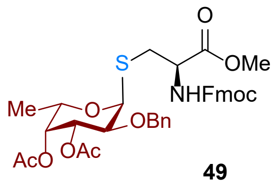

49

Prepared according to General Procedure **A** with MTBE/DCE (5:1) as solvent at 50 ºC for 15 h. The desired product **49** was obtained as a colorless oil (44 mg, 64%, α:β 18:1).

^{1}H NMR (500 MHz, $CDCl_3$) δ 7.76 (dd, *J* = 7.6, 1.0 Hz, 2H), 7.60 – 7.53 (m, 2H), 7.42 – 7.37 (m, 2H), 7.33 – 7.27 (m, 6H), 7.25 – 7.20 (m, 1H), 5.61 – 5.53 (m, 2H), 5.29 – 5.24 (m, 1H), 5.15 (dd, *J* = 10.6, 3.3 Hz, 1H), 4.71 – 4.59 (m, 2H), 4.53 (d, *J* = 11.9 Hz, 1H), 4.40 – 4.33 (m, 3H), 4.24 – 4.18 (m, 1H), 4.06 (dd, *J* = 10.4, 5.5 Hz, 1H), 3.79 (s, 3H), 3.10 (dd, *J* = 14.1, 4.4 Hz, 1H), 2.87 (dd, *J* = 13.8, 6.4 Hz, 1H), 2.12 (s, 3H), 1.97 (s, 3H), 1.14 (d, *J* = 6.5 Hz, 3H).

^{13}C NMR (126 MHz, $CDCl_3$) δ 171.17, 170.55, 170.09, 155.90, 143.94, 143.89, 141.46, 141.45, 137.77, 128.52, 128.24, 128.03, 127.89, 127.82, 127.64, 127.61, 127.25, 125.27, 125.20, 120.15, 83.81, 72.96, 72.31, 71.25, 70.54, 67.37, 65.43, 53.70, 52.97, 47.26, 32.02, 20.92, 20.78, 16.06.

HRMS (ESI): calc. for $C_{36}H_{39}O_{10}NSNa$ $[M+Na]^+$: 700.2187; found: 700.2177.

(3a*R*,5*S*,5a*R*,8a*S*,8b*R*)-5-((((2*R*,3*S*,4*R*,5*R*)-3,4-bis(benzyloxy)-5-((benzyloxy)methyl)tetra-hydrofuran-2-yl)thio)methyl)-2,2,7,7-tetramethyltetrahydro-5*H*-bis([1,3]dioxolo)[4,5-*b*:4',5'-*d*]pyran

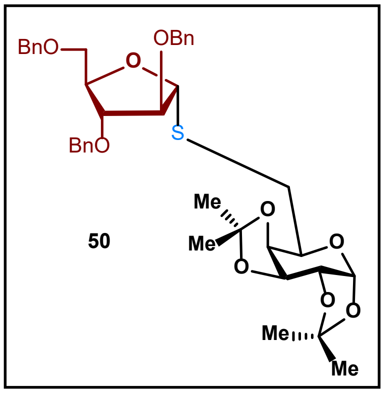

50

Prepared according to General Procedure **A** with PMP base (2 equiv.) at 50 °C for 15 h. The desired product **50** was obtained as a colorless oil (18 mg, 26%, α:β 20:1).

^{1}H NMR (500 MHz, $CDCl_3$) δ 7.27 – 7.17 (m, 15H), 5.48 (d, *J* = 5.1 Hz, 1H), 5.38 (d, *J* = 2.0 Hz, 1H), 4.56 (d, *J* = 11.6 Hz, 1H), 4.53 (dd, *J* = 7.9, 2.4 Hz, 1H), 4.50 – 4.37 (m, 5H), 4.28 (dd, *J* = 7.9, 1.9 Hz, 1H), 4.23 (ddd, *J* = 8.0, 5.7, 2.4 Hz, 2H), 3.96 (td, *J* = 7.4, 1.9 Hz, 1H), 3.93 (dd, *J* = 4.2, 1.9 Hz, 2H), 3.58 (dd, *J* = 10.9, 3.9 Hz, 1H), 3.54 (dd, *J* = 10.9, 4.7 Hz, 1H), 2.90 (dd, *J* = 13.7, 7.5 Hz, 1H), 2.78 (dd, *J* = 13.7, 7.3 Hz, 1H), 1.42 (s, 3H), 1.38 (s, 3H), 1.26 (s, 3H), 1.23 (s, 3H).

^{13}C NMR (126 MHz, $CDCl_3$) δ 138.25, 137.93, 137.61, 128.53, 128.49, 128.47, 128.07, 127.98, 127.96, 127.87, 127.86, 127.72, 109.39, 108.73, 96.90, 88.88, 88.13, 83.76, 80.20, 73.52, 72.38, 72.07, 71.65, 71.15, 70.71, 69.15, 66.55, 31.48, 26.17, 26.15, 25.09, 24.66.

HRMS (ESI): calc. for $C_{38}H_{47}O_9S$ $[M+H]^+$: 679.2935; found: 679.2921.

(2*R*,3*R*,4*S*,5*R*,6*S*)-2-((((2*R*,3*S*,4*R*,5*R*)-3,4-bis(benzyloxy)-5-((benzyloxy)methyl)tetrahydro-furan-2-yl)oxy)methyl)-6-methoxytetrahydro-2*H*-pyran-3,4,5-triyl tribenzoate

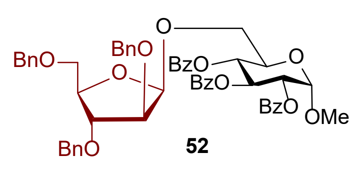

52

Prepared according to General Procedure **B** at room temperature for 6 h. The desired product **52** was obtained as a colorless oil (80 mg, 88%, α:β 1:8). The ^{1}H NMR of compound **52** matches the literature report.[8]

^{1}H NMR (400 MHz, $CDCl_3$) δ 7.98 – 7.95 (m, 2H), 7.93 – 7.90 (m, 2H), 7.83 – 7.80 (m, 2H), 7.52 – 7.19 (m, 24H), 6.11 (t, J = 9.7 Hz, 1H), 5.52 (t, J = 9.8 Hz, 1H), 5.24 – 5.16 (m, 2H), 5.05 (d, J = 3.1 Hz, 1H), 4.80 (d, J = 11.7 Hz, 1H), 4.65 - 4.40 (m, 3H), , 4.47 (d, J = 12.1 Hz, 1H), 4.42 (d, J = 12.0 Hz, 1H), 4.21 – 4.16 (m, 1H), 4.12 - 4.07 (m, 3H), 3.88 (dd, J = 11.6, 2.3 Hz, 1H), 3.60 (dd, J = 11.6, 5.8 Hz, 1H), 3.52 (d, J = 5.6 Hz, 2H), 3.35 (s, 3H).

(2*R*,3*R*,4*S*,5*R*)-3,4-bis(benzyloxy)-2-((benzyloxy)methyl)-5-(2-bromo-2-methylpropoxy)-tetrahydrofuran

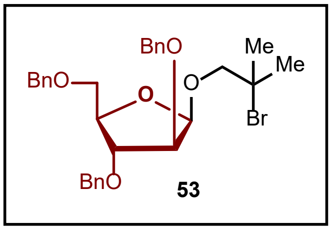

Prepared according to General Procedure **A** with IBO (2 equiv.) at 50 ºC for 15 h. The desired product **53** was obtained as a colorless oil (8 mg, 7%, α:β 1:1).

^{1}H NMR (500 MHz, $CDCl_3$) δ 7.39 – 7.27 (m, 30H), 5.31 (d, J = 4.3 Hz, 1H), 5.04 (d, J = 3.5 Hz, 1H), 4.73 – 4.66 (m, 4H), 4.60 (dd, J = 12.2, 6.0 Hz, 3H), 4.57 – 4.53 (m, 5H), 4.17 – 4.03 (m, 6H), 3.74 (d, J = 10.4 Hz, 1H), 3.64 (dd, J = 9.7, 6.5 Hz, 1H), 3.59 (dd, J = 9.8, 5.0 Hz, 1H), 3.57 – 3.54 (m, 2H), 3.48 (d, J = 10.4 Hz, 1H), 3.44 (d, J = 10.4 Hz, 1H), 3.36 (d, J = 10.4 Hz, 1H), 1.70 (s, 3H), 1.68 (s, 3H), 1.37 (s, 3H), 1.37 (s, 3H).

^{13}C NMR (126 MHz, $CDCl_3$) δ 138.41, 138.39, 138.18, 138.00, 137.97, 128.57, 128.56, 128.53, 128.49, 128.14, 128.10, 128.08, 128.00, 127.98, 127.88, 127.85, 127.83, 127.77, 101.20, 95.69, 84.55, 83.91, 83.51, 83.35, 80.49, 80.01, 77.69, 76.17, 73.51, 73.44, 72.74, 72.57, 72.49, 63.44, 42.49, 31.14, 31.07, 25.59, 25.38.

HRMS (ESI): calc. for $C_{30}H_{39}BrNO_5$ $[M+H]^+$: 572.2006; found: 572.1962.

3 Kinetic Experiments

3.1 Preparation of 4-methylphenyl 1-thio-, 2,3,5-tri-*O*-benzyl-*d*$_7$-a-D-arabinofuranoside

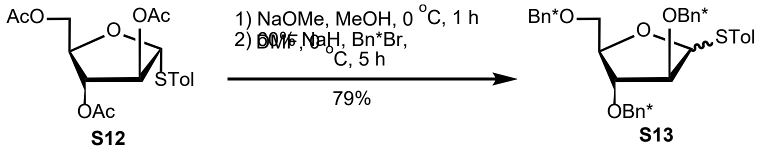

To compound **S12**[10] (450 mg, 1.18 mmol) was dissolved in 3 mL dry MeOH in a 50 mL round-bottom flask and cooled to 0 ºC. Sodium methoxide (19.2 mg, 0.35 mmol) was added to the solution. The resulting mixture was stirred for 1 h, quenched with acid resin, filtered, and evaporated to dryness. The crude product was used without further purification. To the solution of the crude product in dried DMF (3 mL) was added benzyl bromide-d7 (0.56 mL, 4.68 mmol). The solution was then cooled to 0 ºC in an ice bath, and sodium hydride (60% in mineral oil) (187 mg, 4.68 mmol) was added in 2 portions. The mixture was stirred in an ice bath overnight. The resulting solution was poured into chipped ice (~ 100 mL) and extracted with ethyl acetate (200 mL). The organic solution was washed with brine, dried over sodium sulfate, and then concentrated *in vacuo*. The residue was then subjected to silica gel column chromatography to give **S13** (504 mg, 79%) as a colorless oil; R_f 0.34 (1:4, EtOAc/hexanes); **^{1}H NMR** (500 MHz, $CDCl_3$) δ 7.42 (dd, *J* = 16.3, 8.1 Hz, 4H), 7.09 (dd, *J* = 16.6, 8.2 Hz, 4H), 5.53 (d, *J* = 2.9 Hz, 1H), 4.84 (dd, *J* = 6.5, 2.3 Hz, 1H), 4.38 (dt, *J* = 6.8, 4.2 Hz, 1H), 4.26 (ddd, *J* = 12.1, 5.9, 2.2 Hz, 1H), 4.11 (q, *J* = 3.0 Hz, 1H), 4.03 (dd, *J* = 6.7, 3.3 Hz, 1H), 3.91 (td, *J* = 6.5, 2.4 Hz, 1H), 3.81 (dt, *J* = 5.6, 2.8 Hz, 1H), 3.71 – 3.60 (m, 3H), 3.43 (dt, *J* = 12.0, 2.3 Hz, 1H), 2.33 (s, 3H), 2.32 (s, 3H). **HRMS (ESI)**: calc. for $C_{33}H_{17}D_{21}O_4NS$ $[M+NH_4]^+$: 565.3834; found: 565.3811.

3.2 Kinetic Experiment Setup and Data Acquisition

We used initial-rate quench kinetics to track the reaction due to its heterogeneity and relatively slow pace. We divided the reaction mixture into aliquots at different time intervals and diluted them into CD_2Cl_2 to stop the reaction. Analysis of these samples using ^{1}H NMR on the same day showed no change in the product concentration

The 2,3,5-tri-benzyl-*d*$_7$-arabinofuranosyl bromide donor **1*** was generated *in situ* by following General Procedure **1** (see section **2.1**) from compound **S12**. This bromide **1*** was used without further purification. To a 0.5 dram vial equipped with a stir bar and PTFE cap was added bromide **1*** (76.7 mg, 0.15 mmol, 3 equiv), acceptor (0.05 mmol, 1 equiv), phenanthroline catalyst, DTBMP (0.075 mmol, 1.5 equiv), 1,3-dinitrobenzene (0.05 mmol, 1 equiv) as internal standard, and anhydrous CD_2Cl_2 (amount specified in table below). The reaction was stirred at 23 °C under an argon atmosphere. The PTFE cap of the 0.5-dram vial was punctured with a 25 μL glass syringe, and 20 μL of the solution was removed and immediately injected into a new 0.5-dram vial

containing 400 μL of CD_2Cl_2. The mixture was pushed through a syringe filter into an NMR tube. The sample was analyzed by ^{1}H NMR analysis with 16 scans and 10 seconds of relaxation delay. We measured the concentration with respect to time by monitoring the formation of the furanosylated product and then integrating its C1-anomeric proton relative to a peak of 1,3-dinitrobenzene at 9.04 ppm as the internal standard. For the alcohol acceptor, C1-anomeric proton peaks corresponding to the α-product (at 4.96 ppm) and the β-product (at 5.11 ppm) were detected. Conversely, with the thiol acceptor, only the β-product peak (at 5.53 ppm) was evident. These C1-anomeric proton peaks were selected due to their clarity from other peaks. Peak fitting or deconvolution algorithms were not used for integration. The representative example of the NMR kinetics time course for oxygen acceptor, featuring time points at 0, 10, 20, 30, 40, 50, and 60 minutes, arranged from bottom to top is shown in Figure **S1**. Figure **S2** represents the NMR time course for the thiol acceptor featuring time points at 0, 15, 30, and 45 minutes, arranged from bottom to top. Furthermore, in each instance, the product concentration recorded at 0 minutes was subtracted from all subsequent time points to ensure visually appealing charts.

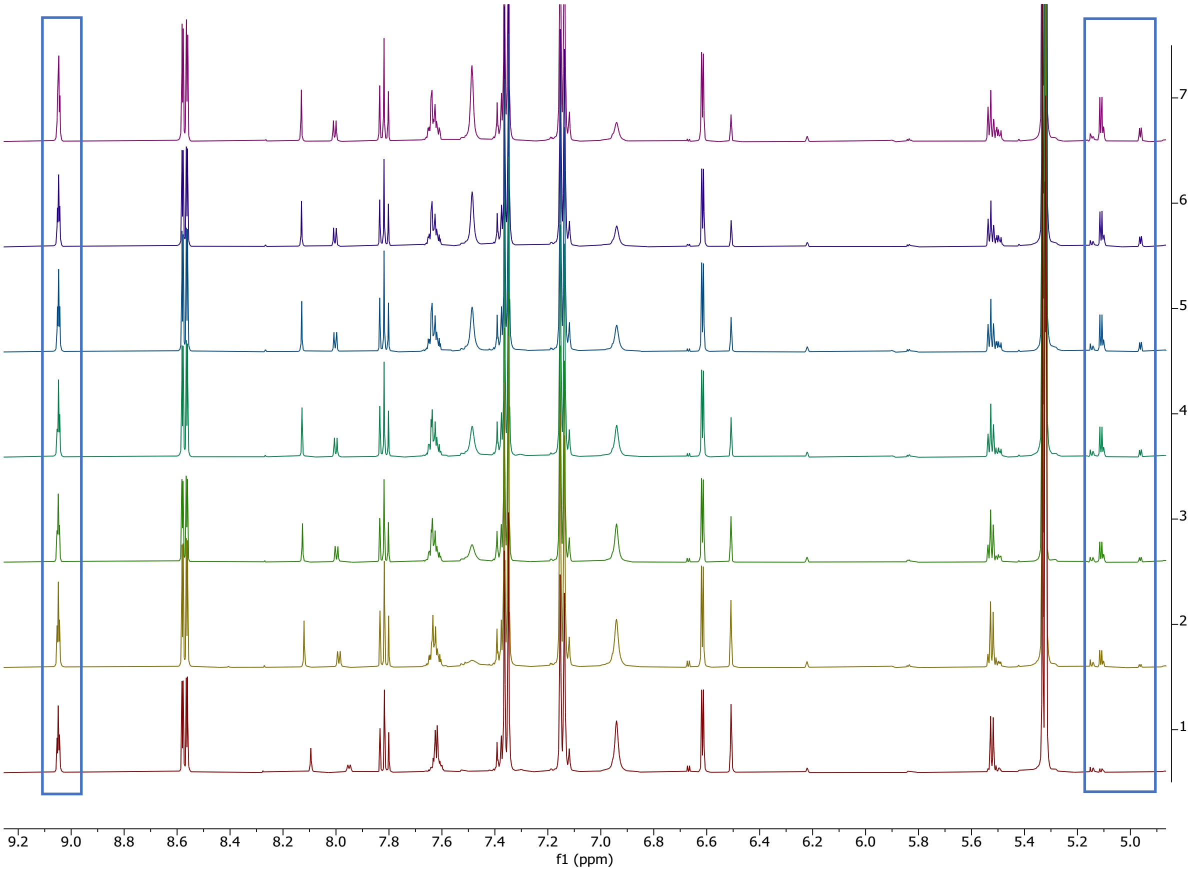

Figure S1: Representative example of ^{1}H NMR time course for the reaction of oxygen acceptor.

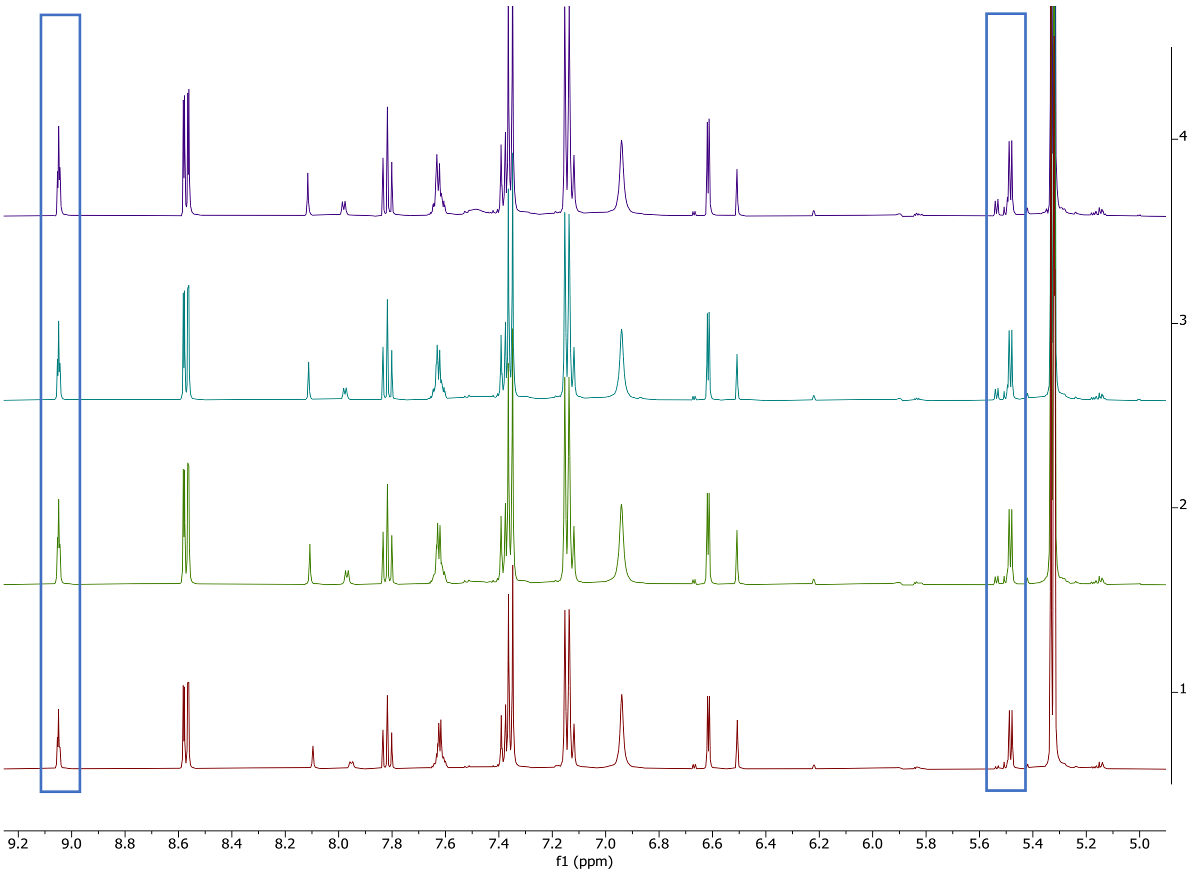

Figure S2: Representative example of ^{1}H NMR time course for the reaction of thiol acceptor.

3.3 Initial Rate Comparison Between Oxygen and Sulfur Acceptor

The initial rates of the reaction between arabinosyl bromide donor **1*** with alcohol acceptor **2** and thiol acceptor **4** were measured using both BPhen and NPhen catalysts. The procedure shown in section **3.2** was followed with bromide **1*** (76.7 mg, 0.15 mmol, 3 equiv), acceptor (0.05 mmol, 1 equiv), BPhen (0.0075 mmol, 5 mol%), DTBMP (0.075 mmol, 1.5 equiv), 1,3-dinitrobenzene (0.05 mmol, 1 equiv), and anhydrous CD_2Cl_2 (0.25 mL, 0.2 M). The reaction was stirred at 25 °C under an argon atmosphere. The observed concentrations are shown in Table **S2**.

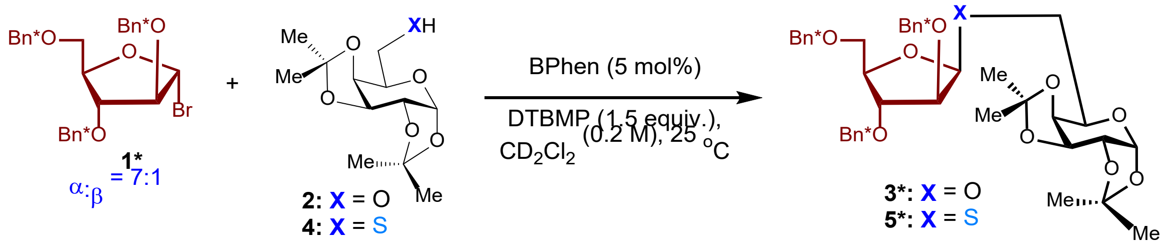

Alcohol as acceptor			Thiol as acceptor			
Time (h)	[Product] (M)		Time (h)	[Product] (M)	Time (h)	[Product] (M)
0	0		0	0	0.5	0.0117
0.167	0.032		0.083	0.0027	0.583	0.0138
0.333	0.0627		0.17	0.0046	0.67	0.0148
0.5	0.0828		0.25	0.0073	0.75	0.0164
0.68	0.102		0.33	0.0091		
0.83	0.1153		0.42	0.0103		

Table S2: Furanosylated product concentration at different time intervals under BPhen catalyst.

The kinetic plot for the alcohol acceptor and thiol acceptor is shown in Figure **S3**. For instance, at 0.3 h interval of time, *O*-furanosylation product **3*** (~ 0.06 M) vs *S*-furanosylated product **5*** (~ 0.012 M) were formed. This result clearly demonstrates that the rate of reaction with alcohol acceptor is nearly five times faster than the reaction with thiol acceptor.

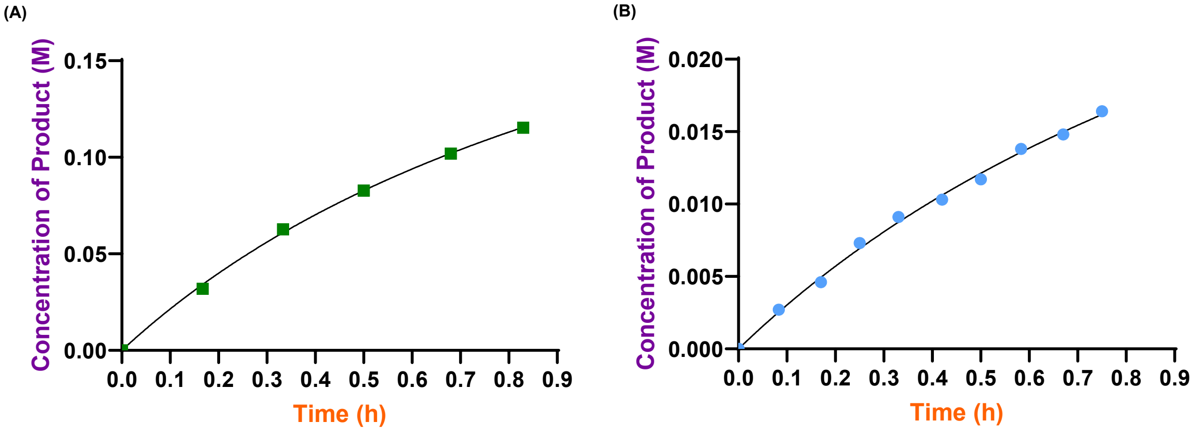

Figure S3: (**A**) Kinetic plot for the reaction with alcohol acceptor. (**B**) Kinetic plot for the reaction with thiol acceptor.

The procedure shown in section **3.2** was followed with bromide donor **1*** (76.7 mg, 0.15 mmol, 3 equiv), acceptor (0.05 mmol, 1 equiv), NPhen (0.0075 mmol, 5 mol%), DTBMP (0.075 mmol, 1.5 equiv), 1,3-dinitrobenzene (0.05 mmol, 1 equiv), and anhydrous CD_2Cl_2 (0.25 mL, 0.2 M). The reaction was stirred at 23 °C under an argon atmosphere. The measured concentrations are shown in Table **S3**.

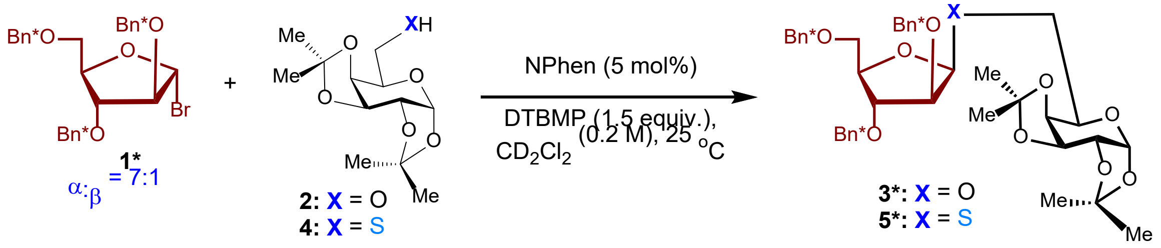

Alcohol as acceptor			Thiol as acceptor	
Time (h)	[Product] (M)		Time (h)	[Product] (M)
0	0		0	0
0.083	0.0192		0.083	0.0042
0.17	0.0457		0.17	0.0107
0.25	0.0626		-	-
0.33	0.0782		0.33	0.0233
0.42	0.0927		0.42	0.027
0.5	0.1008		0.5	0.0323
0.583	0.1095		0.583	0.0374
0.67	0.1184		0.67	0.0388
0.75	0.1205			

Table S3: Furanosylated product concentration at different time intervals under NPhen catalyst.

The kinetic plot for alcohol acceptor and thiol acceptor was shown in Figure **S4**. For instance, at a 0.3 h interval of time, *O*-furanosylation product **3*** (~ 0.075 M) vs *S*-furanosylated product **5*** (~ 0.017 M) were formed. This result clearly demonstrates that the rate of reaction with alcohol acceptor is nearly five times faster than the reaction with thiol acceptor.

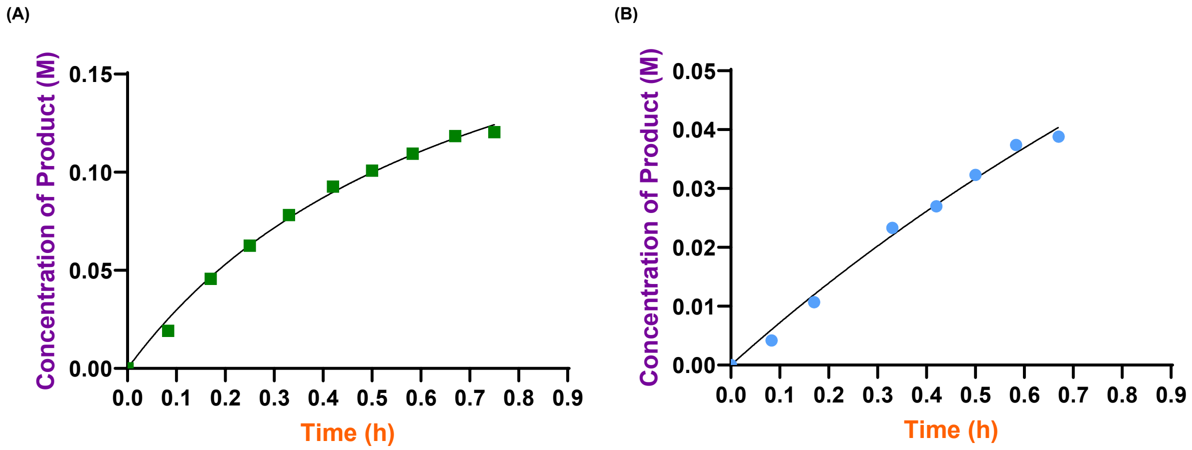

Figure S4: (**A**) Kinetic plot for the reaction with alcohol acceptor. (**B**) Kinetic plot for the reaction with thiol acceptor.

3.4 ^{1}H NMR Data for Titration Experiments

^{1}H NMR titrations were carried out using a Bruker 500 MHz spectrometer. The stock solutions of acceptor **2** (0.02 M), acceptor **4** (0.02 M), and TBAB (0.1 M) in $CDCl_3$ were prepared. The NMR samples (0.6 mL) were prepared by adding an increasing amount of TBAB stock solution to the host acceptor solution (0.3 mL). The NMR spectra were stacked together by referencing with residual TMS peak (δ 0.00 ppm) to see the observed changes in chemical shift due to hydrogen bonding. Figures S5 and S6 show that the alcohol acceptor **2** has a strong hydrogen bonding interaction with the bromide ion compared to the thiol acceptor **4**. Also, we did not observe the shift of the adjacent CH protons in the case of both alcohol and thiol acceptors.

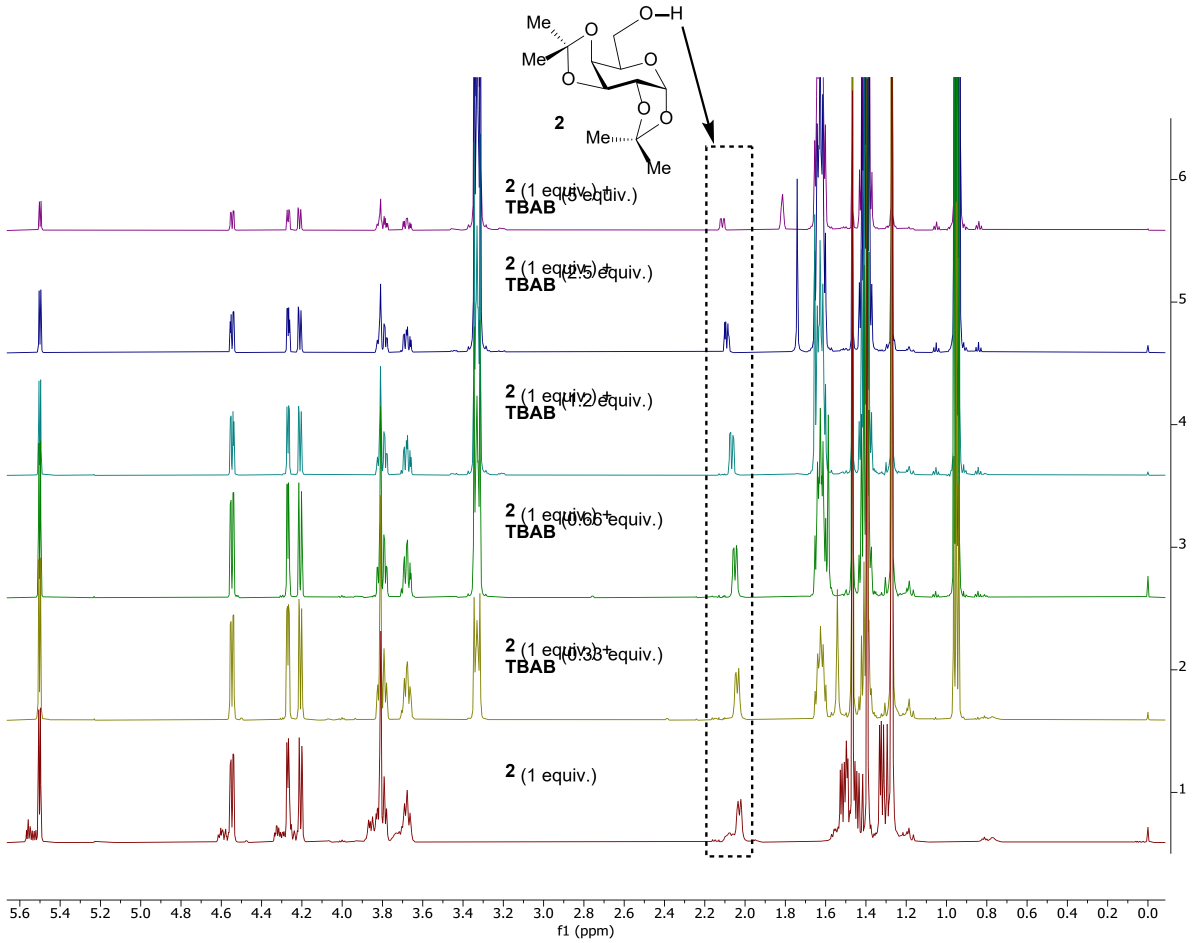

Figure S5: ^{1}H NMR titration experiment between alcohol acceptor **2** and TBAB.

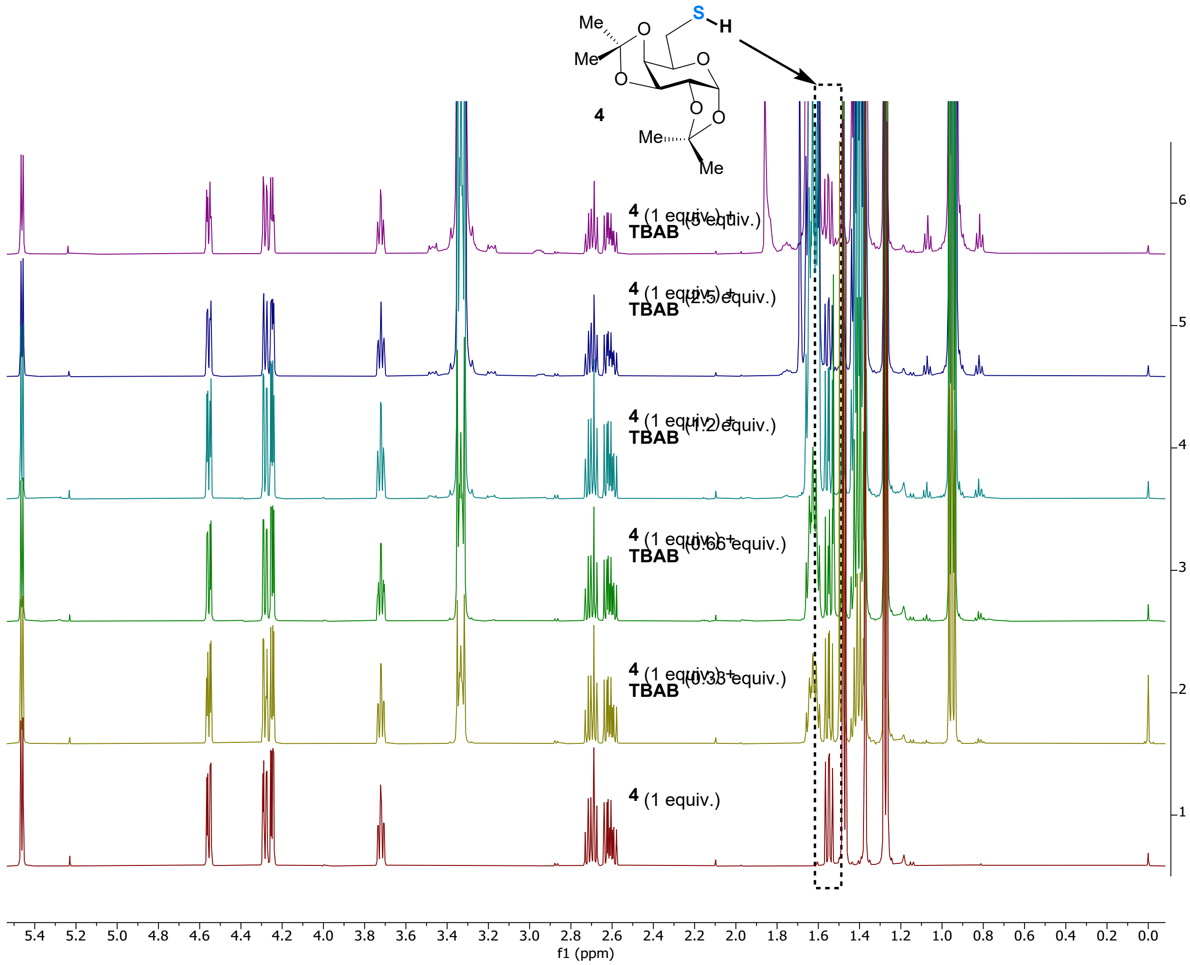

Figure S6: ^{1}H NMR titration experiment between thiol acceptor **4** and TBAB.

4 Computational Study

All calculations were carried out using density functional theory (DFT) implemented in Gaussian 16[11] using the hybrid exchange-correlation functional M06-2X.[12,13] Ahlrich's def2-SVP basis set[14] was utilized for geometry optimizations and vibrational mode calculations. Accurate energies were obtained by performing single-point calculations on the def2-SVP optimized structures utilizing the def2-TZVPP basis set.[15] Solvation effects in diethyl ether were incorporated using the implicit SMD solvation model[16] and included during structure optimization. Harmonic vibrational frequency calculations confirmed all the optimized structures as minima, and the converged wave functions were tested for SCF stability. The zero-point energy, thermal corrections, and entropic contributions were included in the calculation of the free energies. The standard states of 1 M concentration were considered for all the reactants and products to calculate the reactions' free energies. The electrostatic charges mentioned in the text are CHELP charges,[17] i.e. the charges are produced by fitting to the electrostatic potential at points selected according to the CHelp

scheme. A keyword TS was used that requests optimization to a transition state rather than a local minimum, using the Berny algorithm. All the transition state structures had only one imaginary frequency.

To maintain consistency across reactants, intermediates, and transition states, methanol or methanethiol was present in all complexes, forming hydrogen bonds with bromine. Special attention was given to the conformation of all structures, ensuring that the optimized geometries for both major and minor pathways for MeOH and MeSH reactions closely resembled each other. This approach allows small energy differences to be meaningful and ensures they are not affected by conformational changes in remote parts of the molecule.

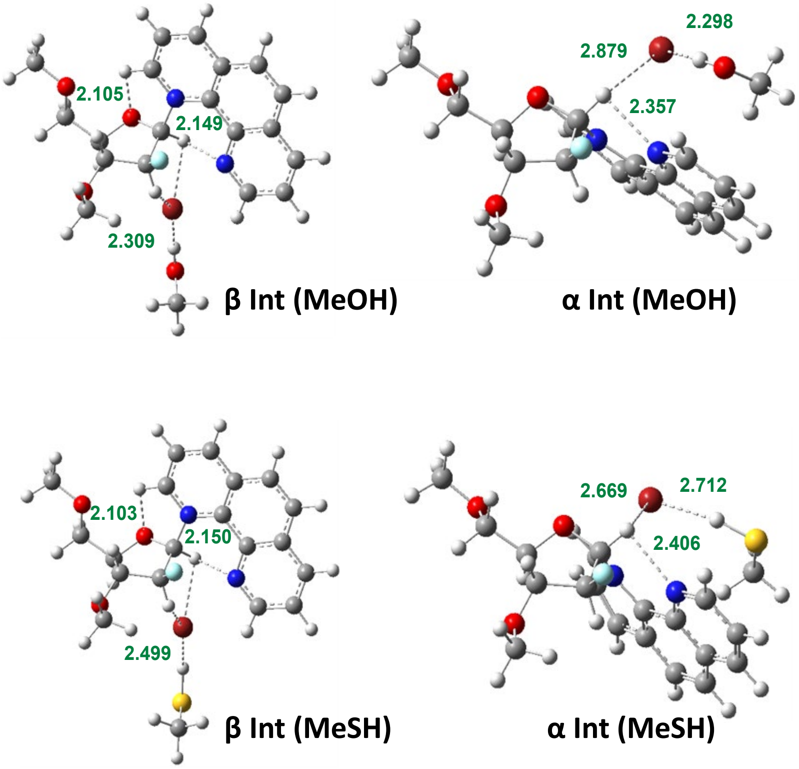

Figure S7. DFT calculated structures of the α- and β-Int. Hydrogen bonding distances are reported in Å (green).

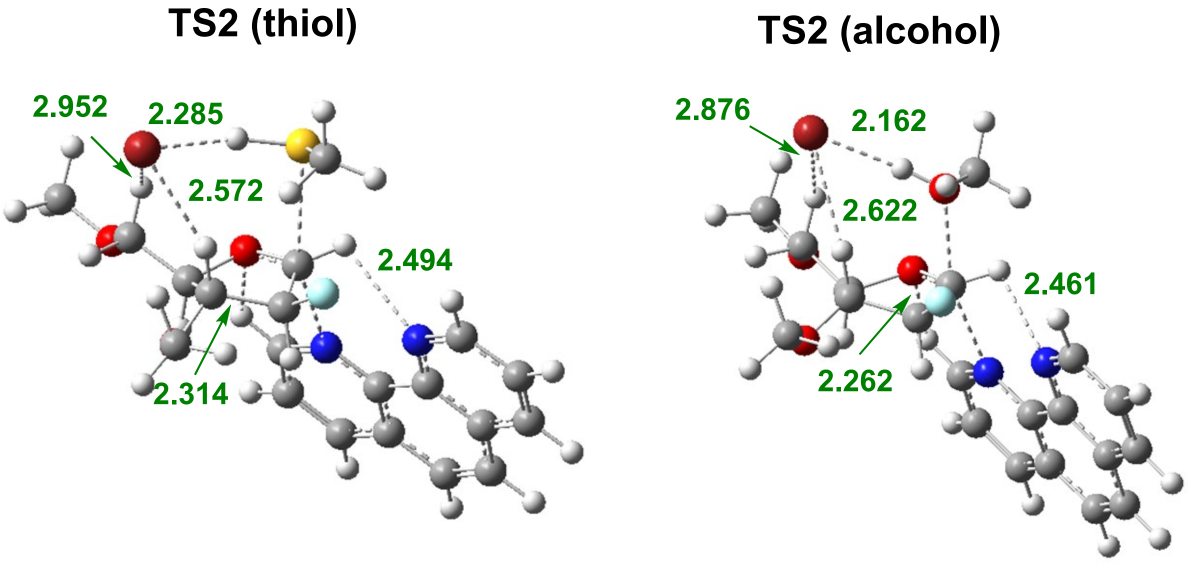

Figure S8. DFT calculated structures of the transition states TS2 with thiol and alcohol nucleophiles are presented. Hydrogen bonding distances are reported in Å (green).

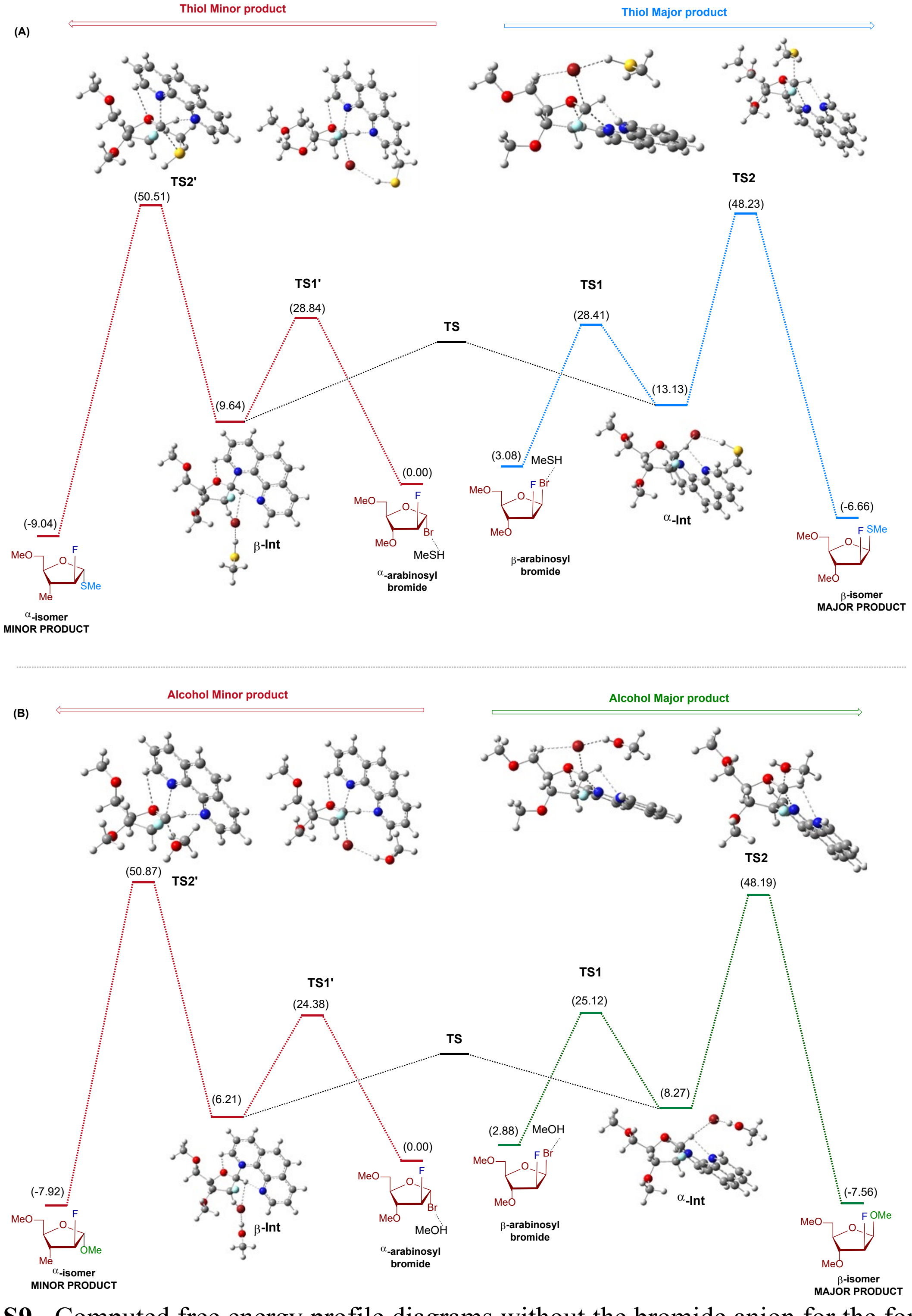

Figure S9. Computed free energy profile diagrams without the bromide anion for the formation of α/β-*S*-linked (**A**) furanoside products from arabinosyl bromide at 50 °C and (**B**) α/β-*O*-linked furanoside products from arabinosyl bromide at 50 °C. Relative free energy changes (ΔG) are in kcal/mol and are computed with the Gaussian 16[11] at the M06-2X/def2-TZVPP //M06-2X/def2-SVP level of theory[12-15] using diethyl ether with SMD implicit solvation.[16]

XYZ coordinates of DFT-calculated structures:

α-arabinosyl bromide (with MeOH)

Gibbs free energy: -2063995.20 kcal/mol

C	-1.710062000	-0.707974000	1.632646000
C	-0.644634000	0.278721000	2.091431000
C	0.070970000	0.629324000	0.788056000
C	-0.142945000	-0.639504000	-0.064249000
C	1.091343000	-1.513560000	-0.166030000
H	1.143405000	0.797445000	0.994592000
H	-0.492693000	-0.354976000	-1.070239000
H	0.832265000	-2.462446000	-0.677127000
H	1.450318000	-1.761281000	0.854820000
O	-1.168634000	-1.399136000	0.595475000
O	-0.510525000	1.766684000	0.219350000
O	2.048655000	-0.792703000	-0.881129000
Br	-3.351870000	0.317693000	1.087332000
H	-2.071148000	-1.387925000	2.412783000
H	-1.014739000	1.149518000	2.647834000
C	0.303920000	2.356334000	-0.772170000
H	0.590333000	1.626735000	-1.550400000
H	-0.278499000	3.165006000	-1.235912000
H	1.221617000	2.775713000	-0.317623000
C	3.258983000	-1.481752000	-1.030921000
H	3.943555000	-0.837154000	-1.602308000
H	3.717631000	-1.711415000	-0.048842000
H	3.117658000	-2.431979000	-1.582542000
F	0.233162000	-0.434337000	2.881797000
H	-2.050400000	1.214014000	-1.118748000
O	-2.067109000	1.055672000	-2.079807000
C	-2.981456000	0.032096000	-2.371726000
H	-2.929348000	-0.160724000	-3.455559000

H	-2.747508000	-0.912737000	-1.841986000
H	-4.024480000	0.311063000	-2.125008000

α-arabinosyl bromide (with MeSH)

Gibbs free energy: -2266662.62 kcal/mol

C	-1.746153000	-0.637706000	1.565262000
C	-0.636209000	0.254880000	2.103807000
C	0.125474000	0.651863000	0.840403000
C	-0.139249000	-0.543946000	-0.097824000
C	1.047043000	-1.479796000	-0.224731000
H	1.201784000	0.738411000	1.078326000
H	-0.440313000	-0.182726000	-1.095885000
H	0.751629000	-2.378366000	-0.802752000
H	1.361709000	-1.811605000	0.786864000
O	-1.225397000	-1.282021000	0.483367000
O	-0.374063000	1.858598000	0.347705000
O	2.063295000	-0.770475000	-0.866494000
Br	-3.327868000	0.495417000	1.090271000
H	-2.141028000	-1.355918000	2.293094000
H	-0.969324000	1.106815000	2.710563000
C	0.473954000	2.451219000	-0.607512000
H	0.670641000	1.775615000	-1.461000000
H	-0.027546000	3.359552000	-0.973960000
H	1.442568000	2.732201000	-0.150671000
C	3.238678000	-1.515313000	-1.026642000
H	3.976007000	-0.874362000	-1.532777000
H	3.651238000	-1.834585000	-0.049153000
H	3.064573000	-2.417892000	-1.644894000
F	0.185593000	-0.555963000	2.860147000

H	-2.099443000	1.561695000	-1.285063000
C	-3.129571000	-0.290750000	-2.395830000
H	-3.162035000	-0.768020000	-3.387430000
H	-2.610410000	-0.958652000	-1.691100000
H	-4.159058000	-0.118298000	-2.048651000
S	-2.228755000	1.276540000	-2.603649000

β-arabinosyl bromide (MeOH)

Gibbs free energy: -2063992.32 kcal/mol

C	0.620774000	-1.331918000	0.039909000
C	-0.580156000	-1.327524000	1.022090000
C	-1.561258000	-0.355835000	0.355820000
C	-0.613398000	0.550200000	-0.435199000
C	0.011900000	1.651112000	0.405321000
H	-2.140733000	0.187358000	1.126746000
H	-1.078939000	0.980905000	-1.333443000
H	0.413415000	1.235148000	1.350321000
H	-0.766614000	2.396068000	0.668323000
O	0.386331000	-0.359150000	-0.901248000
O	-2.386677000	-1.109629000	-0.480120000
O	1.031172000	2.225239000	-0.357312000
Br	2.338105000	-1.025268000	0.989086000
H	0.769167000	-2.297357000	-0.456850000
H	-1.018943000	-2.330001000	1.122967000
C	-3.505276000	-0.412683000	-0.965663000
H	-3.219524000	0.457273000	-1.586808000
H	-4.081363000	-1.112227000	-1.588810000
H	-4.145458000	-0.059655000	-0.134059000
C	1.900392000	3.048202000	0.375312000
H	2.671295000	3.411706000	-0.319771000
H	1.363317000	3.914251000	0.808802000

H	2.388717000	2.484267000	1.193504000
F	-0.249633000	-0.867979000	2.267059000
H	2.188604000	0.943644000	-1.338473000
O	3.011542000	0.842680000	-1.850127000
C	2.938057000	-0.324172000	-2.609584000
H	2.086478000	-0.326951000	-3.320114000
H	3.865931000	-0.404201000	-3.201795000
H	2.861799000	-1.241304000	-1.988920000

β-arabinosyl bromide (MeSH)

Gibbs free energy: -2266659.54 kcal/mol

C	0.629709000	-1.299092000	0.061612000
C	-0.587430000	-1.301086000	1.020940000
C	-1.557625000	-0.342975000	0.325026000
C	-0.591677000	0.595228000	-0.407114000
C	-0.028405000	1.699554000	0.469126000
H	-2.188613000	0.173658000	1.072829000
H	-1.031432000	1.036041000	-1.313888000
H	0.462467000	1.267175000	1.363093000
H	-0.861044000	2.343581000	0.820885000
O	0.438465000	-0.291345000	-0.850425000
O	-2.321265000	-1.103577000	-0.562351000
O	0.882728000	2.428975000	-0.297590000
Br	2.333139000	-1.073716000	1.062320000
H	0.765232000	-2.253337000	-0.461031000
H	-1.016252000	-2.306967000	1.129261000
C	-3.434017000	-0.427065000	-1.088639000
H	-3.142610000	0.463365000	-1.677072000
H	-3.959613000	-1.128439000	-1.753003000
H	-4.123053000	-0.108852000	-0.282417000

C	1.697471000	3.281516000	0.459640000
H	2.361109000	3.815323000	-0.237803000
H	1.095928000	4.025539000	1.018412000
H	2.315610000	2.711433000	1.180833000
F	-0.285364000	-0.816352000	2.264503000
H	2.311127000	1.145928000	-1.380606000
C	3.025733000	-0.749947000	-2.645911000
H	2.040737000	-0.813595000	-3.130982000
H	3.798815000	-1.055161000	-3.368337000
H	3.062903000	-1.429943000	-1.780672000
S	3.406249000	0.956330000	-2.153667000

α-Int$_1$

Gibbs free energy: -2625249.55 kcal/mol

C	0.904353000	-0.557627000	-0.118462000
C	0.806792000	-0.871074000	1.391820000
C	1.642560000	0.239178000	2.055816000
C	1.892469000	1.213661000	0.910808000
C	3.164800000	2.011771000	1.003637000
H	2.602759000	-0.223078000	2.365619000
H	1.036447000	1.913729000	0.849981000
H	4.030595000	1.327378000	0.886654000
H	3.219185000	2.468753000	2.013899000
O	1.932282000	0.368396000	-0.231623000
O	1.026973000	0.888335000	3.118811000
O	3.152922000	2.988142000	0.006914000
H	-0.226727000	-0.858404000	1.767225000
C	0.980101000	0.112091000	4.289370000
H	1.999064000	-0.140196000	4.641458000
H	0.468733000	0.706326000	5.060807000
H	0.419478000	-0.830068000	4.134922000

C	4.333929000	3.736835000	-0.039937000
H	4.226052000	4.485118000	-0.839498000
H	4.520418000	4.262010000	0.918444000
H	5.211379000	3.098496000	-0.265085000
N	-0.363195000	0.064870000	-0.632121000
C	-3.176632000	-2.363873000	-0.002889000
C	-3.342419000	-3.739678000	0.293962000
C	-2.238903000	-4.544863000	0.431756000
C	-0.960085000	-3.961513000	0.268390000
N	-0.778307000	-2.694906000	-0.006744000
H	-2.331144000	-5.609082000	0.657074000
C	-1.845766000	-1.881223000	-0.140821000
C	-2.751731000	0.316432000	-0.731204000
C	-4.286925000	-1.490972000	-0.189991000
C	-4.072149000	-0.192186000	-0.546390000
H	-5.298673000	-1.885132000	-0.067733000
H	-4.909954000	0.486195000	-0.725732000
C	-1.634104000	-0.495794000	-0.483591000
C	-0.215805000	1.131914000	-1.462089000
C	-2.554826000	1.652046000	-1.225983000
C	-1.324285000	2.034291000	-1.632670000
H	0.792239000	1.536106000	-1.503761000
H	-1.129777000	2.999167000	-2.103602000
H	-3.419840000	2.312595000	-1.324008000
H	-4.354550000	-4.138850000	0.404942000
H	-0.058408000	-4.575904000	0.372241000
F	1.364764000	-2.096545000	1.656263000
H	1.148934000	-1.441388000	-0.716529000
Br	0.025639000	0.189988000	-3.677445000
H	-0.687328000	-2.389565000	-3.234509000
C	-3.002650000	-2.144449000	-3.646555000

H	-3.946909000	-2.711420000	-3.635536000
H	-3.038469000	-1.368916000	-2.866525000
H	-2.879190000	-1.666224000	-4.629881000
S	-1.663697000	-3.328008000	-3.320038000

β-Int₂

Gibbs free energy: -2625253.04 kcal/mol

C	-0.161481000	0.164136000	-0.678189000
C	-0.729852000	-0.212253000	0.708678000
C	-1.793435000	0.853974000	0.941985000
C	-2.232607000	1.181829000	-0.479968000
C	-2.794237000	2.567813000	-0.672674000
H	-1.294318000	1.738518000	1.393381000
H	-2.965133000	0.421657000	-0.803291000
H	-3.728998000	2.652142000	-0.083471000
H	-3.046248000	2.719021000	-1.741039000
O	-1.044336000	1.049403000	-1.261248000
O	-2.890622000	0.464860000	1.699873000
O	-1.846805000	3.512308000	-0.249988000
H	-1.165335000	-1.220542000	0.648452000
C	-2.574356000	0.167369000	3.035096000
H	-2.062723000	1.018733000	3.526351000
H	-3.519930000	-0.034547000	3.559929000
H	-1.926955000	-0.725899000	3.115445000
C	-2.293034000	4.834917000	-0.382134000
H	-1.492455000	5.498207000	-0.021157000
H	-2.516678000	5.082546000	-1.438141000
H	-3.204211000	5.013381000	0.221134000
N	1.180999000	0.862973000	-0.487296000
C	3.690344000	-1.902148000	-0.038676000

C	3.721963000	-3.313946000	0.039511000
C	2.547242000	-4.029115000	-0.019496000
C	1.333761000	-3.316582000	-0.140188000
N	1.289291000	-2.006392000	-0.197931000
H	2.541581000	-5.119699000	0.030173000
C	2.419432000	-1.285719000	-0.174411000
C	3.559084000	0.905858000	-0.181263000
C	4.888273000	-1.118098000	0.027866000
C	4.820450000	0.234553000	-0.028432000
H	5.846839000	-1.631378000	0.134703000
H	5.719354000	0.851422000	0.034225000
C	2.354600000	0.166453000	-0.283296000
C	1.157512000	2.197367000	-0.515319000
C	3.518434000	2.314482000	-0.230886000
C	2.314513000	2.962863000	-0.379626000
H	0.175285000	2.655043000	-0.661469000
H	2.237301000	4.049899000	-0.410248000
H	4.454089000	2.872985000	-0.144474000
H	4.686874000	-3.816967000	0.144643000
H	0.364988000	-3.826956000	-0.203161000
F	0.240549000	-0.171311000	1.686785000
Br	-2.093004000	-2.511766000	-1.489693000
H	-0.002067000	-0.704289000	-1.320433000
H	-3.627130000	-3.946374000	-2.844469000
C	-4.511821000	-5.357420000	-1.190462000
H	-5.213019000	-6.190753000	-1.026672000
H	-3.504250000	-5.659029000	-0.868016000
H	-4.830602000	-4.488789000	-0.595610000
S	-4.529406000	-4.962849000	-2.964711000

α-Int$_3$

Gibbs free energy: -2422585.91 kcal/mol

C	0.531073000	-0.349091000	-0.485860000
C	0.663074000	-0.979961000	0.920035000
C	1.739114000	-0.126555000	1.618192000
C	1.908330000	1.056626000	0.675549000
C	3.276318000	1.682605000	0.644134000
H	2.668697000	-0.731993000	1.631476000
H	1.171781000	1.834317000	0.957459000
H	3.993887000	0.963074000	0.198822000
H	3.593521000	1.887626000	1.687944000
O	1.601905000	0.511547000	-0.605542000
O	1.405026000	0.319350000	2.891072000
O	3.205691000	2.858052000	-0.104479000
H	-0.279065000	-0.967468000	1.487590000
C	1.455957000	-0.694395000	3.864203000
H	2.477612000	-1.111333000	3.952086000
H	1.165413000	-0.245039000	4.825074000
H	0.758208000	-1.521738000	3.631069000
C	4.450799000	3.475023000	-0.274497000
H	4.293460000	4.392767000	-0.860684000
H	4.905460000	3.745998000	0.699430000
H	5.157364000	2.819248000	-0.820634000
N	-0.767124000	0.458293000	-0.640176000
C	-3.618473000	-1.960067000	-0.246180000
C	-3.825372000	-3.354028000	-0.136851000
C	-2.750576000	-4.209760000	-0.209288000
C	-1.464924000	-3.654571000	-0.383095000
N	-1.251868000	-2.362821000	-0.462299000
H	-2.877052000	-5.292045000	-0.142239000
C	-2.282024000	-1.509512000	-0.398856000
C	-3.146657000	0.783409000	-0.632430000

C	-4.713247000	-1.035847000	-0.253774000
C	-4.482657000	0.283970000	-0.457077000
H	-5.728043000	-1.420774000	-0.127478000
H	-5.303207000	1.002904000	-0.508815000
C	-2.031198000	-0.084821000	-0.542111000
C	-0.610208000	1.728697000	-1.018020000
C	-2.948813000	2.143586000	-0.939470000
C	-1.677947000	2.607985000	-1.177965000
H	0.415449000	2.054916000	-1.182090000
H	-1.474865000	3.637694000	-1.472315000
H	-3.817149000	2.802783000	-1.016396000
H	-4.844523000	-3.730864000	-0.014743000
H	-0.585859000	-4.302310000	-0.454290000
F	1.109844000	-2.270824000	0.814482000
H	0.526389000	-1.069261000	-1.311146000
Br	-0.920702000	-0.448724000	-3.722447000
H	-0.975473000	-2.720494000	-3.375087000
O	-1.308439000	-3.646514000	-3.308127000
C	-2.669256000	-3.589161000	-3.607081000
H	-3.115580000	-4.580041000	-3.407063000
H	-3.210729000	-2.835922000	-2.998354000
H	-2.861537000	-3.341368000	-4.672030000

β-Int$_4$

Gibbs free energy: -2422588.09 kcal/mol

C	-0.197327000	0.157296000	-0.654127000
C	-0.736339000	-0.192196000	0.751591000
C	-1.784240000	0.887969000	0.992273000
C	-2.256241000	1.193840000	-0.424140000
C	-2.812449000	2.580443000	-0.628075000
H	-1.266296000	1.775889000	1.414455000

H	-3.003391000	0.434989000	-0.715602000
H	-3.731211000	2.682544000	-0.016897000
H	-3.090479000	2.713956000	-1.692315000
O	-1.088686000	1.037198000	-1.232294000
O	-2.864885000	0.522928000	1.784846000
O	-1.847666000	3.524861000	-0.246956000
H	-1.181975000	-1.197192000	0.720230000
C	-2.518068000	0.250705000	3.118121000
H	-1.988732000	1.108282000	3.578781000
H	-3.452022000	0.066905000	3.669647000
H	-1.875296000	-0.645590000	3.201382000
C	-2.287168000	4.848525000	-0.391698000
H	-1.472428000	5.511628000	-0.063642000
H	-2.536081000	5.078792000	-1.445942000
H	-3.180964000	5.045030000	0.231574000
N	1.152696000	0.850555000	-0.504319000
C	3.656057000	-1.922554000	-0.071025000
C	3.682618000	-3.333014000	0.029585000
C	2.503325000	-4.042650000	0.010735000
C	1.291079000	-3.325487000	-0.092899000
N	1.251283000	-2.016207000	-0.172428000
H	2.493019000	-5.132151000	0.079169000
C	2.385530000	-1.301229000	-0.187278000
C	3.537097000	0.883361000	-0.253762000
C	4.859523000	-1.144692000	-0.044440000
C	4.797995000	0.207299000	-0.119702000
H	5.817195000	-1.662340000	0.048472000
H	5.701320000	0.819995000	-0.086856000
C	2.326525000	0.149585000	-0.316958000
C	1.135965000	2.184597000	-0.550977000
C	3.503329000	2.291346000	-0.323234000

C	2.300054000	2.944958000	-0.453316000
H	0.153365000	2.646252000	-0.681553000
H	2.228398000	4.031882000	-0.498218000
H	4.443894000	2.845431000	-0.267323000
H	4.647321000	-3.839269000	0.120028000
H	0.319258000	-3.832848000	-0.122748000
F	0.256960000	-0.145234000	1.705457000
Br	-2.156617000	-2.524875000	-1.385385000
H	-0.055110000	-0.721682000	-1.285719000
H	-3.639193000	-3.943693000	-2.444810000
O	-4.265394000	-4.679468000	-2.624636000
C	-4.387551000	-5.390825000	-1.432566000
H	-5.097527000	-6.221171000	-1.594030000
H	-3.426865000	-5.829828000	-1.091526000
H	-4.775895000	-4.770950000	-0.598260000

TS1 (alcohol)

Gibbs free energy: -2422570.14 kcal/mol

C	-0.711261000	-0.078524000	-0.973433000
C	-0.923153000	0.512293000	0.407708000
C	-2.275909000	-0.066234000	0.828443000
C	-2.441406000	-1.289028000	-0.100631000
C	-3.793422000	-1.451101000	-0.769715000
H	-3.059449000	0.684085000	0.612061000
H	-2.150277000	-2.204541000	0.441128000
H	-3.744938000	-2.292217000	-1.491294000
H	-4.017971000	-0.522184000	-1.330895000
O	-1.490645000	-1.082450000	-1.181869000
O	-2.212696000	-0.380543000	2.182724000

O	-4.733125000	-1.697252000	0.231987000
Br	-2.551609000	1.787814000	-2.181618000
H	-0.176884000	0.364448000	-1.805294000
H	-0.149880000	0.111049000	1.081824000
C	-3.453558000	-0.585648000	2.815512000
H	-3.933959000	-1.521837000	2.484008000
H	-3.255425000	-0.634024000	3.896918000
H	-4.145899000	0.253216000	2.612251000
C	-6.048411000	-1.745648000	-0.252499000
H	-6.713095000	-1.932356000	0.604220000
H	-6.335997000	-0.789967000	-0.731520000
H	-6.175964000	-2.562004000	-0.990404000
N	1.102035000	-1.264925000	-0.621105000
C	3.988128000	1.028333000	-0.207659000
C	4.214019000	2.423388000	-0.179243000
C	3.150041000	3.284269000	-0.337441000
C	1.858958000	2.743513000	-0.529995000
N	1.632363000	1.446206000	-0.570636000
H	3.287623000	4.367161000	-0.314880000
C	2.656430000	0.593304000	-0.407960000
C	3.442567000	-1.747039000	-0.274445000
C	5.045414000	0.068601000	-0.043263000
C	4.783056000	-1.263584000	-0.077004000
H	6.063795000	0.436026000	0.107411000
H	5.582801000	-1.998247000	0.044674000
C	2.373261000	-0.832117000	-0.436601000
C	0.855514000	-2.557458000	-0.670623000
C	3.148914000	-3.127435000	-0.315915000
C	1.850887000	-3.539832000	-0.518086000

H	-0.183710000	-2.851619000	-0.849671000
H	1.582686000	-4.596459000	-0.564251000
H	3.959255000	-3.850866000	-0.191255000
H	5.230339000	2.798266000	-0.028673000
H	0.981677000	3.395986000	-0.644140000
F	-0.886267000	1.863686000	0.473732000
H	-1.396900000	3.541014000	-1.073579000
C	-0.772335000	4.867403000	-2.329439000
H	-0.264854000	5.846363000	-2.286541000
H	-0.164931000	4.191299000	-2.964692000
H	-1.747455000	5.007462000	-2.835146000
O	-0.917146000	4.396147000	-1.019727000

TS1' (alcohol)

Gibbs free energy: -2422570.87 kcal/mol

C	0.921560000	0.820158000	-0.294818000
C	1.121069000	0.364892000	1.152833000
C	2.273025000	-0.635091000	0.994102000
C	2.954005000	-0.146783000	-0.281134000
C	3.748055000	-1.151529000	-1.068906000
H	1.827852000	-1.637271000	0.822041000
H	3.586508000	0.724284000	-0.038073000
H	4.649538000	-1.396279000	-0.472010000
H	4.086009000	-0.686035000	-2.016301000
O	1.850982000	0.376144000	-1.066676000
O	3.196654000	-0.659424000	2.026898000
O	2.978692000	-2.291139000	-1.310527000
Br	2.085998000	3.276098000	0.125946000
H	0.140099000	1.458030000	-0.685736000

H	1.426569000	1.225762000	1.763005000
C	2.681033000	-1.183896000	3.227196000
H	2.280167000	-2.204705000	3.076068000
H	3.509825000	-1.223540000	3.948787000
H	1.878187000	-0.545283000	3.640402000
C	3.667085000	-3.278776000	-2.032376000
H	2.983702000	-4.130491000	-2.166889000
H	3.980244000	-2.906650000	-3.026909000
H	4.565404000	-3.621669000	-1.483683000
N	-0.713909000	-0.755865000	-0.779761000
C	-3.933552000	0.398421000	0.649857000
C	-4.444908000	1.544676000	1.300649000
C	-3.625912000	2.634193000	1.493325000
C	-2.297213000	2.573482000	1.017287000
N	-1.800470000	1.514529000	0.411504000
H	-3.983047000	3.536648000	1.993439000
C	-2.580152000	0.430751000	0.234235000
C	-2.854771000	-1.859282000	-0.665672000
C	-4.744635000	-0.761648000	0.398475000
C	-4.229411000	-1.841929000	-0.242738000
H	-5.786599000	-0.748349000	0.728559000
H	-4.844204000	-2.722224000	-0.446807000
C	-2.017229000	-0.743824000	-0.418886000
C	-0.213048000	-1.802487000	-1.398923000
C	-2.296007000	-2.975406000	-1.328924000
C	-0.972947000	-2.949377000	-1.706829000
H	0.852565000	-1.761377000	-1.655189000
H	-0.504982000	-3.790042000	-2.222368000
H	-2.926073000	-3.846026000	-1.531683000
H	-5.484692000	1.548170000	1.639600000
H	-1.628446000	3.436511000	1.137458000

F	0.021027000	-0.207253000	1.723725000
H	0.394730000	4.680831000	0.932769000
O	-0.392294000	5.265767000	0.954115000
C	-0.725221000	5.543944000	-0.377901000
H	0.095702000	6.056126000	-0.917083000
H	-0.986143000	4.632474000	-0.953527000
H	-1.602777000	6.212090000	-0.378953000

TS1 (thiol)

Gibbs free energy: -2625234.27 kcal/mol

C	-0.619507000	-0.119016000	-0.990750000
C	-0.822422000	0.469881000	0.393442000
C	-2.188449000	-0.080644000	0.807908000
C	-2.374901000	-1.298477000	-0.123480000
C	-3.730023000	-1.437277000	-0.791608000
H	-2.954860000	0.686064000	0.588201000
H	-2.099039000	-2.219488000	0.417449000
H	-3.695114000	-2.277457000	-1.515182000
H	-3.938820000	-0.503196000	-1.350258000
O	-1.422507000	-1.105412000	-1.204517000
O	-2.136911000	-0.397051000	2.162603000
O	-4.673478000	-1.671766000	0.209943000
Br	-2.447465000	1.773954000	-2.196118000
H	-0.089341000	0.327481000	-1.823445000
H	-0.061566000	0.048878000	1.069882000
C	-3.383848000	-0.573262000	2.791898000
H	-3.883139000	-1.500080000	2.462147000
H	-3.190447000	-0.621889000	3.874207000
H	-4.057451000	0.279502000	2.583425000
C	-5.989727000	-1.691195000	-0.273310000

H	-6.657438000	-1.868868000	0.583051000
H	-6.258608000	-0.727591000	-0.747301000
H	-6.134624000	-2.501016000	-1.015291000
N	1.152089000	-1.329636000	-0.637863000
C	4.078851000	0.898421000	-0.147758000
C	4.340669000	2.287238000	-0.128431000
C	3.307340000	3.173421000	-0.340458000
C	2.012821000	2.660596000	-0.575836000
N	1.750187000	1.371273000	-0.605034000
H	3.470714000	4.252828000	-0.329039000
C	2.744354000	0.494382000	-0.391480000
C	3.467879000	-1.862480000	-0.211480000
C	5.105510000	-0.084701000	0.064202000
C	4.811813000	-1.410185000	0.030714000
H	6.126466000	0.259457000	0.248505000
H	5.588475000	-2.163044000	0.185277000
C	2.426609000	-0.923834000	-0.415384000
C	0.878720000	-2.616753000	-0.692020000
C	3.145198000	-3.236168000	-0.258478000
C	1.846432000	-3.620321000	-0.505243000
H	-0.160649000	-2.887754000	-0.902474000
H	1.556136000	-4.670708000	-0.558738000
H	3.934651000	-3.976482000	-0.103271000
H	5.360224000	2.637328000	0.056011000
H	1.166192000	3.340432000	-0.737104000
F	-0.755678000	1.819825000	0.468670000
H	-1.524029000	3.698959000	-0.791876000
S	-1.103646000	4.993209000	-0.761670000
C	-1.440931000	5.305759000	-2.520179000
H	-1.088135000	6.323606000	-2.748562000
H	-0.908384000	4.580399000	-3.151917000

H	-2.518963000	5.239052000	-2.728519000

TS1’ (thiol)

Gibbs free energy: -2625233.84 kcal/mol

C	0.888103000	0.849110000	-0.076577000
C	0.942193000	0.413410000	1.386426000
C	2.076341000	-0.612828000	1.370108000
C	2.889769000	-0.177911000	0.139068000
C	3.666187000	-1.222790000	-0.633123000
H	1.625597000	-1.609425000	1.180864000
H	3.551139000	0.658465000	0.423499000
H	3.949266000	-0.796025000	-1.616590000
H	3.037255000	-2.119106000	-0.818547000
O	1.887718000	0.380405000	-0.746218000
O	2.767371000	-0.587653000	2.571249000
O	4.794455000	-1.551468000	0.116038000
Br	2.060045000	3.296076000	0.434664000
H	0.193061000	1.531132000	-0.545437000
H	1.215261000	1.274476000	2.010024000
C	3.319829000	-1.821112000	2.969680000
H	4.056256000	-2.190128000	2.236105000
H	3.822392000	-1.650076000	3.933139000
H	2.526682000	-2.580339000	3.113030000
C	5.609868000	-2.505534000	-0.509475000
H	6.470825000	-2.691771000	0.149262000
H	5.067600000	-3.457961000	-0.671716000
H	5.978292000	-2.138476000	-1.487140000
N	-0.676019000	-0.666347000	-0.689669000
C	-3.985874000	0.520857000	0.487940000
C	-4.537528000	1.675443000	1.089700000

C	-3.727600000	2.760276000	1.339122000
C	-2.366946000	2.683689000	0.968744000
N	-1.832790000	1.620046000	0.407607000
H	-4.113476000	3.669714000	1.803914000
C	-2.604832000	0.541520000	0.176959000
C	-2.827611000	-1.749830000	-0.734478000
C	-4.783450000	-0.635532000	0.182330000
C	-4.229495000	-1.722077000	-0.414249000
H	-5.847187000	-0.614133000	0.432869000
H	-4.833563000	-2.598792000	-0.660492000
C	-2.002792000	-0.639818000	-0.426039000
C	-0.145033000	-1.712907000	-1.280027000
C	-2.231976000	-2.869163000	-1.358008000
C	-0.885925000	-2.852620000	-1.646851000
H	0.931736000	-1.664013000	-1.481692000
H	-0.391338000	-3.692872000	-2.136920000
H	-2.852665000	-3.734595000	-1.606176000
H	-5.600623000	1.688730000	1.346015000
H	-1.703778000	3.541315000	1.141187000
F	-0.211583000	-0.129324000	1.870626000
H	0.478469000	5.146299000	1.228426000
S	-0.528326000	6.025973000	0.976109000
C	-0.638873000	5.618862000	-0.792295000
H	-0.902311000	4.560175000	-0.940533000
H	-1.427699000	6.253240000	-1.225541000
H	0.314326000	5.828452000	-1.299220000

TS2 (alcohol)

Gibbs free energy: -2422569.74 kcal/mol

C	0.131763000	0.014733000	-0.667271000
C	0.253113000	-0.646660000	0.686100000

C	1.558661000	-0.028051000	1.198064000
C	1.518420000	1.340922000	0.522638000
C	2.832254000	2.011222000	0.244496000
H	2.413656000	-0.615911000	0.807071000
H	0.863271000	2.019350000	1.095603000
H	3.404643000	1.422076000	-0.499396000
H	3.417226000	2.004477000	1.187887000
O	0.838874000	1.068845000	-0.748649000
O	1.631658000	0.140091000	2.572994000
O	2.563769000	3.312324000	-0.190585000
H	-0.377521000	-0.365317000	-1.547432000
H	-0.587087000	-0.325920000	1.322720000
C	2.084888000	-1.015255000	3.245524000
H	3.072937000	-1.328667000	2.861268000
H	2.169067000	-0.759848000	4.311933000
H	1.373634000	-1.854382000	3.131308000
C	3.720322000	4.004905000	-0.573013000
H	3.416860000	5.014363000	-0.889191000
H	4.435701000	4.092761000	0.268481000
H	4.233531000	3.502467000	-1.415938000
N	-1.847226000	1.223323000	-0.375755000
C	-4.703559000	-1.100530000	0.022592000
C	-4.917892000	-2.496699000	0.058850000
C	-3.845965000	-3.349381000	-0.088165000
C	-2.563847000	-2.791201000	-0.275770000
N	-2.339384000	-1.494966000	-0.321548000
H	-3.971823000	-4.433421000	-0.062280000
C	-3.375121000	-0.649458000	-0.169168000
C	-4.198972000	1.681388000	-0.068338000
C	-5.776509000	-0.154409000	0.168473000
C	-5.534175000	1.180893000	0.121558000

H	-6.790283000	-0.536338000	0.313377000
H	-6.346660000	1.904244000	0.226452000
C	-3.113947000	0.781694000	-0.205348000
C	-1.616047000	2.518579000	-0.442834000
C	-3.922660000	3.065234000	-0.128310000
C	-2.627532000	3.490866000	-0.322162000
H	-0.576740000	2.827381000	-0.605178000
H	-2.372785000	4.550226000	-0.382729000
H	-4.743521000	3.780117000	-0.024413000
H	-5.931780000	-2.879891000	0.203945000
H	-1.691106000	-3.443479000	-0.388801000
O	1.777020000	-1.340127000	-1.583610000
C	1.678834000	-2.733178000	-1.743764000
H	2.302761000	-3.081160000	-2.588533000
H	1.989788000	-3.277850000	-0.834731000
H	0.628352000	-2.984849000	-1.966398000
F	0.270095000	-2.003453000	0.622270000
H	2.717318000	-1.144996000	-1.299582000
Br	4.681235000	-1.153017000	-0.395739000

TS2' (alcohol)

Gibbs free energy: -2422568.16 kcal/mol

C	-0.448385000	-0.167977000	-0.866764000
C	-0.710504000	-0.362295000	0.615312000
C	-1.429940000	0.948746000	0.955341000
C	-2.167915000	1.200849000	-0.356431000
C	-2.624165000	2.600392000	-0.649784000
H	-0.657497000	1.728309000	1.127310000
H	-3.021719000	0.498755000	-0.404285000
H	-3.459229000	2.819186000	0.045240000
H	-3.025138000	2.645247000	-1.682491000

O	-1.194094000	0.747117000	-1.357944000
O	-2.348380000	0.913433000	1.985001000
O	-1.572153000	3.501548000	-0.475172000
H	0.246148000	-0.716340000	-1.490539000
H	-1.414162000	-1.205543000	0.750385000
C	-1.831246000	0.444210000	3.204667000
H	-0.921136000	1.002701000	3.499145000
H	-2.610221000	0.594458000	3.966197000
H	-1.592592000	-0.634101000	3.154150000
C	-1.945362000	4.835999000	-0.699732000
H	-1.056741000	5.463094000	-0.533177000
H	-2.303288000	4.985526000	-1.736692000
H	-2.743793000	5.150526000	-0.000574000
N	1.575326000	1.004049000	-0.562199000
C	4.247975000	-1.443979000	0.215046000
C	4.422488000	-2.846785000	0.206969000
C	3.395673000	-3.654604000	-0.227934000
C	2.202598000	-3.042454000	-0.667996000
N	2.015652000	-1.740612000	-0.666748000
H	3.492205000	-4.741824000	-0.246540000
C	2.999932000	-0.936585000	-0.220828000
C	3.845962000	1.354170000	0.175602000
C	5.292217000	-0.546794000	0.629920000
C	5.103178000	0.797340000	0.598268000
H	6.243832000	-0.972680000	0.958169000
H	5.898708000	1.484645000	0.897110000
C	2.780292000	0.503327000	-0.206372000
C	1.398587000	2.307690000	-0.610583000
C	3.629876000	2.750084000	0.128259000
C	2.408194000	3.234527000	-0.280544000
H	0.401258000	2.661130000	-0.899820000

H	2.203452000	4.305207000	-0.337665000
H	4.440158000	3.425655000	0.416171000
H	5.372880000	-3.269604000	0.544648000
H	1.372872000	-3.655748000	-1.037167000
O	-1.700073000	-1.928765000	-1.573843000
C	-2.337324000	-1.585842000	-2.777633000
H	-3.066047000	-0.762553000	-2.646565000
H	-1.570175000	-1.265749000	-3.502606000
H	-2.875588000	-2.452257000	-3.205138000
F	0.410465000	-0.575859000	1.357232000
H	-2.421404000	-2.121373000	-0.900009000
Br	-4.018257000	-1.966708000	0.458265000

TS2 (thiol)

Gibbs free energy: -2625233.50 kcal/mol

C	-0.000333000	0.044174000	-0.704212000
C	0.181786000	-0.599083000	0.647191000
C	1.480876000	0.066694000	1.115749000
C	1.361919000	1.433317000	0.442589000
C	2.633333000	2.161767000	0.117963000
H	2.350965000	-0.480298000	0.699178000
H	0.695328000	2.082850000	1.035494000
H	3.209521000	1.601097000	-0.644312000
H	3.254168000	2.180211000	1.037200000
O	0.645903000	1.133506000	-0.806423000
O	1.596771000	0.241099000	2.485266000
O	2.288381000	3.446861000	-0.309354000
H	-0.559388000	-0.338521000	-1.554321000
H	-0.653616000	-0.288177000	1.296775000
C	2.171995000	-0.874116000	3.137209000
H	3.157343000	-1.113252000	2.696730000

H	2.293010000	-0.602726000	4.196228000
H	1.515408000	-1.761841000	3.067992000
C	3.399316000	4.200034000	-0.713065000
H	3.036826000	5.192570000	-1.020331000
H	4.126142000	4.323076000	0.113793000
H	3.920527000	3.726534000	-1.567865000
N	-2.066254000	1.236283000	-0.340197000
C	-4.827400000	-1.186426000	0.104281000
C	-4.991085000	-2.589408000	0.138474000
C	-3.891924000	-3.402596000	-0.030460000
C	-2.633893000	-2.797957000	-0.236011000
N	-2.455728000	-1.494316000	-0.277830000
H	-3.978224000	-4.490583000	-0.008329000
C	-3.519517000	-0.687034000	-0.106955000
C	-4.426358000	1.613122000	0.012911000
C	-5.931391000	-0.279739000	0.270699000
C	-5.739134000	1.063899000	0.224368000
H	-6.928116000	-0.698732000	0.430844000
H	-6.575884000	1.756489000	0.345406000
C	-3.312539000	0.753286000	-0.145583000
C	-1.879740000	2.538380000	-0.406728000
C	-4.197983000	3.005830000	-0.048455000
C	-2.921416000	3.475944000	-0.263727000
H	-0.854274000	2.882716000	-0.588207000
H	-2.704692000	4.543762000	-0.325090000
H	-5.040244000	3.692805000	0.071364000
H	-5.988165000	-3.009183000	0.298496000
H	-1.740934000	-3.418421000	-0.368744000
C	2.213623000	-3.163031000	-1.166988000
H	3.018404000	-3.719867000	-1.669809000
H	2.488718000	-2.994058000	-0.115992000

H	1.276929000	-3.736345000	-1.227028000
F	0.224968000	-1.955859000	0.595767000
Br	4.821621000	-0.772457000	0.047735000
S	1.963336000	-1.567432000	-1.998893000
H	3.107478000	-1.005215000	-1.444533000

TS2' (thiol)

Gibbs free energy: -2625231.17 kcal/mol

C	-0.412735000	-0.090353000	-0.856280000
C	-0.676036000	-0.215823000	0.625152000
C	-1.295576000	1.157348000	0.923570000
C	-2.052018000	1.387255000	-0.383448000
C	-2.428052000	2.794367000	-0.745520000
H	-0.468638000	1.889646000	1.035522000
H	-2.946290000	0.737198000	-0.374166000
H	-3.226410000	3.105653000	-0.042655000
H	-2.855957000	2.806900000	-1.768517000
O	-1.128580000	0.826291000	-1.382460000
O	-2.190392000	1.240387000	1.970000000
O	-1.313969000	3.629026000	-0.650624000
H	0.301154000	-0.652745000	-1.449063000
H	-1.452014000	-0.991987000	0.797875000
C	-1.737656000	0.679238000	3.177389000
H	-0.754391000	1.094813000	3.472491000
H	-2.482040000	0.929301000	3.947228000
H	-1.667195000	-0.421242000	3.104323000
C	-1.601769000	4.970957000	-0.947143000
H	-0.667491000	5.542568000	-0.842631000
H	-1.979061000	5.082881000	-1.981891000
H	-2.355349000	5.381767000	-0.248250000
N	1.750043000	1.027146000	-0.584740000

C	4.251136000	-1.571043000	0.259037000
C	4.351944000	-2.980623000	0.237213000
C	3.301203000	-3.726621000	-0.247463000
C	2.159212000	-3.046093000	-0.720099000
N	2.038725000	-1.735886000	-0.705612000
H	3.340377000	-4.817101000	-0.279884000
C	3.049117000	-0.990963000	-0.214426000
C	4.006175000	1.245149000	0.245115000
C	5.326625000	-0.738189000	0.725714000
C	5.213671000	0.614611000	0.707110000
H	6.239679000	-1.221333000	1.083086000
H	6.033878000	1.252679000	1.045961000
C	2.910368000	0.459669000	-0.189010000
C	1.646030000	2.339449000	-0.620804000
C	3.867887000	2.651171000	0.208926000
C	2.690598000	3.206316000	-0.239337000
H	0.680874000	2.749317000	-0.944009000
H	2.546979000	4.287285000	-0.289183000
H	4.701897000	3.278138000	0.536760000
H	5.265223000	-3.458061000	0.603250000
H	1.315936000	-3.613658000	-1.129434000
C	-3.116021000	-1.229892000	-2.669168000
H	-3.956591000	-1.091435000	-1.969058000
H	-2.679101000	-0.252104000	-2.929523000
H	-3.474270000	-1.719365000	-3.587316000
F	0.436598000	-0.496769000	1.355551000
H	-2.598335000	-2.356129000	-0.649360000
Br	-3.925595000	-1.677639000	0.964140000
S	-1.880832000	-2.279646000	-1.844975000

Arabinose product Alpha (alcohol)

Gibbs free energy: -448286.26 kcal/mol

C	-1.312034000	0.363161000	0.113023000
C	-0.177696000	1.360010000	0.286180000
C	1.051967000	0.562782000	-0.128874000
C	0.471840000	-0.486850000	-1.097073000
C	0.903636000	-1.899435000	-0.762883000
H	1.454612000	0.073612000	0.780707000
H	0.795266000	-0.239139000	-2.120864000
H	2.001710000	-1.988671000	-0.903107000
H	0.410128000	-2.605525000	-1.460856000
O	-0.943343000	-0.374139000	-1.029568000
O	2.047718000	1.298515000	-0.773954000
O	0.539885000	-2.176146000	0.559103000
H	-1.369766000	-0.308331000	0.997133000
H	-0.339318000	2.212261000	-0.395305000
C	2.687523000	2.216917000	0.073024000
H	3.129904000	1.710483000	0.953632000
H	3.490565000	2.697575000	-0.505686000
H	1.993060000	2.999409000	0.434144000
C	0.858901000	-3.478193000	0.960727000
H	0.543081000	-3.594439000	2.008523000
H	0.333257000	-4.233271000	0.343110000
H	1.948584000	-3.668518000	0.892790000
O	-2.506500000	1.008446000	-0.084834000
C	-3.615810000	0.141626000	-0.133808000
H	-4.517313000	0.766546000	-0.212592000
H	-3.561918000	-0.529678000	-1.008372000
H	-3.680073000	-0.470691000	0.786137000
F	-0.104870000	1.827206000	1.575859000

Arabinose product beta (alcohol)

Gibbs free energy: -448285.89 kcal/mol

C	-0.074538000	1.586482000	0.594881000
C	-1.418261000	0.948780000	0.251330000
C	-1.011446000	-0.508645000	0.057955000
C	0.082287000	-0.669513000	1.121388000
C	1.247226000	-1.539476000	0.703590000
H	-0.583495000	-0.604464000	-0.958913000
H	-0.379391000	-1.116414000	2.018593000
H	0.880396000	-2.578496000	0.564543000
H	1.996030000	-1.551809000	1.522233000
O	0.519880000	0.648997000	1.448310000
O	-2.014405000	-1.453156000	0.275769000
O	1.808742000	-1.042274000	-0.473595000
H	-0.139645000	2.537984000	1.155445000
H	-2.117927000	1.037983000	1.099430000
C	-3.012603000	-1.448503000	-0.712496000
H	-2.576767000	-1.566544000	-1.723835000
H	-3.681512000	-2.298432000	-0.509307000
H	-3.607575000	-0.516241000	-0.695521000
C	2.935707000	-1.757064000	-0.890835000
H	3.301140000	-1.295897000	-1.821082000
H	3.744017000	-1.721564000	-0.133007000
H	2.693356000	-2.820139000	-1.091251000
O	0.611342000	1.762732000	-0.592966000
C	1.942183000	2.180243000	-0.421726000
H	2.355790000	2.367522000	-1.423880000
H	1.991970000	3.117187000	0.168359000
H	2.540832000	1.401610000	0.078737000
F	-1.996114000	1.502612000	-0.859468000

Arabinose product Alpha (thiol)

Gibbs free energy: -650954.80 kcal/mol

C	1.058294000	-0.201297000	0.091238000
C	0.038637000	-1.320164000	0.275336000
C	-1.267169000	-0.635470000	-0.123867000
C	-0.802324000	0.458974000	-1.102078000
C	-1.339985000	1.831987000	-0.757706000
H	-1.693368000	-0.184168000	0.794658000
H	-1.123728000	0.185694000	-2.119560000
H	-2.444560000	1.831057000	-0.873941000
H	-0.921717000	2.574021000	-1.467441000
O	0.623215000	0.460975000	-1.070047000
O	-2.201603000	-1.461773000	-0.748529000
O	-0.971888000	2.142477000	0.555645000
H	1.019421000	0.471620000	0.968348000
H	0.246454000	-2.163107000	-0.407320000
C	-2.747291000	-2.426811000	0.113316000
H	-3.217438000	-1.954237000	0.998178000
H	-3.515245000	-2.977254000	-0.450518000
H	-1.983325000	-3.145510000	0.466466000
C	-1.397694000	3.411752000	0.964039000
H	-1.068237000	3.559244000	2.003627000
H	-0.957974000	4.209297000	0.332934000
H	-2.501442000	3.501856000	0.922711000
C	3.619921000	0.750734000	-0.042526000
H	4.693099000	0.527856000	-0.144993000
H	3.303009000	1.392490000	-0.877979000
H	3.450140000	1.265738000	0.916420000
F	0.024050000	-1.787496000	1.564814000
S	2.737067000	-0.830774000	-0.087744000

Arabinose product beta (thiol)

Gibbs free energy: -650952.42 kcal/mol

C	0.345658000	-1.719968000	0.737165000
C	1.695440000	-1.122787000	0.334877000
C	1.326405000	0.334155000	0.067870000
C	0.246880000	0.577671000	1.124764000
C	-0.852518000	1.528696000	0.710454000
H	0.895283000	0.395260000	-0.950770000
H	0.741202000	0.994124000	2.020579000
H	-0.405928000	2.533590000	0.556571000
H	-1.588994000	1.608663000	1.536492000
O	-0.287282000	-0.703482000	1.454359000
O	2.356487000	1.257192000	0.237774000
O	-1.462366000	1.063449000	-0.454916000
H	0.425843000	-2.599056000	1.393849000
H	2.415771000	-1.182978000	1.168743000
C	3.342657000	1.190570000	-0.761455000
H	2.897780000	1.287231000	-1.770856000
H	4.037362000	2.027332000	-0.594058000
H	3.911066000	0.242747000	-0.718699000
C	-2.526035000	1.866176000	-0.880174000
H	-2.936216000	1.420196000	-1.798989000
H	-3.328481000	1.916144000	-0.117083000
H	-2.192033000	2.899355000	-1.103295000
C	-2.225966000	-2.171367000	-0.099566000
H	-2.908642000	-2.528704000	-0.886316000
H	-2.315607000	-2.831955000	0.778305000
H	-2.477050000	-1.135289000	0.169180000
F	2.242542000	-1.748711000	-0.756457000
S	-0.556825000	-2.220800000	-0.793239000

----

5 References

1. H. Xu, R. N. Schaugaard, J. Li, H. B. Schlegel, H. M. Nguyen, *J. Am. Chem. Soc.* **2022**, *144*, 7441−7456.
2. P. M. DeMent, C. Liu, J. Wakpal, R. N. Schaugaard, H. B. Schlegel, H. M. Nguyen, *ACS Catal.* **2021**, *11*, 2108−2120.
3. S. Zhu, G. Samala, E. T. Sletten, J. L. Stockdill, H. M. Nguyen, *Chem. Sci*. **2019**, *10*, 10475–10480.
4. M. Kicsak, M. Bege, I. Bereczki, M. Csavas, M. Herczeg, Z. Kupihar, L. Kovacs, A. Borbas, P. Herczegh, *Org. Biomol. Chem.*, **2016**, *14*, 3190–3192.
5. T. K. Chakraborty, D. Koley, R. Ravi, V. Krishnakumari, R. agaraj, A. C. Kunwar, *J. Org. Chem.* **2008**, *73*, 8731–8744.
6. C. Jia, Y. Zhang, L.-H. Zhang, P. Sinay, M. Sollogoub, *Carbohydr. Res.* **2006**, *341*, 2135–2144.
7. S. Fuse, Y. Mifune, T. Takahashi, *Angew. Chem. Int. Ed.* **2014**, *53*, 851–855.
8. A. B. Mayfield, J. B. Metternich, A. H. Trotta, E. N. Jacobsen, *J. Am. Chem. Soc.* **2020,** *142*, 4061–4069.
9. Y. J. Lee, K. Lee, E. H. Jung, H. B. Jeon, K. S. Kim, *Org. Lett.* **2005**, *7*, 3263–3266.
10. M. O. Mohammed, J. R. Al Dulayymi, M. S. Baird, *Carbohydr. Res.* **2017**, *437*, 36–42.
11. M. J. Frisch, G. W. Trucks, H. B. Schlegel, G. E. Scuseria, M. A. Robb, J. R. Cheeseman, G. Scalmani, V. Barone, G. A. Petersson, H. Nakatsuji, X. Li, M. Caricato, A. V. Marenich, J. Bloino, B. G. Janesko, R. Gomperts, B. Mennucci, H. P. Hratchian, J. V. Ortiz, A. F. Izmaylov, J. L. Sonnenberg, D. Williams-Young, F. Ding, F. Lipparini, F. Egidi, J. Goings, B. Peng, A. Petrone, T. Henderson, D. Ranasinghe, V. G. Zakrzewski, J. Gao, N. Rega, G. Zheng, W. Liang, M. Hada, M. Ehara, K. Toyota, R. Fukuda, J. Hasegawa, M. Ishida, T. Nakajima, Y. Honda, O. Kitao, H. Nakai, T. Vreven, K. Throssell, J. A. Montgomery, Jr., J. E. Peralta, F. Ogliaro, M. J. Bearpark, J. J. Heyd, E. N. Brothers, K. N. Kudin, V. N. Staroverov, T. A. Keith, R. Kobayashi, J. Normand, K. Raghavachari, A. P. Rendell, J. C. Burant, S. S. Iyengar, J. Tomasi, M. Cossi, J. M. Millam, M. Klene, C. Adamo, R. Cammi, J. W. Ochterski, R. L. Martin, K. Morokuma, O. Farkas, J. B. Foresman, and D. J. Fox, Gaussian 16, Revision B.01, Gaussian, Inc., Wallingford CT, 2016.
12. Y. Wang, P. Verma, X. Jin, D. G. Truhlar, X. He, *Proc. Natl. Acad. Sci. U.S.A.* **2018**, *115*, 10257–10262.
13. Y. Zhao, D. G. Truhlar, *Theor. Chem. Acc.* **2008**, *120*, 215–241.
14. F. Weigend, R. Ahlrichs, *Phys. Chem. Chem. Phys.* **2005**, *7*, 3297–3305.
15. A. Schäfer, C. Huber, R. Ahlrichs, *J. Chem. Phys.* **1994**, *100*, 5829–5835.
16. A. V. Marenich, C. J. Cramer, D. G. Truhlar, *J. Phys. Chem. B.* **2009**, *113*, 6378–6396.
17. L. E. Chirlian, M. M. Francl, *J. Comb. Chem.* **1987**, *8*, 894–905.

6 Spectral Data

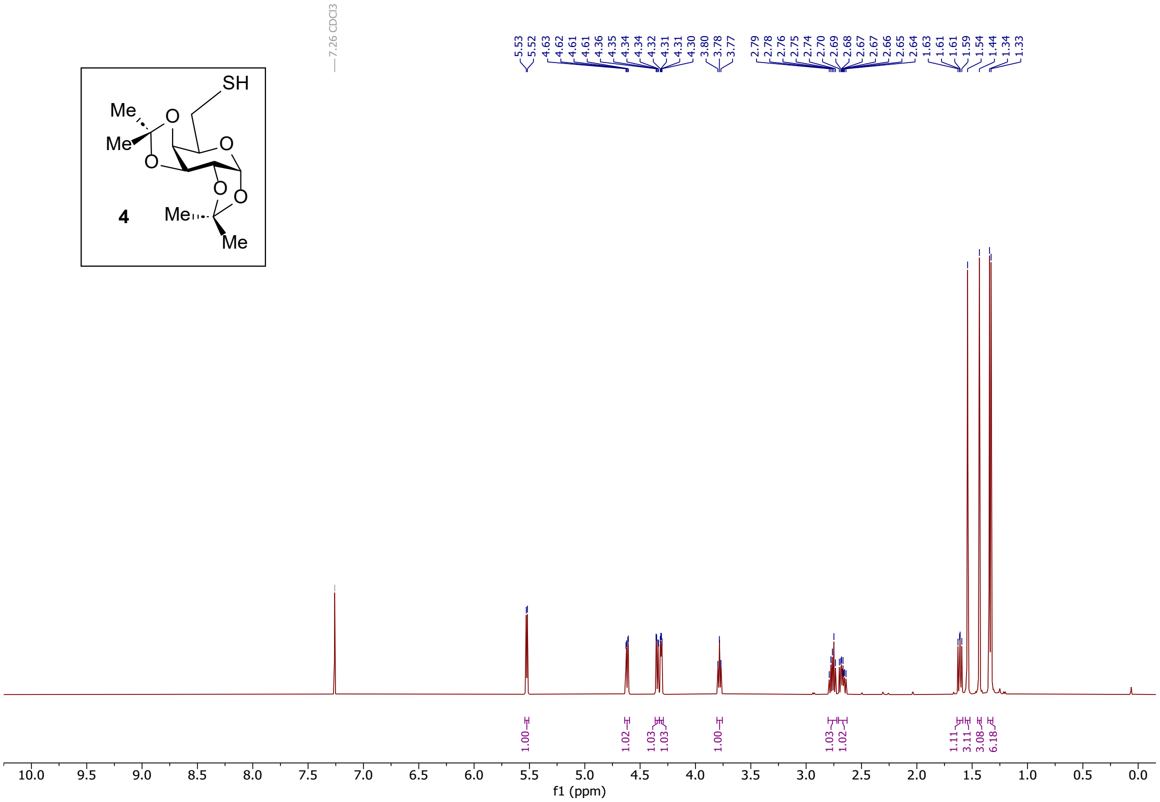

4

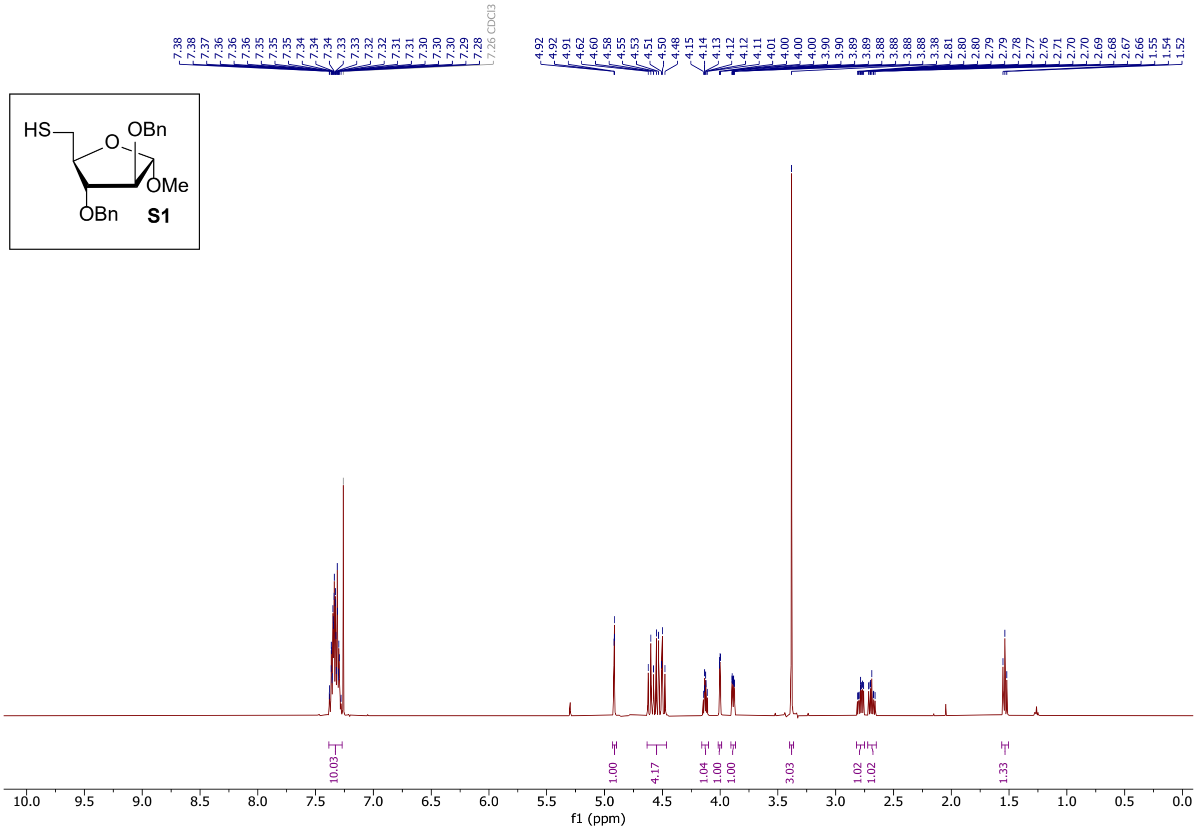

S1

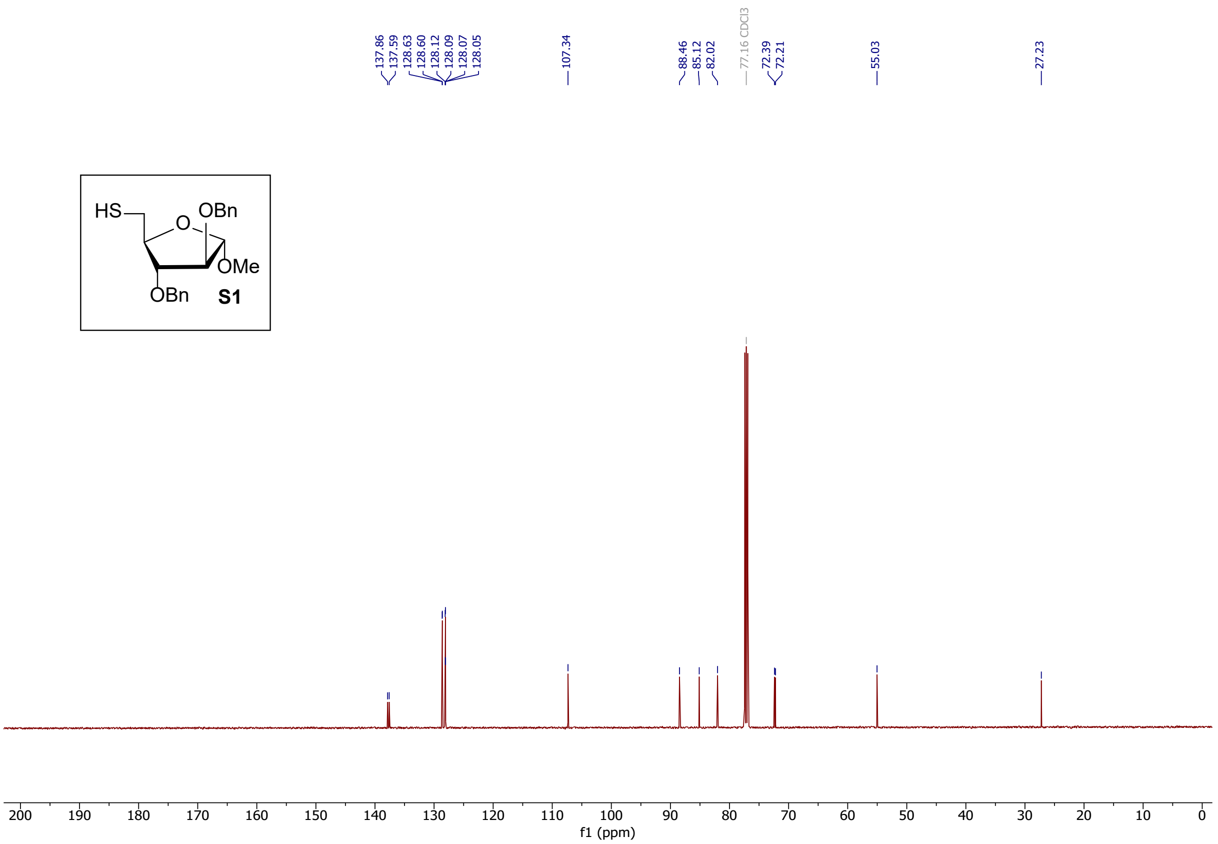

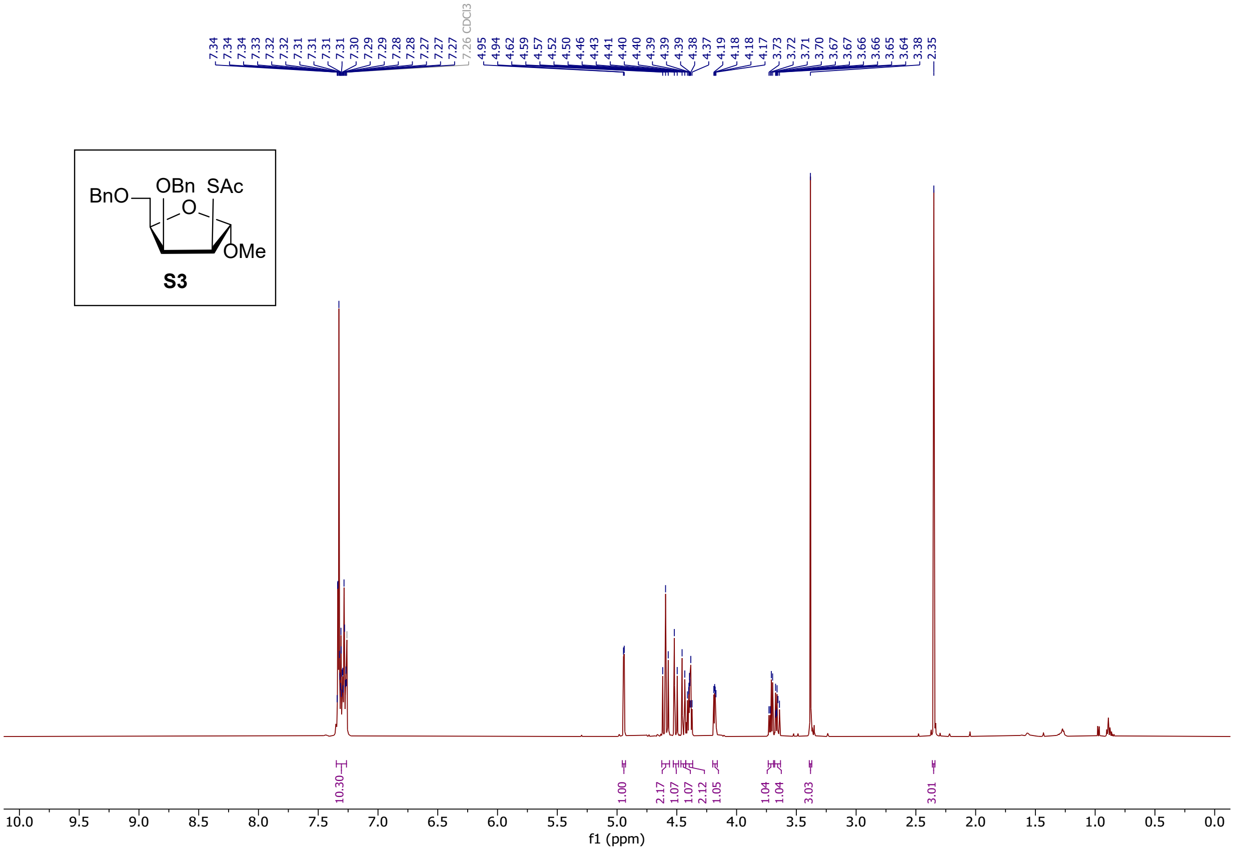

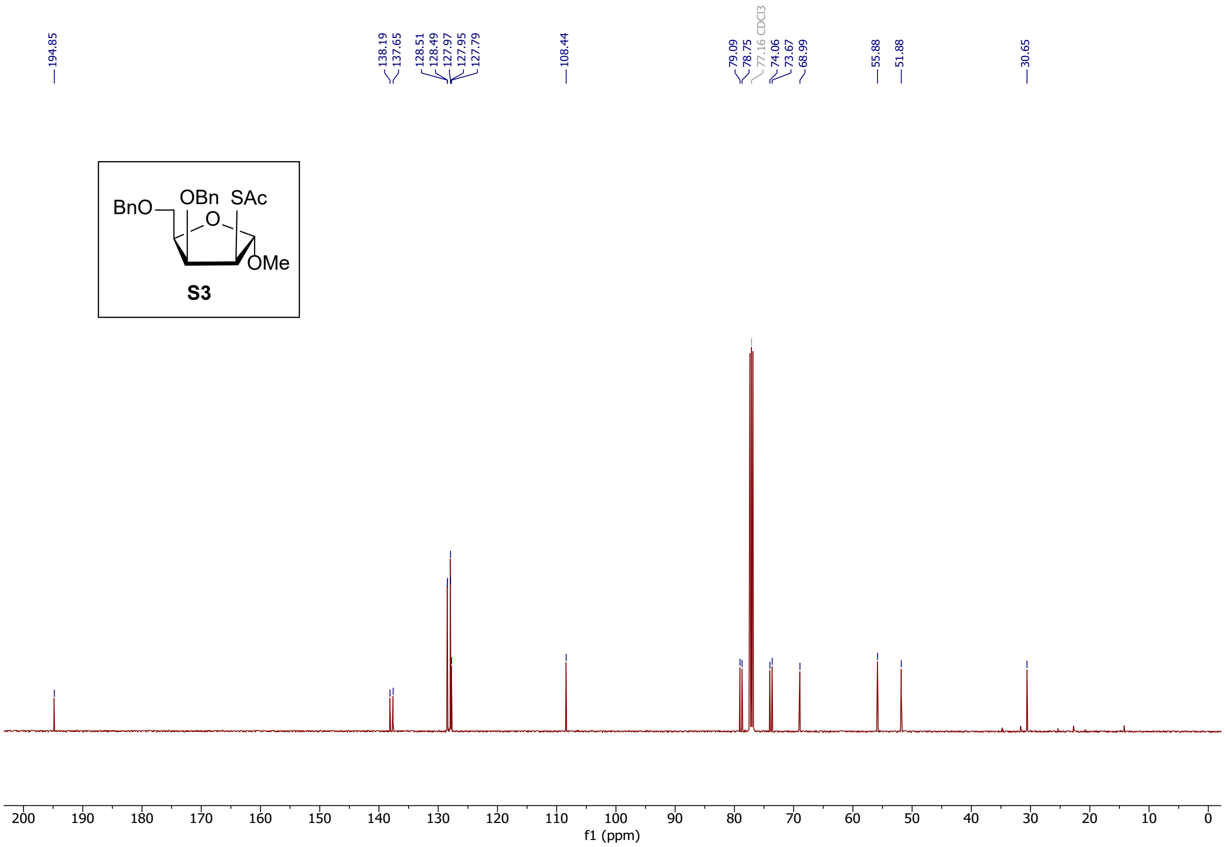
194.85
138.19
137.65
128.51
128.49
127.97
127.95
127.79
108.44
79.09
78.75
77.16 CDCl3
74.06
73.67
68.99
55.88
51.88
30.65
BnO
OBn
SAc
O
OMe
S3
f1 (ppm)

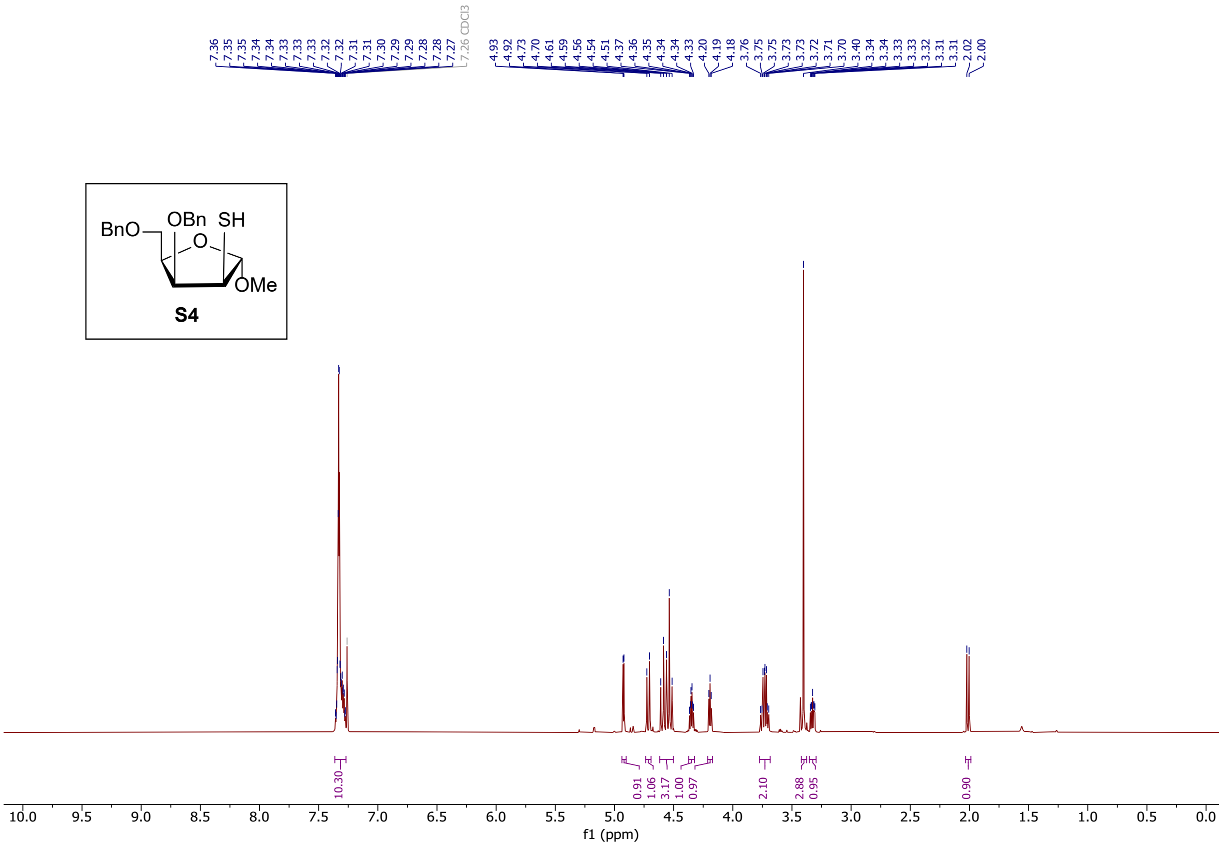
7.36
7.35
7.35
7.34
7.34
7.33
7.33
7.33
7.32
7.32
7.31
7.31
7.30
7.29
7.29
7.28
7.28
7.27
7.26 CDCl3
4.93
4.92
4.73
4.70
4.61
4.59
4.56
4.54
4.51
4.37
4.36
4.35
4.34
4.34
4.33
4.20
4.19
4.18
3.76
3.75
3.75
3.73
3.73
3.72
3.71
3.70
3.40
3.34
3.34
3.33
3.33
3.32
3.31
3.31
2.02
2.00
BnO
OBn
SH
O
OMe
S4
10.30
0.91
1.06
3.17
1.00
0.97
2.10
2.88
0.95
0.90
f1 (ppm)

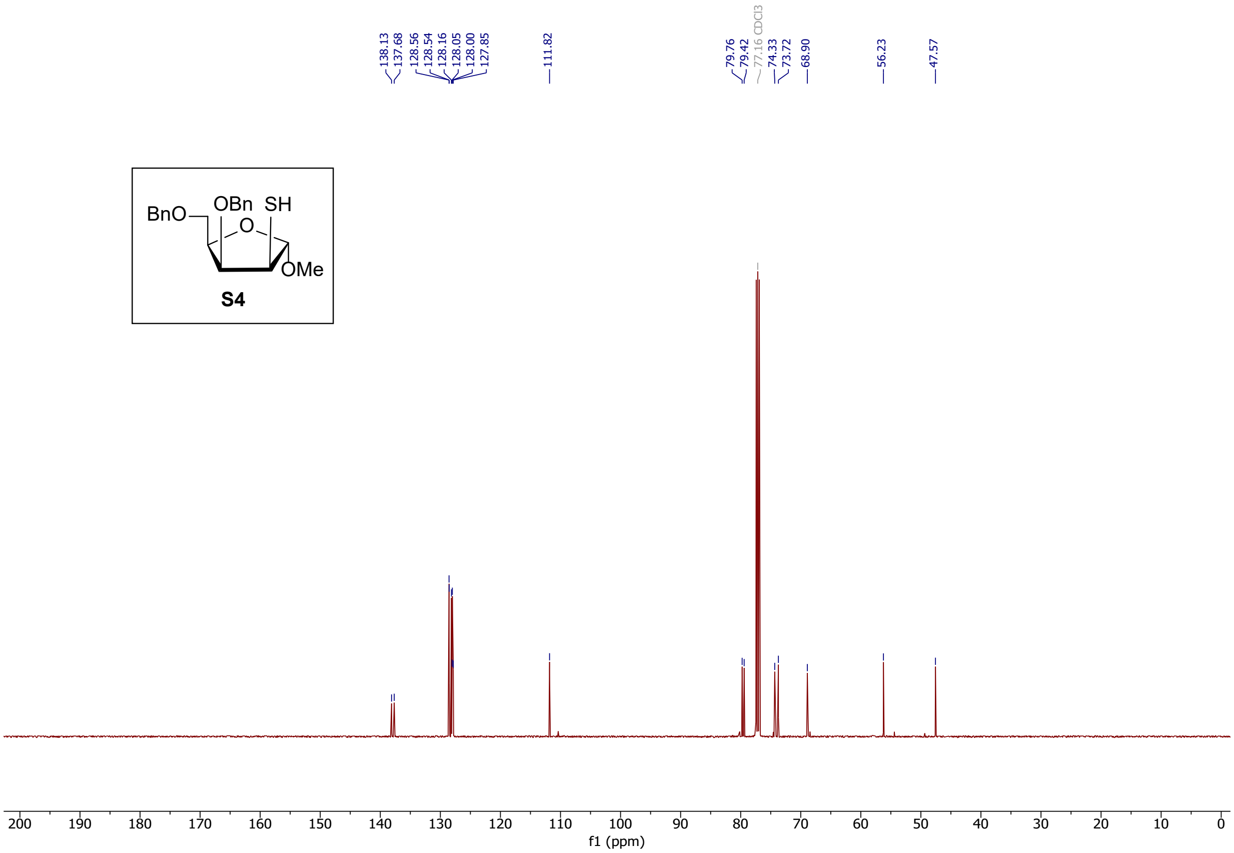
138.13
137.68
128.56
128.54
128.16
128.05
128.00
127.85
111.82
79.76
79.42
77.16 CDCl3
74.33
73.72
68.90
56.23
47.57
BnO
OBn
SH
O
OMe
S4
200 190 180 170 160 150 140 130 120 110 100 90 80 70 60 50 40 30 20 10 0
f1 (ppm)

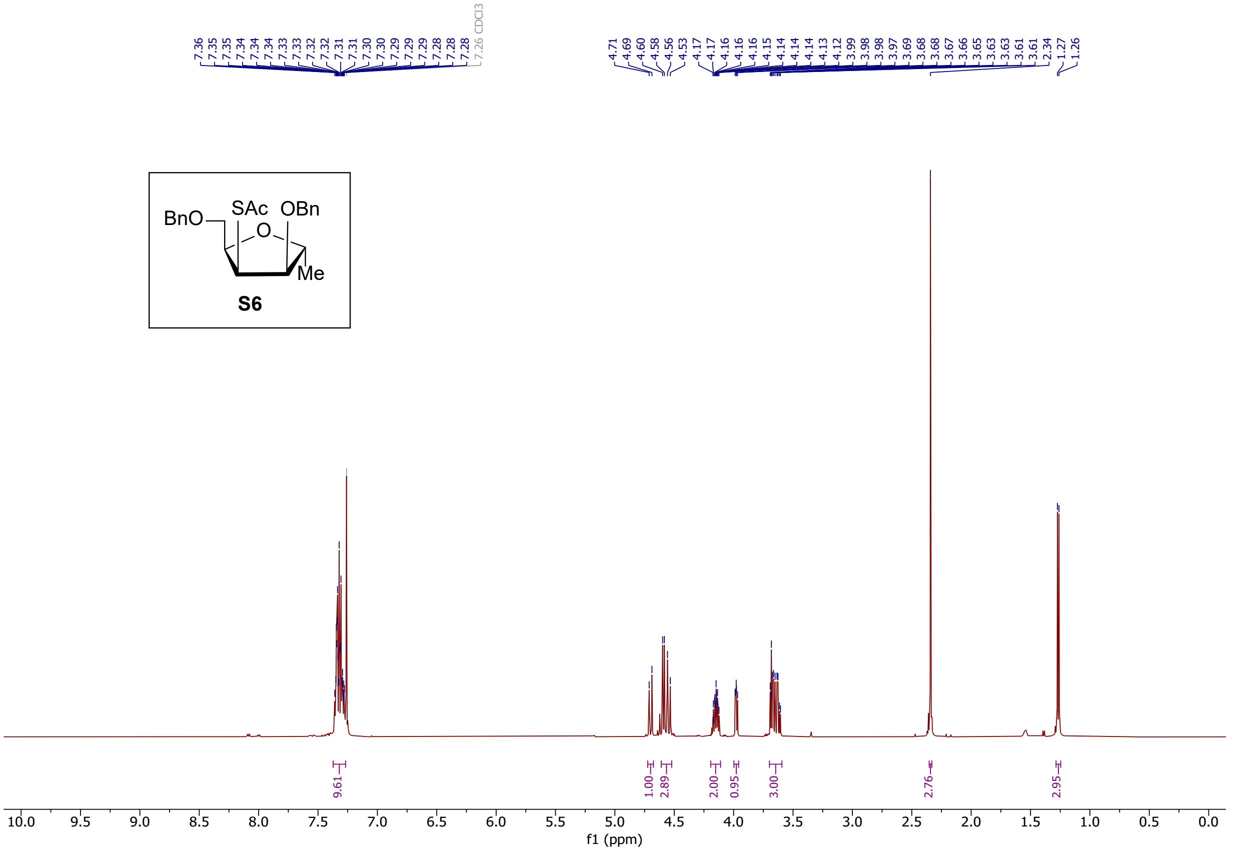
7.36
7.35
7.35
7.34
7.34
7.34
7.33
7.33
7.32
7.32
7.31
7.31
7.30
7.30
7.29
7.29
7.29
7.28
7.28
7.28
7.26 CDCl3
4.71
4.69
4.60
4.58
4.56
4.53
4.17
4.16
4.16
4.16
4.15
4.14
4.14
4.14
4.13
4.12
3.99
3.98
3.98
3.97
3.69
3.68
3.68
3.67
3.66
3.65
3.63
3.63
3.61
3.61
2.34
1.27
1.26
BnO
SAc
OBn
O
Me
S6
9.61
1.00
2.89
2.00
0.95
3.00
2.76
2.95
10.0 9.5 9.0 8.5 8.0 7.5 7.0 6.5 6.0 5.5 5.0 4.5 4.0 3.5 3.0 2.5 2.0 1.5 1.0 0.5 0.0
f1 (ppm)

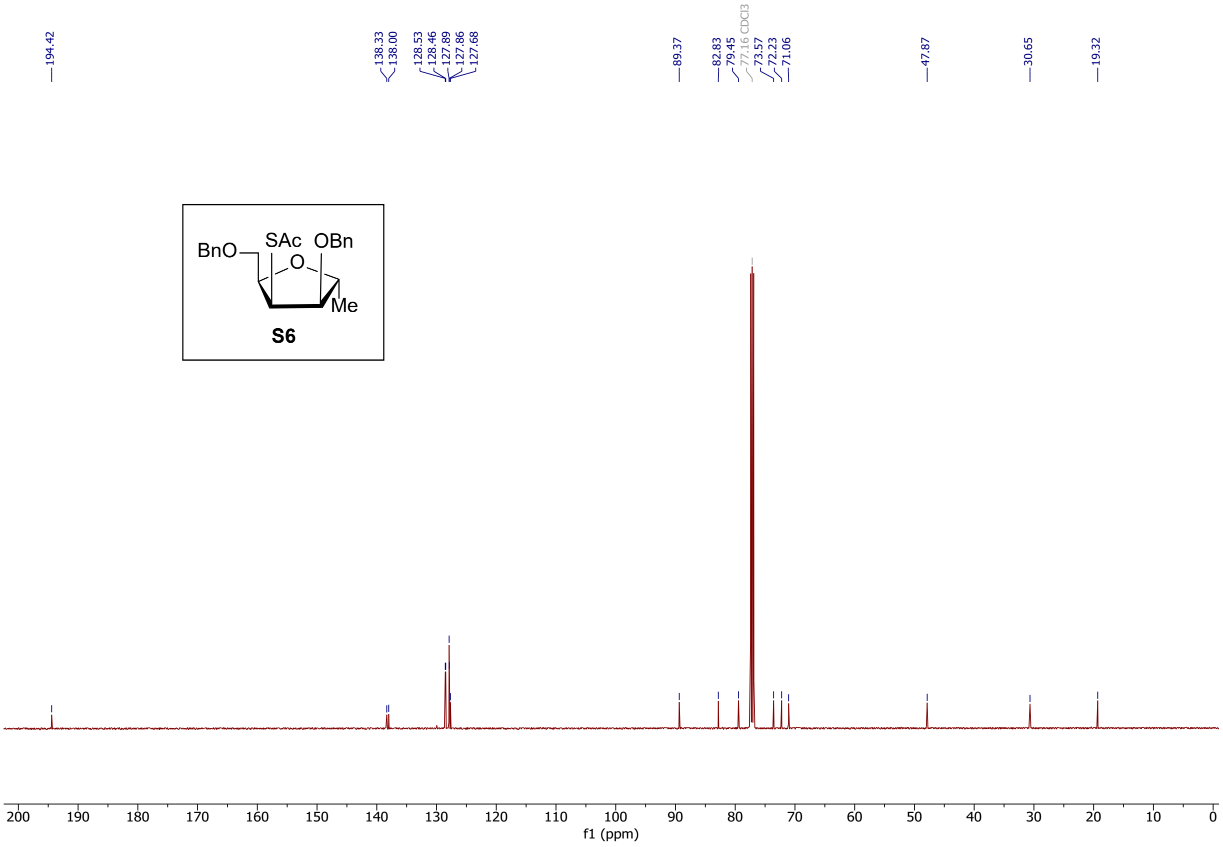
194.42
138.33
138.00
128.53
128.46
127.89
127.86
127.68
89.37
82.83
79.45
77.16 CDCl3
73.57
72.23
71.06
47.87
30.65
19.32
BnO
SAc
OBn
O
Me
S6
200 190 180 170 160 150 140 130 120 110 100 90 80 70 60 50 40 30 20 10 0
f1 (ppm)

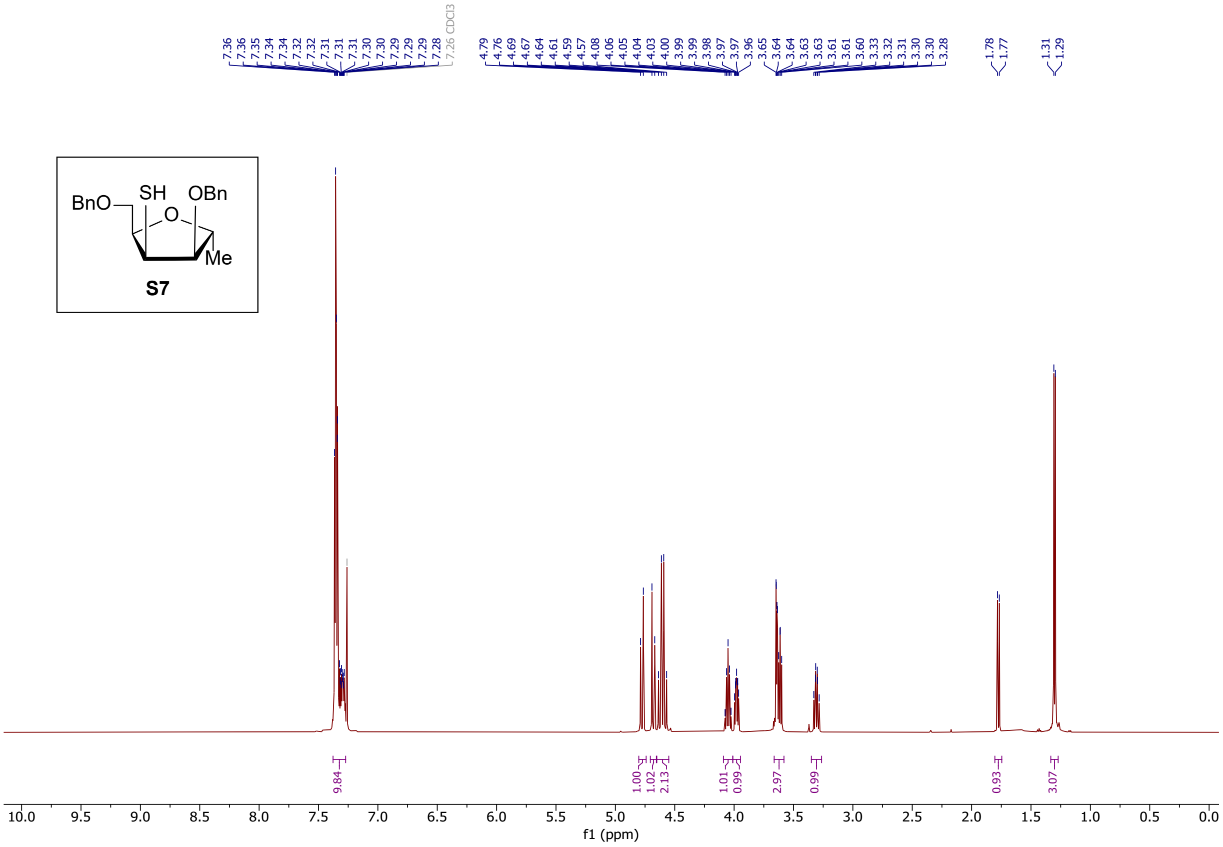
7.36
7.36
7.35
7.34
7.34
7.32
7.32
7.31
7.31
7.31
7.30
7.30
7.29
7.29
7.29
7.28
7.26 CDCl3
4.79
4.76
4.69
4.67
4.64
4.61
4.59
4.57
4.08
4.06
4.05
4.04
4.03
4.00
3.99
3.99
3.98
3.97
3.97
3.96
3.65
3.64
3.64
3.63
3.63
3.61
3.61
3.60
3.33
3.32
3.31
3.30
3.30
3.28
1.78
1.77
1.31
1.29
BnO
SH
OBn
O
Me
S7
9.84
1.00
1.02
2.13
1.01
0.99
2.97
0.99
0.93
3.07
10.0 9.5 9.0 8.5 8.0 7.5 7.0 6.5 6.0 5.5 5.0 4.5 4.0 3.5 3.0 2.5 2.0 1.5 1.0 0.5 0.0
f1 (ppm)

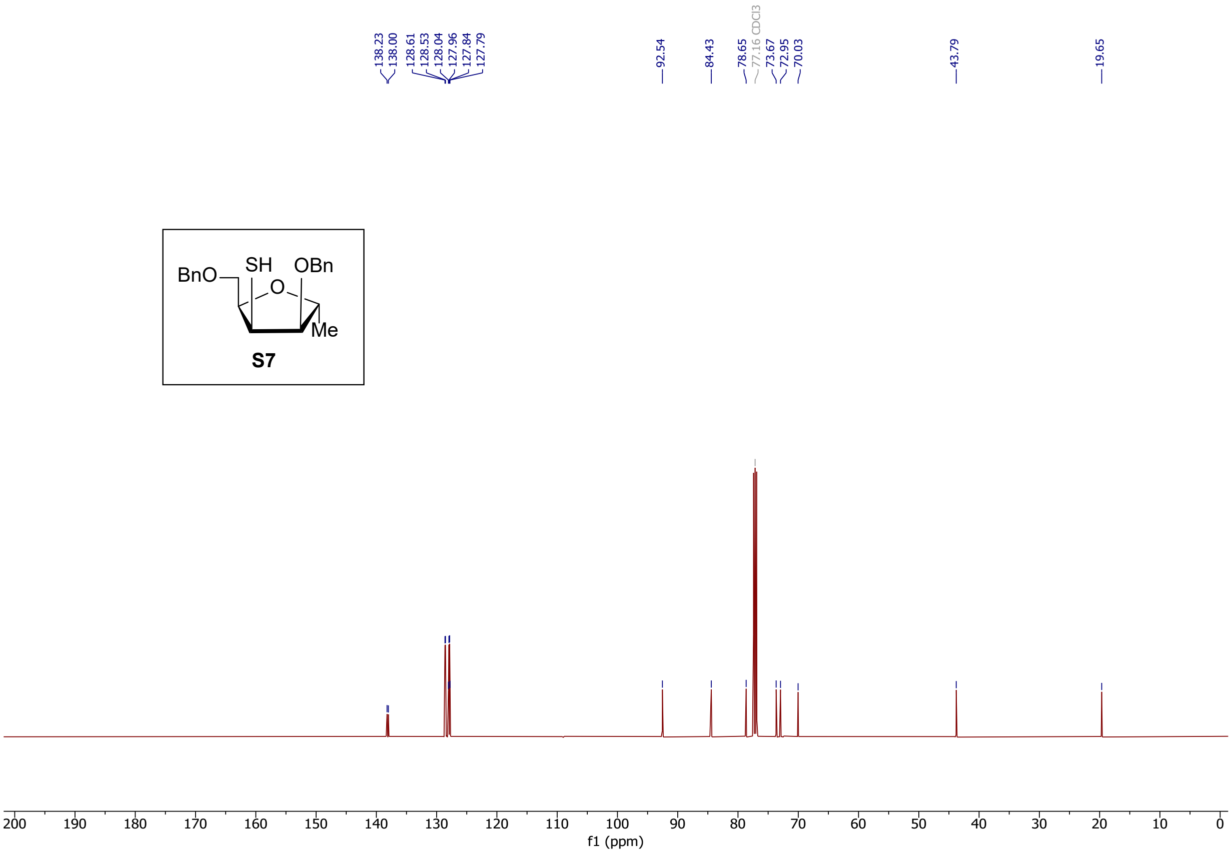
138.23
138.00
128.61
128.53
128.04
127.96
127.84
127.79
92.54
84.43
78.65
77.16 CDCl3
73.67
72.95
70.03
43.79
19.65
BnO
SH
OBn
O
Me
S7
f1 (ppm)

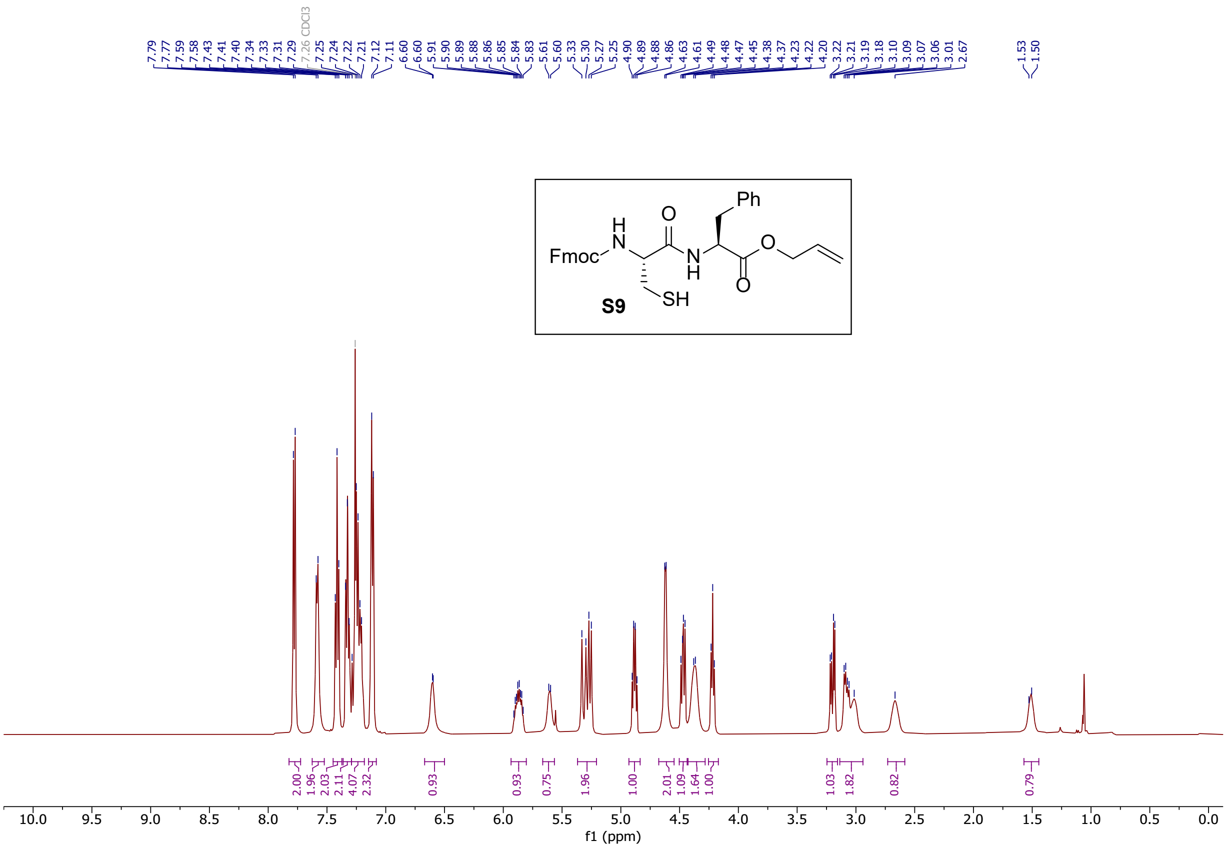
7.79
7.77
7.59
7.58
7.43
7.41
7.40
7.34
7.33
7.31
7.29
7.26 CDCl3
7.25
7.24
7.22
7.21
7.12
7.11
6.60
6.60
5.91
5.90
5.89
5.88
5.88
5.86
5.85
5.84
5.83
5.61
5.60
5.33
5.30
5.27
5.25
4.90
4.89
4.88
4.86
4.63
4.61
4.49
4.48
4.47
4.45
4.38
4.37
4.23
4.22
4.20
3.22
3.21
3.19
3.18
3.10
3.09
3.07
3.06
3.01
2.67
1.53
1.50
Fmoc
H
N
O
N
H
Ph
O
O
SH
S9
2.00
1.96
2.03
2.11
4.07
2.32
0.93
0.93
0.75
1.96
1.00
2.01
1.09
1.64
1.00
1.03
1.82
0.82
0.79
f1 (ppm)

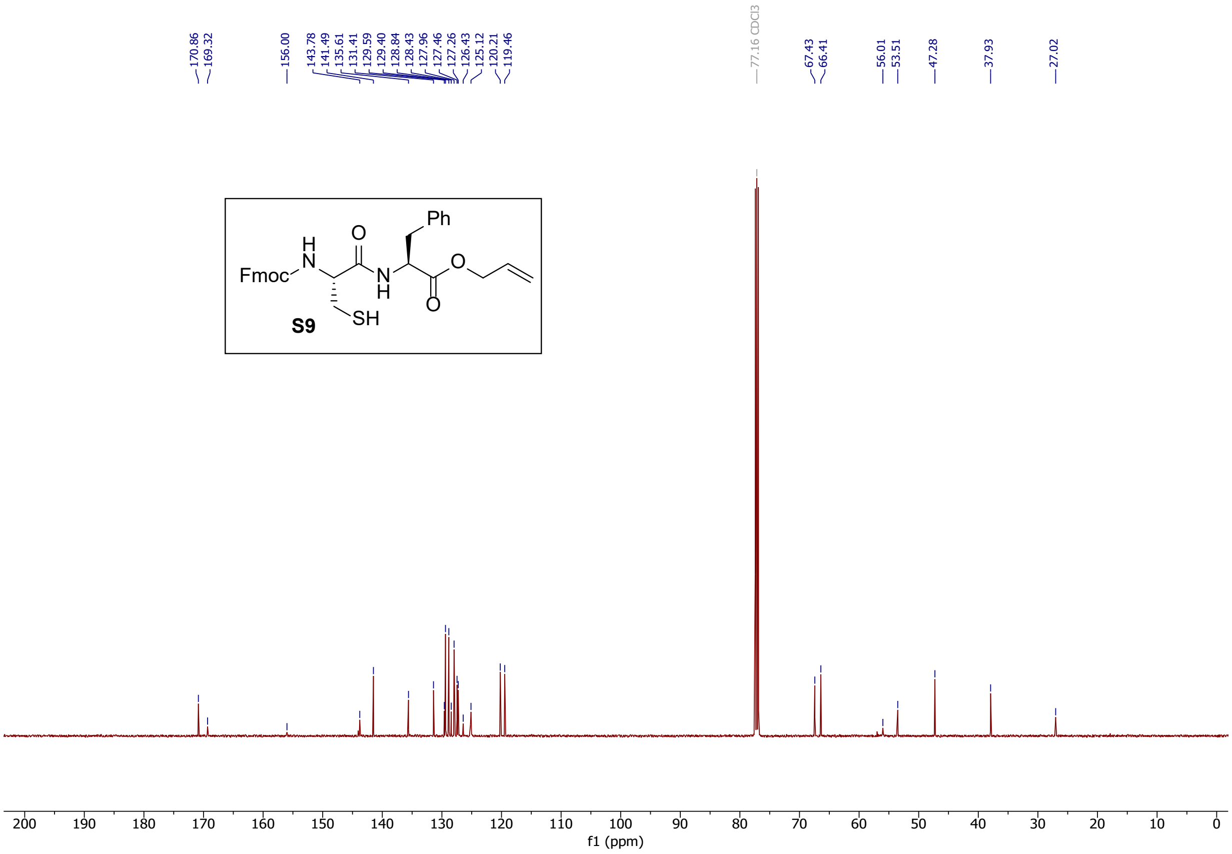
170.86 169.32 156.00 143.78 141.49 135.61 131.41 129.59 129.40 128.84 128.43 127.96 127.46 127.26 126.43 125.12 120.21 119.46
77.16 CDCl3
67.43 66.41 56.01 53.51 47.28 37.93 27.02
Ph
O
H
N
Fmoc
N
H
O
O
SH
S9
200 190 180 170 160 150 140 130 120 110 100 90 80 70 60 50 40 30 20 10 0
f1 (ppm)

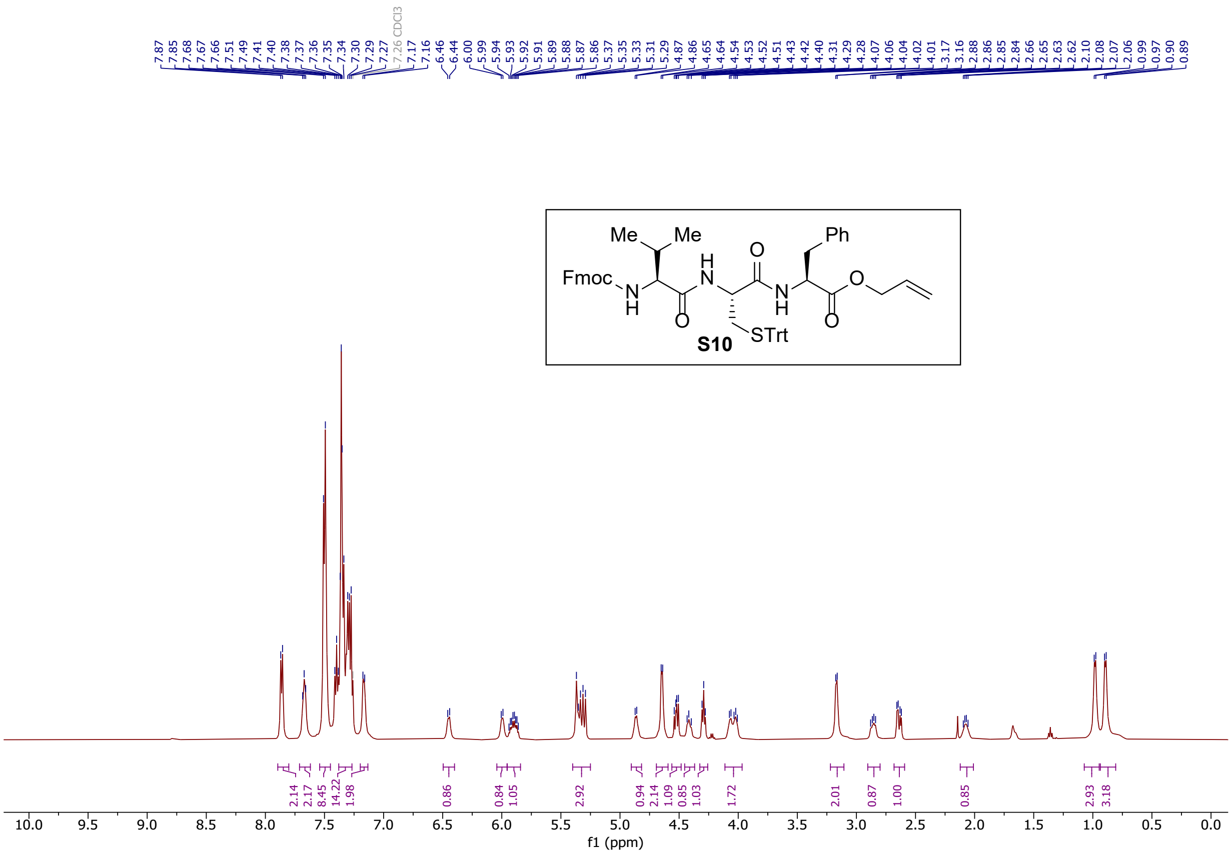
7.87 7.85 7.68 7.67 7.66 7.51 7.49 7.41 7.40 7.38 7.37 7.36 7.35 7.34 7.30 7.29 7.27
7.26 CDCl3
7.17 7.16 6.46 6.44 6.00 5.99 5.94 5.93 5.92 5.91 5.89 5.88 5.87 5.86 5.37 5.35 5.33 5.31 5.29 4.87 4.86 4.65 4.64 4.54 4.53 4.52 4.51 4.43 4.42 4.40 4.31 4.29 4.28 4.07 4.06 4.04 4.02 4.01 3.17 3.16 2.88 2.86 2.85 2.84 2.66 2.65 2.63 2.62 2.10 2.08 2.07 2.06 0.99 0.97 0.90 0.89
Me
Me
Ph
Fmoc
N
H
H
N
O
N
H
O
O
O
STrt
S10
2.14 2.17 8.45 14.22 1.98 0.86 0.84 1.05 2.92 0.94 2.14 1.09 0.85 1.03 1.72 2.01 0.87 1.00 0.85 2.93 3.18
10.0 9.5 9.0 8.5 8.0 7.5 7.0 6.5 6.0 5.5 5.0 4.5 4.0 3.5 3.0 2.5 2.0 1.5 1.0 0.5 0.0
f1 (ppm)

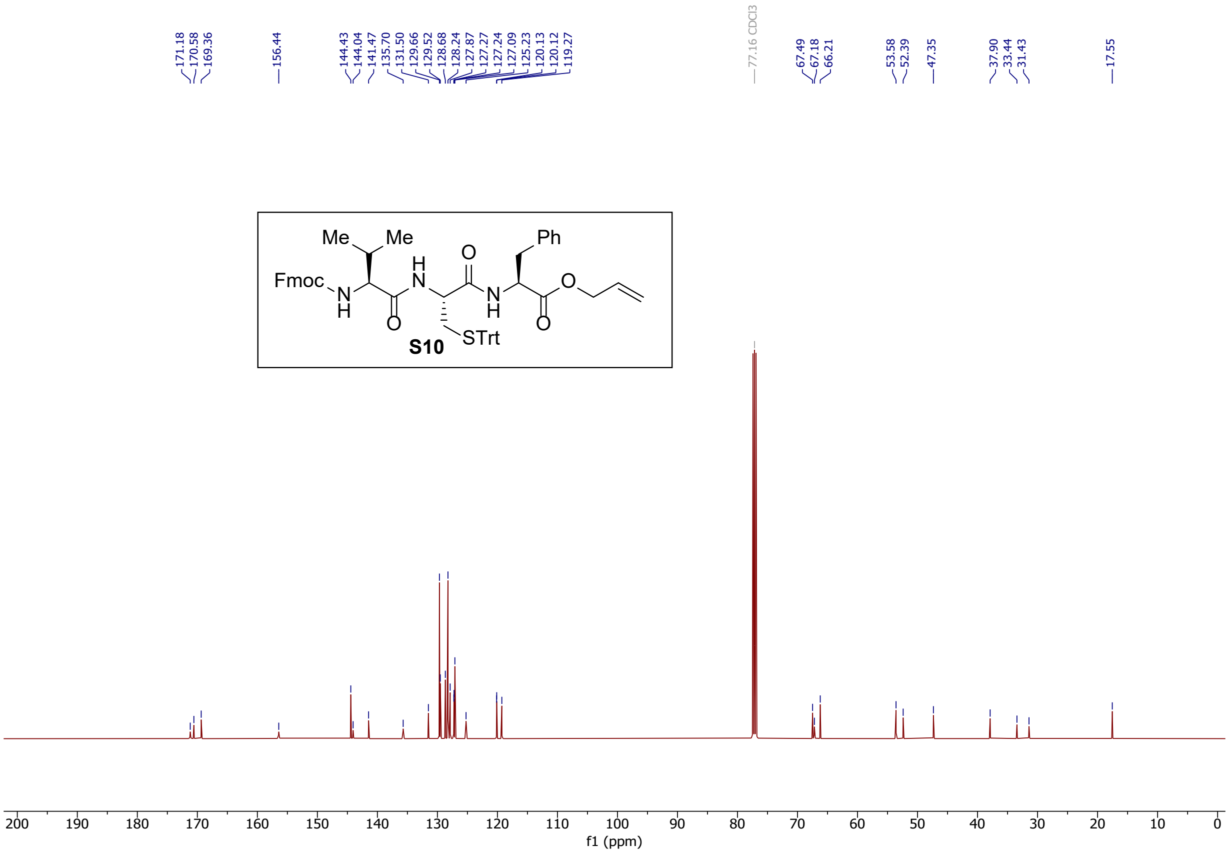
171.18
170.58
169.36
156.44
144.43
144.04
141.47
135.70
131.50
129.66
129.52
128.68
128.24
127.87
127.27
127.24
127.09
125.23
120.13
120.12
119.27
77.16 CDCl3
67.49
67.18
66.21
53.58
52.39
47.35
37.90
33.44
31.43
17.55
Me
Me
Ph
Fmoc
H
N
O
STrt
S10
f1 (ppm)

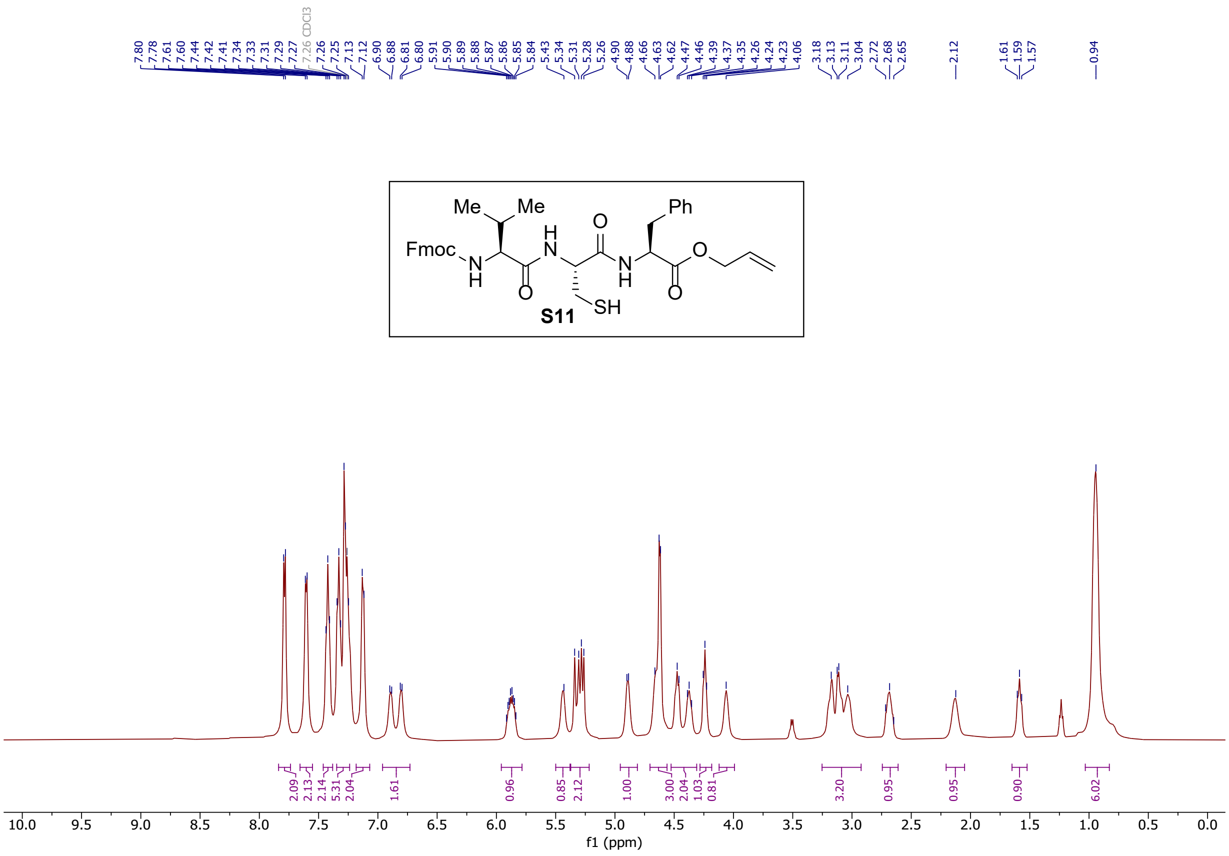
7.80
7.78
7.61
7.60
7.44
7.42
7.41
7.34
7.33
7.31
7.29
7.27
7.26 CDCl3
7.26
7.25
7.13
7.12
6.90
6.88
6.81
6.80
5.91
5.90
5.89
5.88
5.87
5.86
5.85
5.84
5.43
5.34
5.31
5.28
5.26
4.90
4.88
4.66
4.63
4.62
4.47
4.46
4.39
4.37
4.35
4.26
4.24
4.23
4.06
3.18
3.13
3.11
3.04
2.72
2.68
2.65
2.12
1.61
1.59
1.57
0.94
Me
Me
Ph
Fmoc
H
N
O
SH
S11
2.09
2.13
2.14
5.31
2.04
1.61
0.96
0.85
2.12
1.00
3.00
2.04
1.03
0.81
3.20
0.95
0.95
0.90
6.02
f1 (ppm)

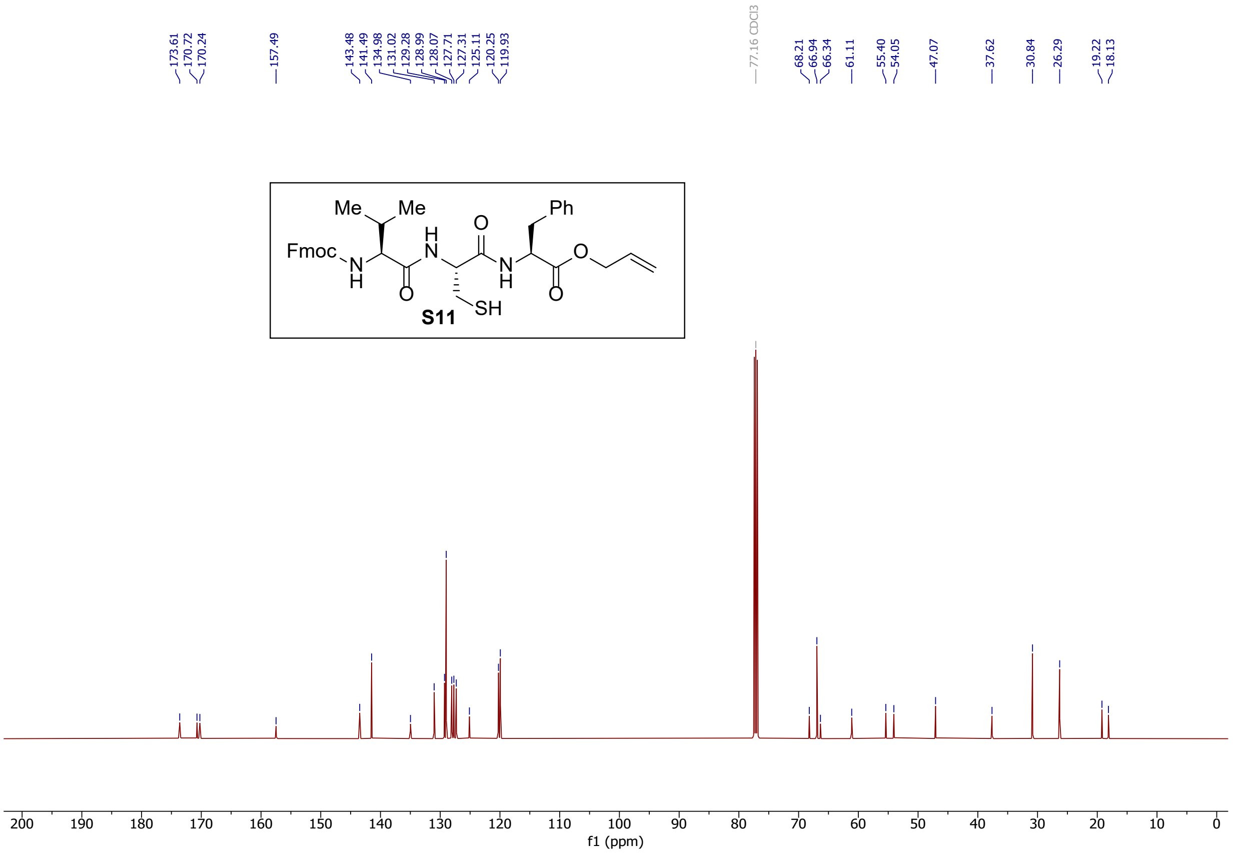
173.61
170.72
170.24
157.49
143.48
141.49
134.98
131.02
129.28
128.99
128.07
127.71
127.31
125.11
120.25
119.93
77.16 CDCl3
68.21
66.94
66.34
61.11
55.40
54.05
47.07
37.62
30.84
26.29
19.22
18.13
Me
Me
Ph
Fmoc
H
N
O
S11
SH
200 190 180 170 160 150 140 130 120 110 100 90 80 70 60 50 40 30 20 10 0
f1 (ppm)

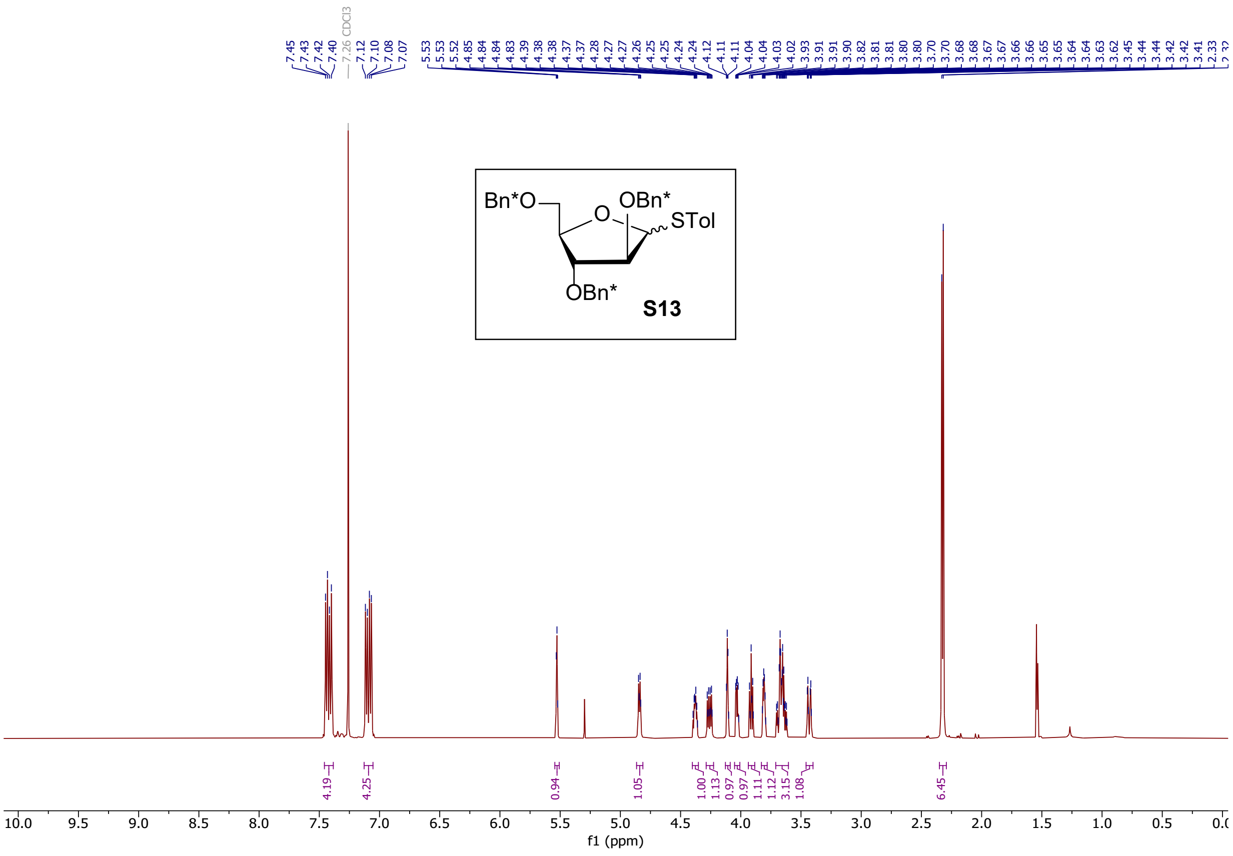
7.45
7.43
7.42
7.40
7.26 CDCl3
7.12
7.10
7.08
7.07
5.53
5.53
5.52
4.85
4.84
4.84
4.83
4.39
4.38
4.38
4.37
4.37
4.28
4.27
4.27
4.26
4.25
4.25
4.24
4.24
4.12
4.11
4.11
4.04
4.04
4.03
4.02
3.93
3.91
3.91
3.90
3.82
3.81
3.81
3.80
3.80
3.70
3.68
3.68
3.67
3.66
3.66
3.65
3.65
3.64
3.64
3.63
3.62
3.45
3.44
3.44
3.42
3.42
3.41
2.33
Bn*O
O
OBn*
STol
OBn*
S13
4.19
4.25
0.94
1.05
1.00
1.13
0.97
0.97
1.11
1.12
3.15
1.08
6.45
10.0 9.5 9.0 8.5 8.0 7.5 7.0 6.5 6.0 5.5 5.0 4.5 4.0 3.5 3.0 2.5 2.0 1.5 1.0 0.5
f1 (ppm)

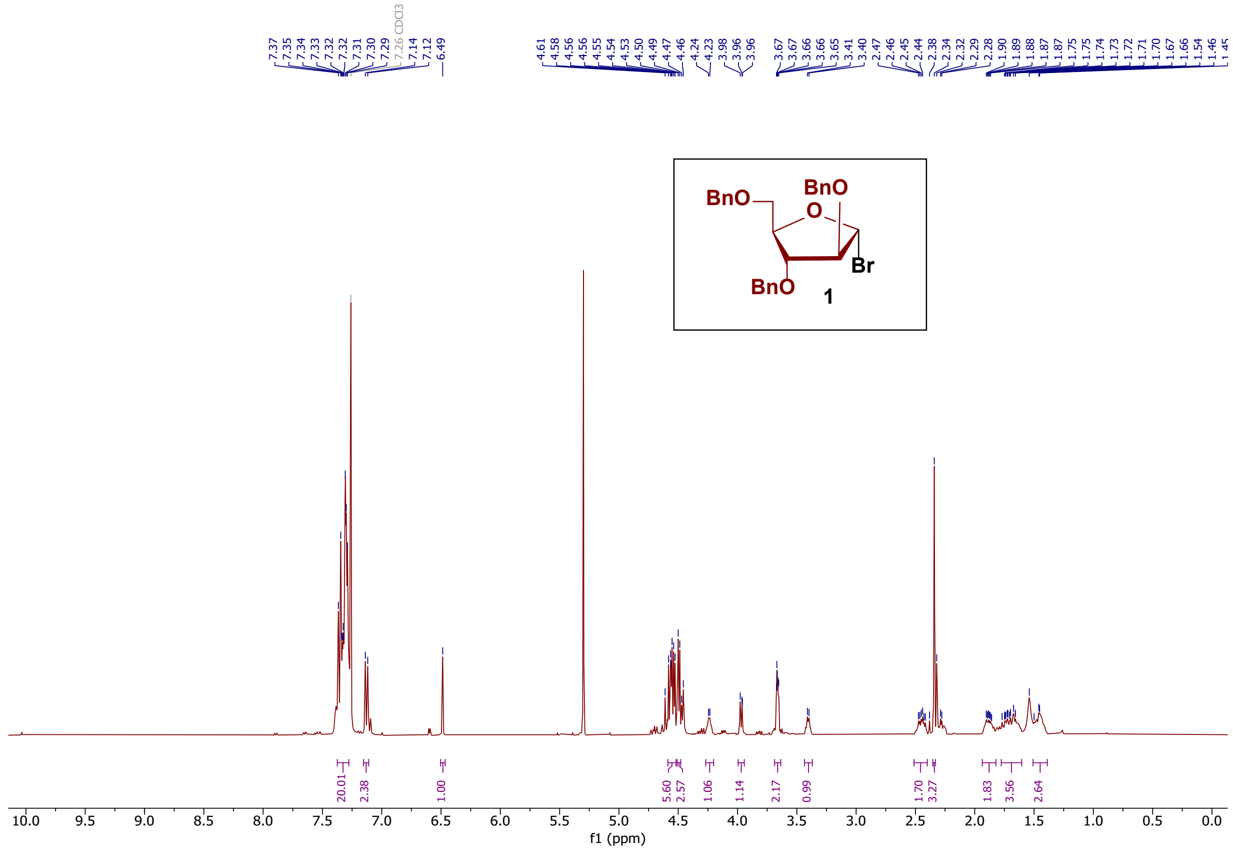

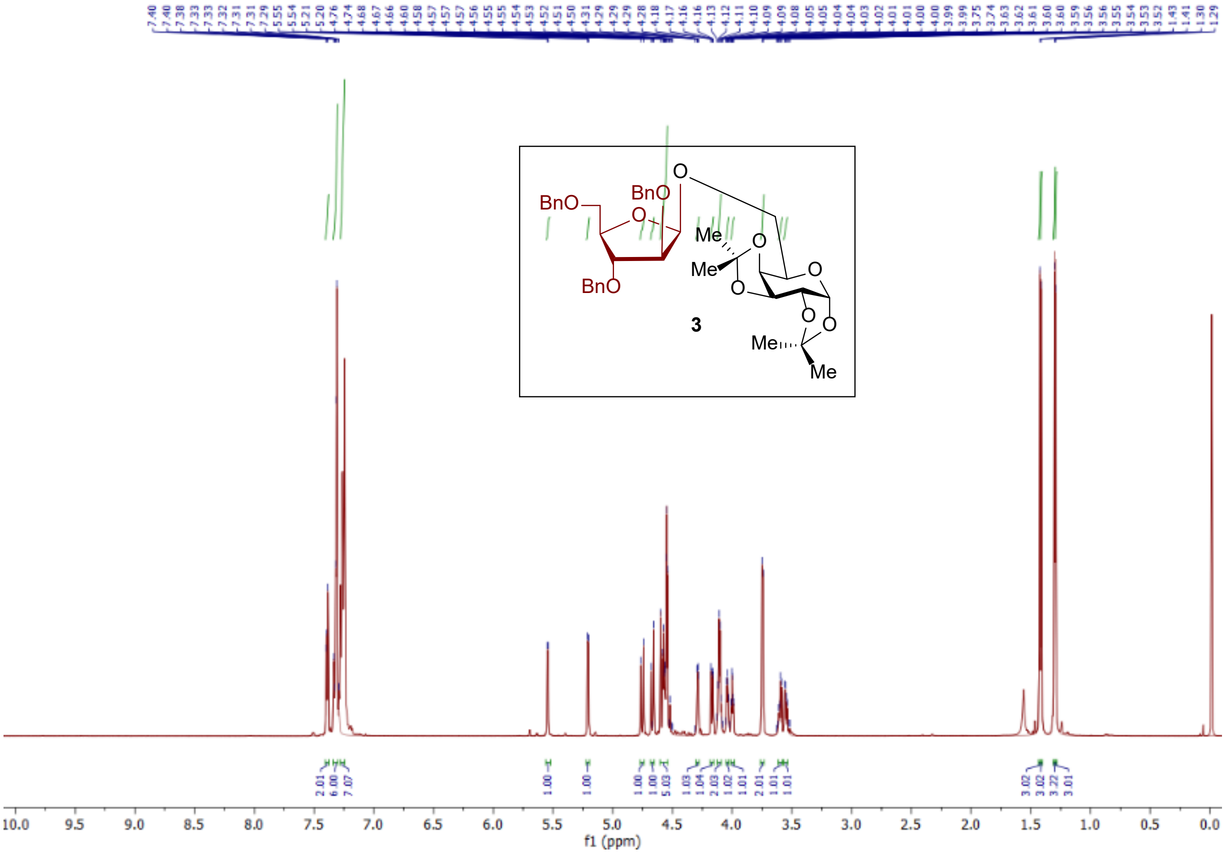

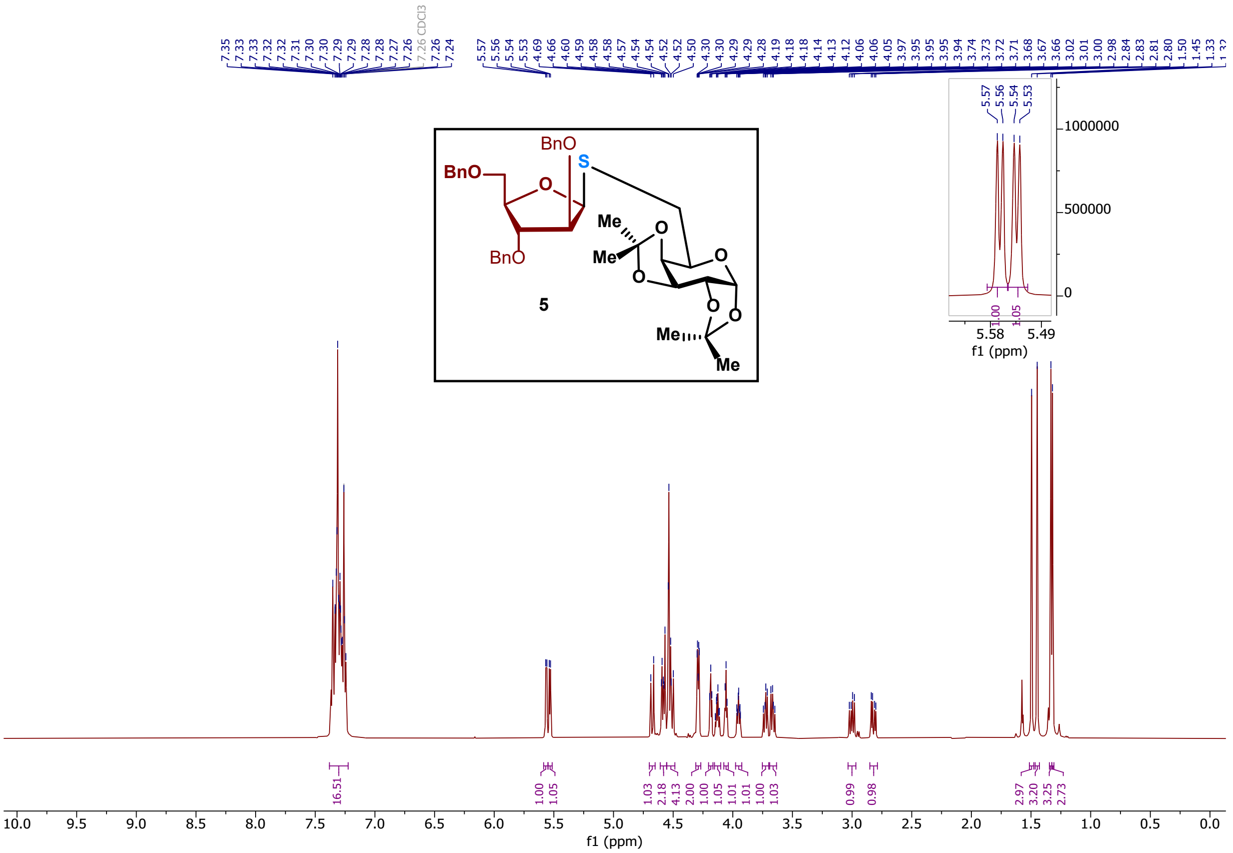

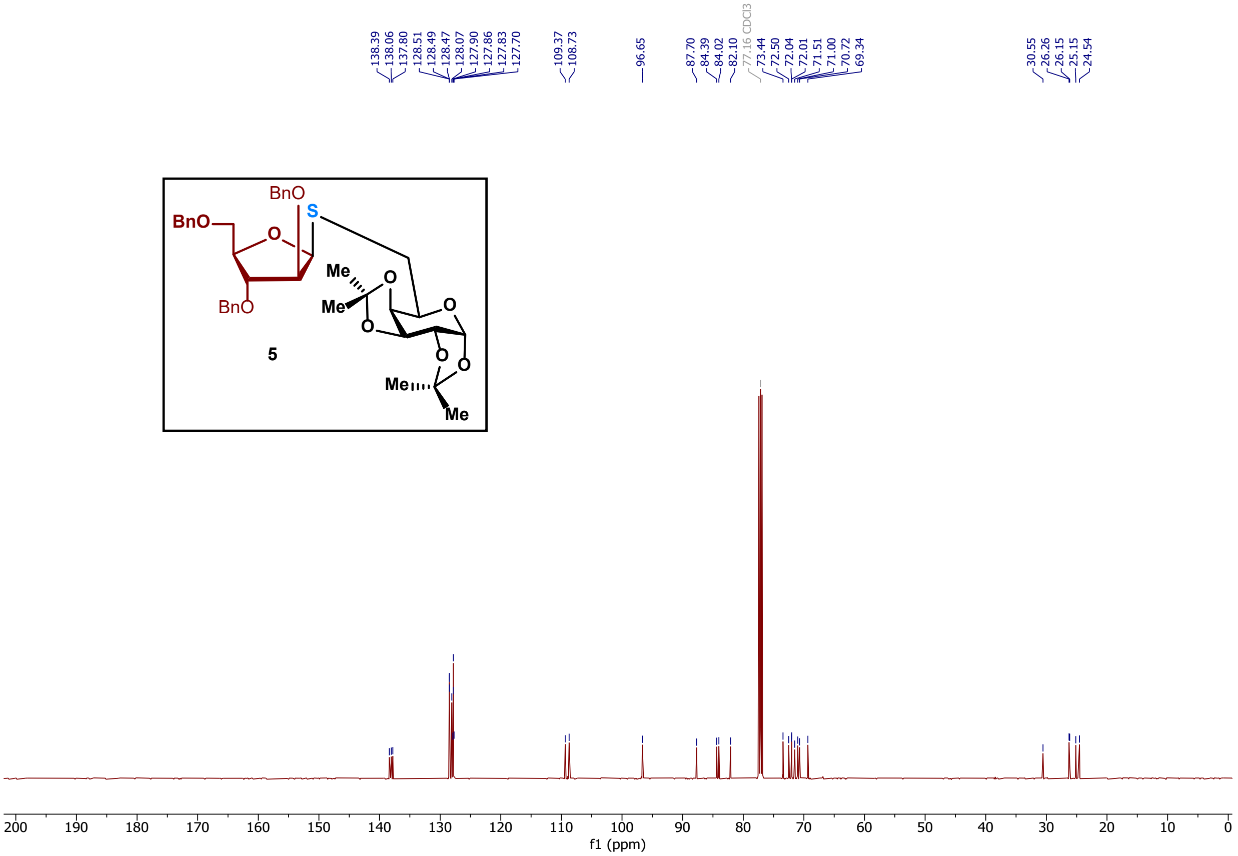

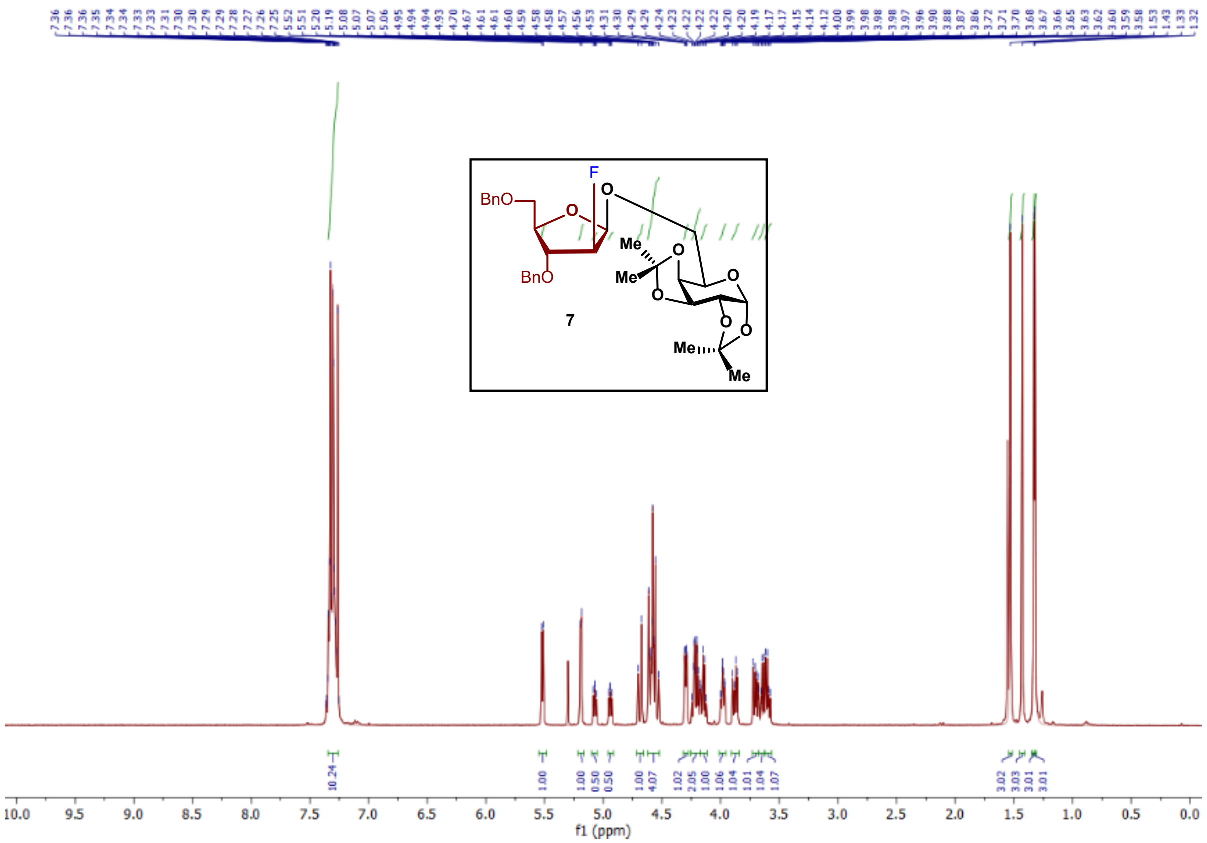

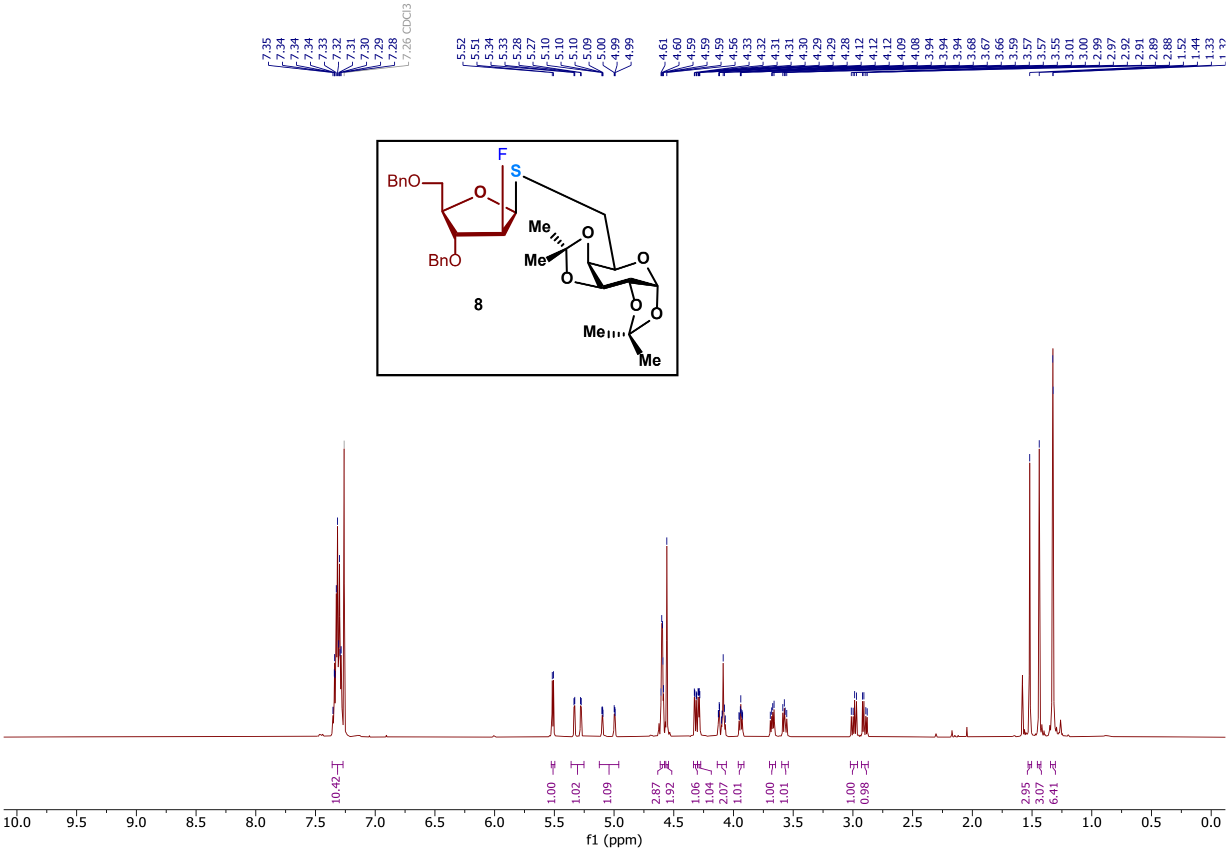

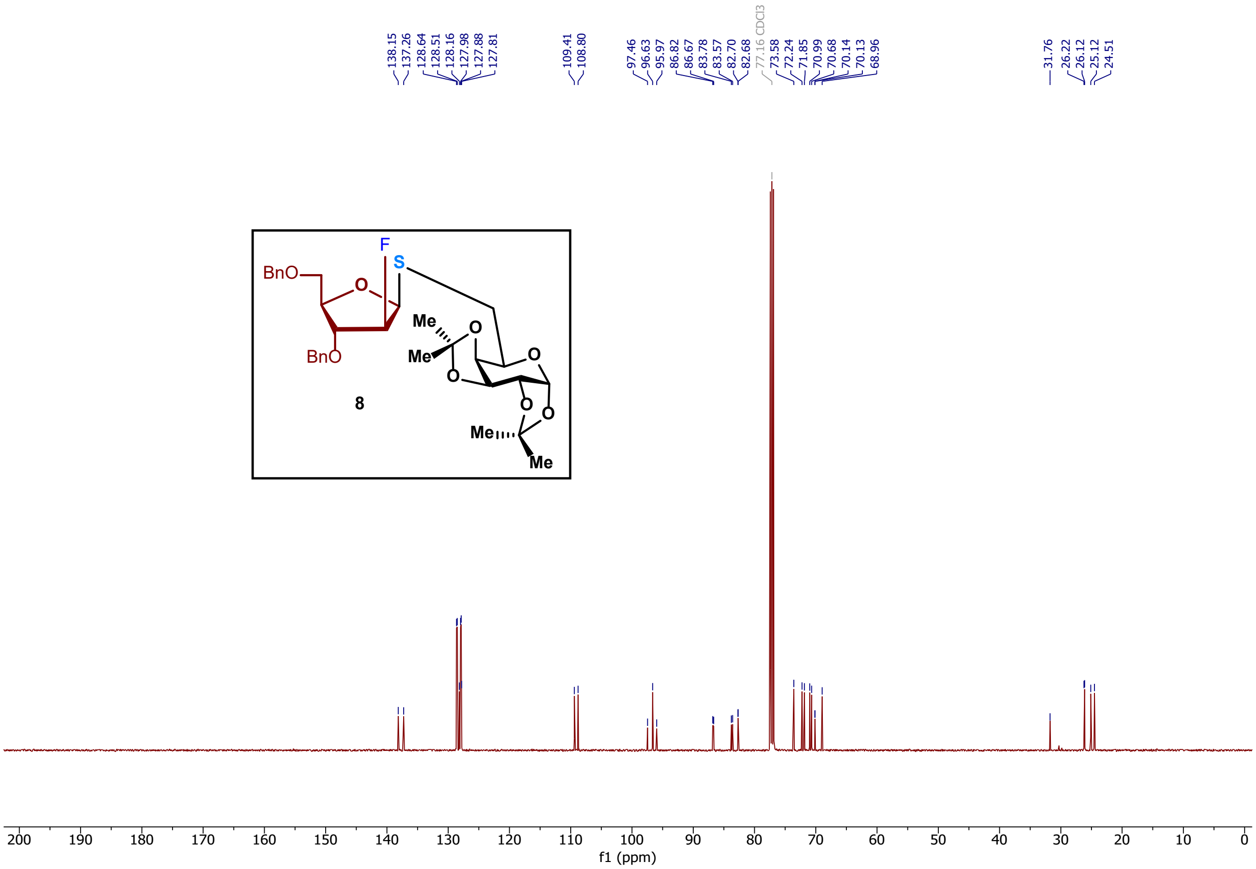
138.15
137.26
128.64
128.51
128.16
127.98
127.88
127.81
109.41
108.80
97.46
96.63
95.97
86.82
86.67
83.78
83.57
82.70
82.68
77.16 CDCl3
73.58
72.24
71.85
70.99
70.68
70.14
70.13
68.96
31.76
26.22
26.12
25.12
24.51
BnO
F
S
O
Me
Me
O
O
O
O
O
BnO
8
Me
Me
f1 (ppm)

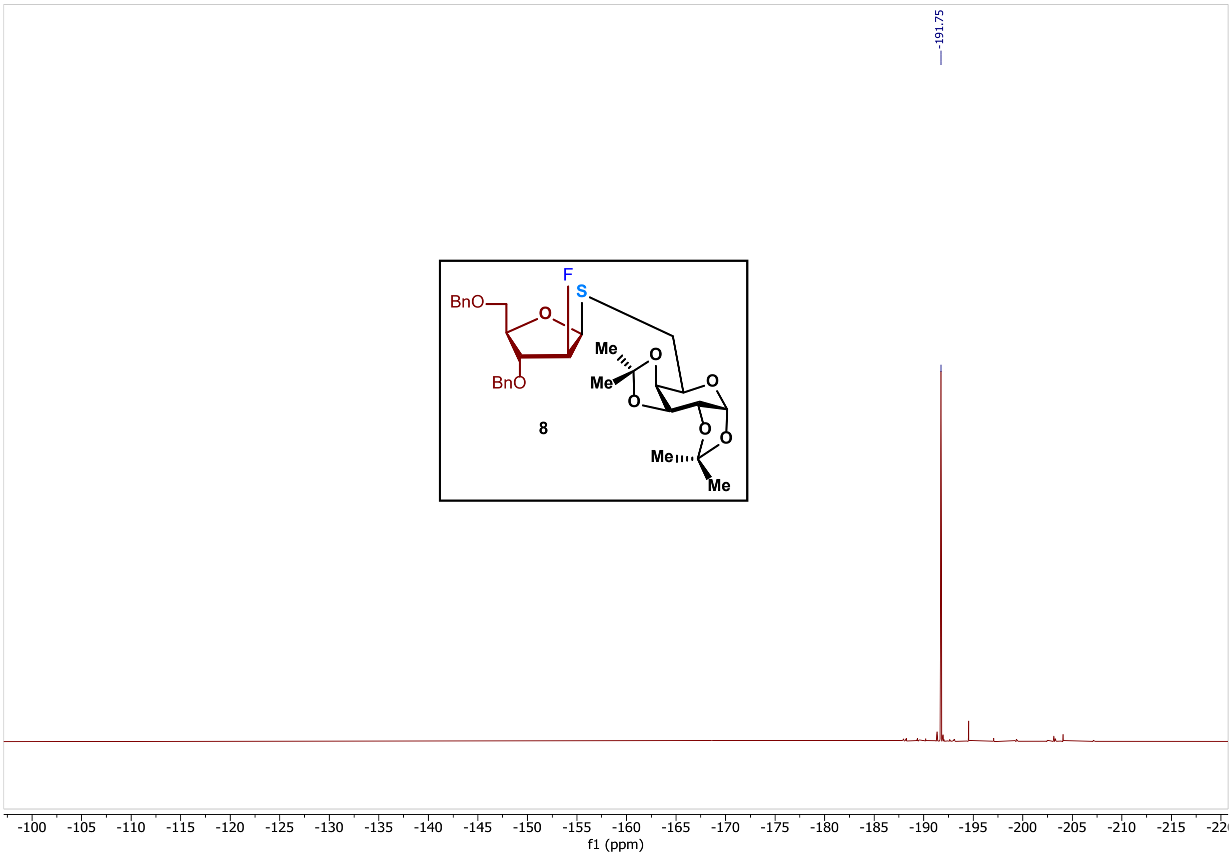
-191.75
BnO
F
S
O
Me
Me
O
O
O
O
O
BnO
8
Me
Me
f1 (ppm)

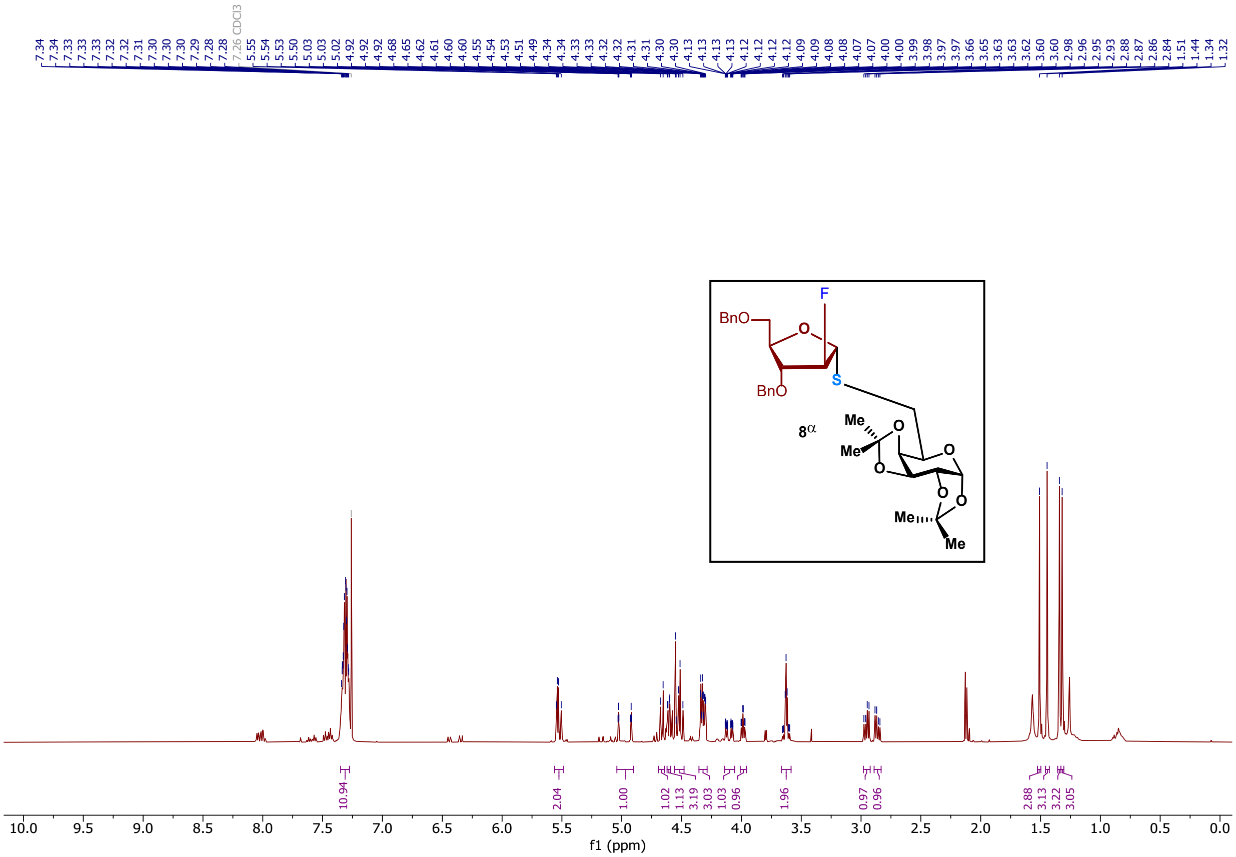
BnO
F
O
BnO
S
8α
Me
O
Me
O
O
O
O
Me
Me
f1 (ppm)

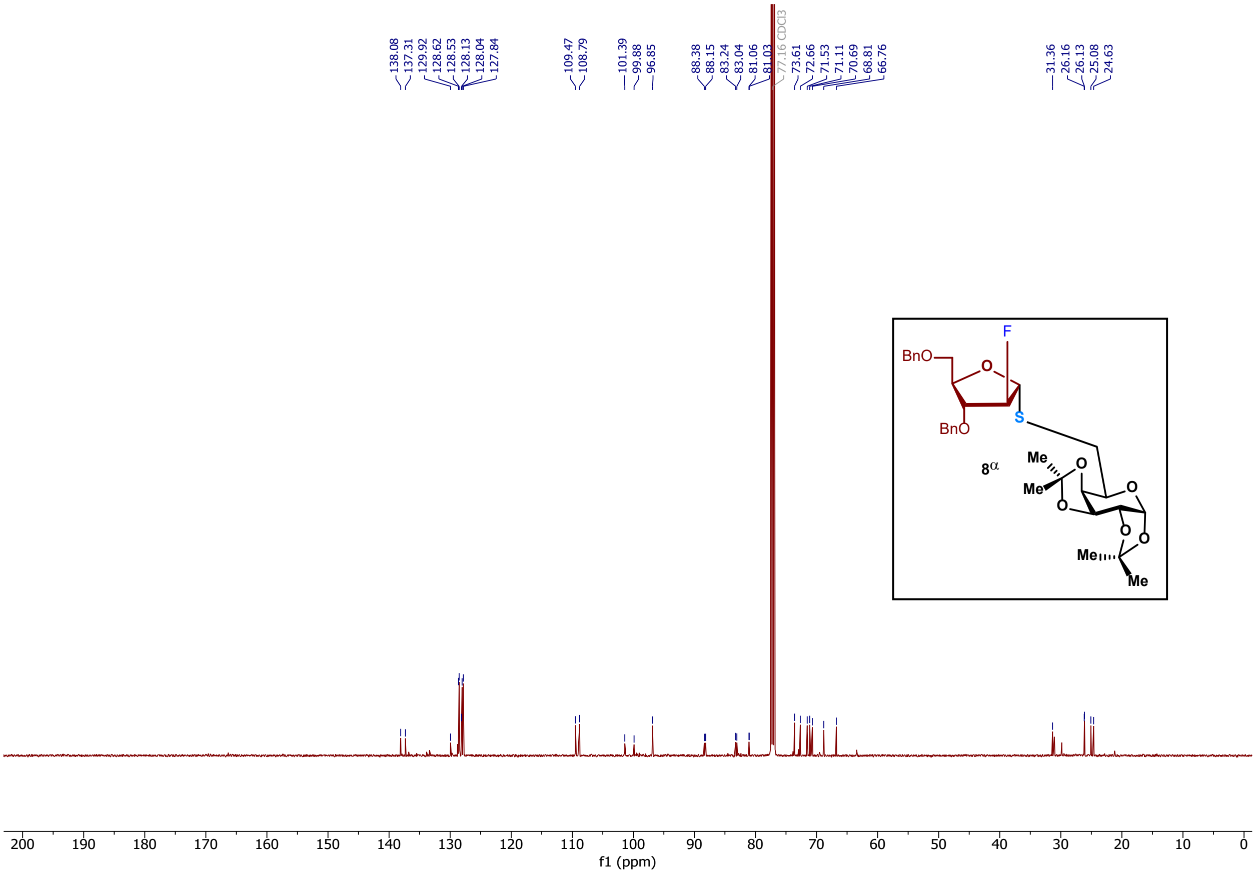
138.08
137.31
129.92
128.62
128.53
128.13
128.04
127.84
109.47
108.79
101.39
99.88
96.85
88.38
88.15
83.24
83.04
81.06
81.03
77.16 CDCl3
73.61
72.66
71.53
71.11
70.69
68.81
66.76
31.36
26.16
26.13
25.08
24.63
BnO
O
F
S
BnO
8α
Me
Me
O
O
O
O
O
Me
Me
200
190
180
170
160
150
140
130
120
110
100
90
80
70
60
50
40
30
20
10
0
f1 (ppm)

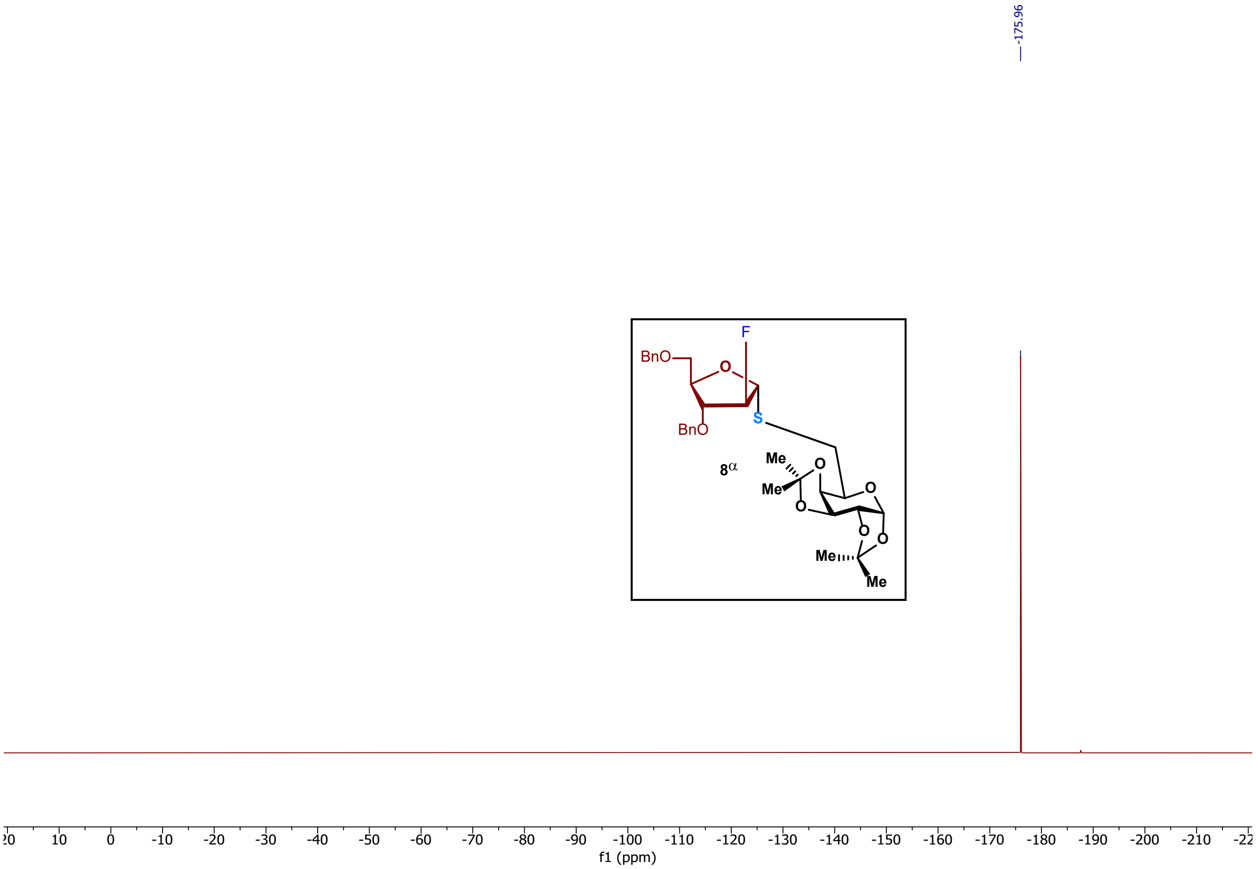

-175.96
F
BnO
O
BnO
S
8α
Me
Me
O
O
O
O
O
Me
Me
f1 (ppm)

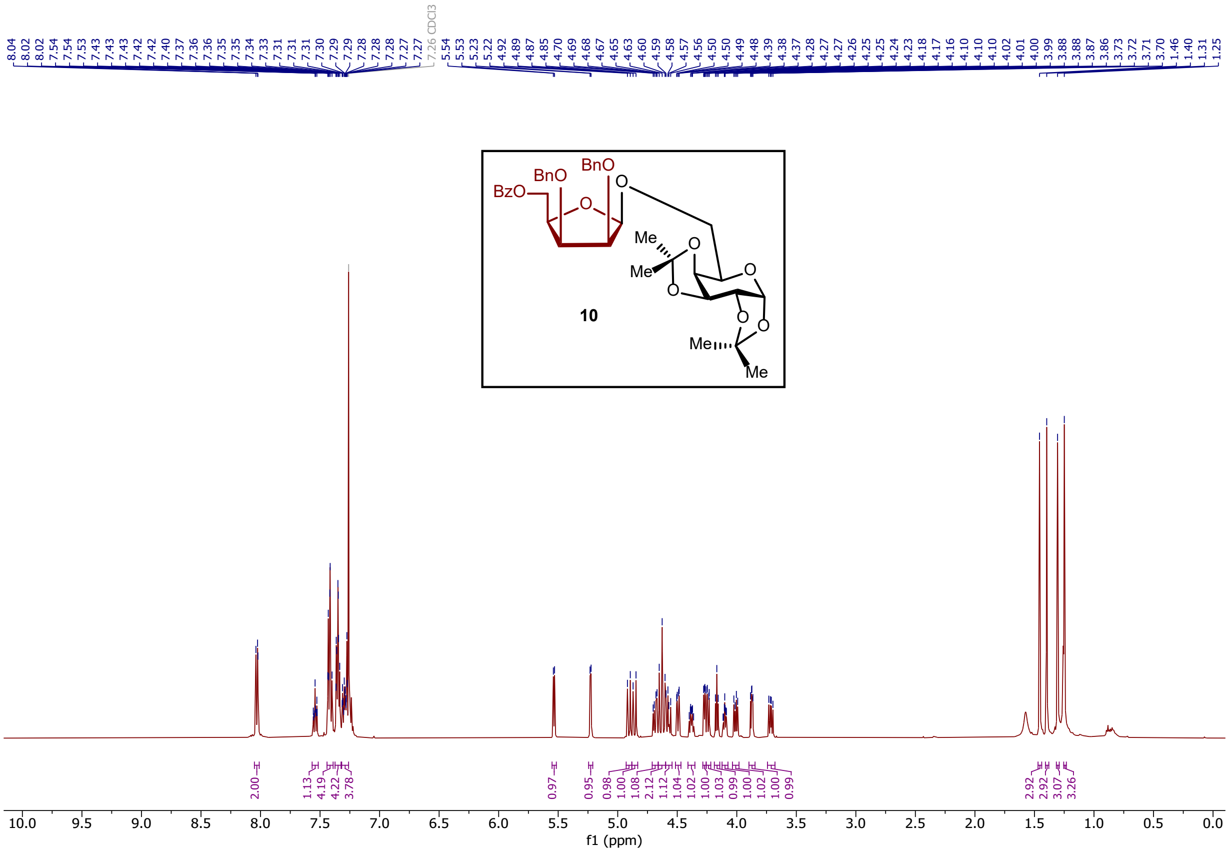

7.26 CDCl3
BzO
BnO
BnO
O
O
10
Me
Me
O
O
O
O
O
Me
Me
2.00
1.13
4.19
4.22
3.78
0.97
0.95
0.98
1.00
1.08
2.12
1.12
1.04
1.02
1.00
1.03
0.99
1.00
1.02
1.00
0.99
2.92
2.92
3.07
3.26
f1 (ppm)

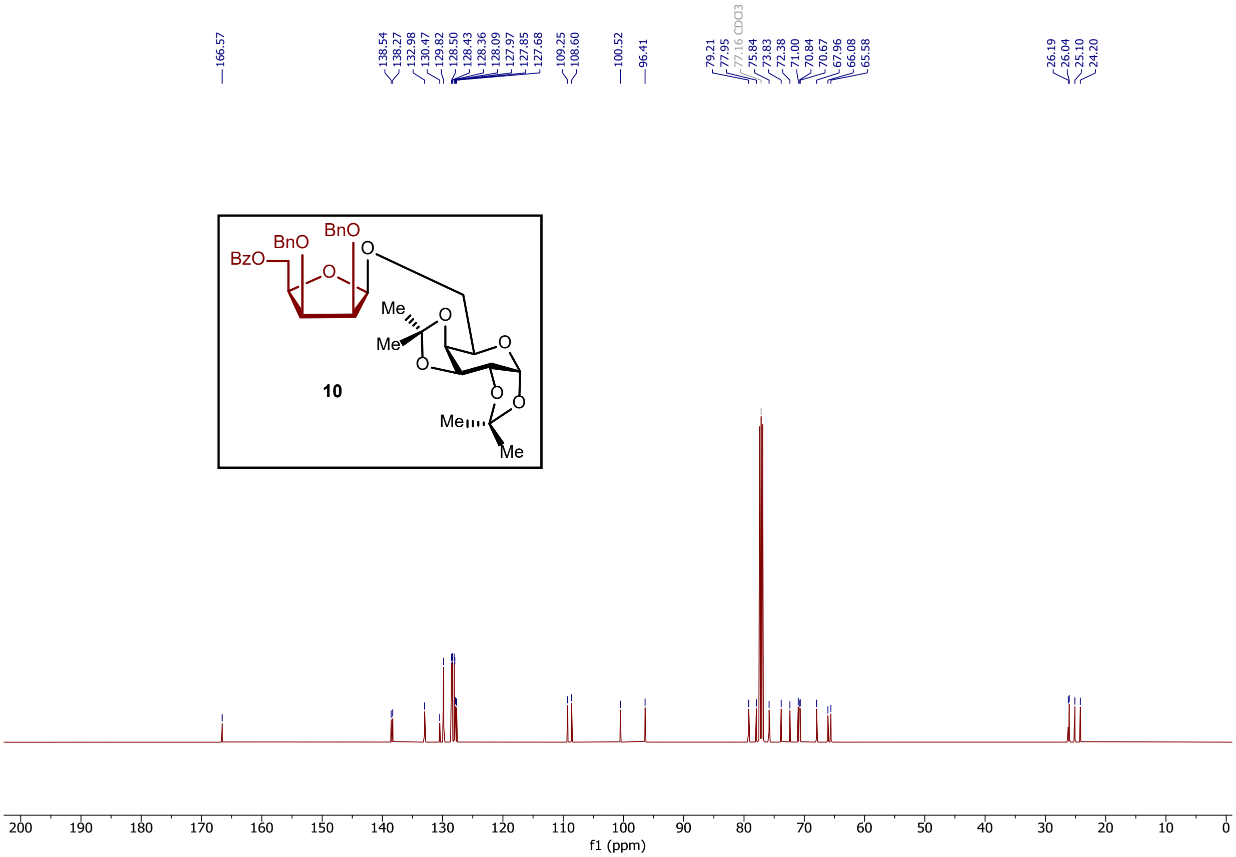
166.57
138.54
138.27
132.98
130.47
129.82
128.50
128.43
128.36
128.09
127.97
127.85
127.68
109.25
108.60
100.52
96.41
79.21
77.95
77.16 CDCl3
75.84
73.83
72.38
71.00
70.84
70.67
67.96
66.08
65.58
26.19
26.04
25.10
24.20
BzO
BnO
BnO
O
O
Me
Me
O
O
O
O
O
Me
Me
10
200 190 180 170 160 150 140 130 120 110 100 90 80 70 60 50 40 30 20 10 0
f1 (ppm)

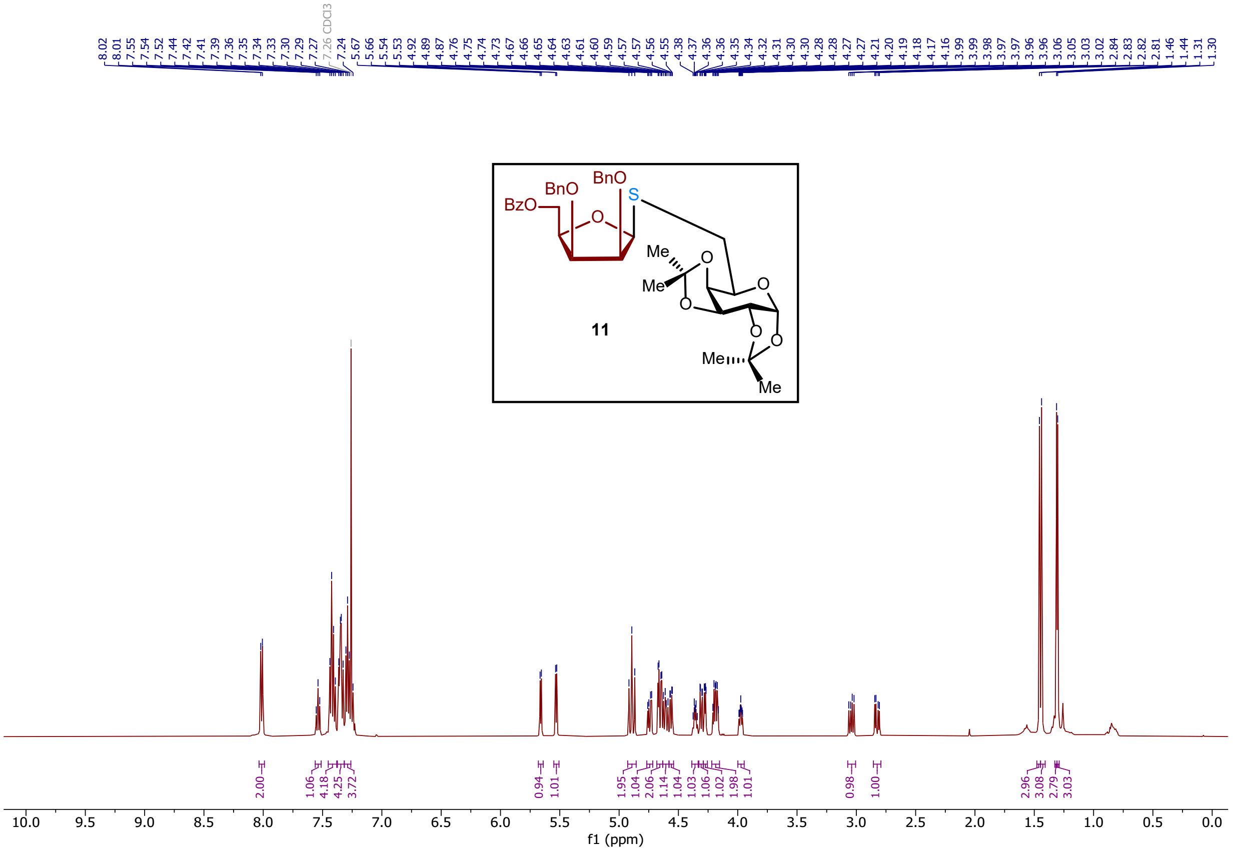
8.02
8.01
7.55
7.54
7.52
7.44
7.42
7.41
7.39
7.36
7.35
7.34
7.33
7.30
7.29
7.27
7.26 CDCl3
7.24
5.67
5.66
5.54
5.53
4.92
4.89
4.87
4.76
4.75
4.74
4.73
4.67
4.66
4.65
4.64
4.63
4.61
4.60
4.59
4.57
4.57
4.56
4.55
4.38
4.37
4.36
4.36
4.35
4.34
4.32
4.31
4.30
4.30
4.28
4.28
4.27
4.27
4.21
4.20
4.19
4.18
4.17
4.16
3.99
3.99
3.98
3.97
3.97
3.96
3.96
3.06
3.05
3.03
3.02
2.84
2.83
2.82
2.81
1.46
1.44
1.31
1.30
BzO
BnO
BnO
S
O
Me
Me
O
O
O
O
O
Me
Me
11
2.00
1.06
4.18
4.25
3.72
0.94
1.01
1.95
1.04
2.06
1.14
1.04
1.03
1.06
1.02
1.98
1.01
0.98
1.00
2.96
3.08
2.79
3.03
10.0 9.5 9.0 8.5 8.0 7.5 7.0 6.5 6.0 5.5 5.0 4.5 4.0 3.5 3.0 2.5 2.0 1.5 1.0 0.5 0.0
f1 (ppm)

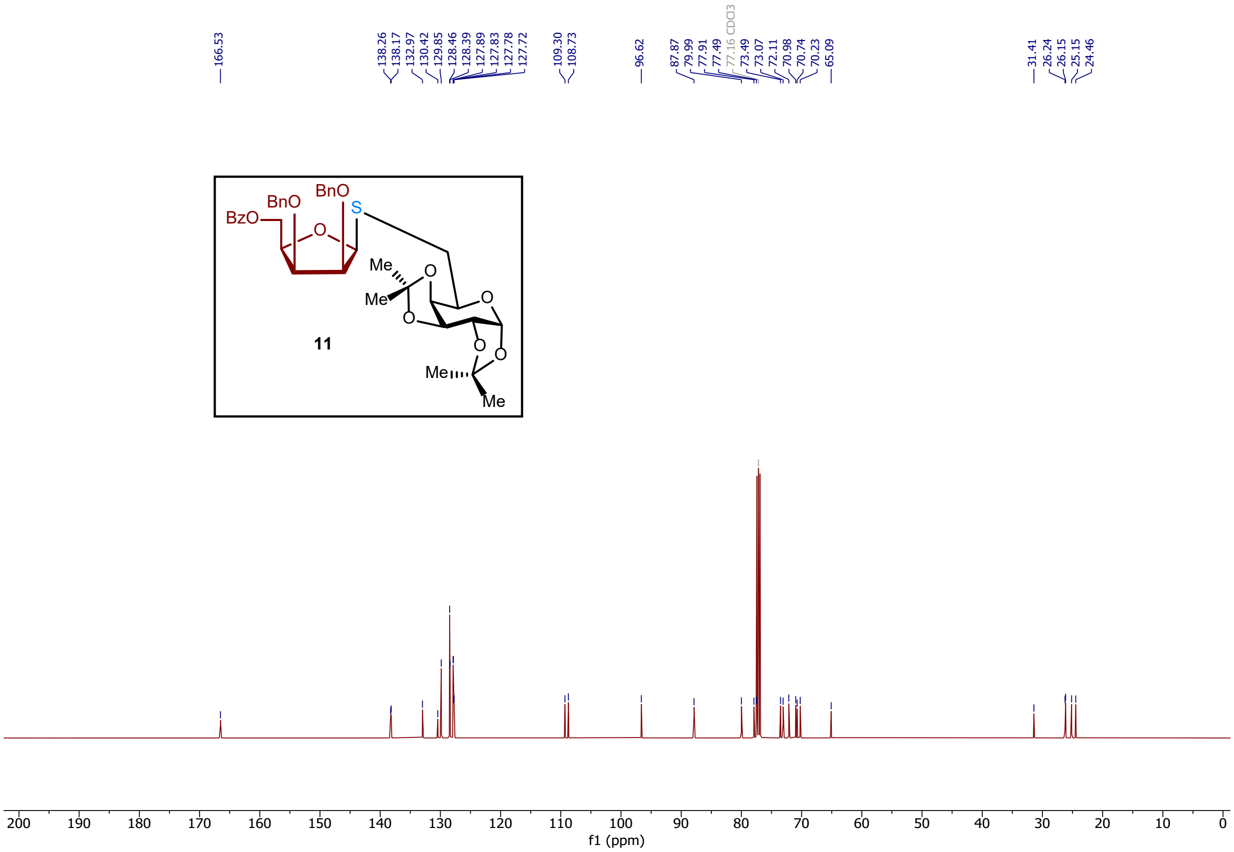

166.53
138.26
138.17
132.97
130.42
129.85
128.46
128.39
127.89
127.83
127.78
127.72
109.30
108.73
96.62
87.87
79.99
77.91
77.49
77.16 CDCl3
73.49
73.07
72.11
70.98
70.74
70.23
65.09
31.41
26.24
26.15
25.15
24.46
BzO
BnO
BnO
S
O
Me
Me
O
O
O
O
O
Me
Me
11
f1 (ppm)

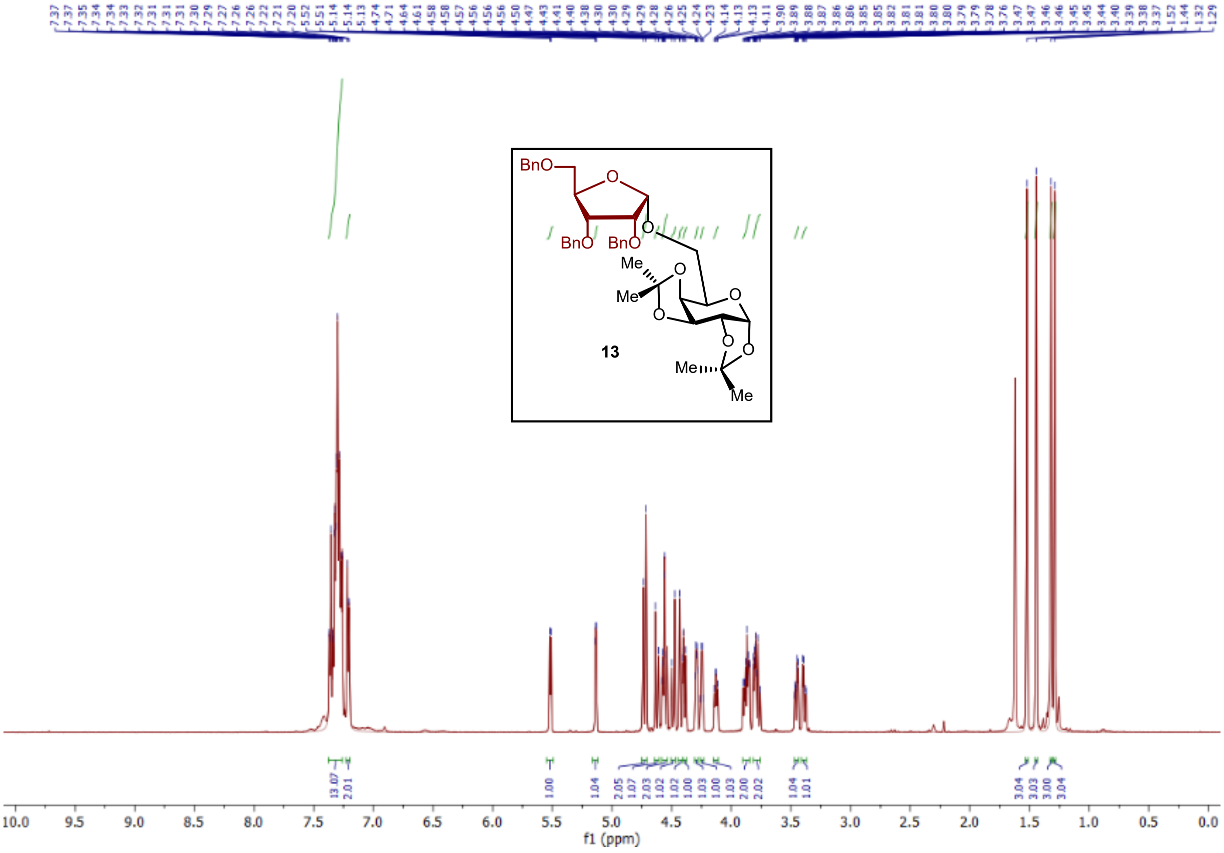

BnO
O
BnO
BnO
O
Me
Me
O
O
O
O
O
Me
Me
13
13.07
2.01
1.00
1.04
2.05
1.07
2.03
1.02
1.02
1.00
1.03
1.00
1.03
2.00
2.02
1.04
1.01
3.04
3.03
3.00
3.04
f1 (ppm)

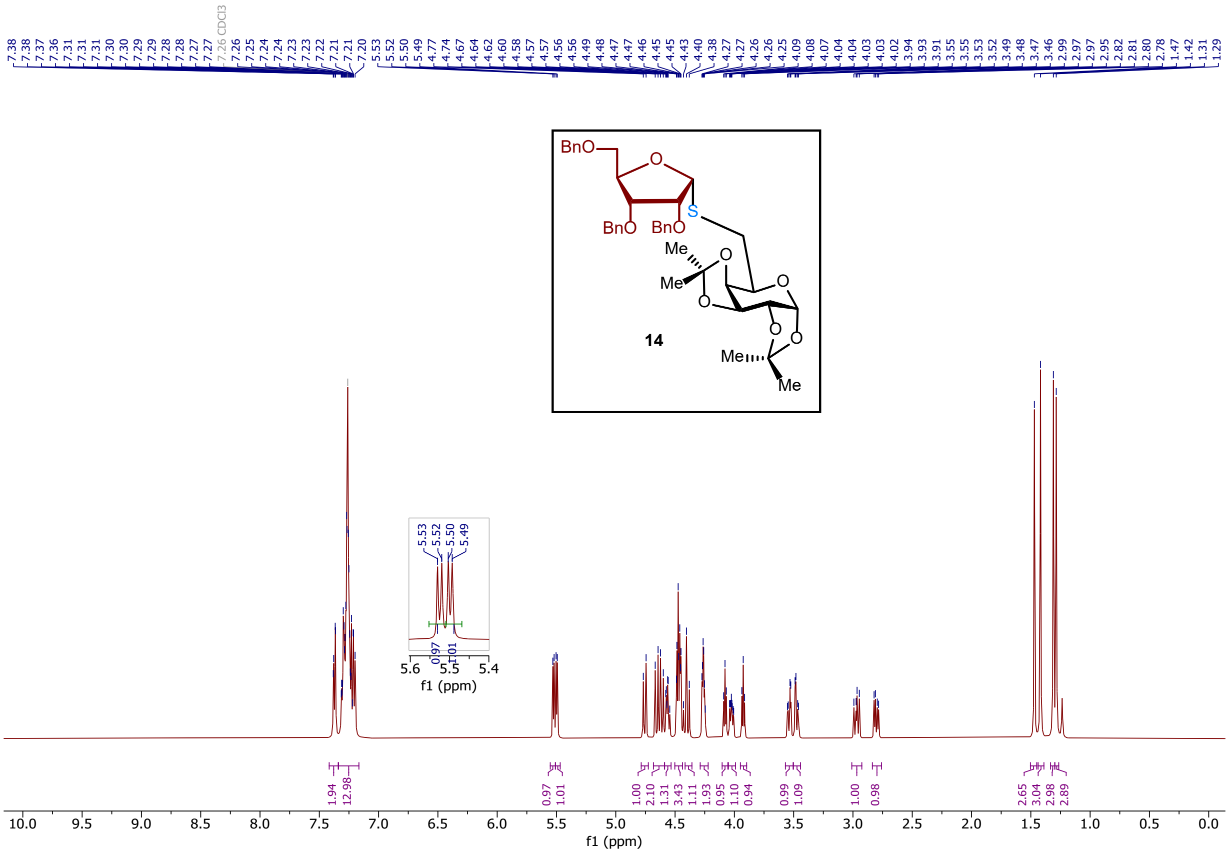

BnO
O
S
BnO
BnO
Me
Me
O
O
O
O
O
Me
Me
14
5.53
5.52
5.50
5.49
f1 (ppm)
f1 (ppm)

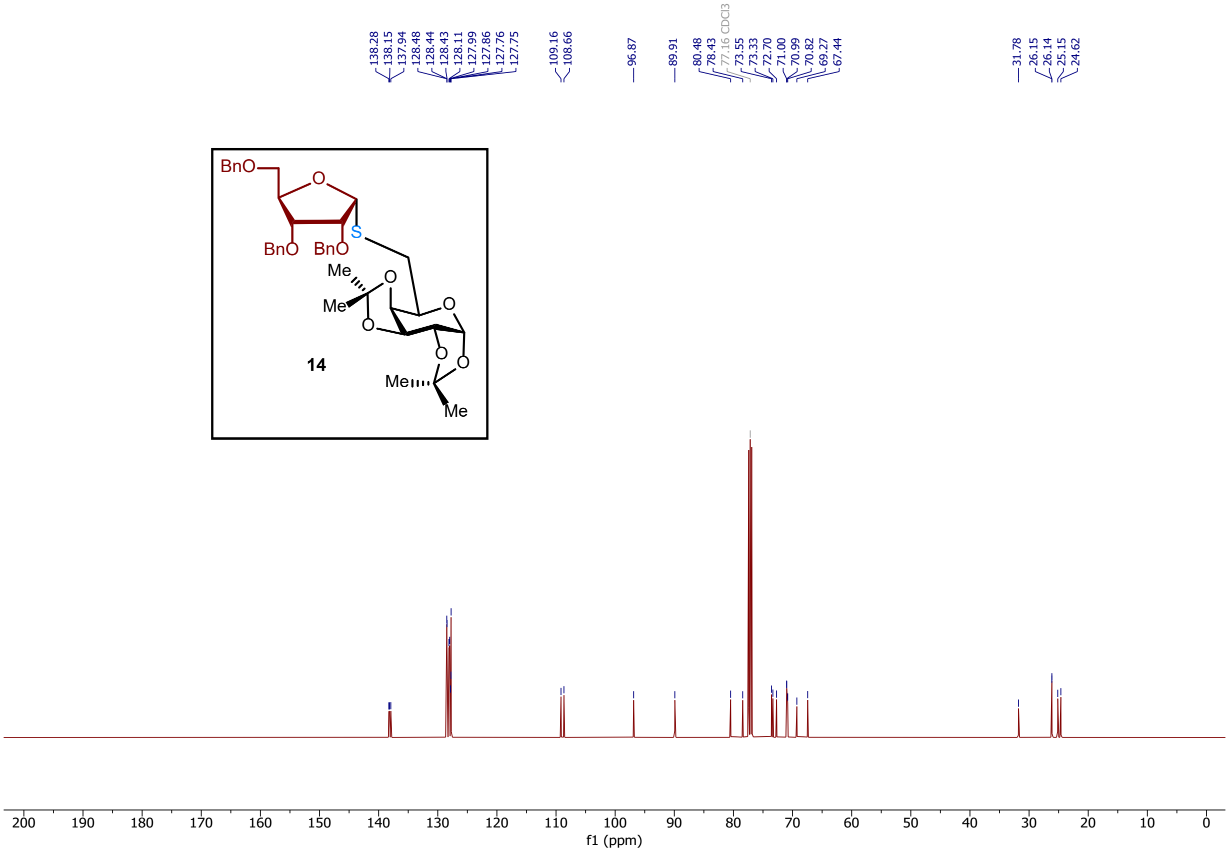

138.28
138.15
137.94
128.48
128.44
128.43
128.11
127.99
127.86
127.76
127.75
109.16
108.66
96.87
89.91
80.48
78.43
77.16 CDCl3
73.55
73.33
72.70
71.00
70.99
70.82
69.27
67.44
31.78
26.15
26.14
25.15
24.62
14
f1 (ppm)

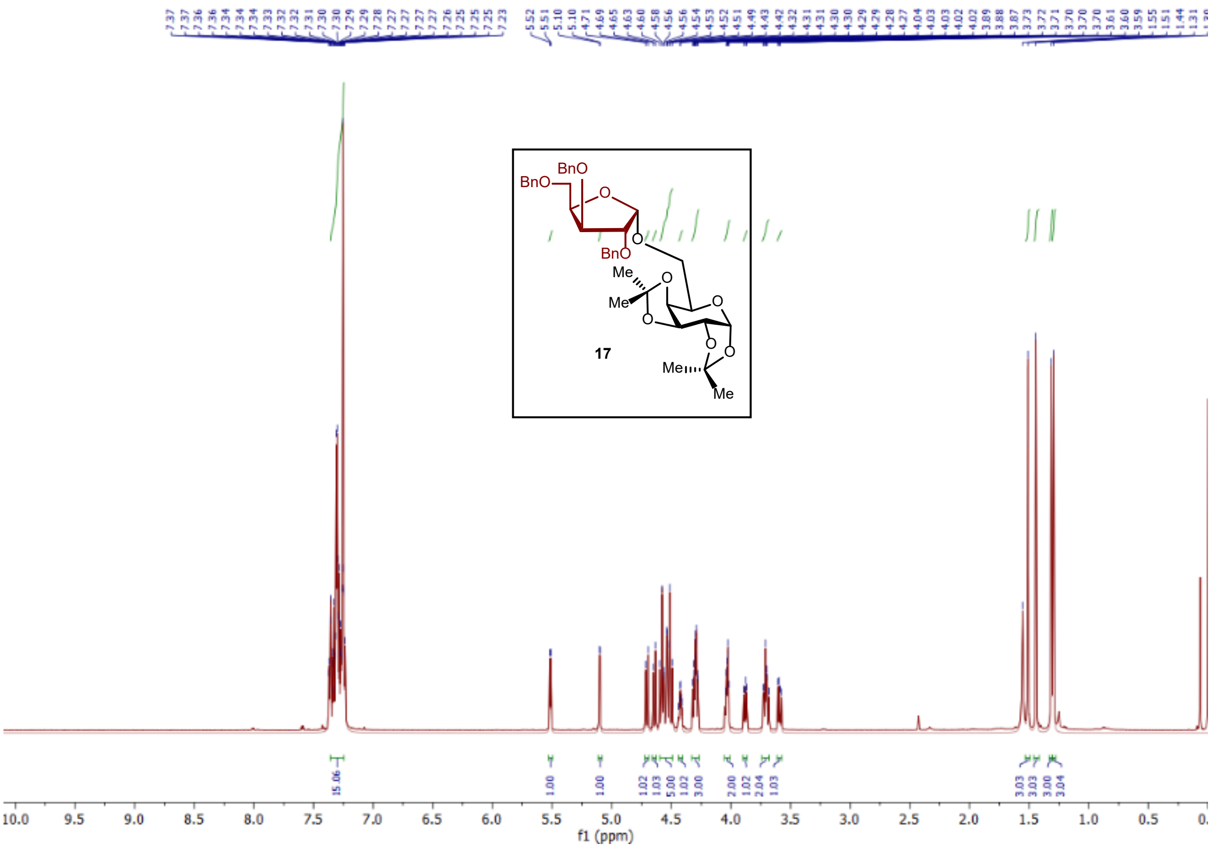
BnO
BnO
BnO
O
Me
Me
17
Me
Me
f1 (ppm)

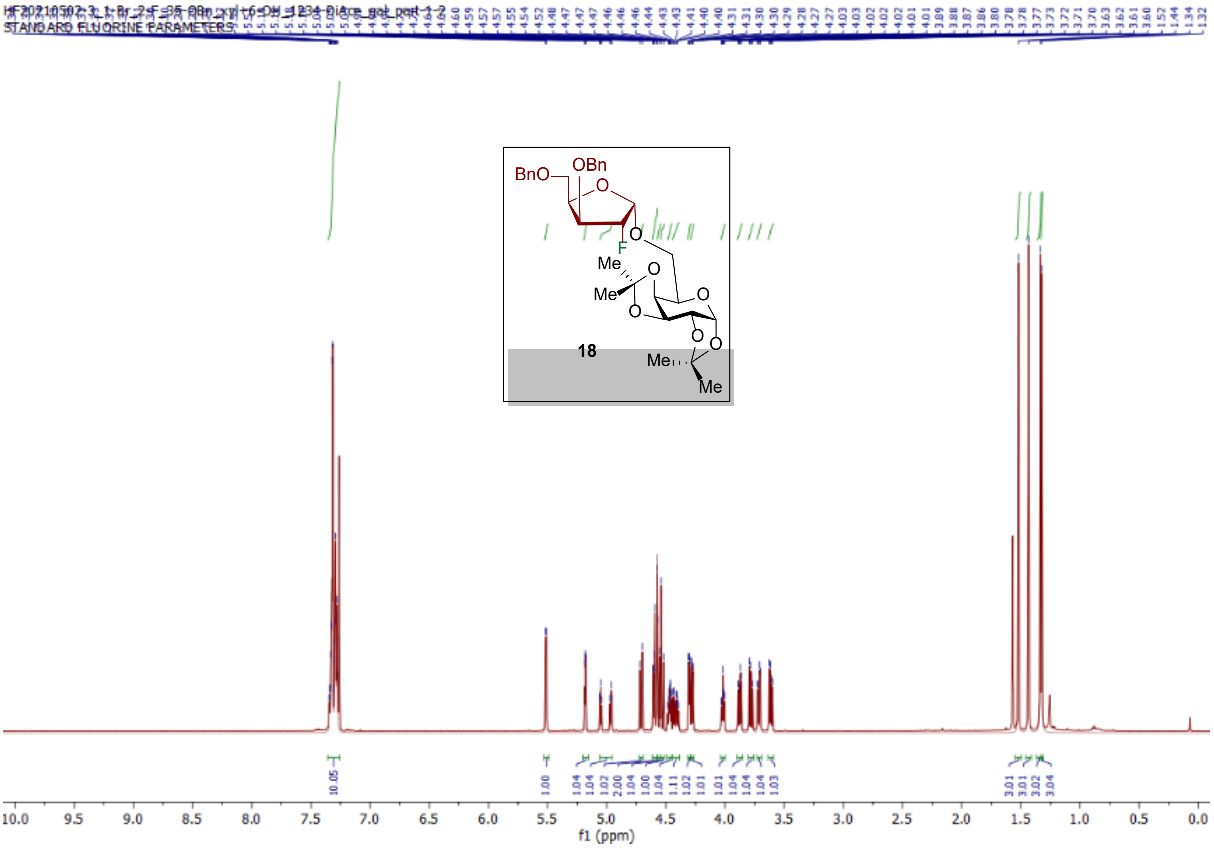
STANDARD FLUORINE PARAMETERS
BnO
OBn
O
F
Me
Me
18
Me
Me
f1 (ppm)

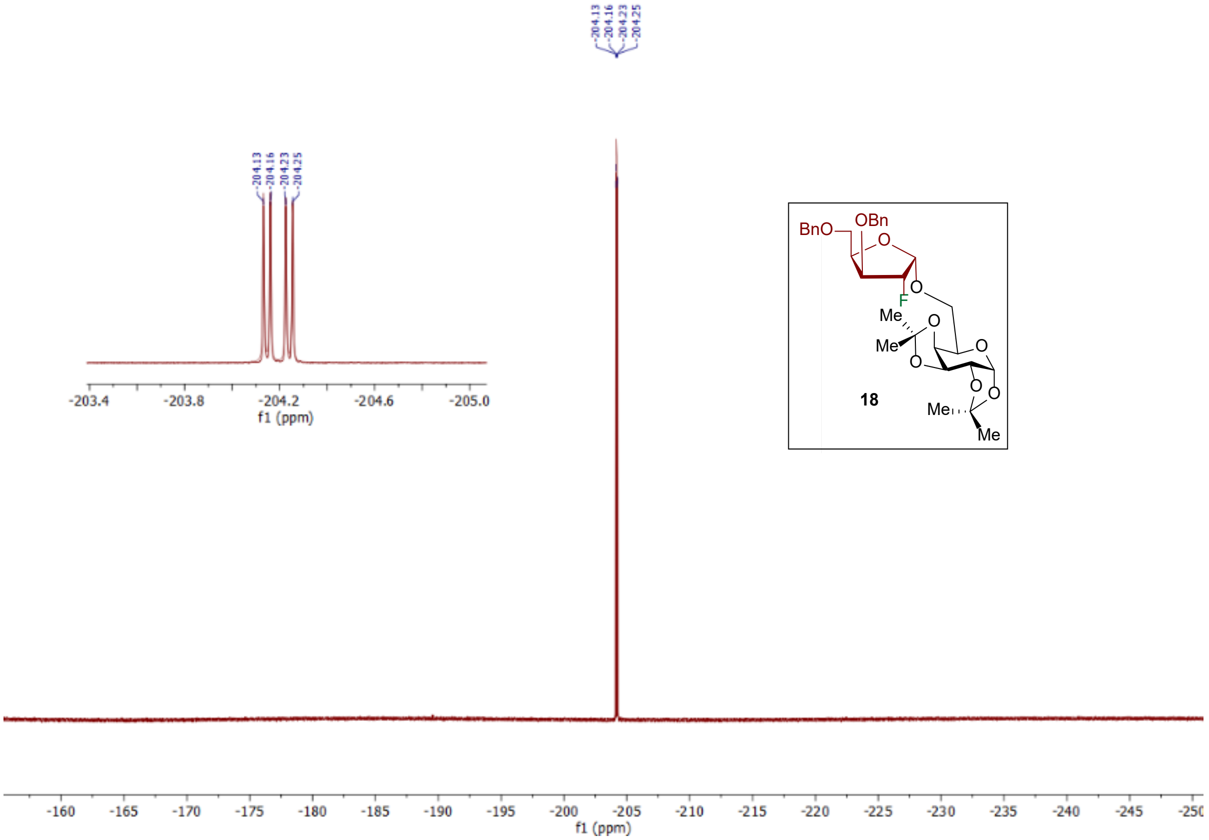
-204.13
-204.16
-204.23
-204.25
-203.4 -203.8 -204.2 -204.6 -205.0
f1 (ppm)
BnO
OBn
O
F
Me
18
-160 -165 -170 -175 -180 -185 -190 -195 -200 -205 -210 -215 -220 -225 -230 -235 -240 -245
f1 (ppm)

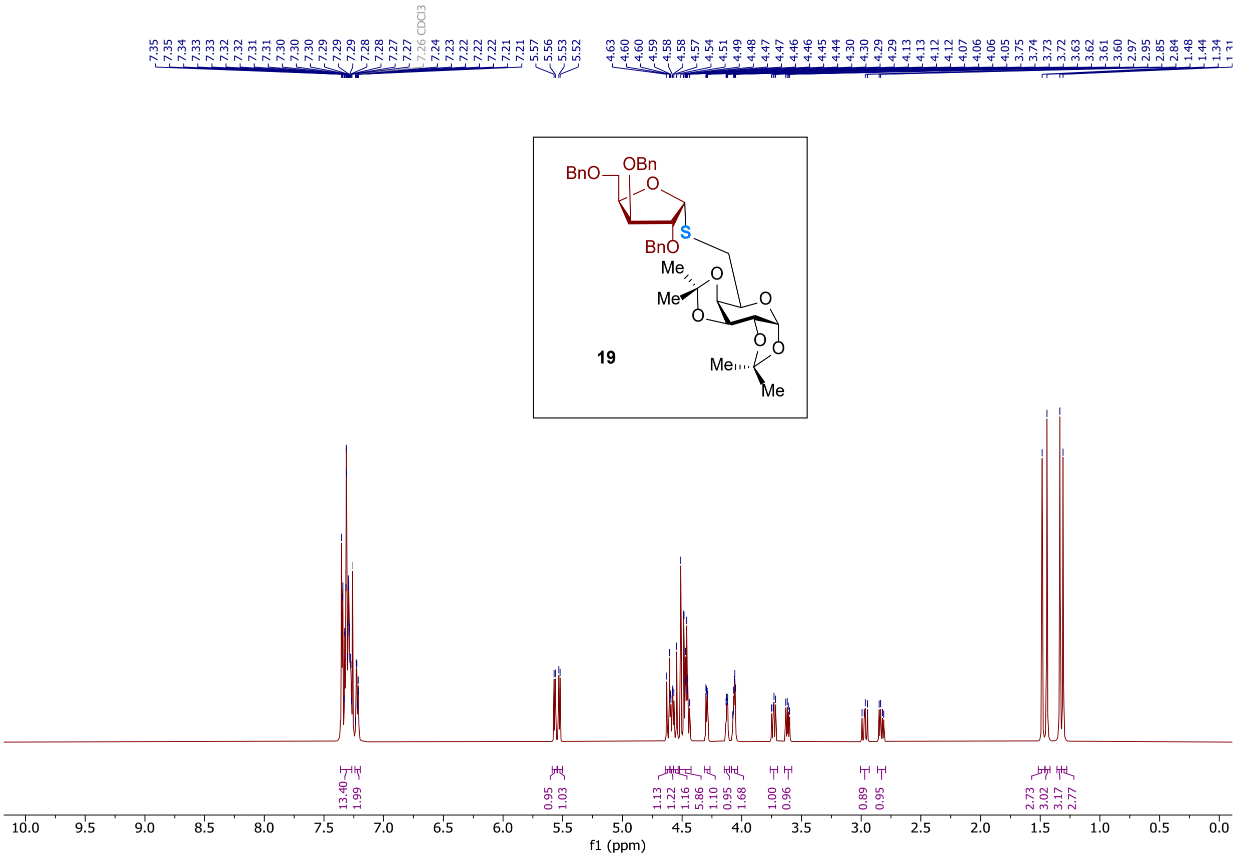
7.26 CDCl3
BnO
OBn
S
Me
19
13.40
1.99
0.95
1.03
1.13
1.22
1.16
5.86
1.10
0.95
1.68
1.00
0.96
0.89
0.95
2.73
3.02
3.17
2.77
10.0 9.5 9.0 8.5 8.0 7.5 7.0 6.5 6.0 5.5 5.0 4.5 4.0 3.5 3.0 2.5 2.0 1.5 1.0 0.5 0.0
f1 (ppm)

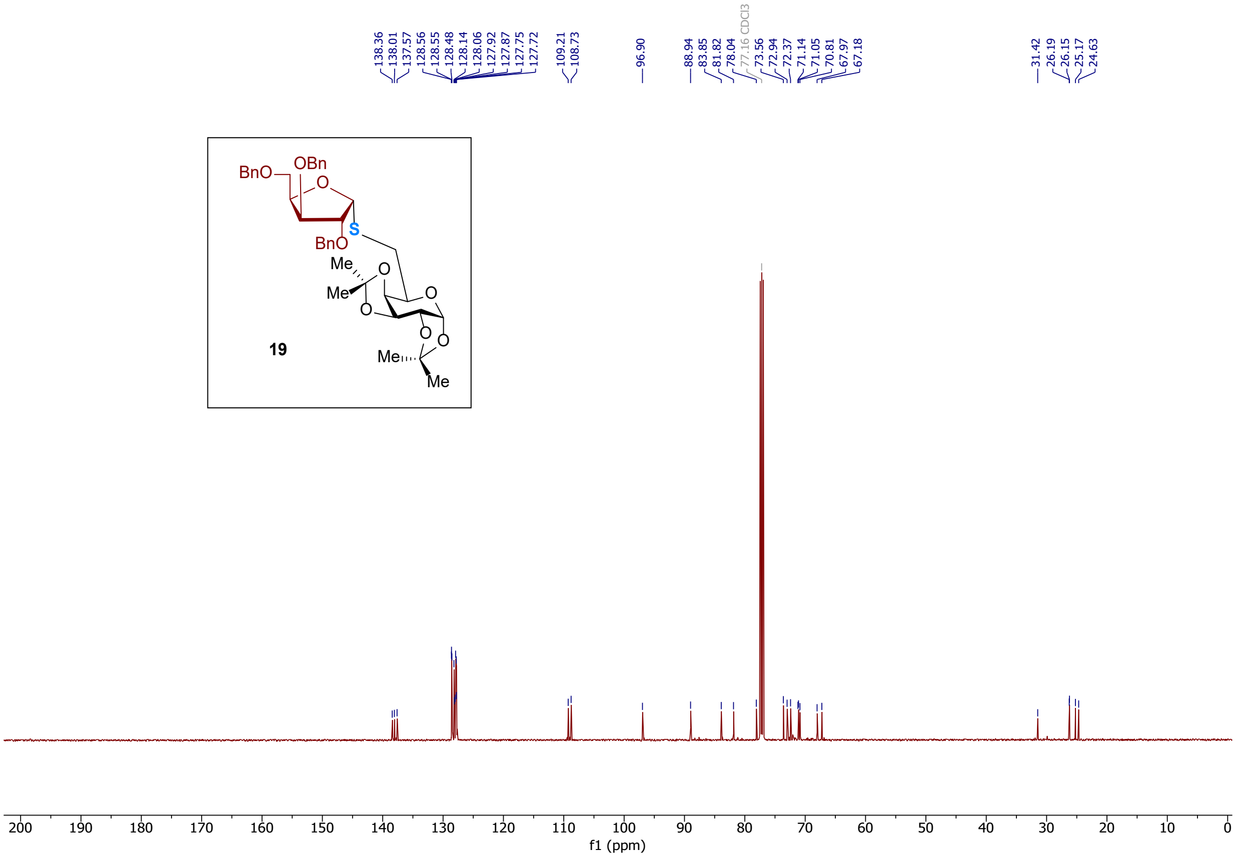
138.36
138.01
137.57
128.56
128.55
128.48
128.14
128.06
127.92
127.87
127.75
127.72
109.21
108.73
96.90
88.94
83.85
81.82
78.04
77.16 CDCl3
73.56
72.94
72.37
71.14
71.05
70.81
67.97
67.18
31.42
26.19
26.15
25.17
24.63
BnO
OBn
O
S
BnO
Me
Me
O
O
O
O
O
Me
Me
19
200 190 180 170 160 150 140 130 120 110 100 90 80 70 60 50 40 30 20 10 0
f1 (ppm)

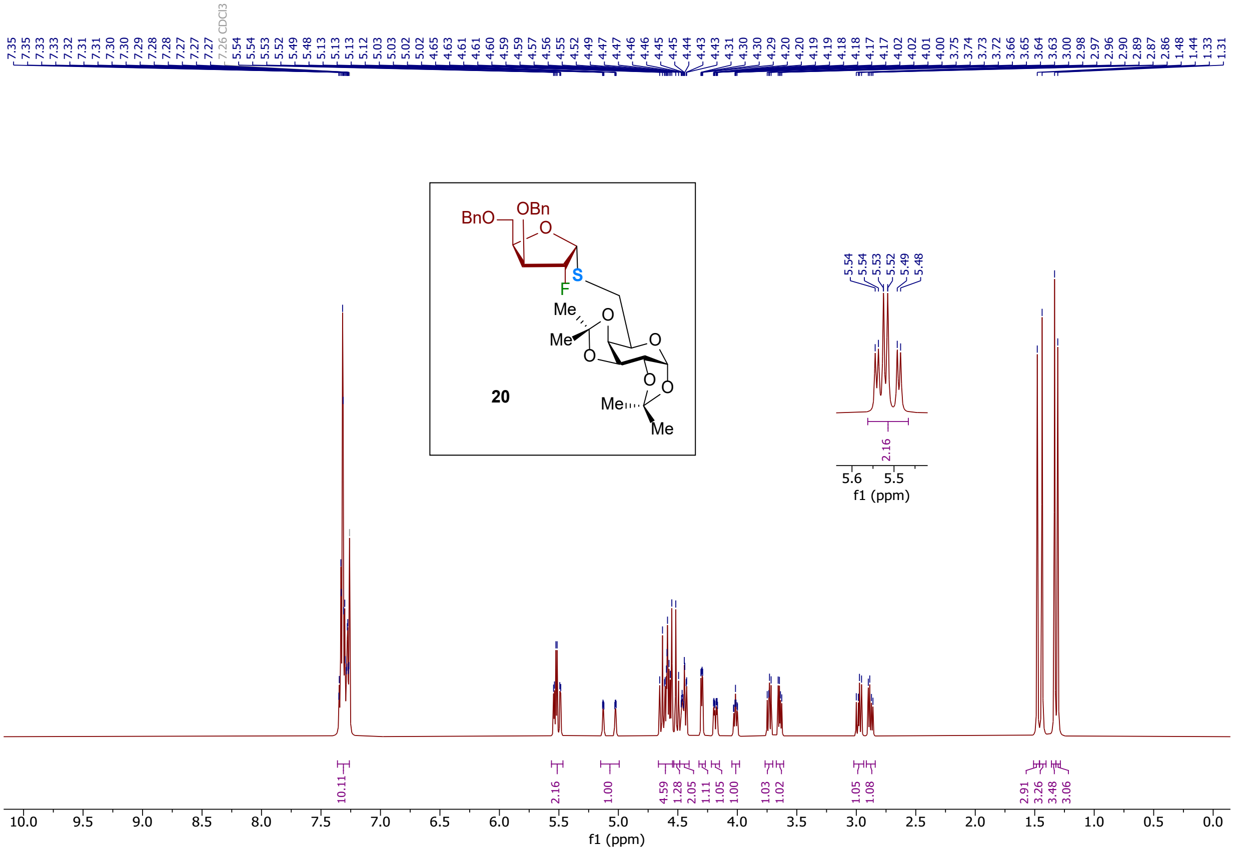
7.35
7.35
7.33
7.32
7.31
7.31
7.30
7.30
7.29
7.28
7.28
7.27
7.27
7.26 CDCl3
5.54
5.54
5.53
5.52
5.49
5.48
5.13
5.13
5.13
5.12
5.03
5.03
5.02
5.02
4.65
4.63
4.61
4.61
4.60
4.59
4.59
4.57
4.56
4.55
4.52
4.49
4.47
4.47
4.46
4.46
4.45
4.45
4.44
4.43
4.43
4.31
4.30
4.30
4.29
4.20
4.20
4.19
4.19
4.18
4.18
4.17
4.17
4.02
4.02
4.01
4.00
3.75
3.74
3.73
3.72
3.66
3.65
3.64
3.63
3.00
2.98
2.97
2.96
2.90
2.89
2.87
2.86
1.48
1.44
1.33
1.31
BnO
OBn
O
S
F
Me
Me
O
O
O
O
O
Me
Me
20
5.54
5.54
5.53
5.52
5.49
5.48
2.16
5.6 5.5
f1 (ppm)
10.11
2.16
1.00
4.59
1.28
2.05
1.11
1.05
1.00
1.03
1.02
1.05
1.08
2.91
3.26
3.48
3.06
10.0 9.5 9.0 8.5 8.0 7.5 7.0 6.5 6.0 5.5 5.0 4.5 4.0 3.5 3.0 2.5 2.0 1.5 1.0 0.5 0.0
f1 (ppm)

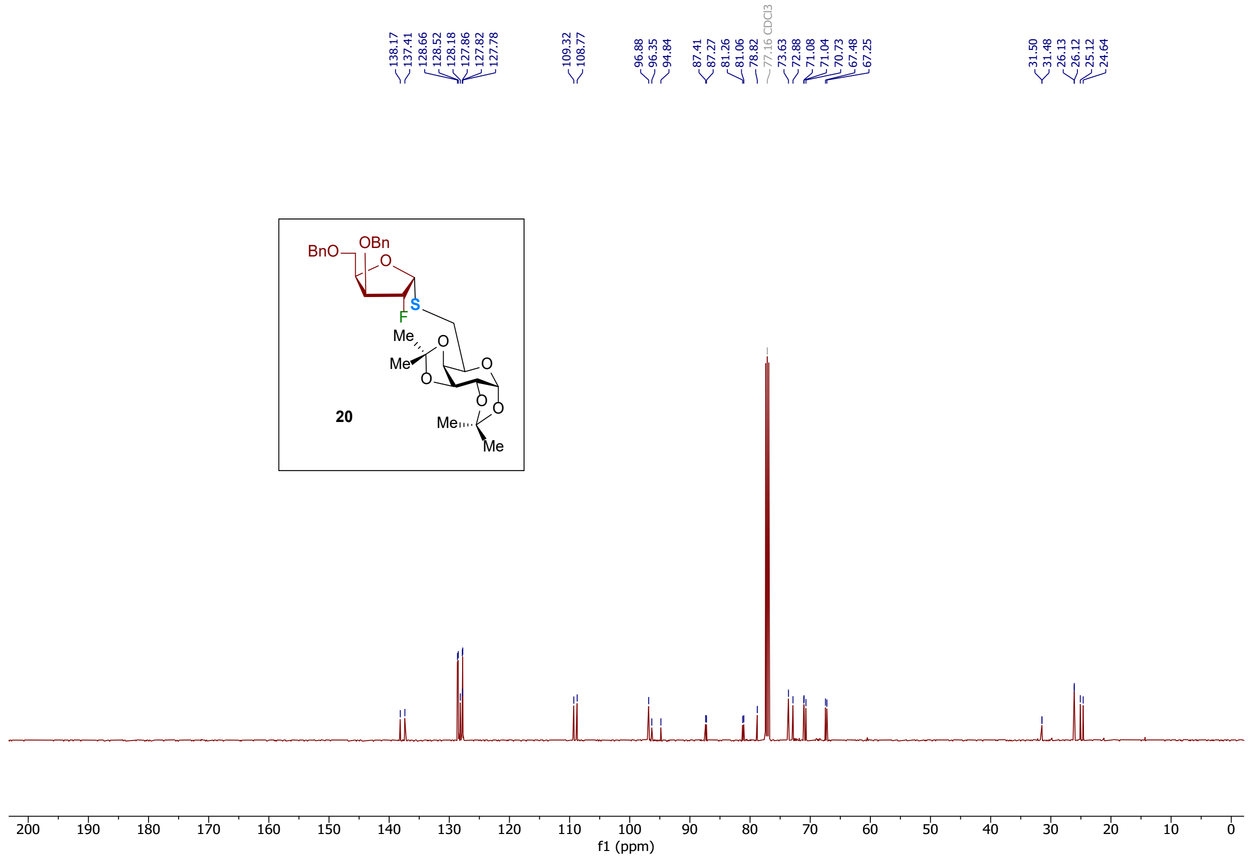
138.17
137.41
128.66
128.52
128.18
127.86
127.82
127.78
109.32
108.77
96.88
96.35
94.84
87.41
87.27
81.26
81.06
78.82
77.16 CDCl3
73.63
72.88
71.08
71.04
70.73
67.48
67.25
31.50
31.48
26.13
26.12
25.12
24.64
BnO
OBn
O
F
S
Me
Me
O
O
O
O
O
Me
Me
20
f1 (ppm)

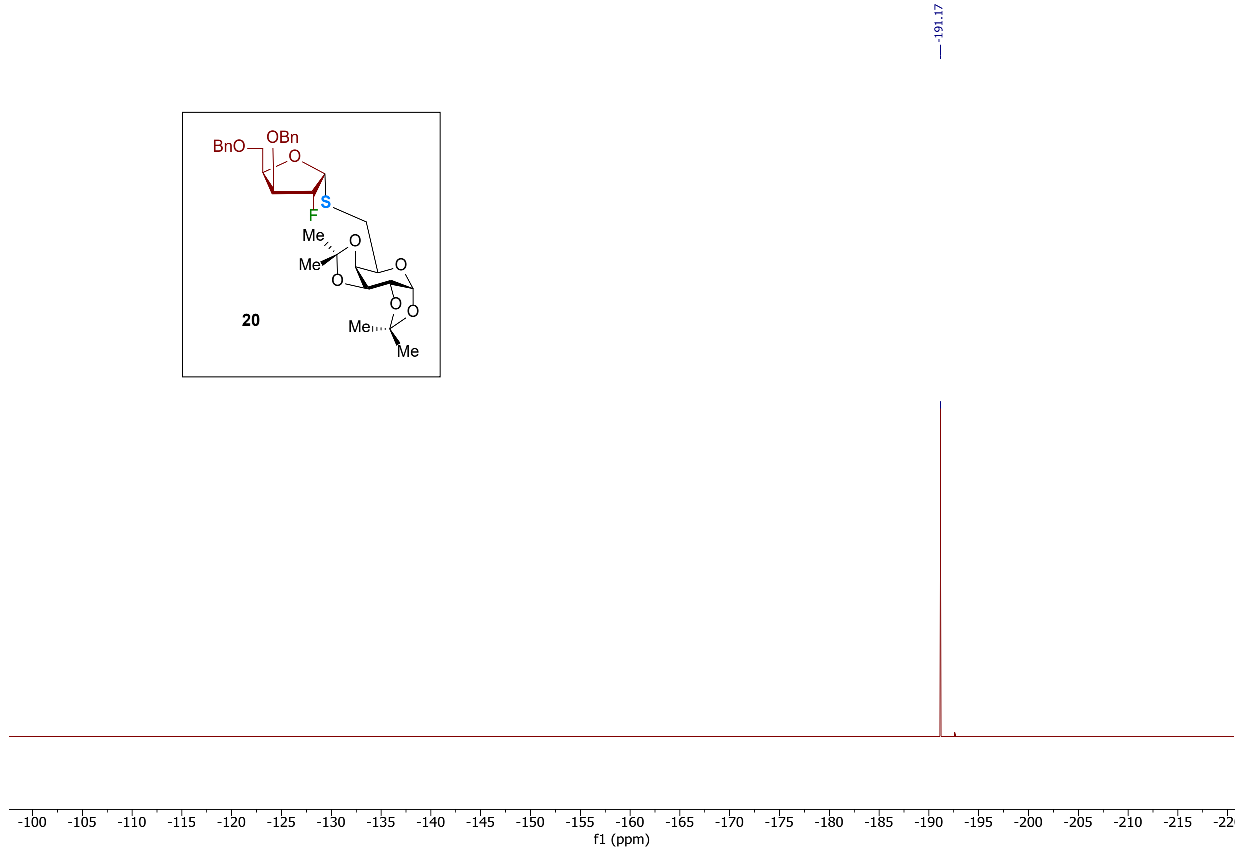
-191.17
BnO
OBn
O
F
S
Me
Me
O
O
O
O
O
Me
Me
20
f1 (ppm)

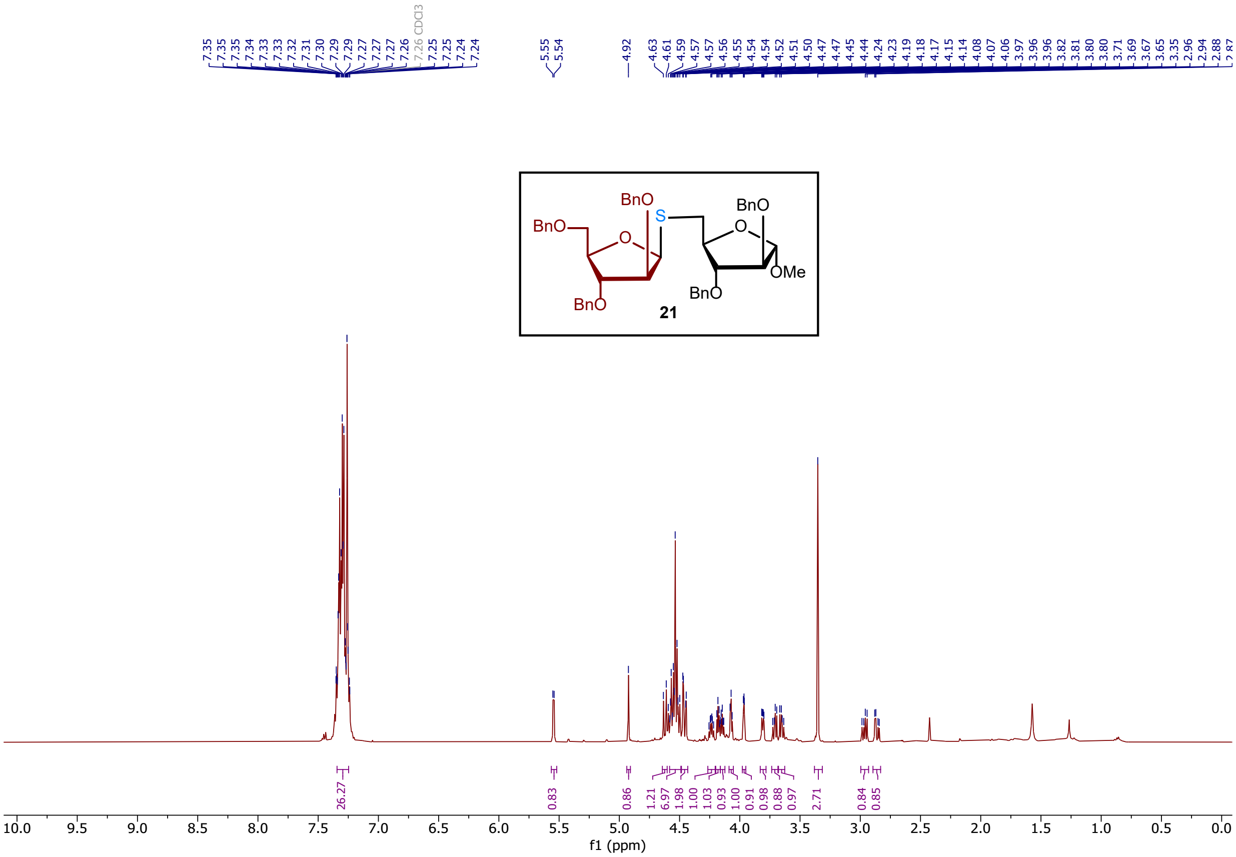

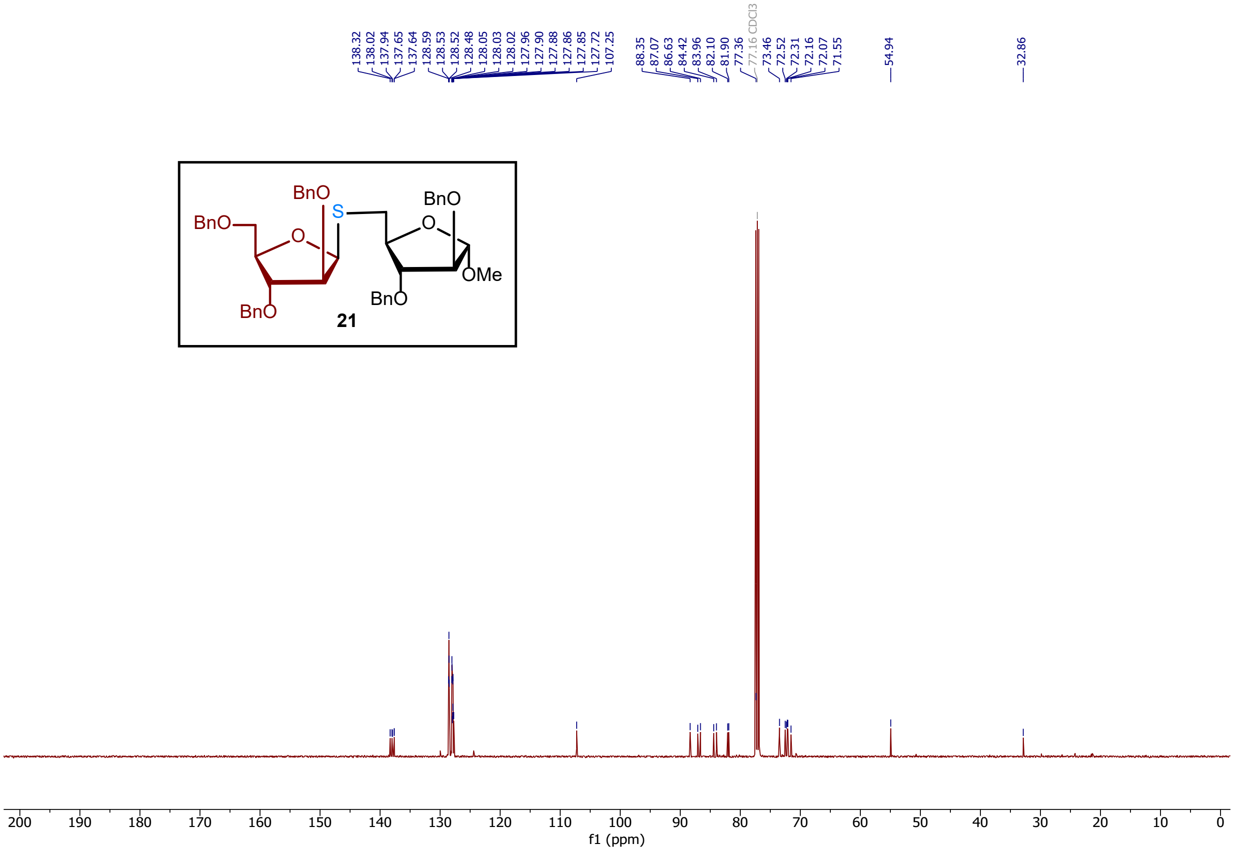

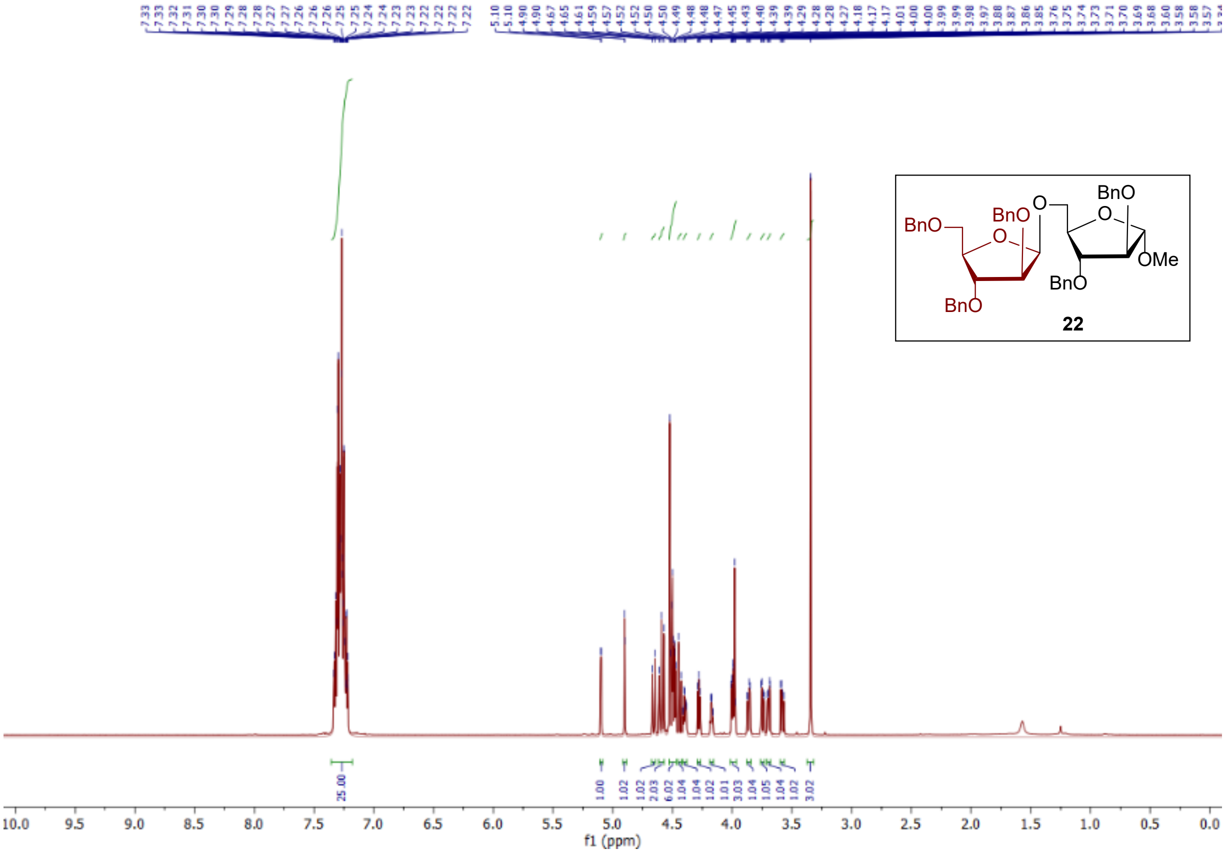
BnO
BnO
O
O
BnO
O
OMe
BnO
BnO
22
f1 (ppm)

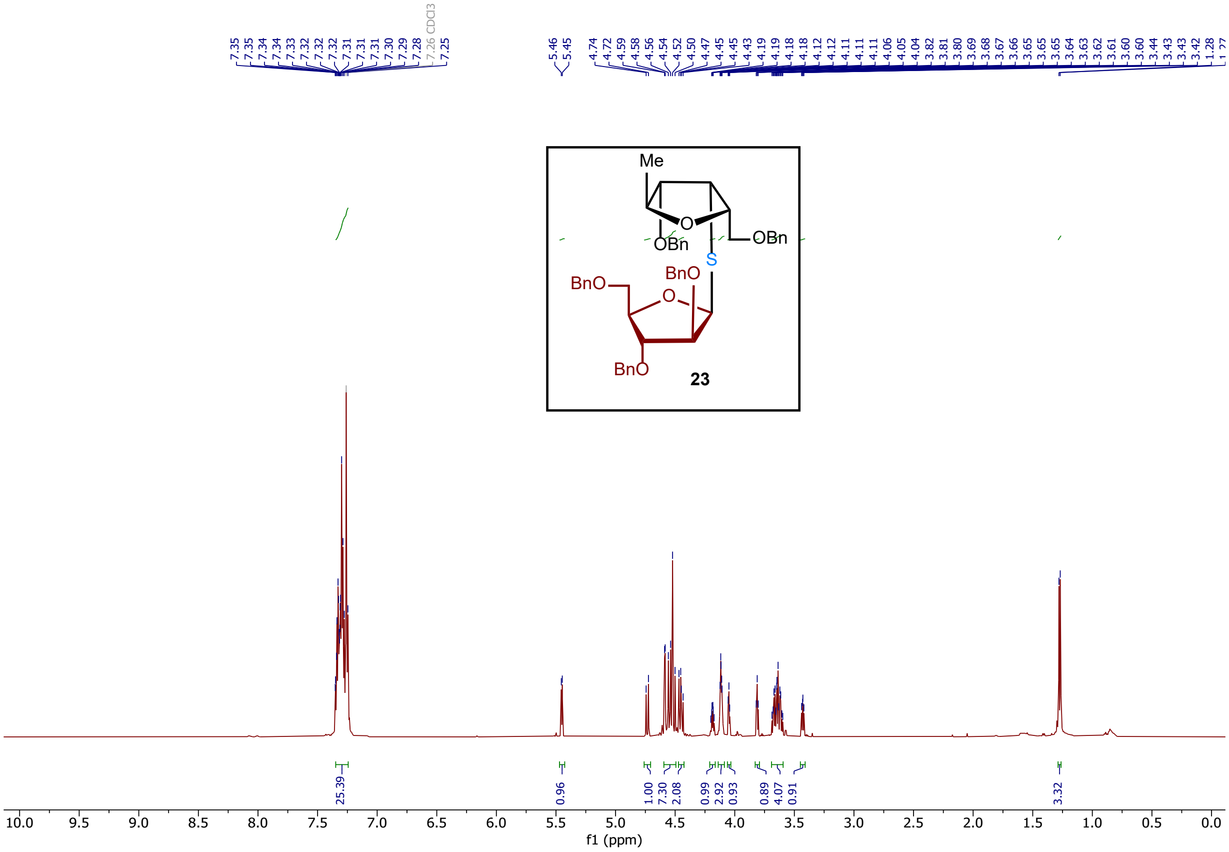
Me
O
OBn
OBn
S
BnO
BnO
O
BnO
23
f1 (ppm)

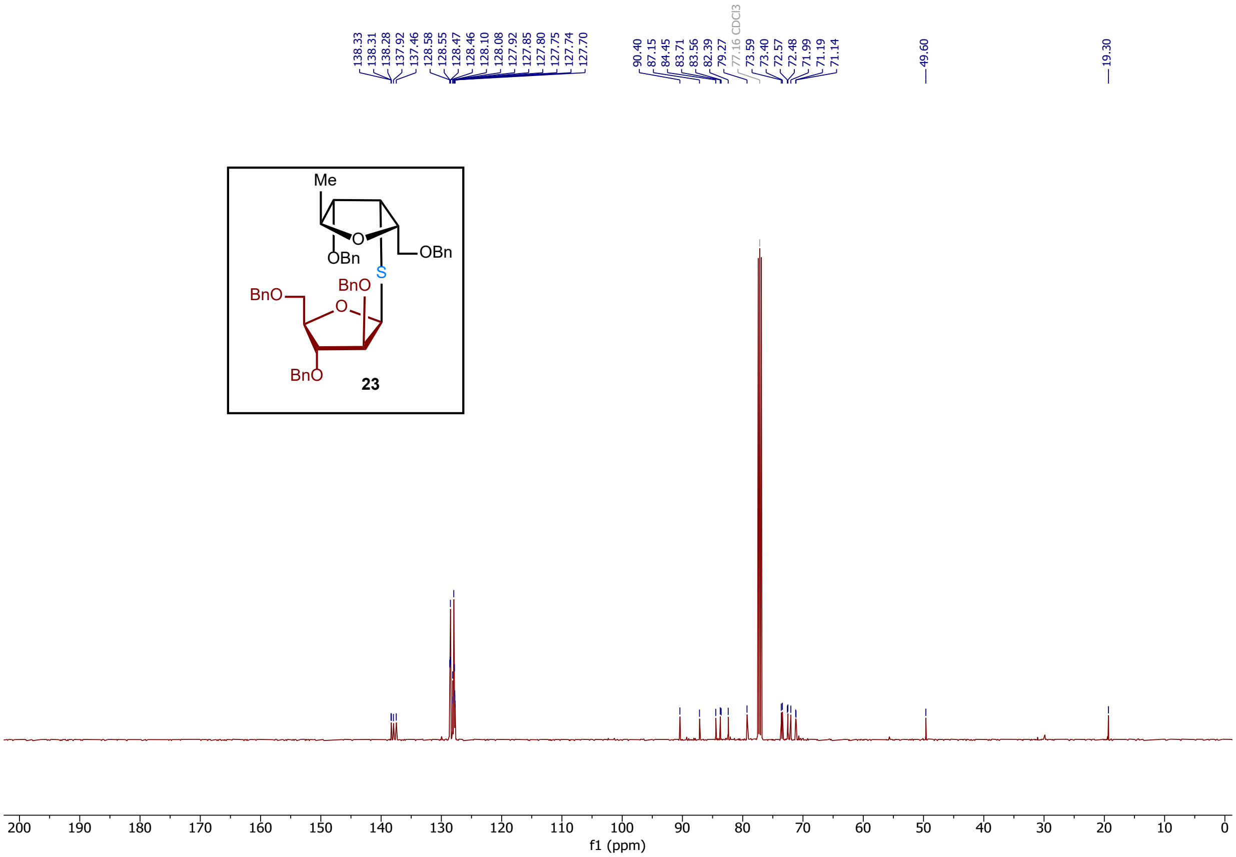
138.33
138.31
138.28
137.92
137.46
128.58
128.55
128.47
128.46
128.10
128.08
127.92
127.85
127.80
127.75
127.74
127.70
90.40
87.15
84.45
83.71
83.56
82.39
79.27
77.16 CDCl3
73.59
73.40
72.57
72.48
71.99
71.19
71.14
49.60
19.30
Me
O
OBn
OBn
S
BnO
BnO
O
BnO
23
200 190 180 170 160 150 140 130 120 110 100 90 80 70 60 50 40 30 20 10 0
f1 (ppm)

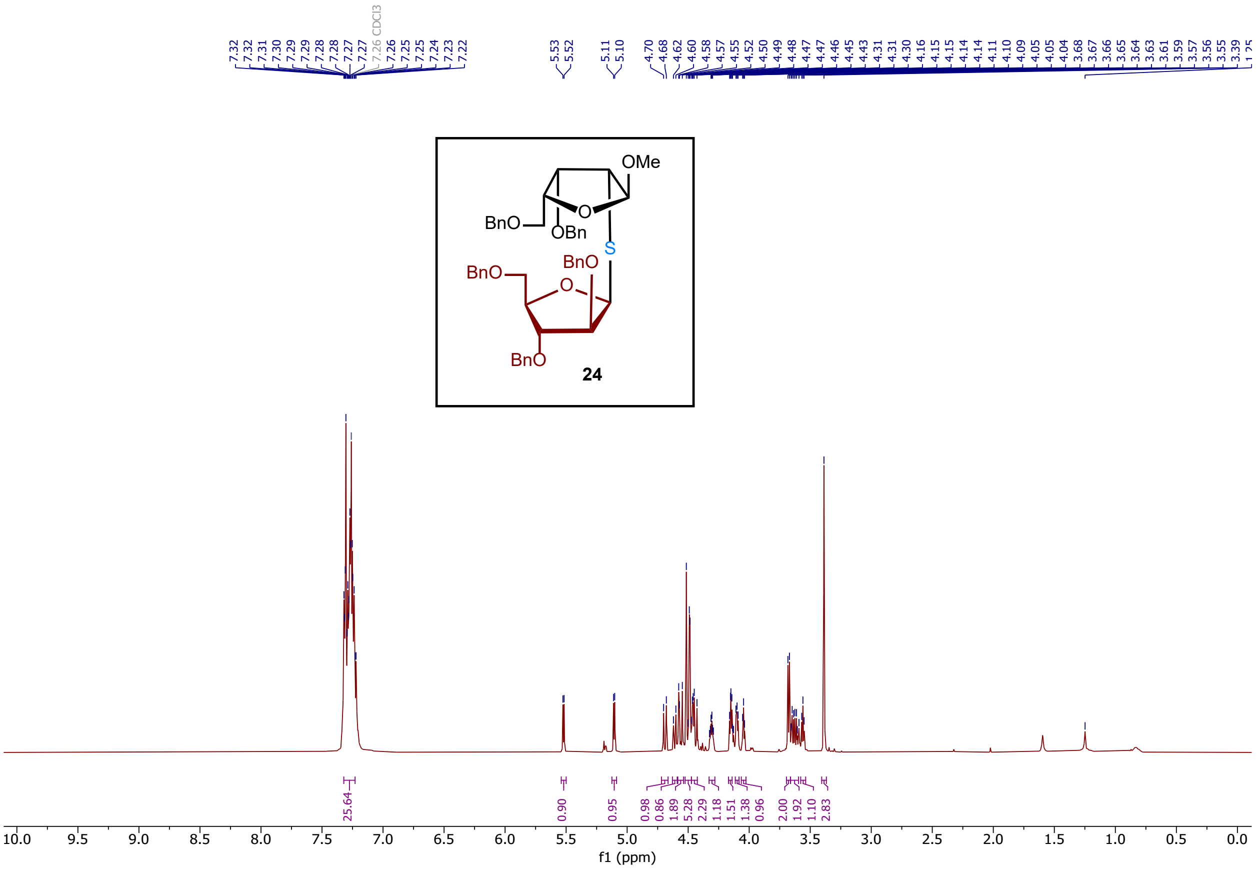
7.32 7.32 7.31 7.30 7.29 7.29 7.28 7.28 7.27 7.27 7.26 CDCl3 7.26 7.25 7.25 7.24 7.23 7.22
5.53 5.52
5.11 5.10
4.70 4.68 4.62 4.60 4.58 4.57 4.55 4.52 4.50 4.49 4.48 4.47 4.47 4.46 4.45 4.43 4.31 4.31 4.30 4.16 4.15 4.15 4.14 4.14 4.11 4.10 4.09 4.05 4.05 4.04 3.68 3.67 3.66 3.65 3.64 3.63 3.61 3.59 3.57 3.56 3.55 3.39
OMe
BnO
O
OBn
S
BnO
BnO
O
BnO
24
25.64
0.90
0.95
0.98 0.86 1.89 5.28 2.29 1.18 1.51 1.38 0.96
2.00 1.92 1.10 2.83
10.0 9.5 9.0 8.5 8.0 7.5 7.0 6.5 6.0 5.5 5.0 4.5 4.0 3.5 3.0 2.5 2.0 1.5 1.0 0.5 0.0
f1 (ppm)

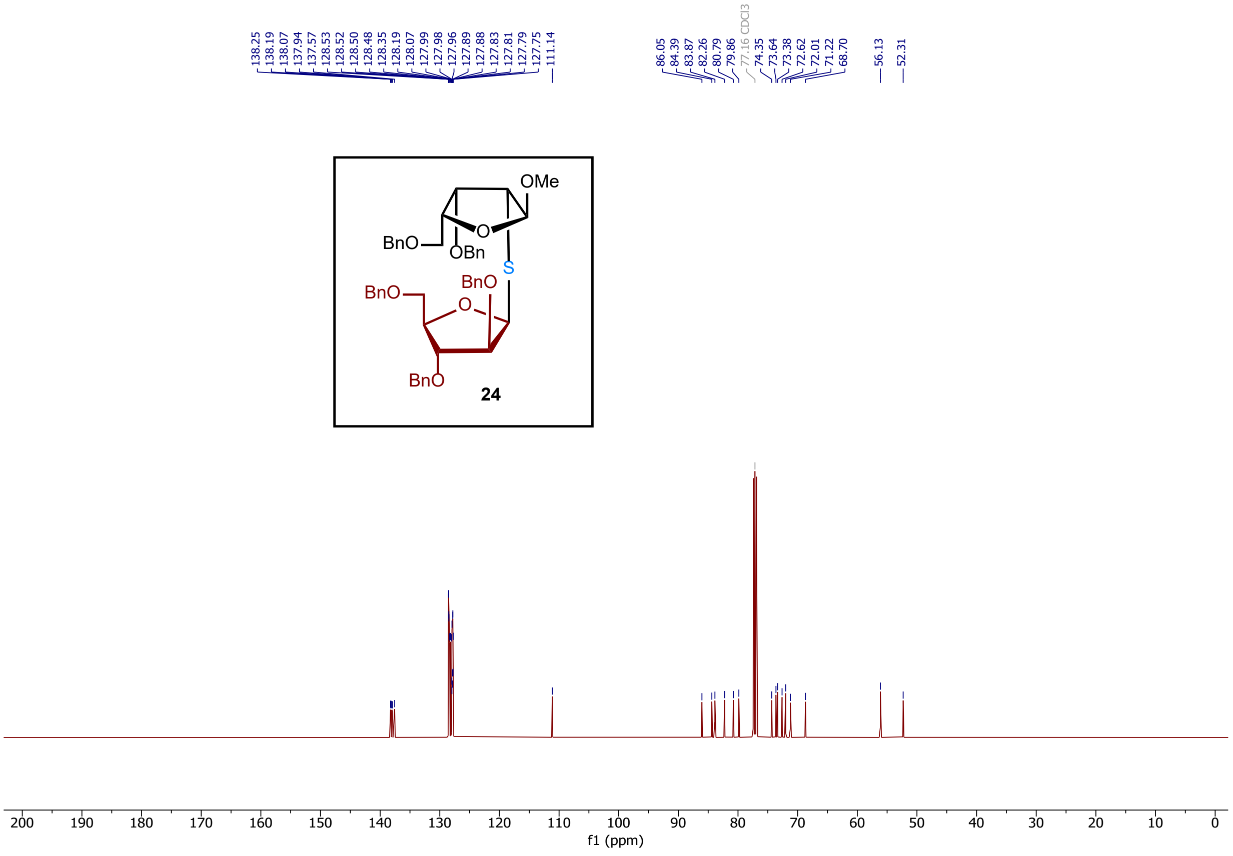
138.25 138.19 138.07 137.94 137.57 128.53 128.52 128.50 128.48 128.35 128.19 128.07 127.99 127.98 127.96 127.89 127.88 127.83 127.81 127.79 127.75
111.14
86.05 84.39 83.87 82.26 80.79 79.86 77.16 CDCl3 74.35 73.64 73.38 72.62 72.01 71.22 68.70
56.13
52.31
OMe
BnO
O
OBn
S
BnO
BnO
O
BnO
24
200 190 180 170 160 150 140 130 120 110 100 90 80 70 60 50 40 30 20 10 0
f1 (ppm)

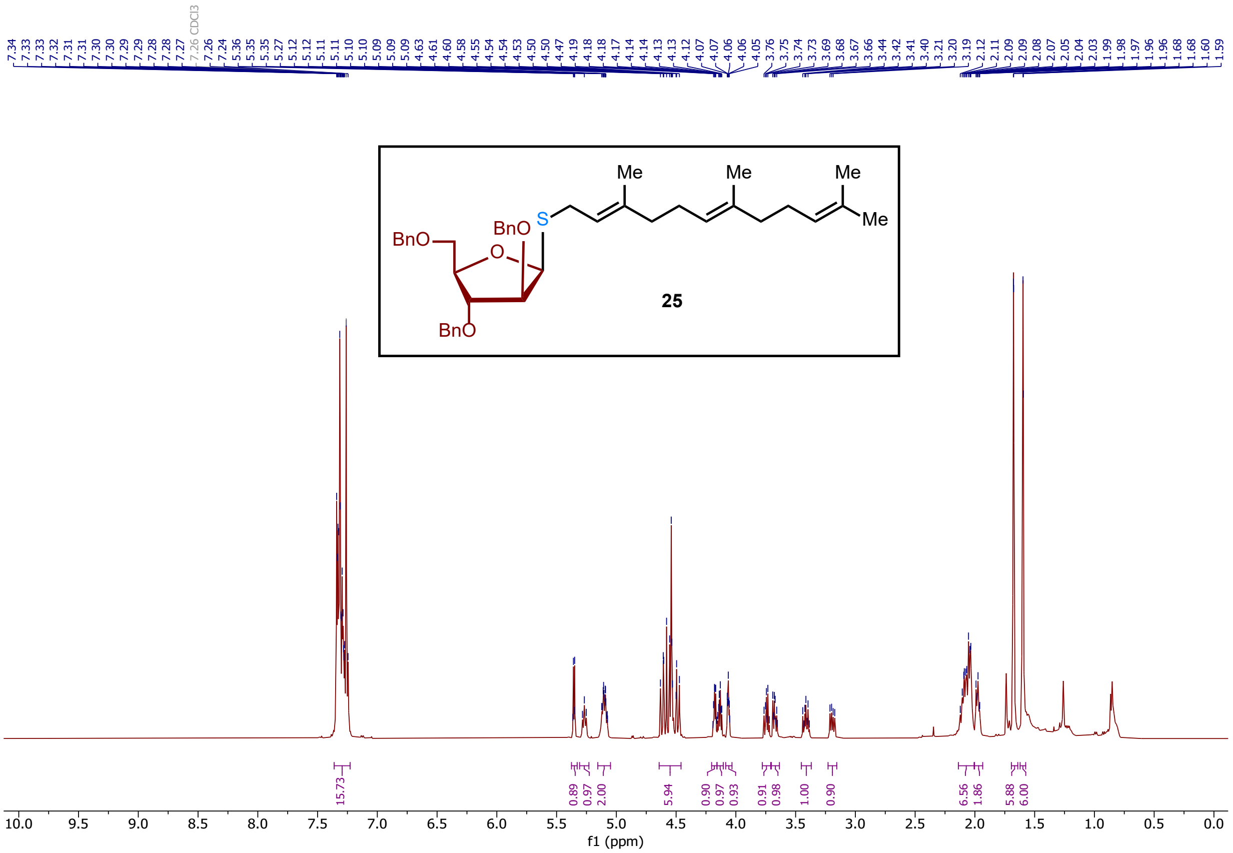
7.34 7.33 7.33 7.32 7.31 7.31 7.30 7.30 7.29 7.29 7.28 7.28 7.27 7.26 CDCl3 7.26 7.24 5.36 5.35 5.35 5.27 5.12 5.12 5.11 5.11 5.10 5.10 5.09 5.09 5.09 4.63 4.61 4.60 4.58 4.55 4.54 4.54 4.53 4.50 4.50 4.47 4.19 4.18 4.18 4.17 4.14 4.14 4.13 4.13 4.12 4.07 4.07 4.06 4.06 4.05 3.76 3.75 3.74 3.73 3.69 3.68 3.67 3.66 3.44 3.42 3.41 3.40 3.21 3.20 3.19 2.12 2.11 2.09 2.09 2.08 2.07 2.05 2.04 2.03 1.99 1.98 1.97 1.96 1.96 1.68 1.68 1.60 1.59
Me
Me
Me
Me
S
BnO
BnO
O
BnO
25
15.73
0.89
0.97
2.00
5.94
0.90
0.97
0.93
0.91
0.98
1.00
0.90
6.56
1.86
5.88
6.00
10.0 9.5 9.0 8.5 8.0 7.5 7.0 6.5 6.0 5.5 5.0 4.5 4.0 3.5 3.0 2.5 2.0 1.5 1.0 0.5 0.0
f1 (ppm)

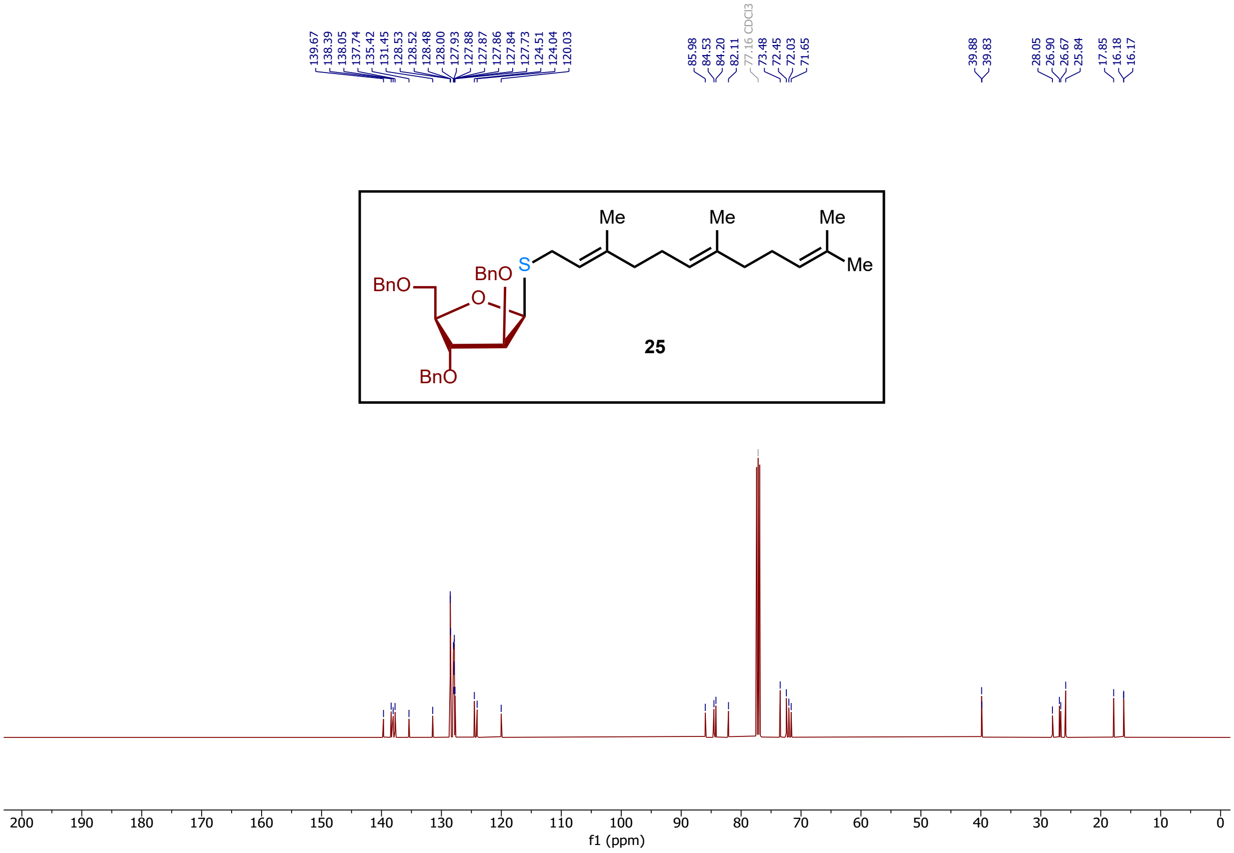
139.67 138.39 138.05 137.74 135.42 131.45 128.53 128.52 128.48 128.00 127.93 127.88 127.87 127.86 127.84 127.73 124.51 124.04 120.03
85.98 84.53 84.20 82.11 77.16 CDCl3 73.48 72.45 72.03 71.65
39.88 39.83
28.05 26.90 26.67 25.84 17.85 16.18 16.17
Me
Me
Me
Me
S
BnO
BnO
O
BnO
25
200 190 180 170 160 150 140 130 120 110 100 90 80 70 60 50 40 30 20 10 0
f1 (ppm)

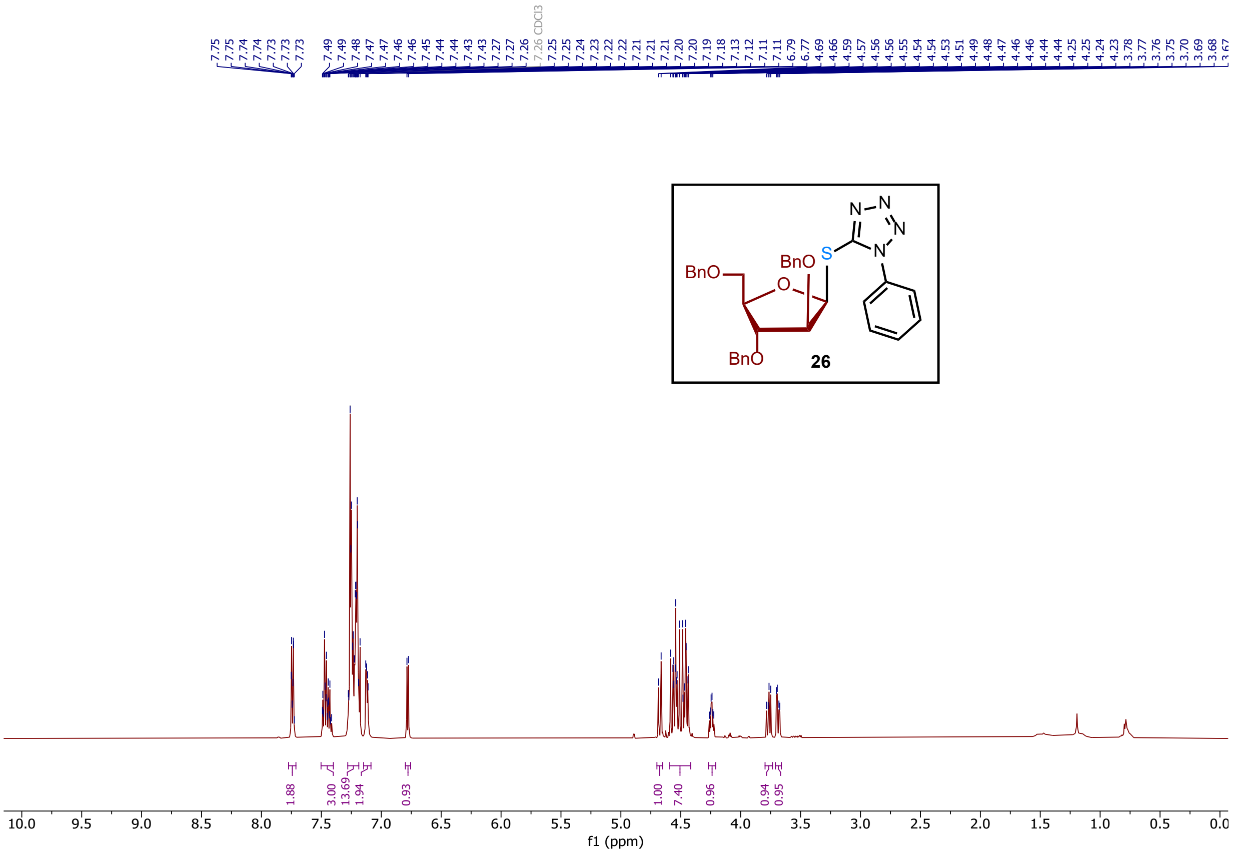

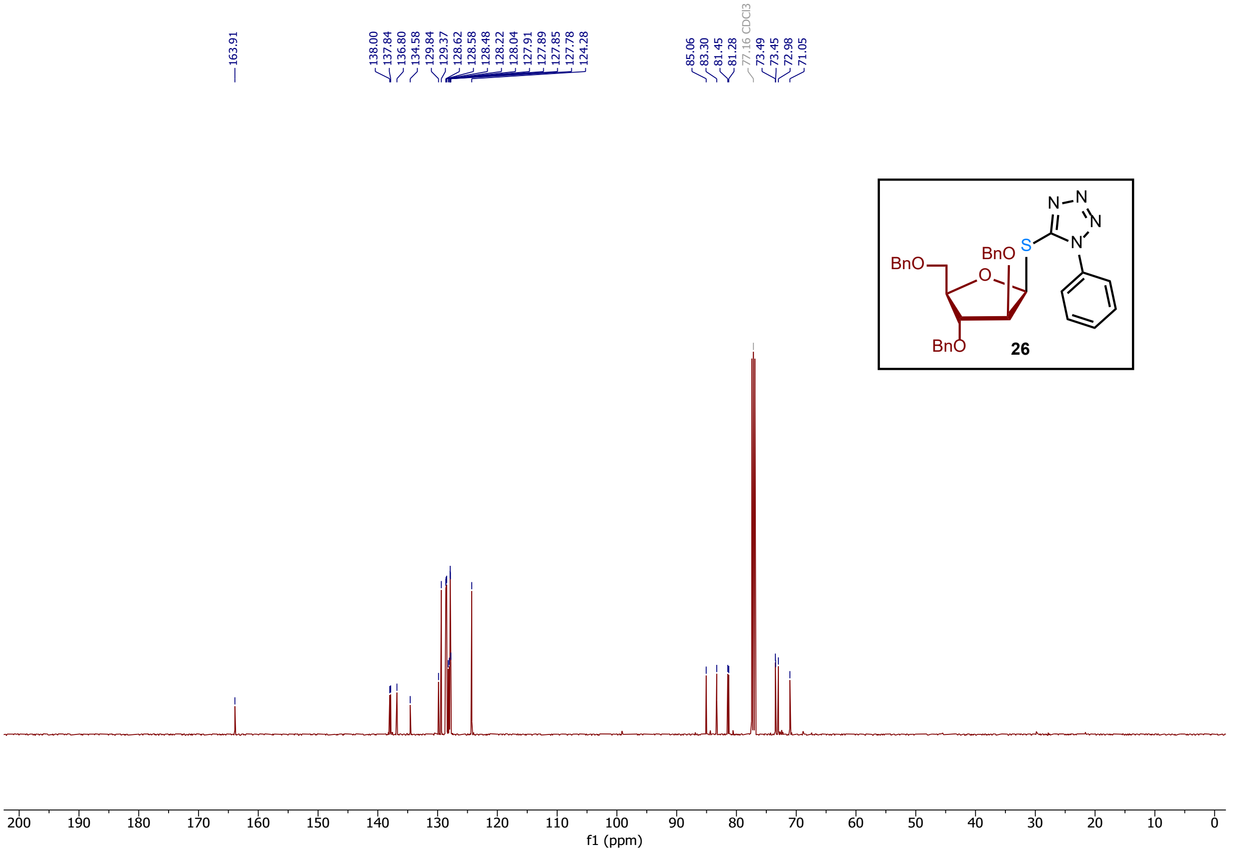

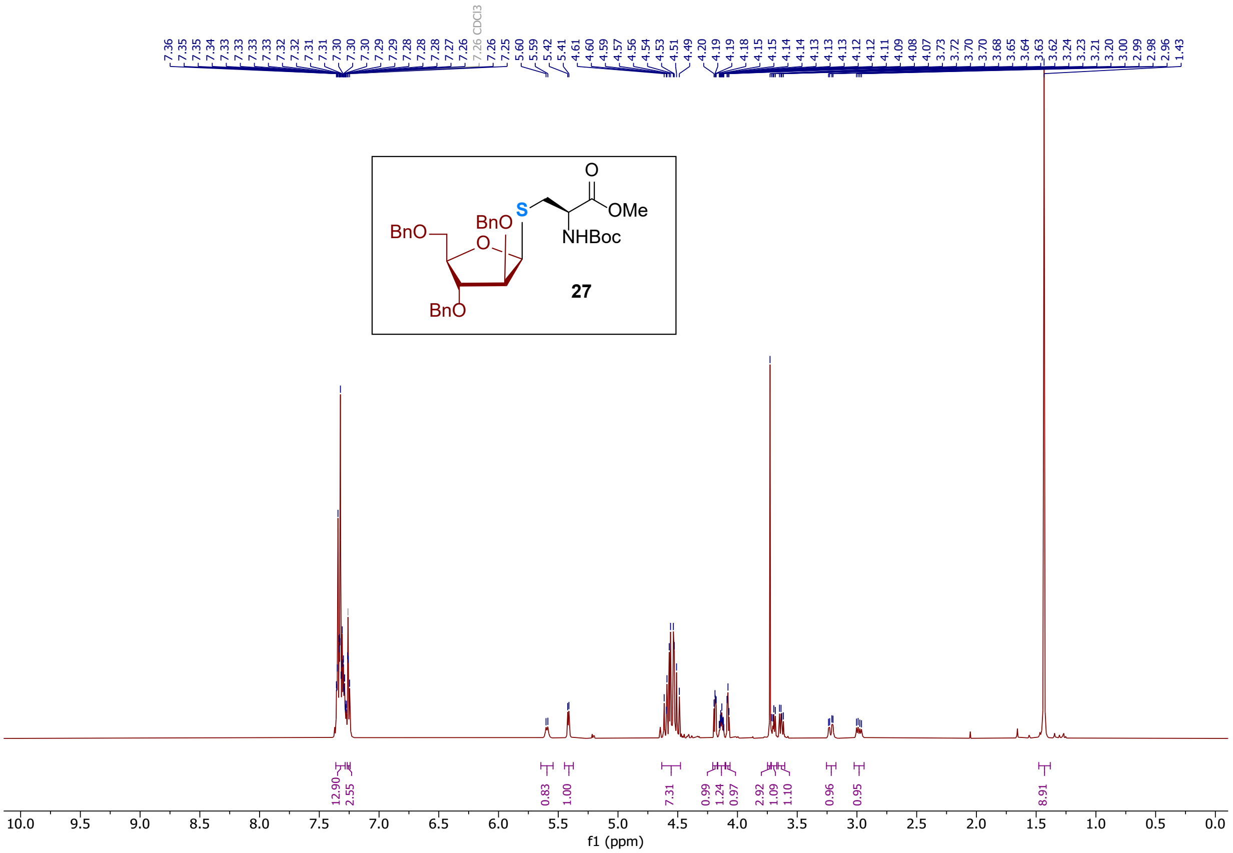

7.36 7.35 7.35 7.34 7.33 7.33 7.33 7.33 7.32 7.32 7.31 7.31 7.30 7.30 7.30 7.29 7.29 7.28 7.28 7.28 7.27 7.26 7.26 CDCl3 7.26 7.25 5.60 5.59 5.42 5.41 4.61 4.60 4.59 4.57 4.56 4.54 4.53 4.51 4.49 4.20 4.19 4.19 4.18 4.15 4.15 4.14 4.14 4.13 4.13 4.13 4.12 4.12 4.11 4.09 4.08 4.07 3.73 3.72 3.70 3.68 3.65 3.64 3.63 3.62 3.24 3.23 3.21 3.20 3.00 2.99 2.98 2.96 1.43
BnO
BnO
S
O
OMe
NHBoc
O
BnO
27
12.90 2.55 0.83 1.00 7.31 0.99 1.24 0.97 2.92 1.09 1.10 0.96 0.95 8.91
10.0 9.5 9.0 8.5 8.0 7.5 7.0 6.5 6.0 5.5 5.0 4.5 4.0 3.5 3.0 2.5 2.0 1.5 1.0 0.5 0.0
f1 (ppm)

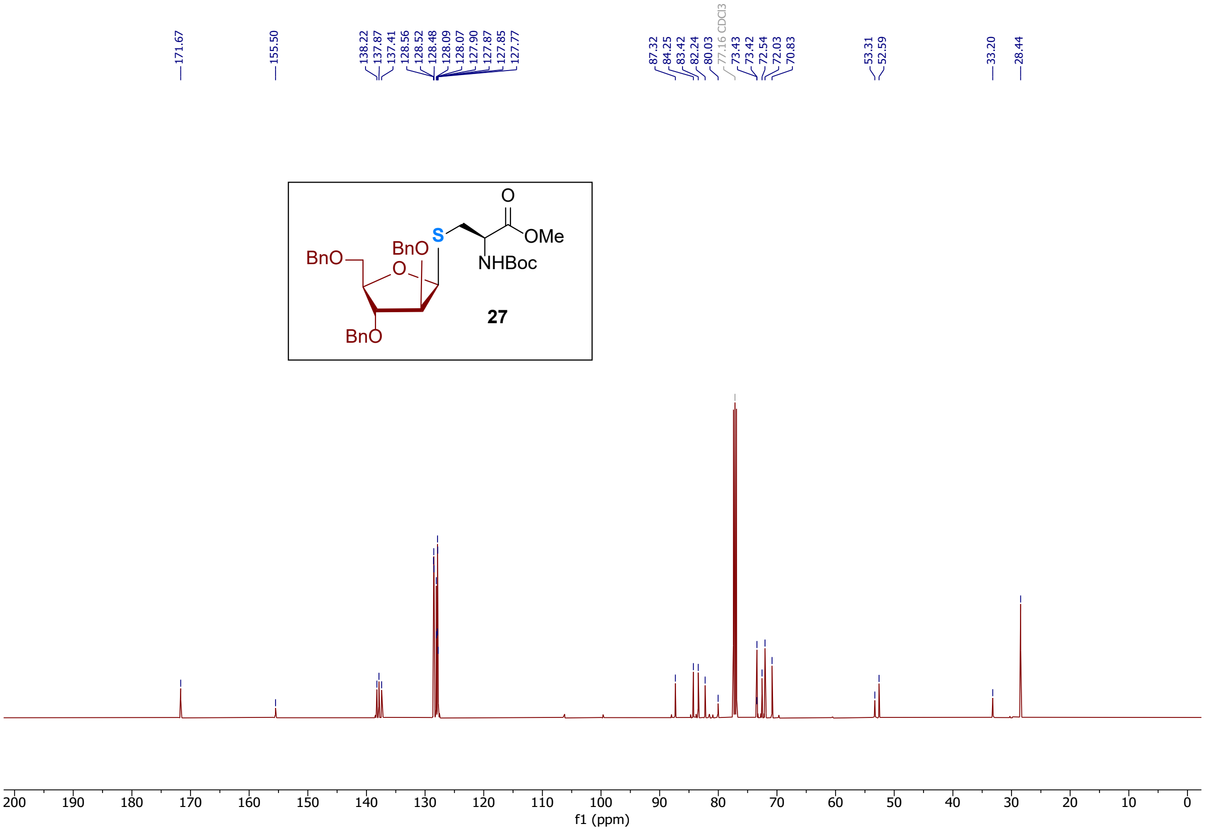

171.67 155.50 138.22 137.87 137.41 128.56 128.52 128.48 128.09 128.07 127.90 127.87 127.85 127.77 87.32 84.25 83.42 82.24 80.03 77.16 CDCl3 73.43 73.42 72.54 72.03 70.83 53.31 52.59 33.20 28.44
BnO
BnO
S
O
OMe
NHBoc
O
BnO
27
200 190 180 170 160 150 140 130 120 110 100 90 80 70 60 50 40 30 20 10 0
f1 (ppm)

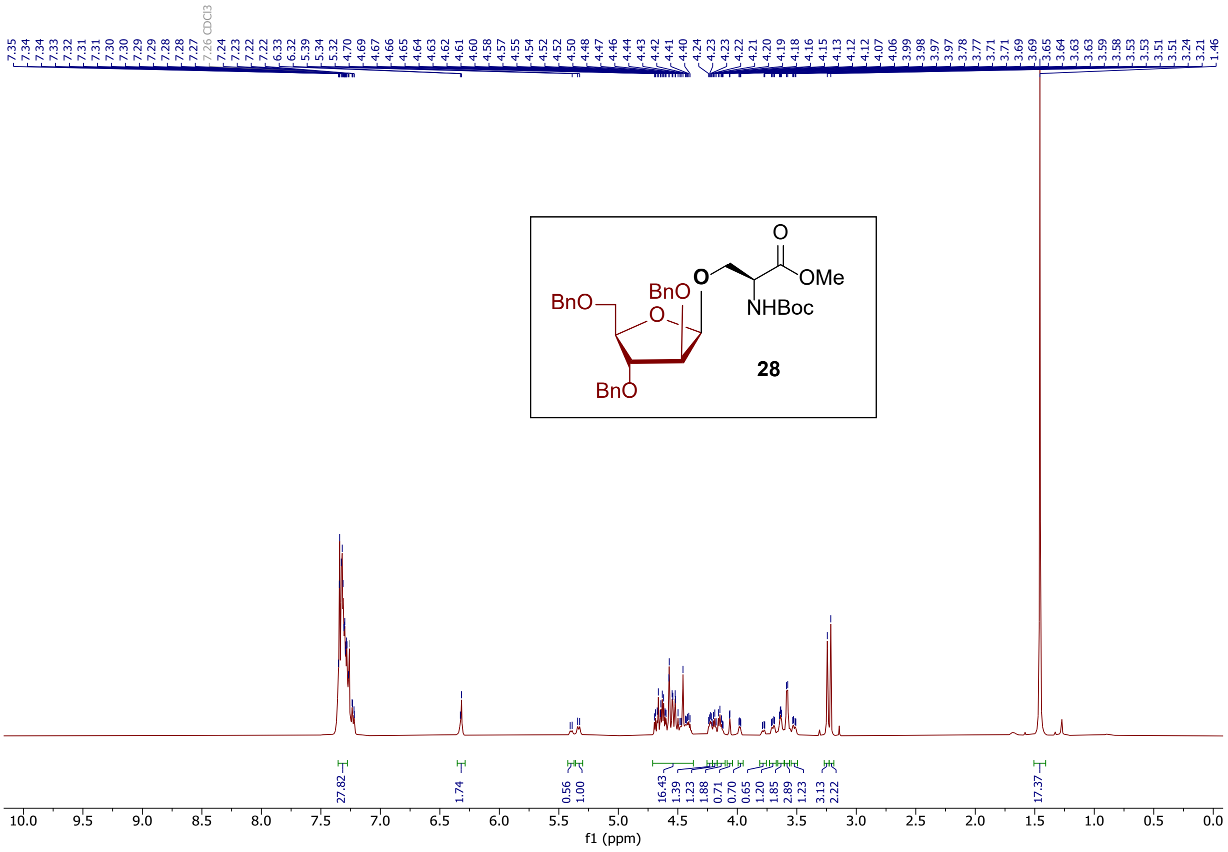

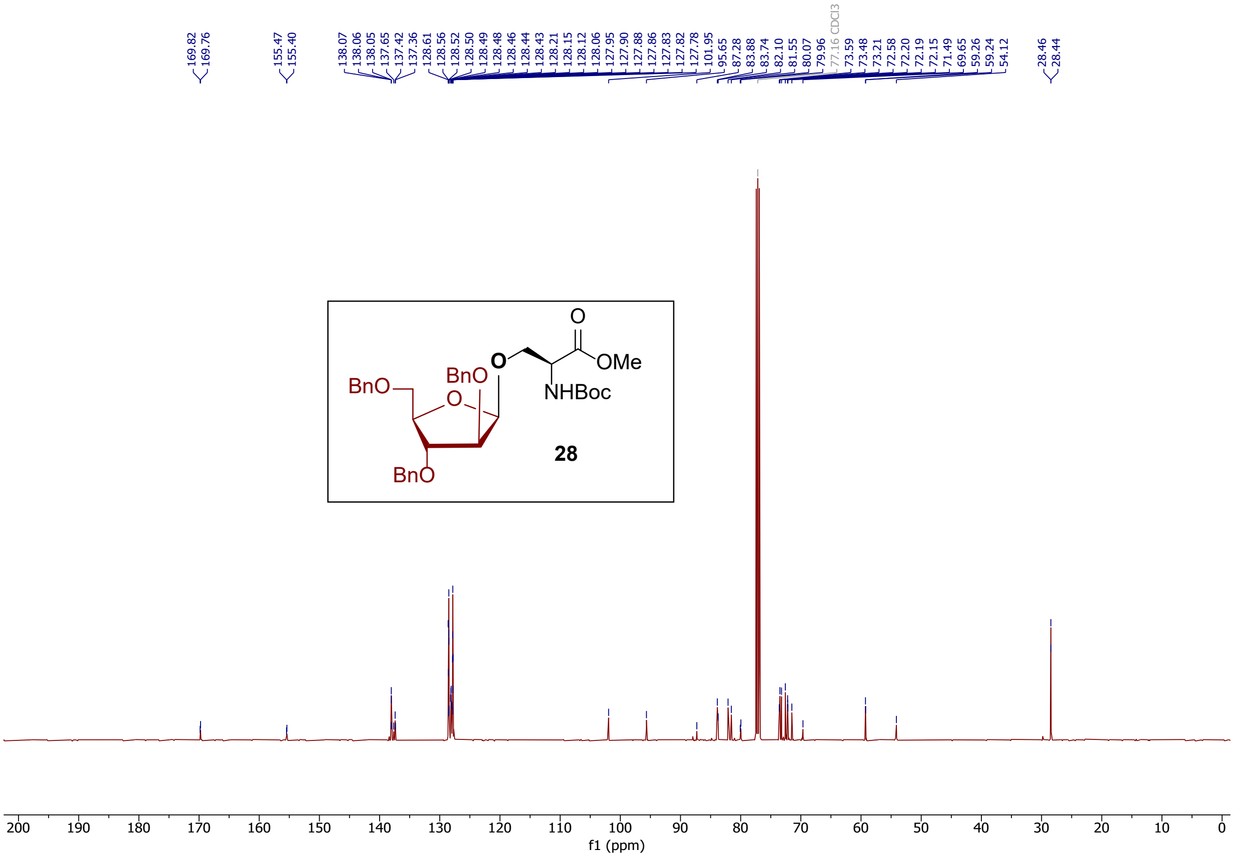

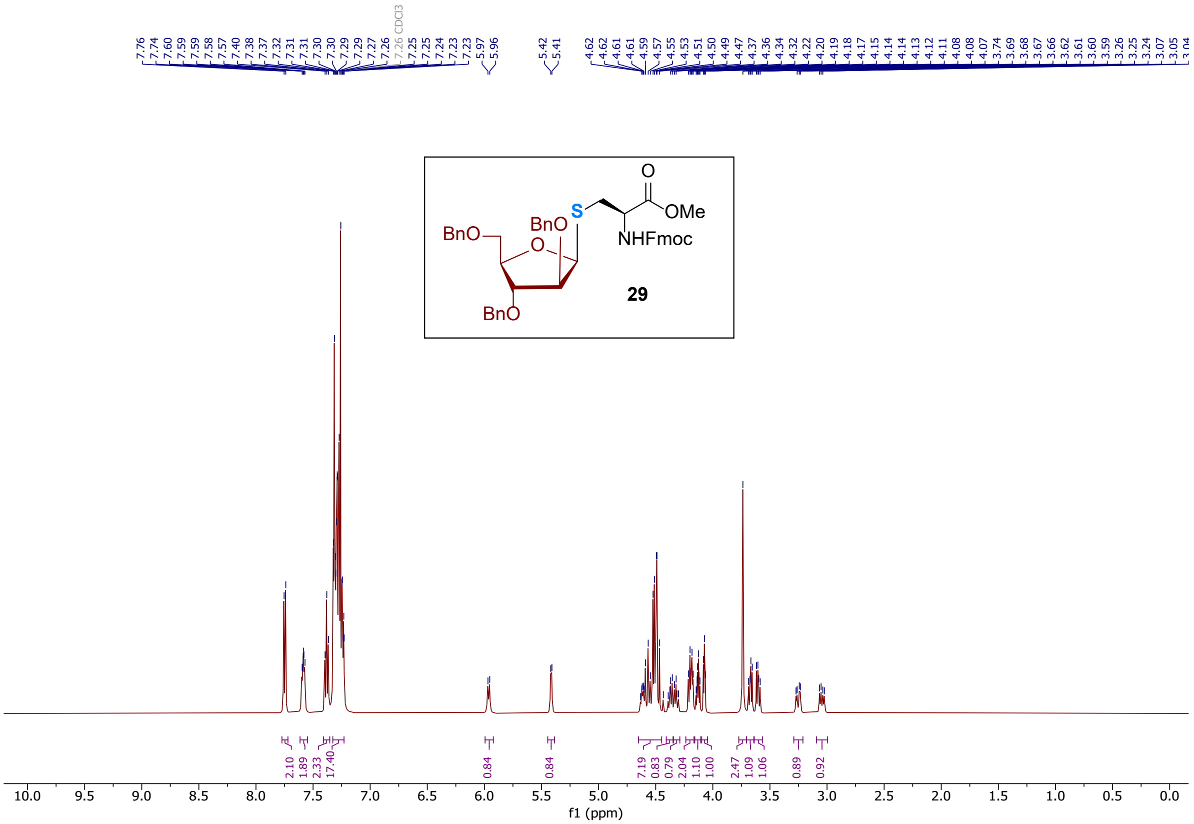
BnO
BnO
S
O
OMe
NHFmoc
BnO
29
f1 (ppm)

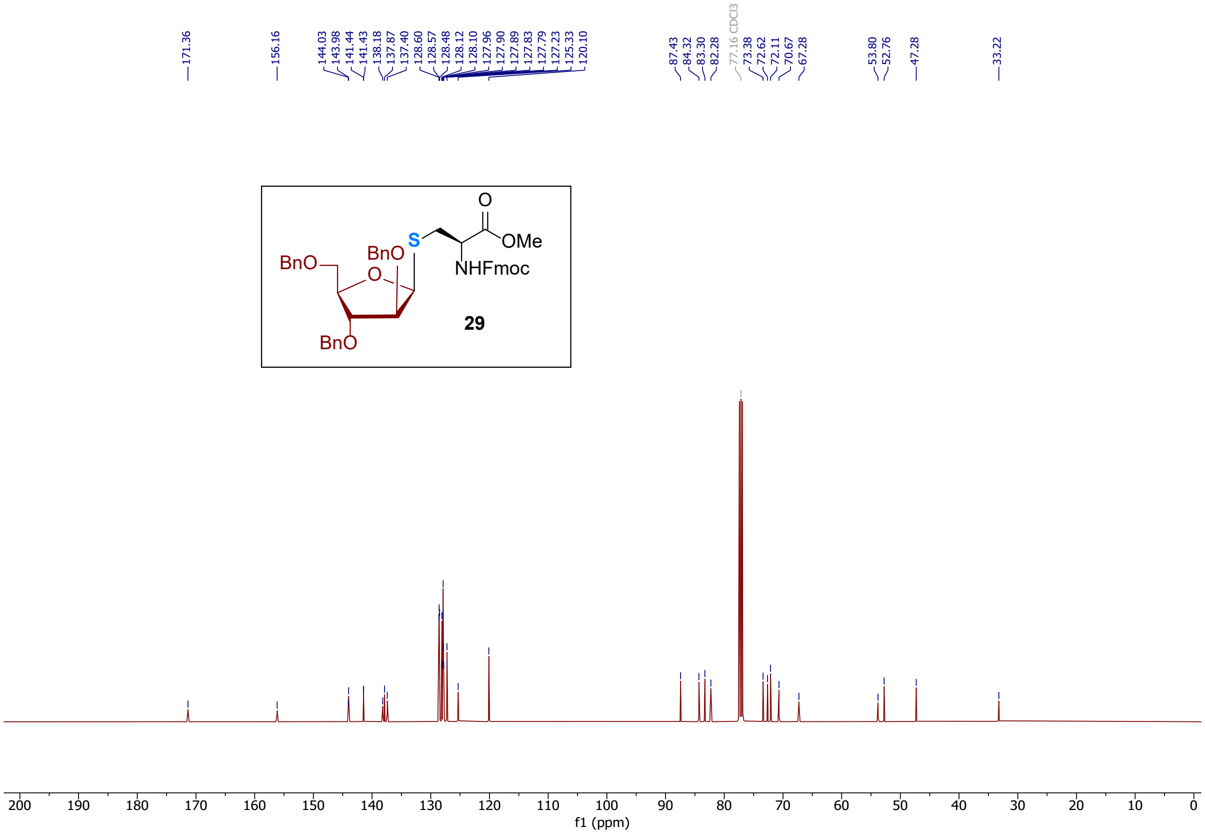
BnO
BnO
S
O
OMe
NHFmoc
BnO
29
f1 (ppm)

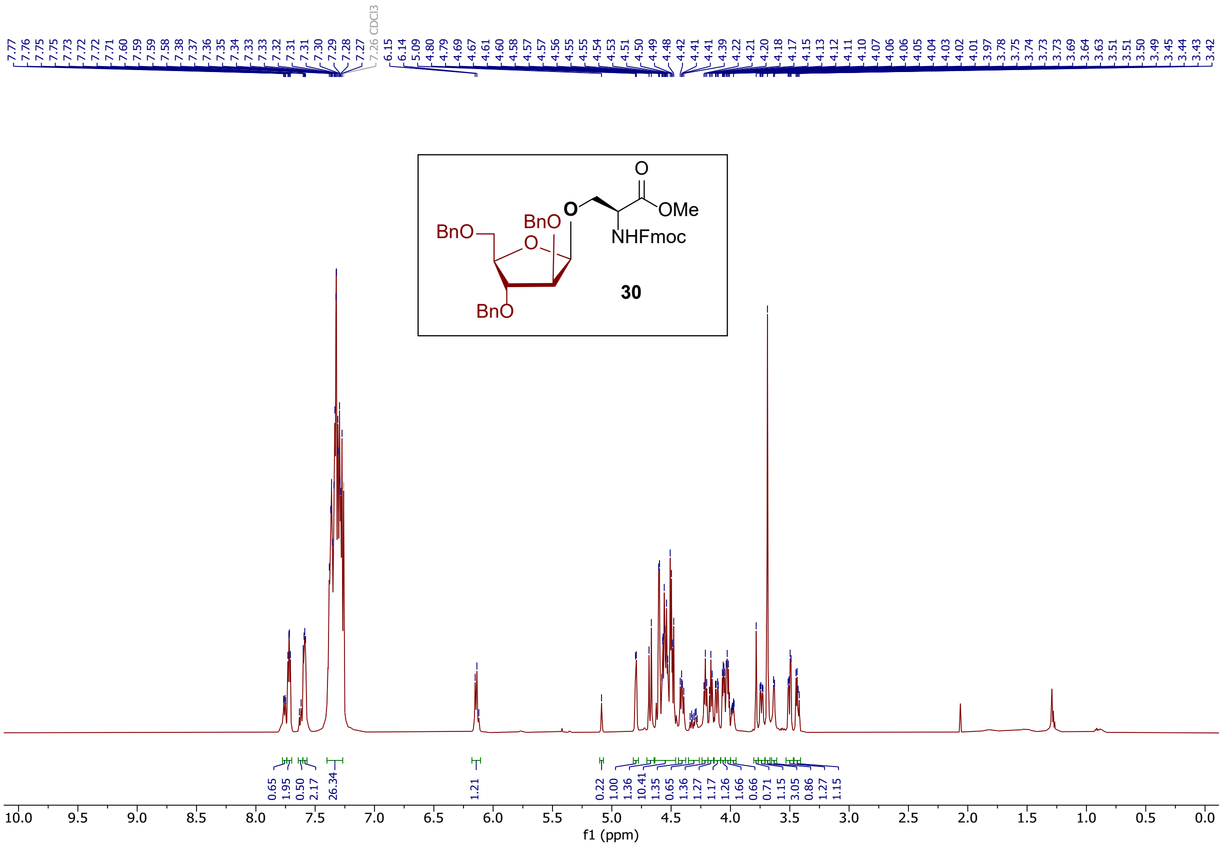

7.77 7.76 7.75 7.75 7.73 7.72 7.72 7.71 7.60 7.59 7.59 7.58 7.38 7.37 7.36 7.35 7.34 7.33 7.33 7.32 7.31 7.31 7.30 7.29 7.28 7.27
7.26 CDCl3
6.15 6.14 5.09 4.80 4.79 4.69 4.67 4.61 4.60 4.58 4.57 4.57 4.56 4.55 4.55 4.54 4.53 4.51 4.50 4.49 4.48 4.42 4.41 4.39 4.22 4.21 4.20 4.18 4.17 4.15 4.13 4.12 4.11 4.10 4.07 4.06 4.06 4.05 4.04 4.03 4.02 4.01 3.97 3.78 3.75 3.74 3.73 3.73 3.69 3.64 3.63 3.51 3.51 3.50 3.49 3.45 3.44 3.43 3.42
O
OMe
BnO
BnO
O
O
NHFmoc
BnO
30
0.65 1.95 0.50 2.17 26.34 1.21 0.22 1.00 1.36 10.41 1.35 0.65 1.36 1.27 1.17 1.26 1.66 0.66 0.71 1.15 3.05 0.86 1.27 1.15
10.0 9.5 9.0 8.5 8.0 7.5 7.0 6.5 6.0 5.5 5.0 4.5 4.0 3.5 3.0 2.5 2.0 1.5 1.0 0.5 0.0
f1 (ppm)

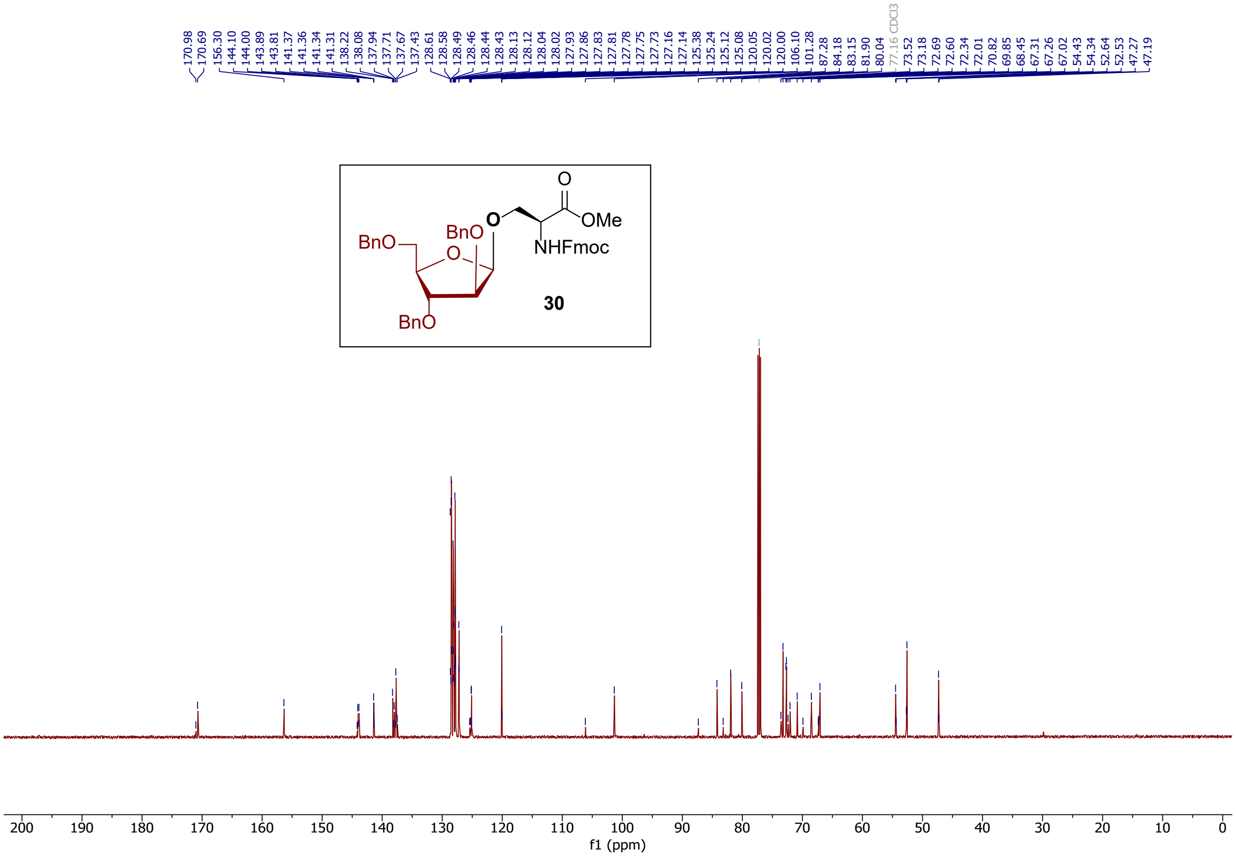

170.98 170.69 156.30 144.10 144.00 143.89 143.81 141.37 141.36 141.34 141.31 138.22 138.08 137.94 137.71 137.67 137.43 128.61 128.58 128.49 128.46 128.44 128.43 128.13 128.12 128.04 128.02 127.93 127.86 127.83 127.81 127.78 127.75 127.73 127.16 127.14 125.38 125.24 125.12 125.08 120.05 120.02 120.00 106.10 101.28 87.28 84.18 83.15 81.90 80.04
77.16 CDCl3
73.52 73.18 72.69 72.60 72.34 72.01 70.82 69.85 68.45 67.31 67.26 67.02 54.43 54.34 52.64 52.53 47.27 47.19
O
OMe
BnO
BnO
O
O
NHFmoc
BnO
30
200 190 180 170 160 150 140 130 120 110 100 90 80 70 60 50 40 30 20 10 0
f1 (ppm)

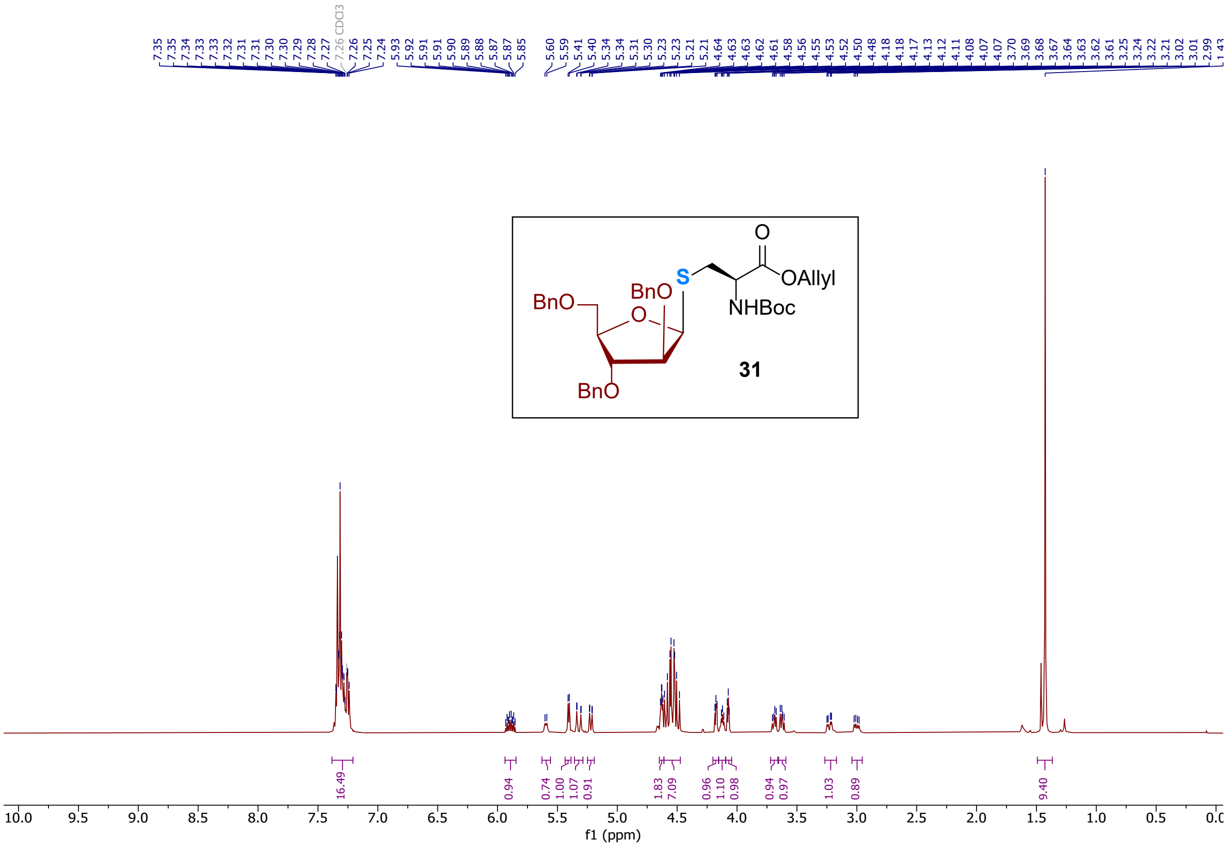
7.35 7.35 7.34 7.33 7.33 7.32 7.31 7.31 7.30 7.30 7.29 7.28 7.27 7.26 CDCl3 7.26 7.25 7.24 7.24 5.93 5.92 5.91 5.91 5.90 5.89 5.88 5.87 5.87 5.85 5.60 5.59 5.41 5.40 5.34 5.34 5.31 5.30 5.23 5.23 5.21 5.21 4.64 4.63 4.63 4.62 4.61 4.58 4.56 4.55 4.53 4.52 4.50 4.48 4.18 4.18 4.17 4.13 4.12 4.11 4.08 4.07 4.07 3.70 3.69 3.68 3.67 3.64 3.63 3.62 3.61 3.25 3.24 3.22 3.21 3.02 3.01 2.99
O
BnO
S
OAllyl
NHBoc
BnO
O
BnO
31
16.49
0.94
0.74
1.00
1.07
0.91
1.83
7.09
0.96
1.10
0.98
0.94
0.97
1.03
0.89
9.40
f1 (ppm)

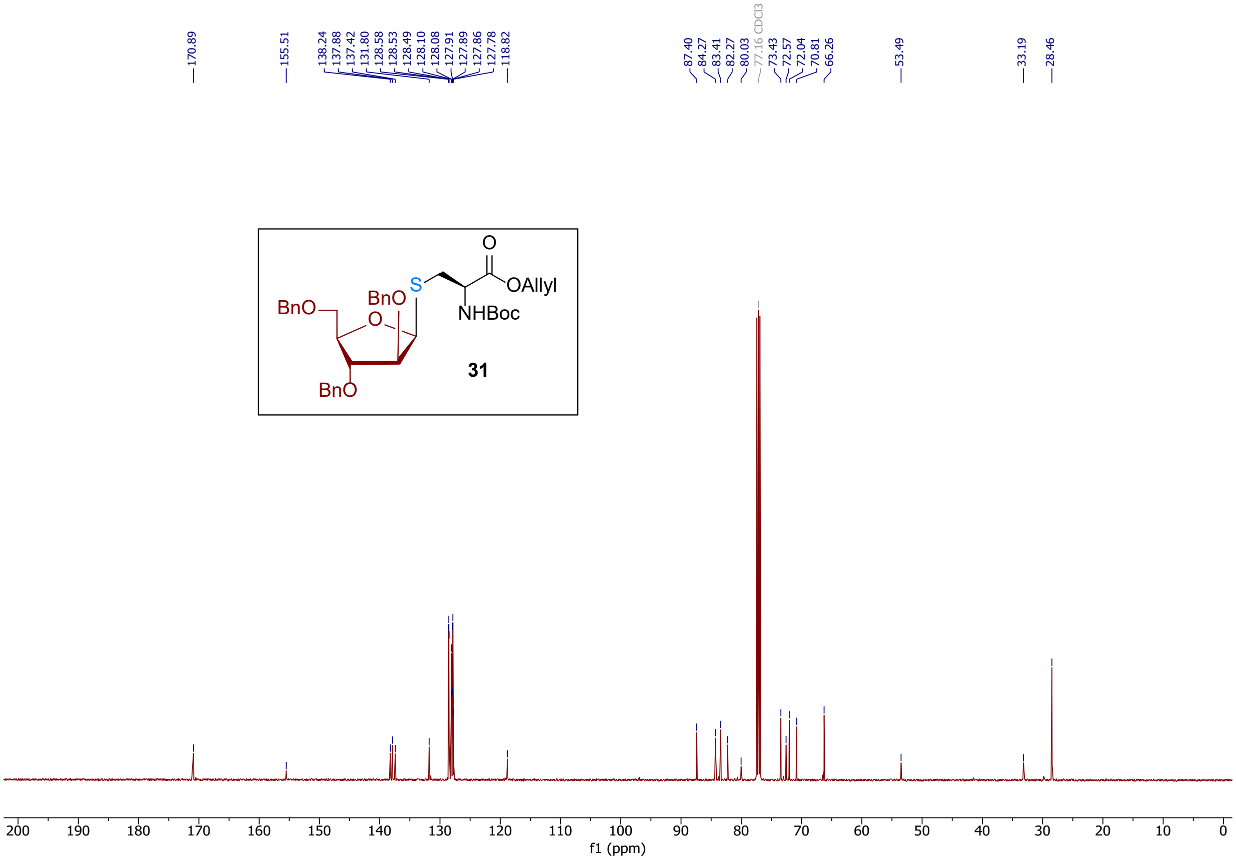
170.89
155.51
138.24 137.88 137.42 131.80 128.58 128.53 128.49 128.10 128.08 127.91 127.89 127.86 127.78 118.82
87.40 84.27 83.41 82.27 80.03 77.16 CDCl3 73.43 72.57 72.04 70.81 66.26
53.49
33.19
28.46
O
BnO
BnO
S
OAllyl
NHBoc
BnO
31
f1 (ppm)

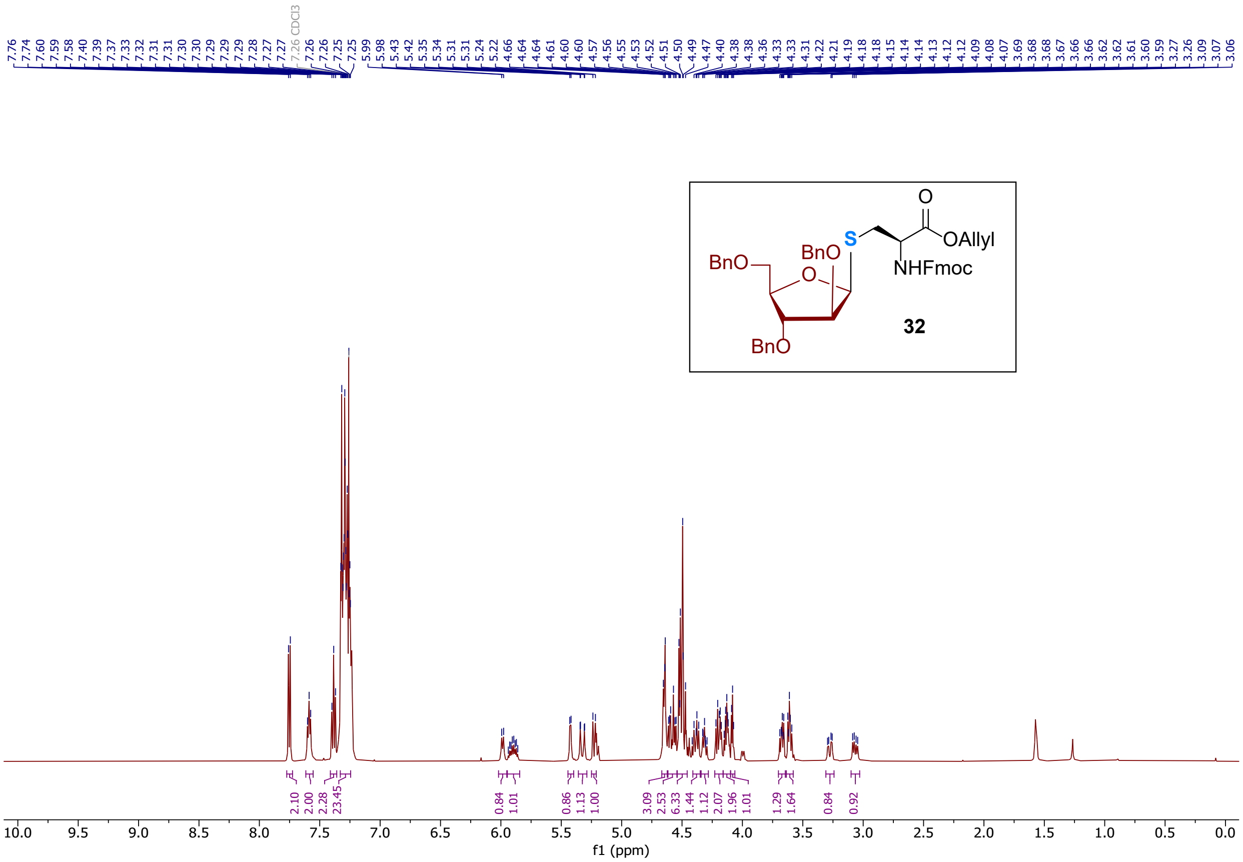

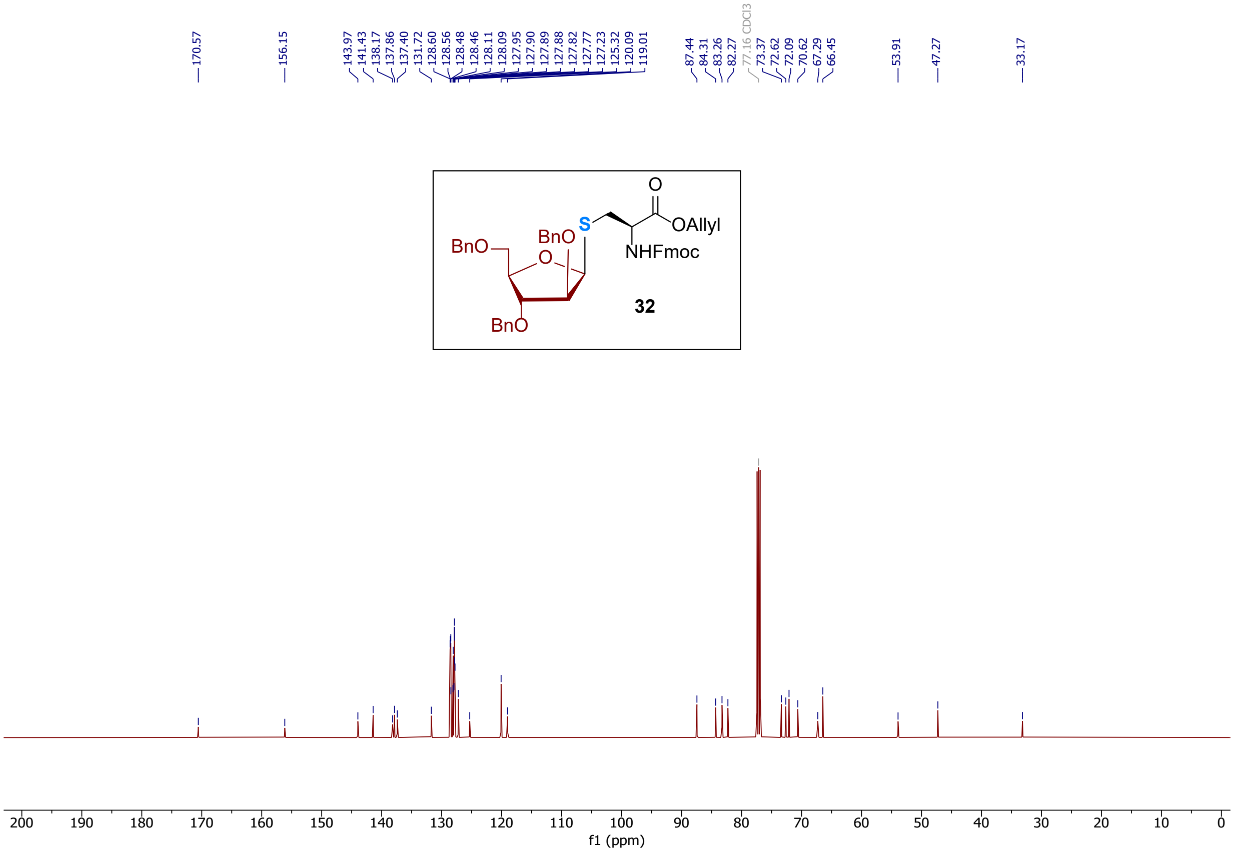

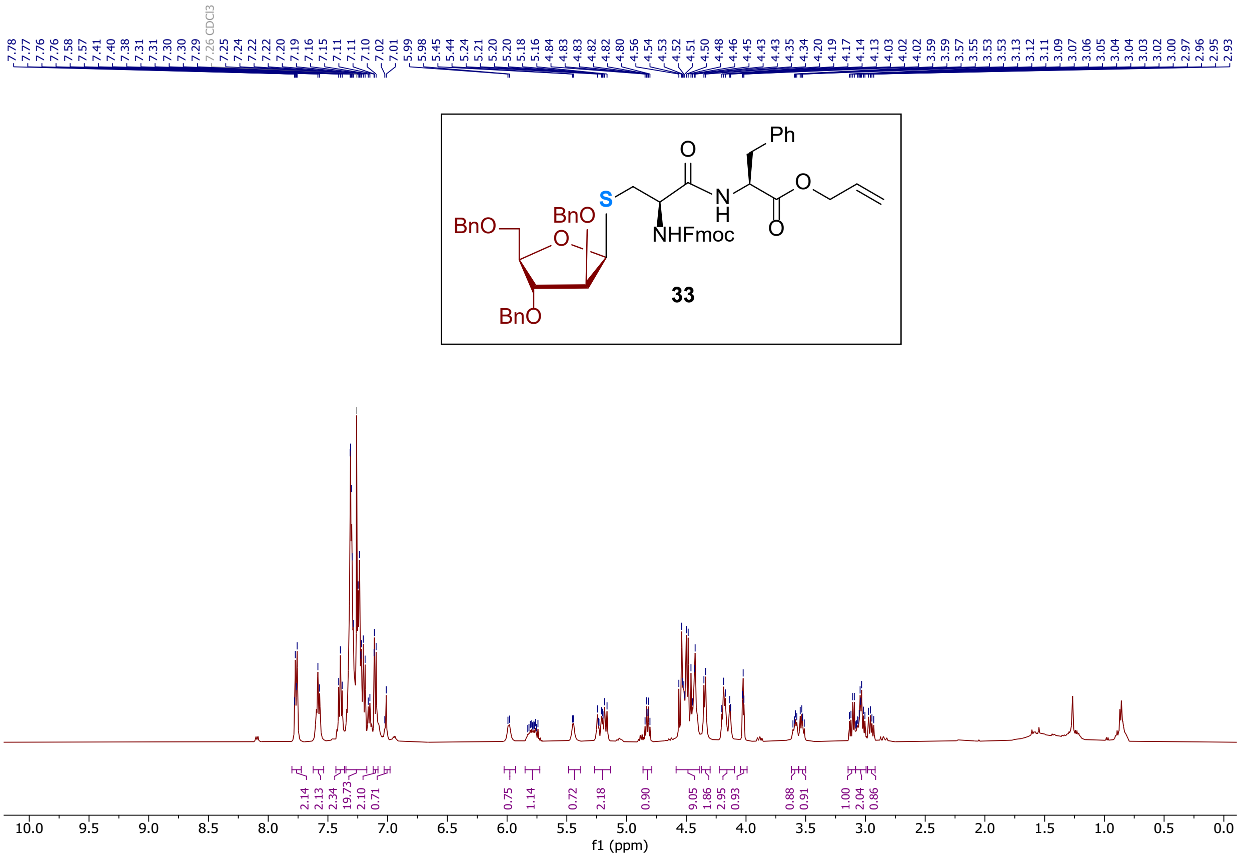
7.78 7.77 7.76 7.76 7.58 7.57 7.41 7.40 7.38 7.31 7.31 7.30 7.30 7.29 7.26 CDCl3 7.25 7.24 7.22 7.20 7.19 7.16 7.15 7.11 7.11 7.10 7.02 7.01 5.99 5.98 5.45 5.44 5.24 5.21 5.20 5.20 5.18 5.16 4.84 4.83 4.83 4.82 4.80 4.56 4.54 4.53 4.52 4.51 4.50 4.48 4.46 4.45 4.43 4.43 4.35 4.34 4.20 4.19 4.17 4.14 4.13 4.03 4.02 4.02 3.59 3.59 3.57 3.55 3.53 3.53 3.13 3.12 3.11 3.09 3.07 3.06 3.05 3.04 3.04 3.03 3.02 3.00 2.97 2.96 2.95 2.93
Ph
O
S
N
H
O
BnO
BnO
O
NHFmoc
O
BnO
33
2.14 2.13 2.34 19.73 2.10 0.71 0.75 1.14 0.72 2.18 0.90 9.05 1.86 2.95 0.93 0.88 0.91 1.00 2.04 0.86
10.0 9.5 9.0 8.5 8.0 7.5 7.0 6.5 6.0 5.5 5.0 4.5 4.0 3.5 3.0 2.5 2.0 1.5 1.0 0.5 0.0
f1 (ppm)

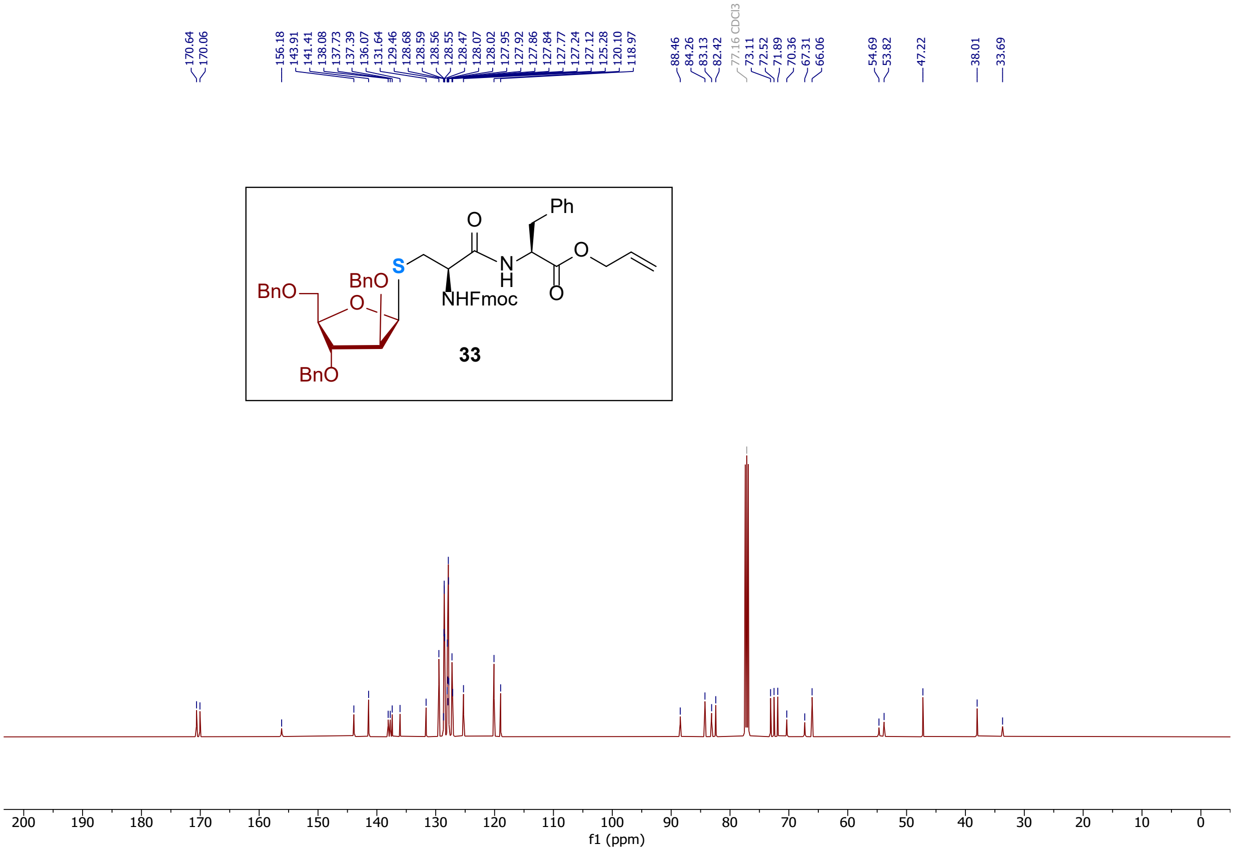
170.64 170.06 156.18 143.91 141.41 138.08 137.73 137.39 136.07 131.64 129.46 128.68 128.59 128.56 128.55 128.47 128.07 128.02 127.95 127.92 127.86 127.84 127.77 127.24 127.12 125.28 120.10 118.97 88.46 84.26 83.13 82.42 77.16 CDCl3 73.11 72.52 71.89 70.36 67.31 66.06 54.69 53.82 47.22 38.01 33.69
Ph
O
S
N
H
O
BnO
BnO
O
NHFmoc
O
BnO
33
200 190 180 170 160 150 140 130 120 110 100 90 80 70 60 50 40 30 20 10 0
f1 (ppm)

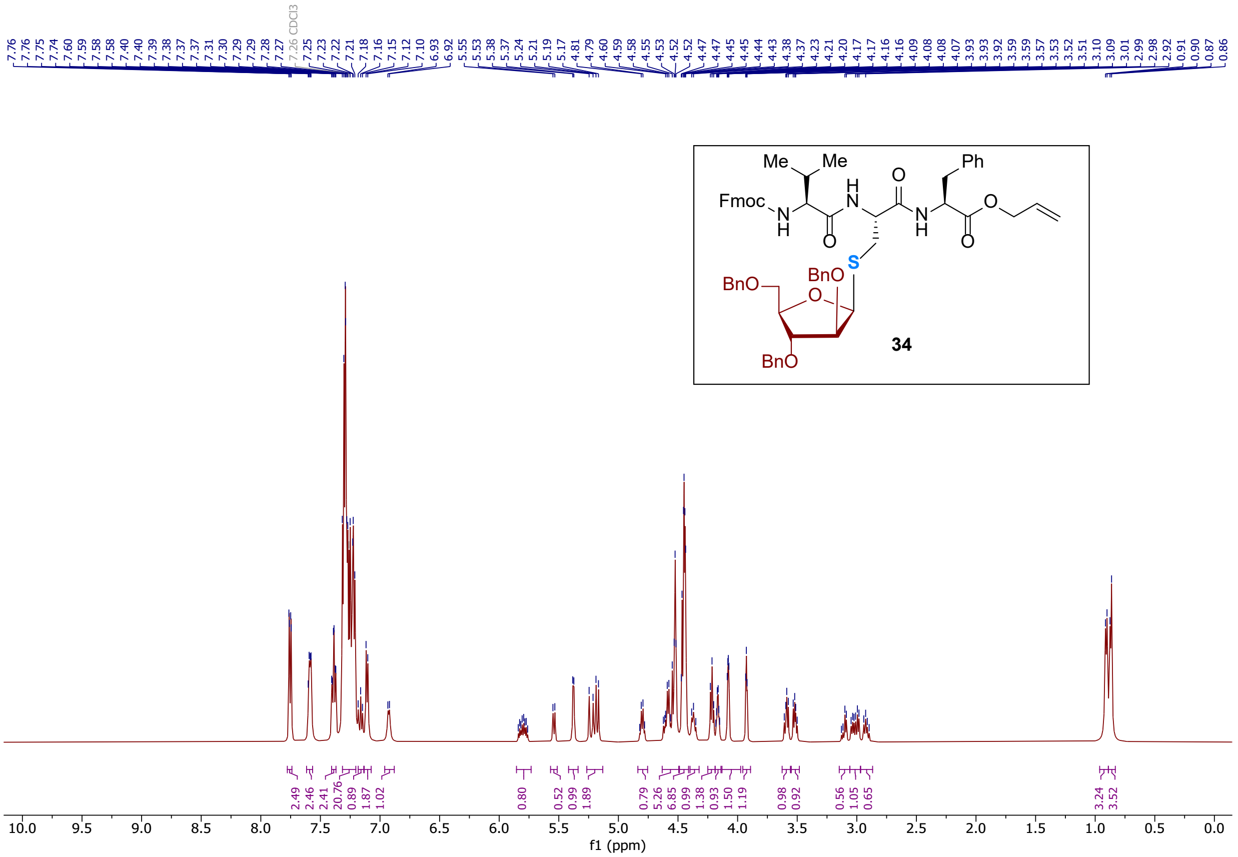

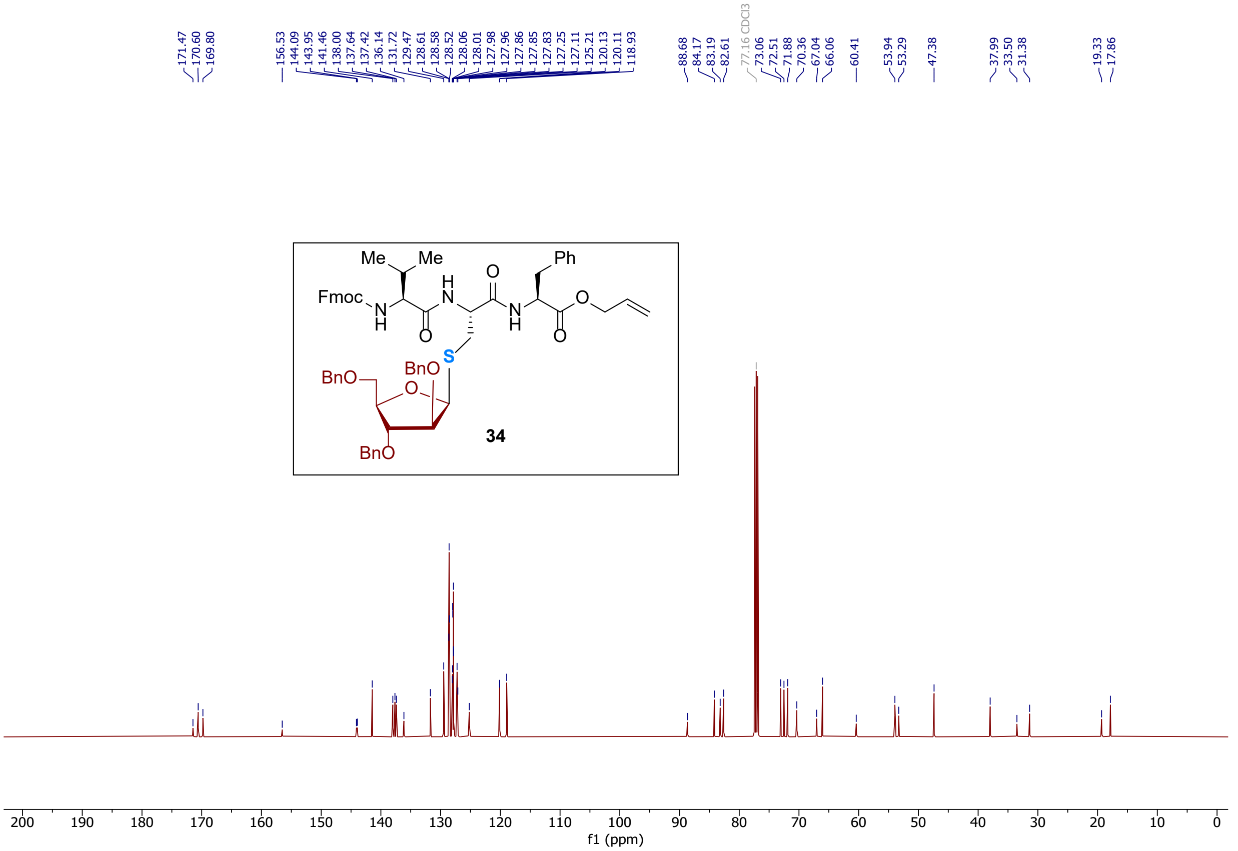

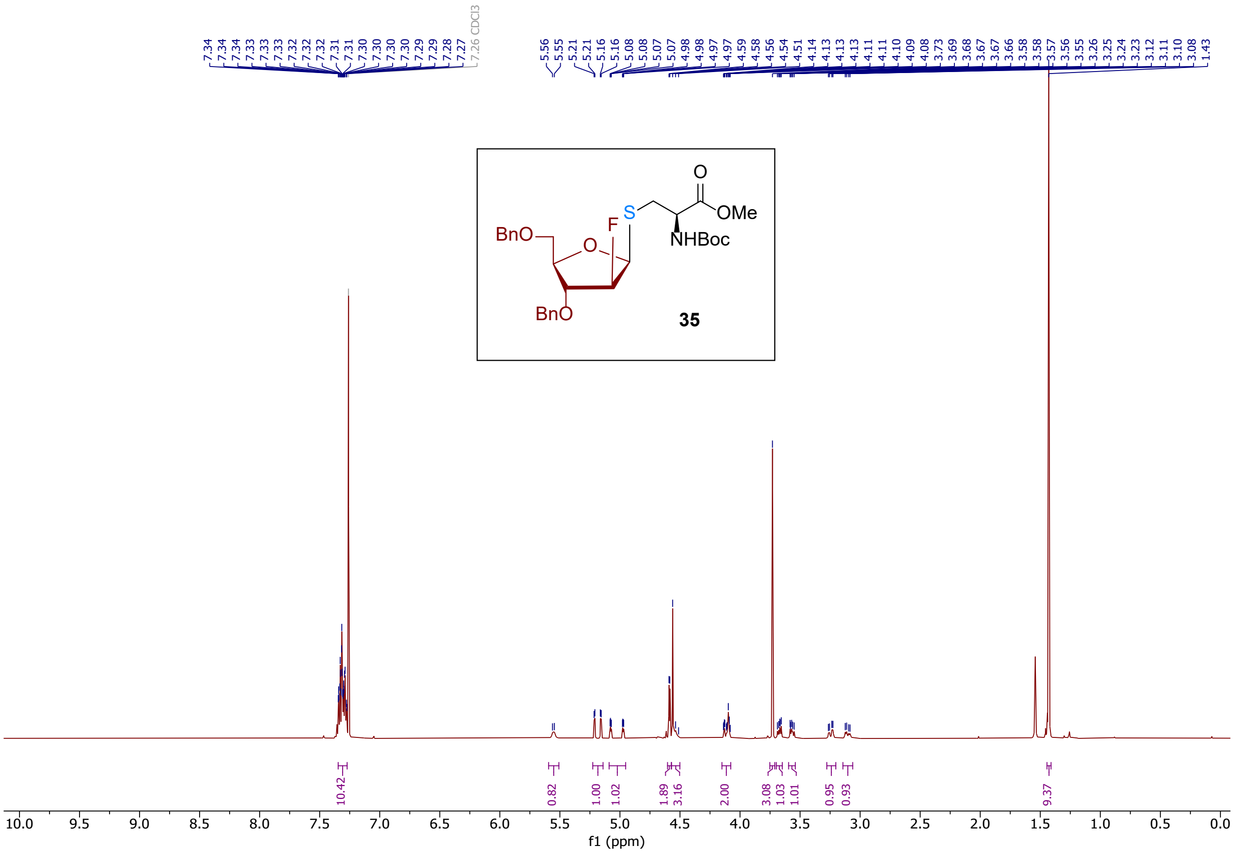

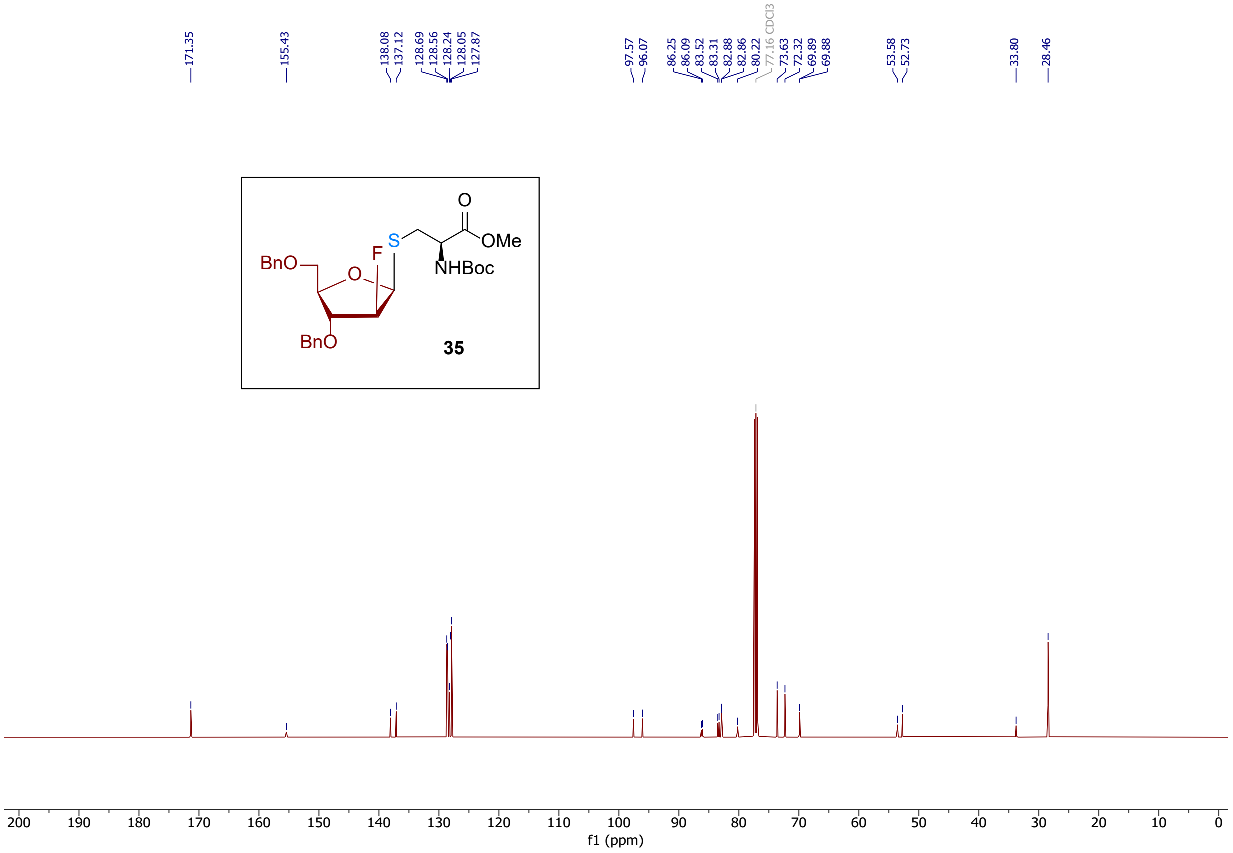

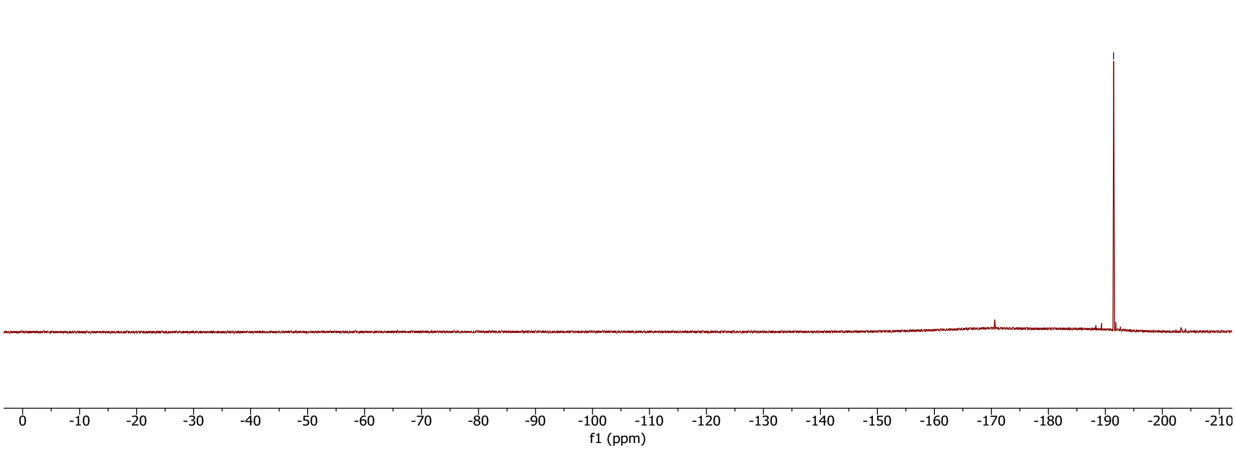
-191.52
f1 (ppm)

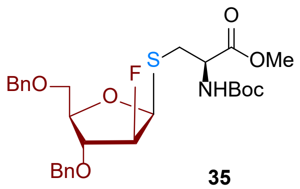
O
OMe
S
F
BnO
NHBoc
BnO

35

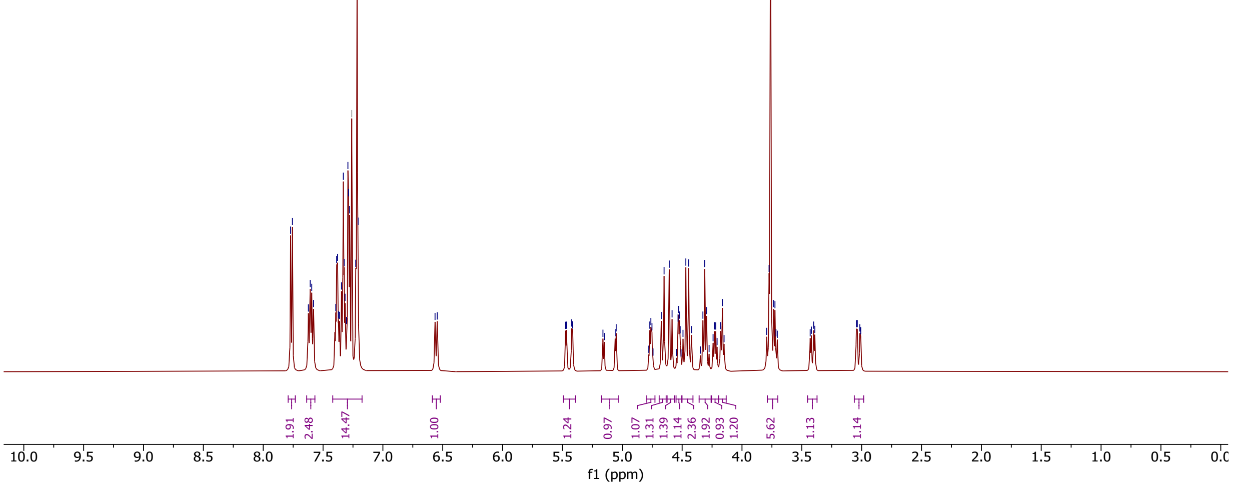
7.26 CDCl3
f1 (ppm)

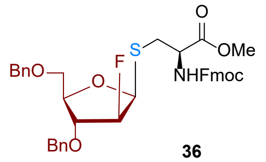
O
OMe
S
F
BnO
NHFmoc
BnO

36

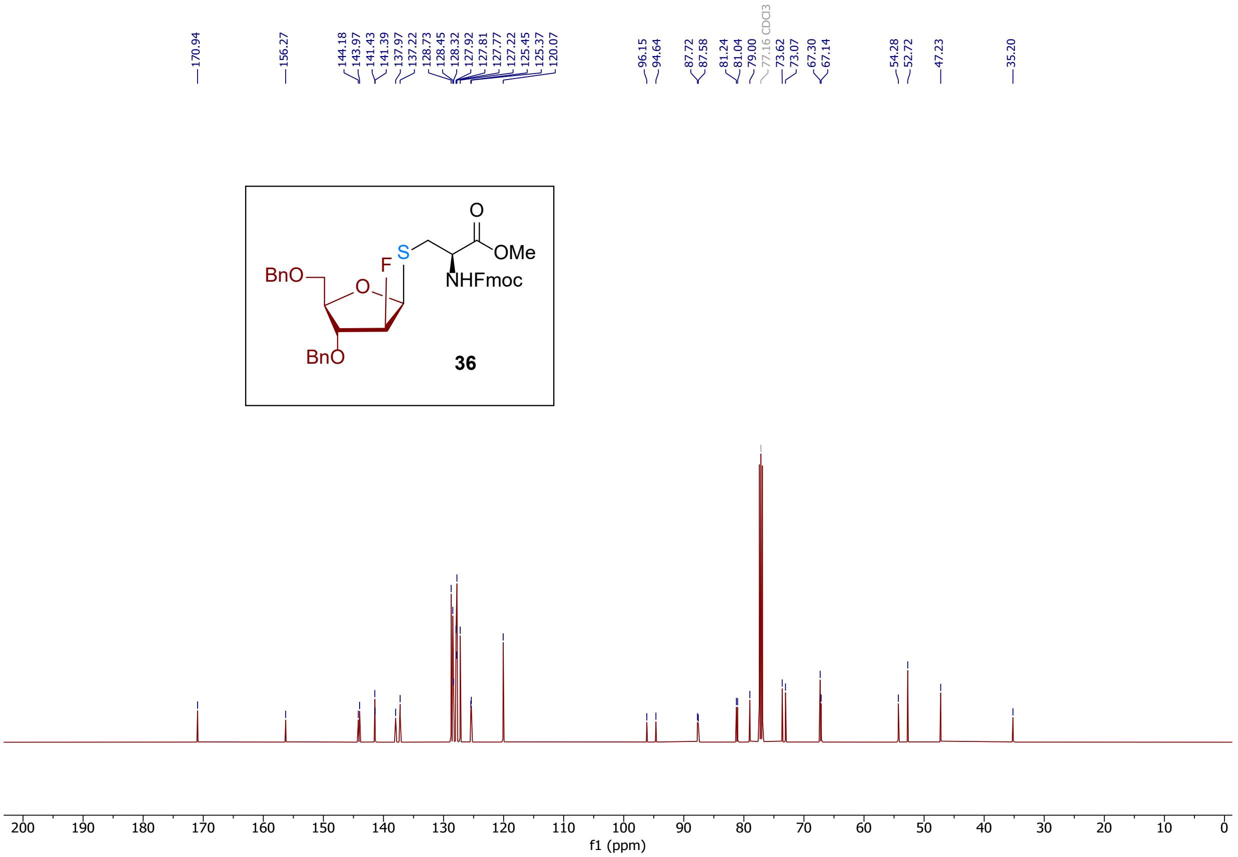
170.94
156.27
144.18
143.97
141.43
141.39
137.97
137.22
128.73
128.45
128.32
127.92
127.81
127.77
127.22
125.45
125.37
120.07
96.15
94.64
87.72
87.58
81.24
81.04
79.00
77.16 CDCl3
73.62
73.07
67.30
67.14
54.28
52.72
47.23
35.20
BnO
O
F
S
O
OMe
NHFmoc
BnO
36
f1 (ppm)

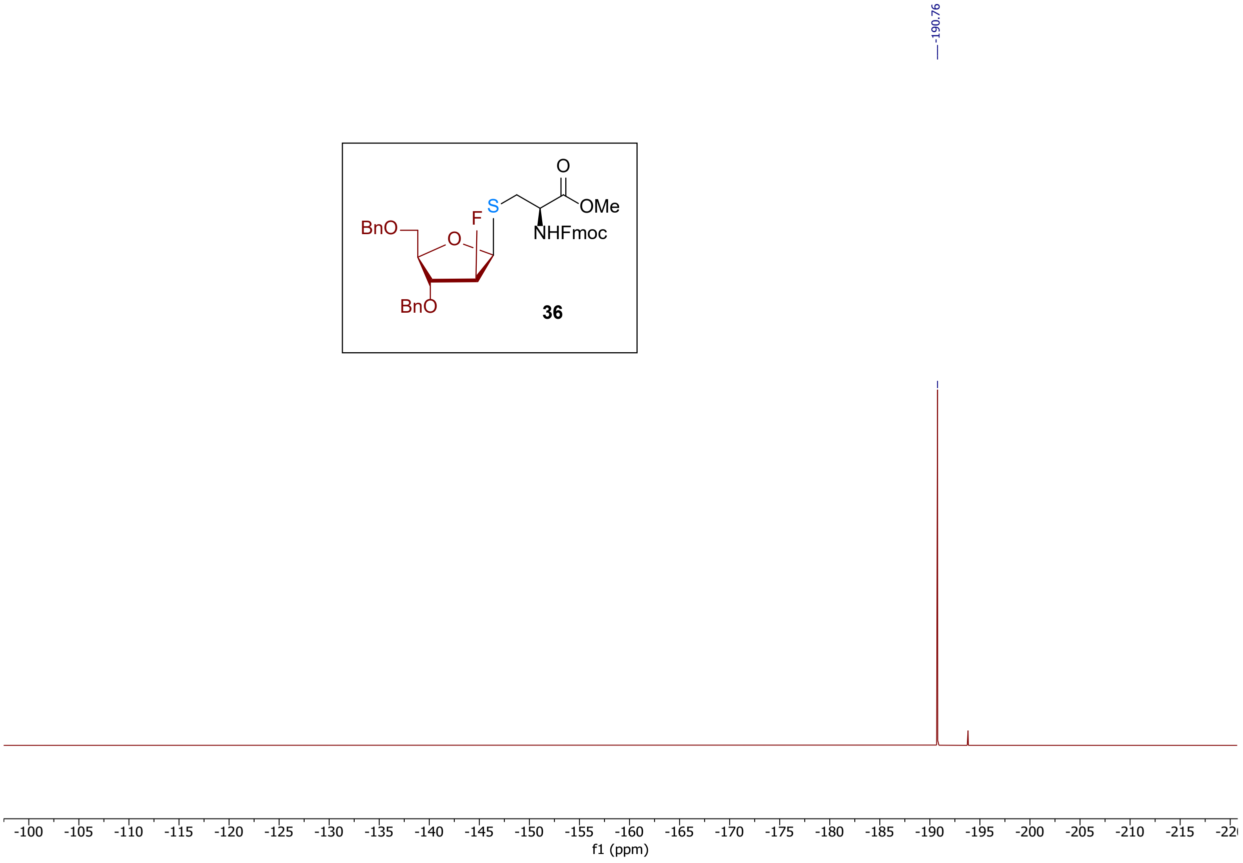
-190.76
BnO
O
F
S
O
OMe
NHFmoc
BnO
36
f1 (ppm)

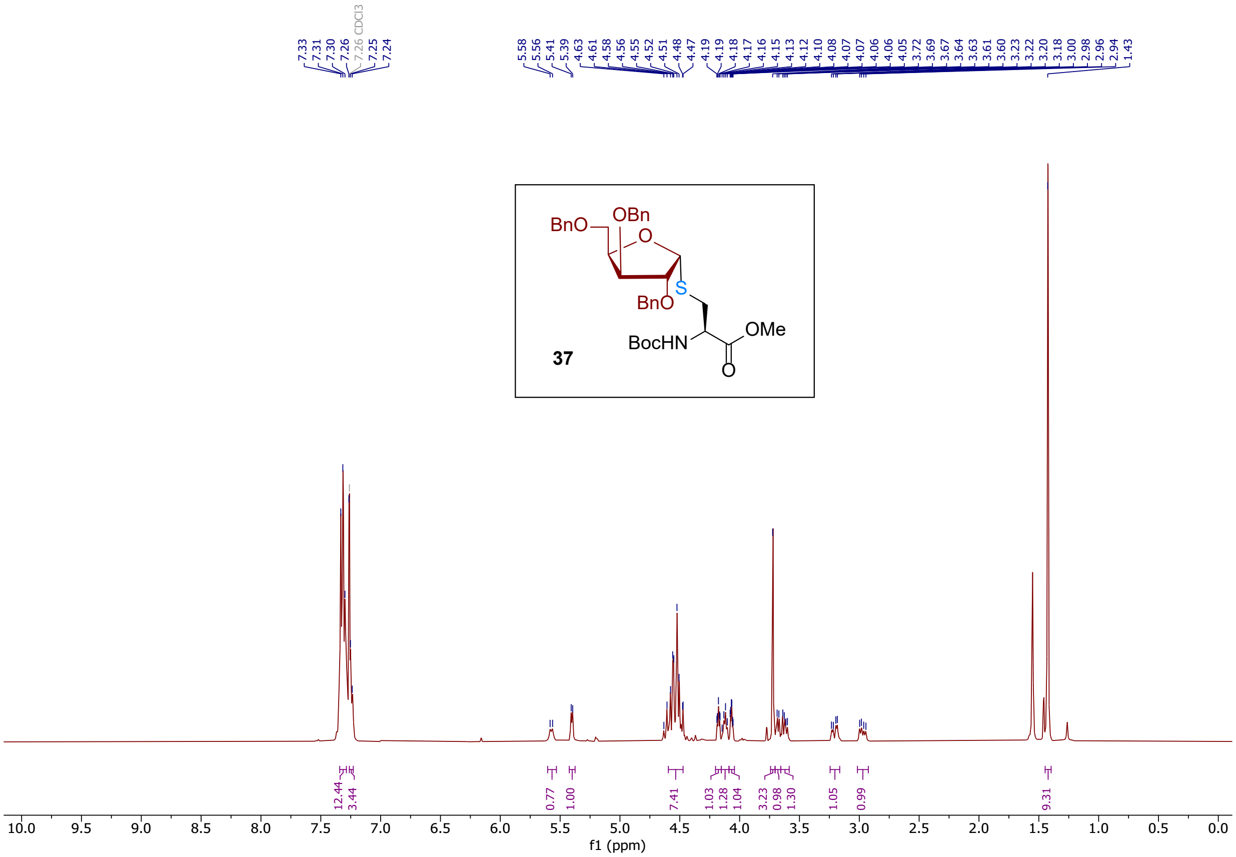
7.33 7.31 7.30 7.26 7.26 CDCl3 7.25 7.24
5.58 5.56 5.41 5.39 4.63 4.61 4.58 4.56 4.55 4.52 4.51 4.48 4.47 4.19 4.19 4.18 4.17 4.16 4.15 4.13 4.12 4.10 4.08 4.07 4.07 4.06 4.06 4.05 3.72 3.69 3.67 3.64 3.63 3.61 3.60 3.23 3.22 3.20 3.18 3.00 2.98 2.96 2.94 1.43
BnO
OBn
O
S
BnO
BocHN
OMe
O
37
12.44 3.44 0.77 1.00 7.41 1.03 1.28 1.04 3.23 0.98 1.30 1.05 0.99 9.31
10.0 9.5 9.0 8.5 8.0 7.5 7.0 6.5 6.0 5.5 5.0 4.5 4.0 3.5 3.0 2.5 2.0 1.5 1.0 0.5 0.0
f1 (ppm)

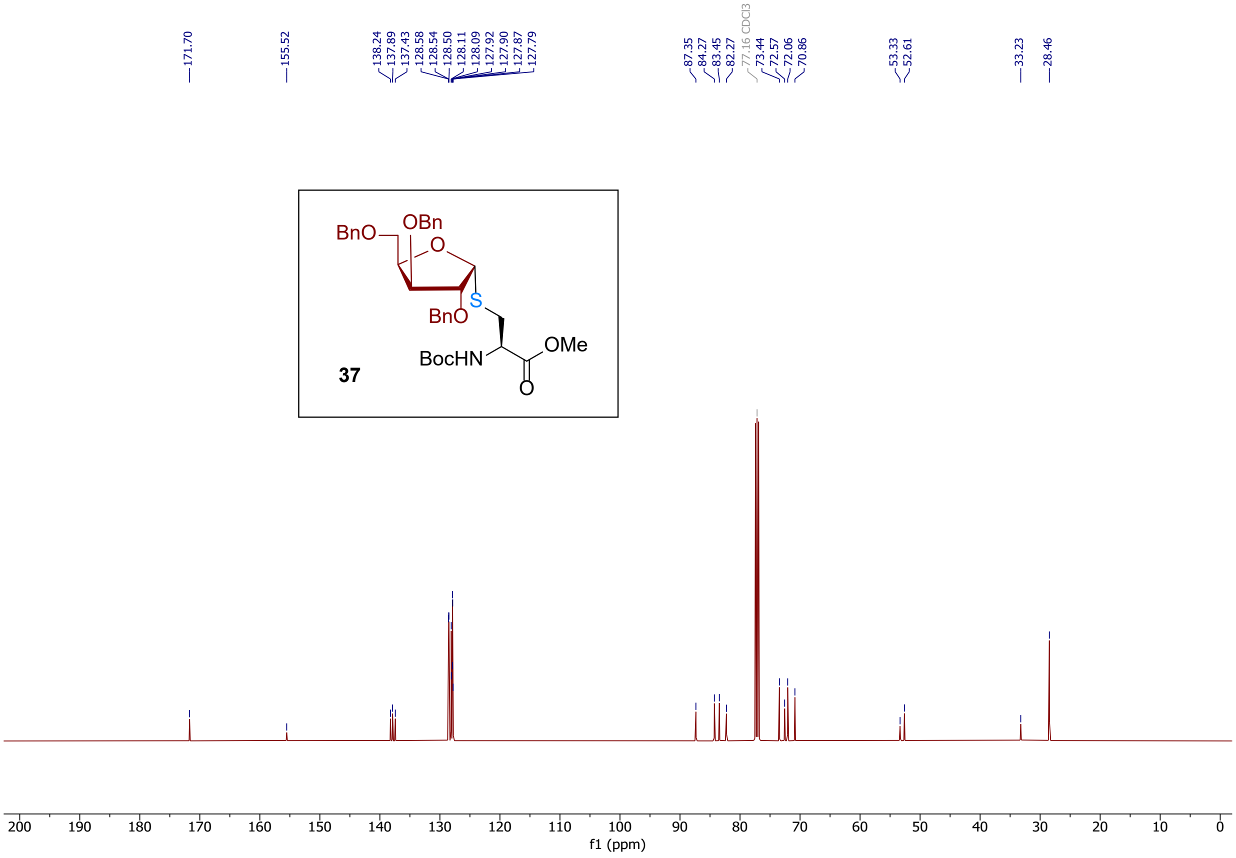
171.70
155.52
138.24 137.89 137.43 128.58 128.54 128.50 128.11 128.09 127.92 127.90 127.87 127.79
87.35 84.27 83.45 82.27 77.16 CDCl3 73.44 72.57 72.06 70.86
53.33 52.61
33.23 28.46
BnO
OBn
O
S
BnO
BocHN
OMe
O
37
200 190 180 170 160 150 140 130 120 110 100 90 80 70 60 50 40 30 20 10 0
f1 (ppm)

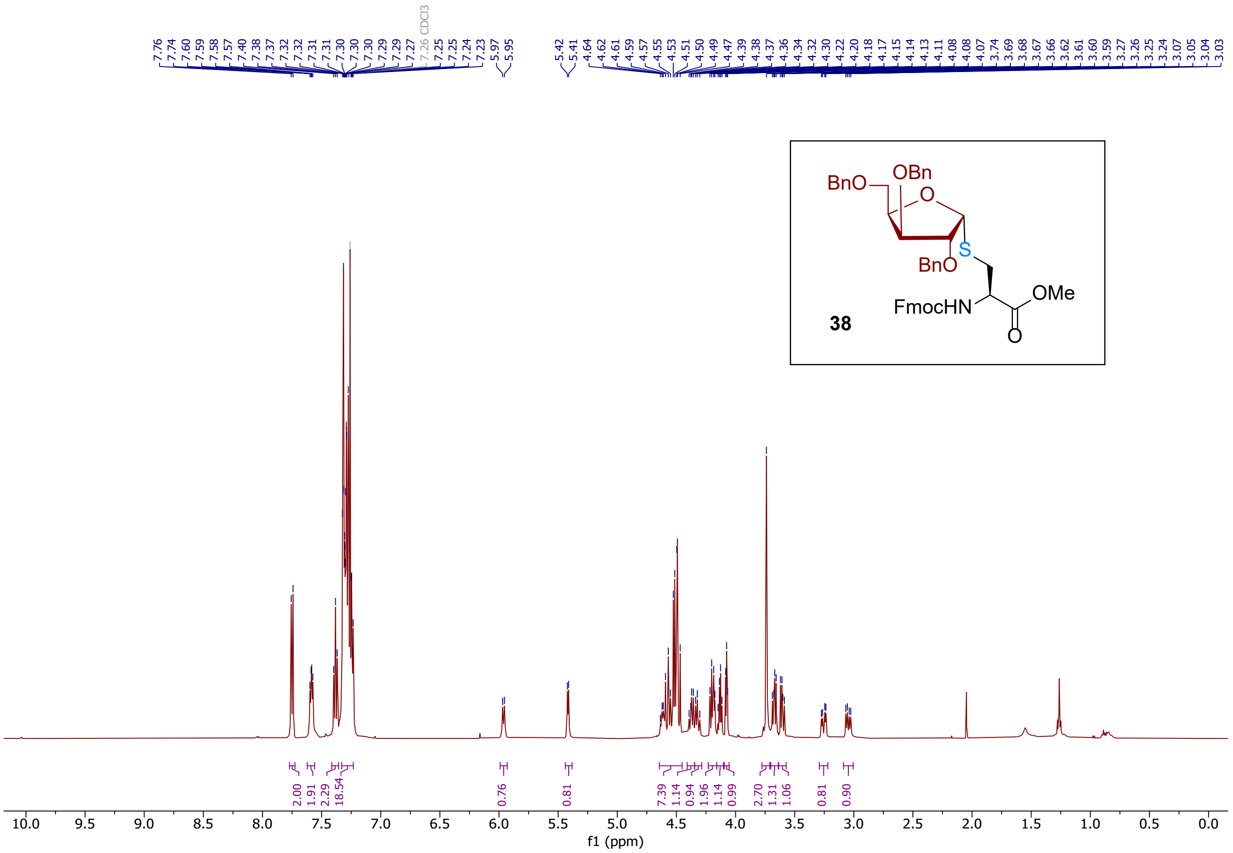
7.76 7.74 7.60 7.59 7.58 7.57 7.40 7.38 7.37 7.32 7.32 7.31 7.31 7.30 7.30 7.30 7.29 7.29 7.27 7.26 CDCl3 7.25 7.25 7.24 7.23 5.97 5.95
5.42 5.41 4.64 4.62 4.61 4.59 4.57 4.55 4.53 4.51 4.50 4.49 4.47 4.39 4.38 4.37 4.36 4.34 4.32 4.30 4.22 4.20 4.18 4.17 4.15 4.14 4.13 4.11 4.08 4.08 4.07 3.74 3.69 3.68 3.67 3.66 3.62 3.61 3.60 3.59 3.27 3.26 3.25 3.24 3.07 3.05 3.04 3.03
BnO
OBn
O
S
BnO
OMe
FmocHN
O
38
2.00 1.91 2.29 18.54 0.76 0.81 7.39 1.14 0.94 1.96 1.14 0.99 2.70 1.31 1.06 0.81 0.90
10.0 9.5 9.0 8.5 8.0 7.5 7.0 6.5 6.0 5.5 5.0 4.5 4.0 3.5 3.0 2.5 2.0 1.5 1.0 0.5 0.0
f1 (ppm)

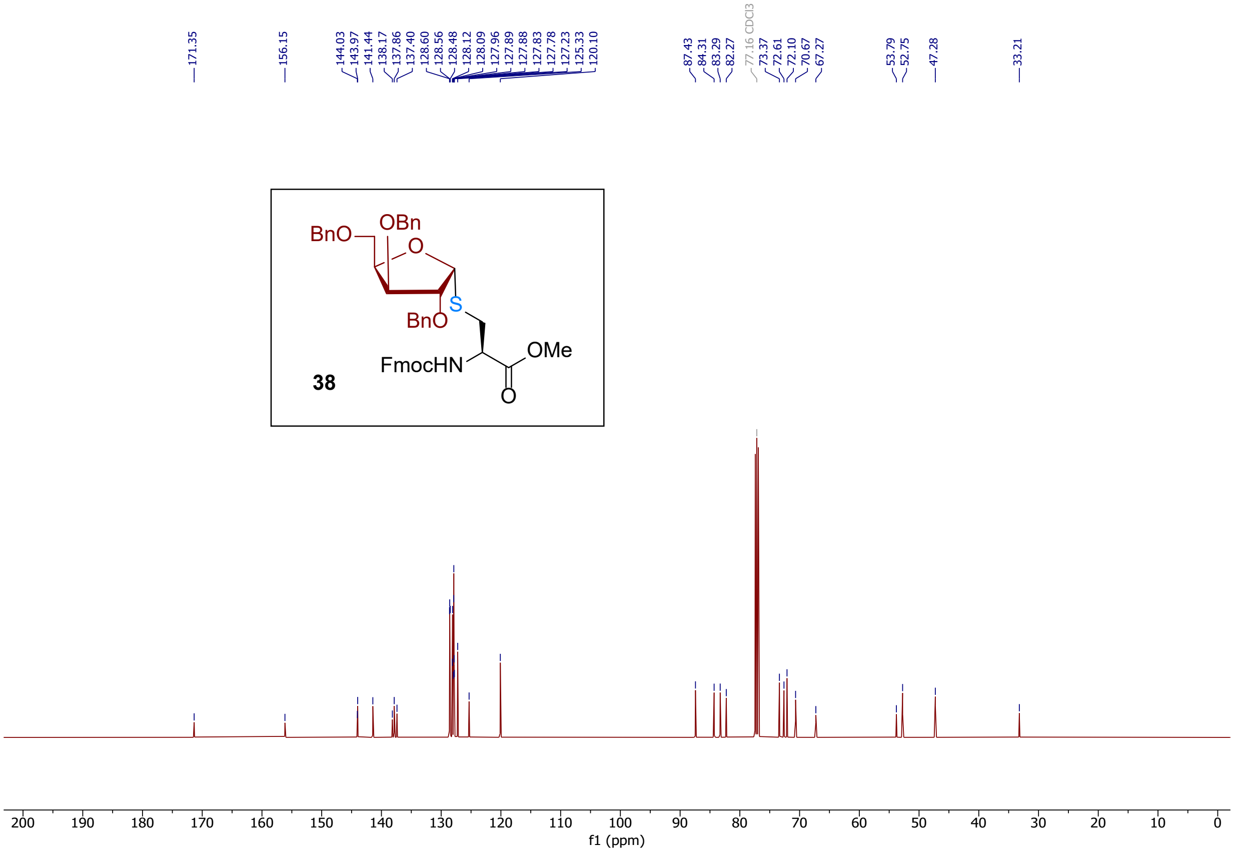
171.35 156.15 144.03 143.97 141.44 138.17 137.86 137.40 128.60 128.56 128.48 128.12 128.09 127.96 127.89 127.88 127.83 127.78 127.23 125.33 120.10
87.43 84.31 83.29 82.27 77.16 CDCl3 73.37 72.61 72.10 70.67 67.27 53.79 52.75 47.28 33.21
BnO
OBn
O
S
BnO
OMe
FmocHN
O
38
200 190 180 170 160 150 140 130 120 110 100 90 80 70 60 50 40 30 20 10 0
f1 (ppm)

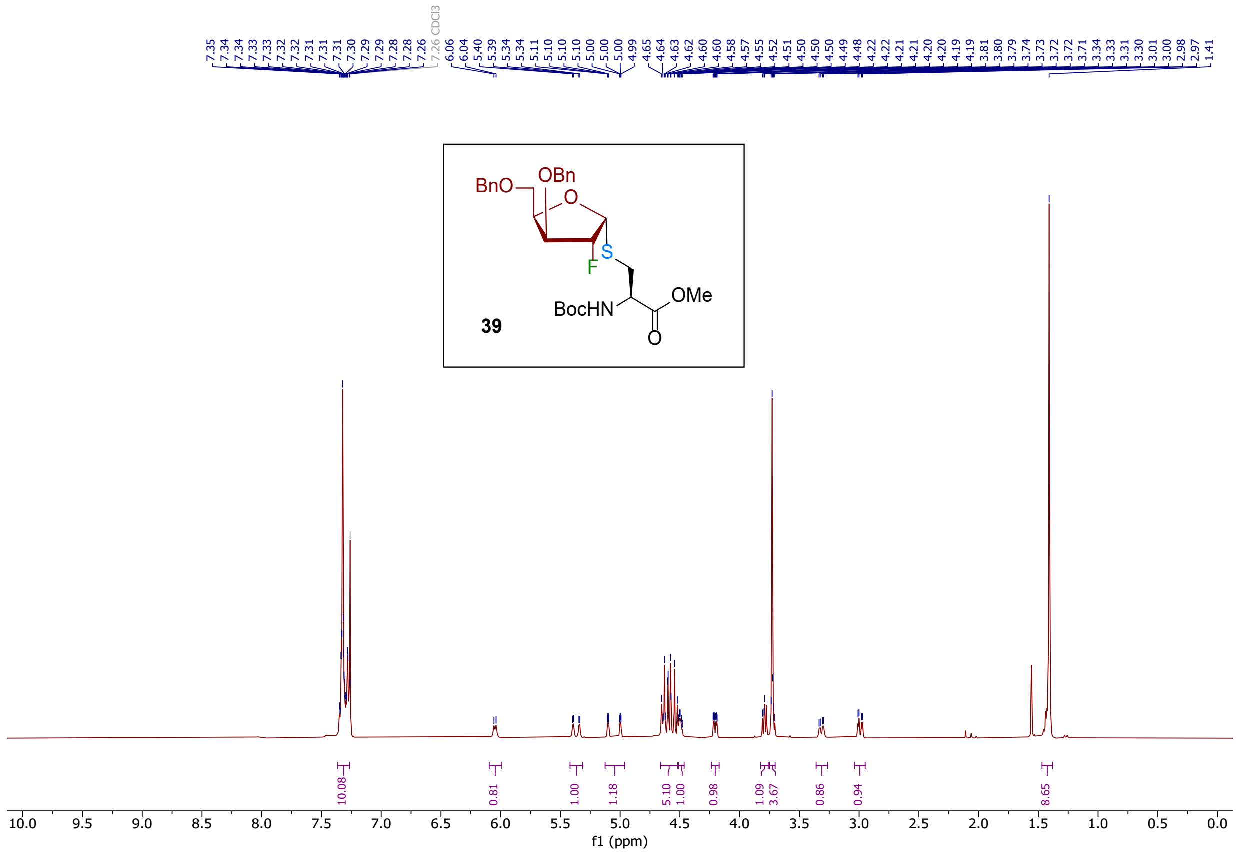
7.35 7.34 7.34 7.33 7.33 7.32 7.32 7.31 7.31 7.31 7.30 7.29 7.29 7.28 7.28 7.26 7.26 CDCl3 6.06 6.04 5.40 5.39 5.34 5.34 5.11 5.10 5.10 5.00 5.00 5.00 4.99 4.65 4.64 4.63 4.62 4.60 4.60 4.58 4.57 4.55 4.52 4.51 4.50 4.50 4.50 4.49 4.48 4.22 4.21 4.21 4.20 4.20 4.19 4.19 3.81 3.80 3.79 3.74 3.73 3.72 3.72 3.71 3.34 3.33 3.31 3.30 3.01 3.00 2.98 2.97 1.41
BnO
OBn
O
S
F
BocHN
OMe
O
39
10.08
0.81
1.00
1.18
5.10
1.00
0.98
1.09
3.67
0.86
0.94
8.65
f1 (ppm)

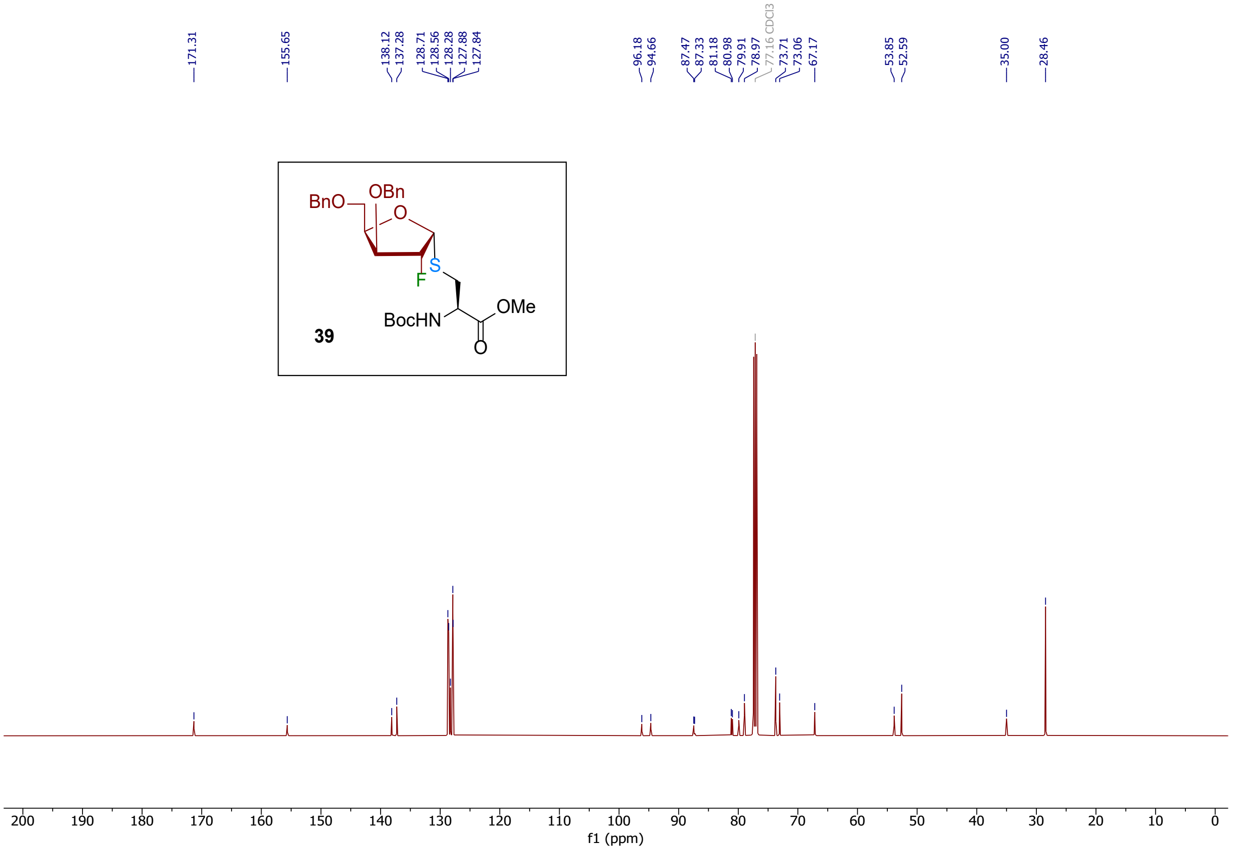
171.31 155.65 138.12 137.28 128.71 128.56 128.28 127.88 127.84 96.18 94.66 87.47 87.33 81.18 80.98 79.91 78.97 77.16 CDCl3 73.71 73.06 67.17 53.85 52.59 35.00 28.46
BnO
OBn
O
S
F
BocHN
OMe
O
39
f1 (ppm)

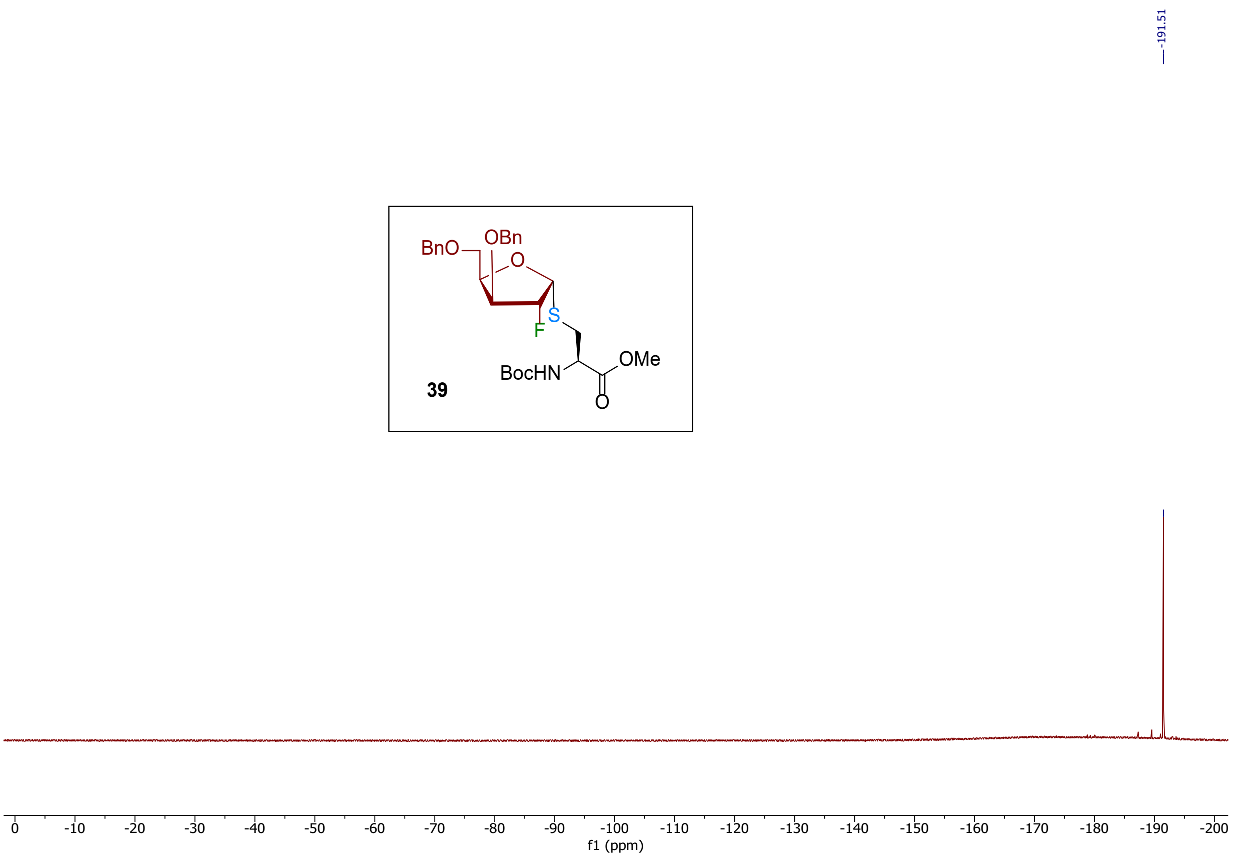

-191.51
BnO
OBn
O
S
F
BocHN
OMe
O
39
f1 (ppm)

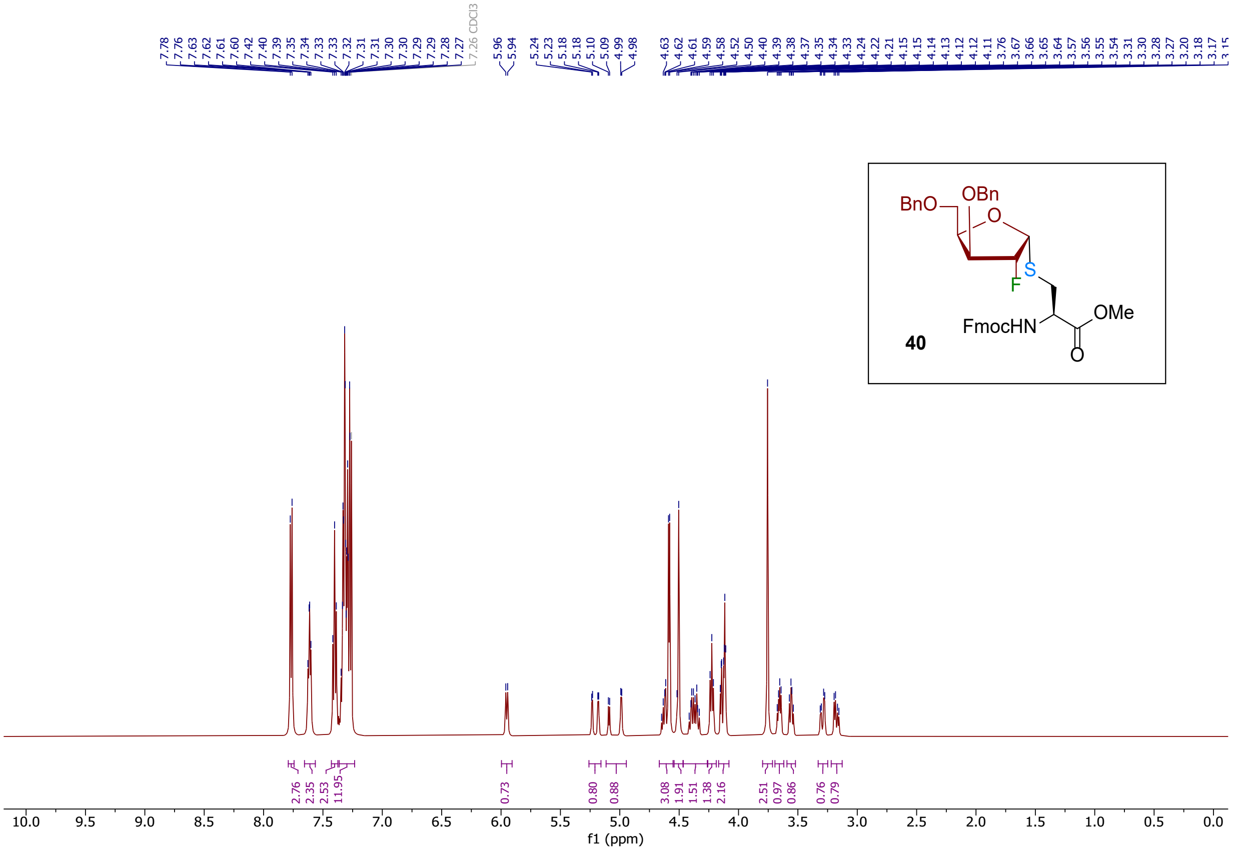

7.26 CDCl3
BnO
OBn
O
S
F
FmocHN
OMe
O
40
2.76
2.35
2.53
11.95
0.73
0.80
0.88
3.08
1.91
1.51
1.38
2.16
2.51
0.97
0.86
0.76
0.79
f1 (ppm)

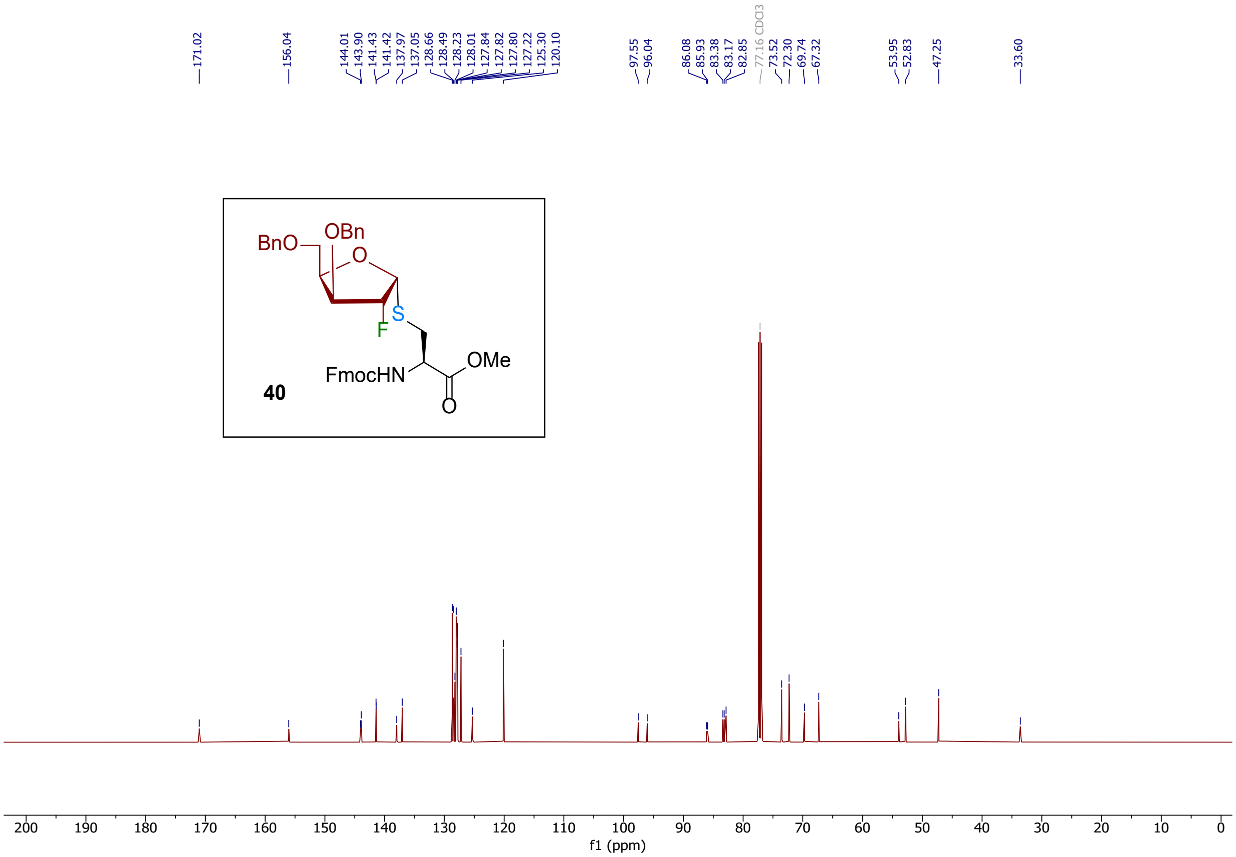
171.02
156.04
144.01
143.90
141.43
141.42
137.97
137.05
128.66
128.49
128.23
128.01
127.84
127.82
127.80
127.22
125.30
120.10
97.55
96.04
86.08
85.93
83.38
83.17
82.85
77.16 CDCl3
73.52
72.30
69.74
67.32
53.95
52.83
47.25
33.60
BnO
OBn
O
S
F
FmocHN
OMe
O
40
f1 (ppm)

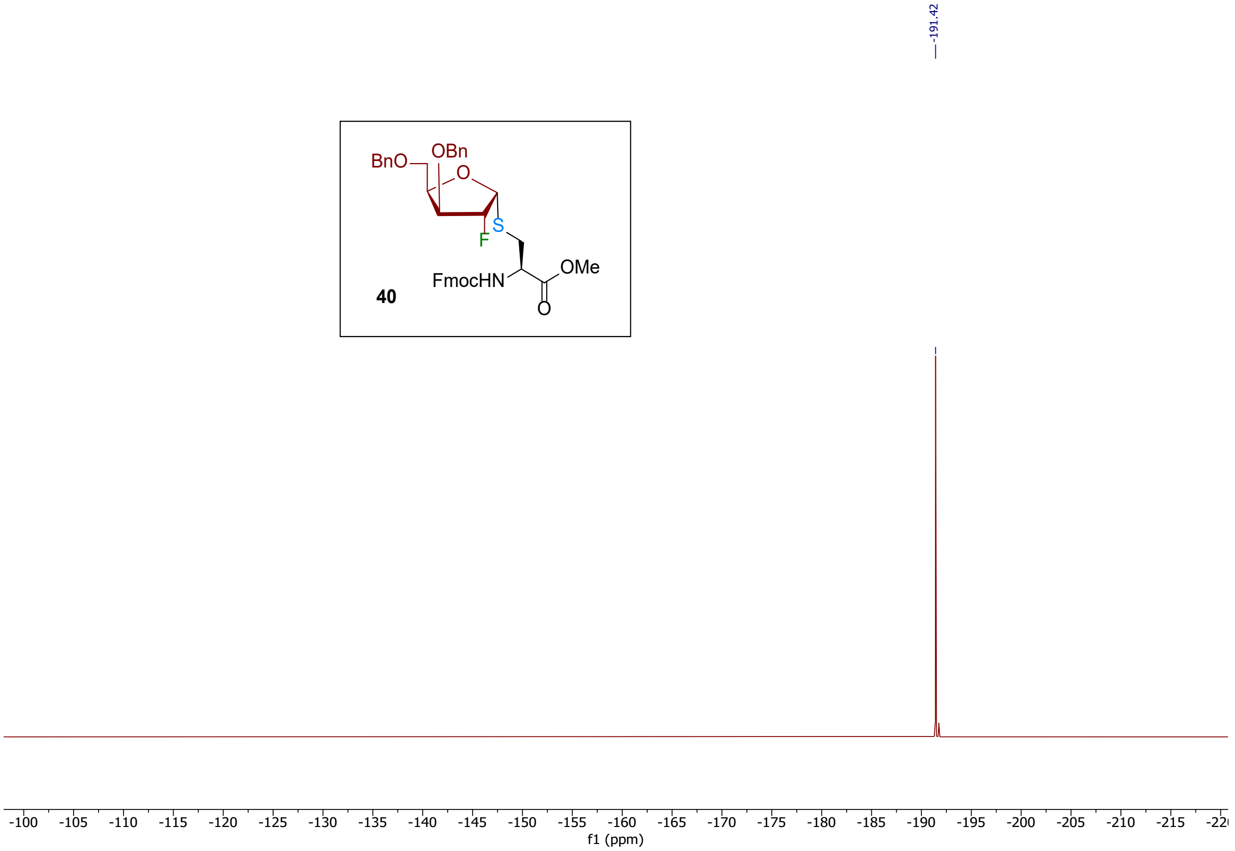
-191.42
BnO
OBn
O
S
F
FmocHN
OMe
O
40
f1 (ppm)

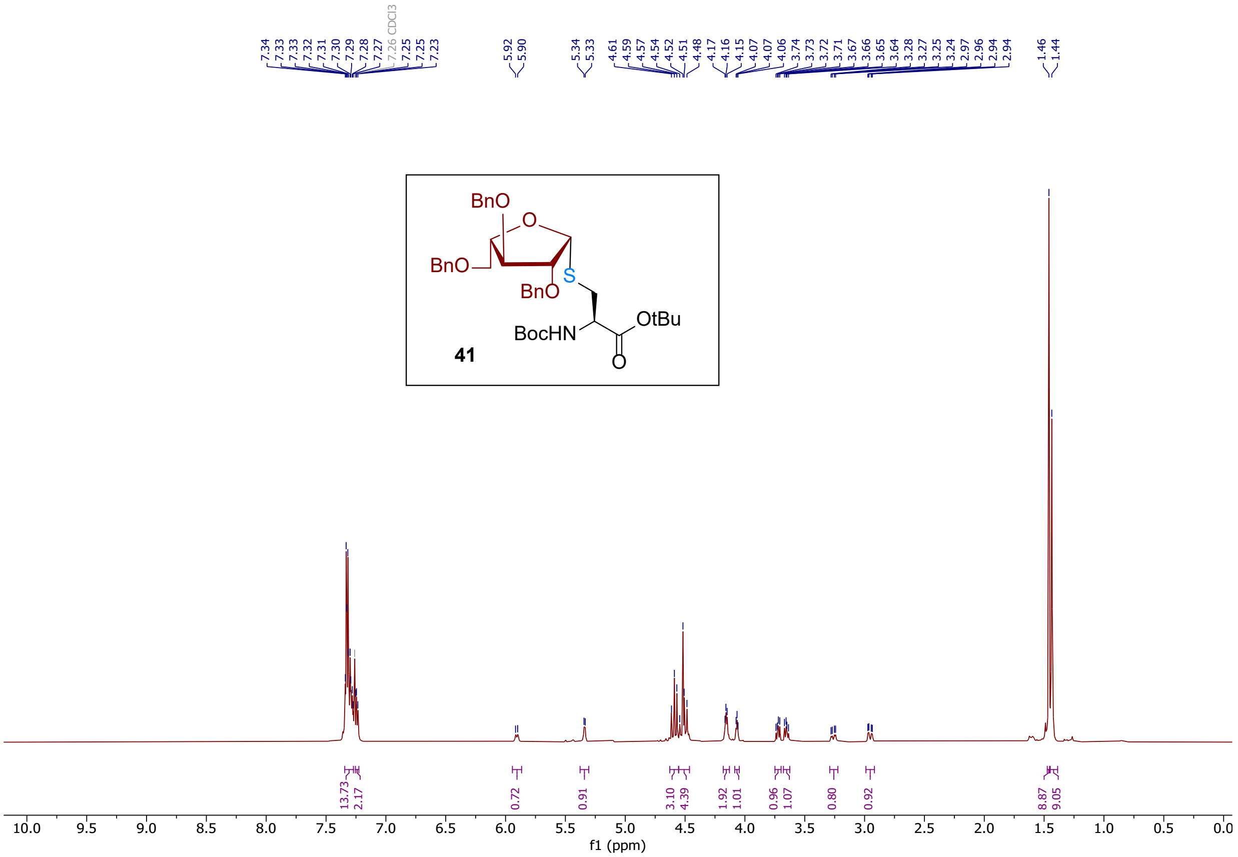
7.34 7.33 7.33 7.32 7.31 7.30 7.29 7.28 7.27 7.26 CDCl3 7.25 7.25 7.23
5.92 5.90
5.34 5.33
4.61 4.59 4.57 4.54 4.52 4.51 4.48 4.17 4.16 4.15 4.07 4.07 4.06 3.74 3.73 3.72 3.71 3.67 3.66 3.65 3.64 3.28 3.27 3.25 3.24 2.97 2.96 2.94 2.94
1.46 1.44
BnO
BnO
BnO
S
OtBu
BocHN
O
41
13.73 2.17 0.72 0.91 3.10 4.39 1.92 1.01 0.96 1.07 0.80 0.92 8.87 9.05
f1 (ppm)

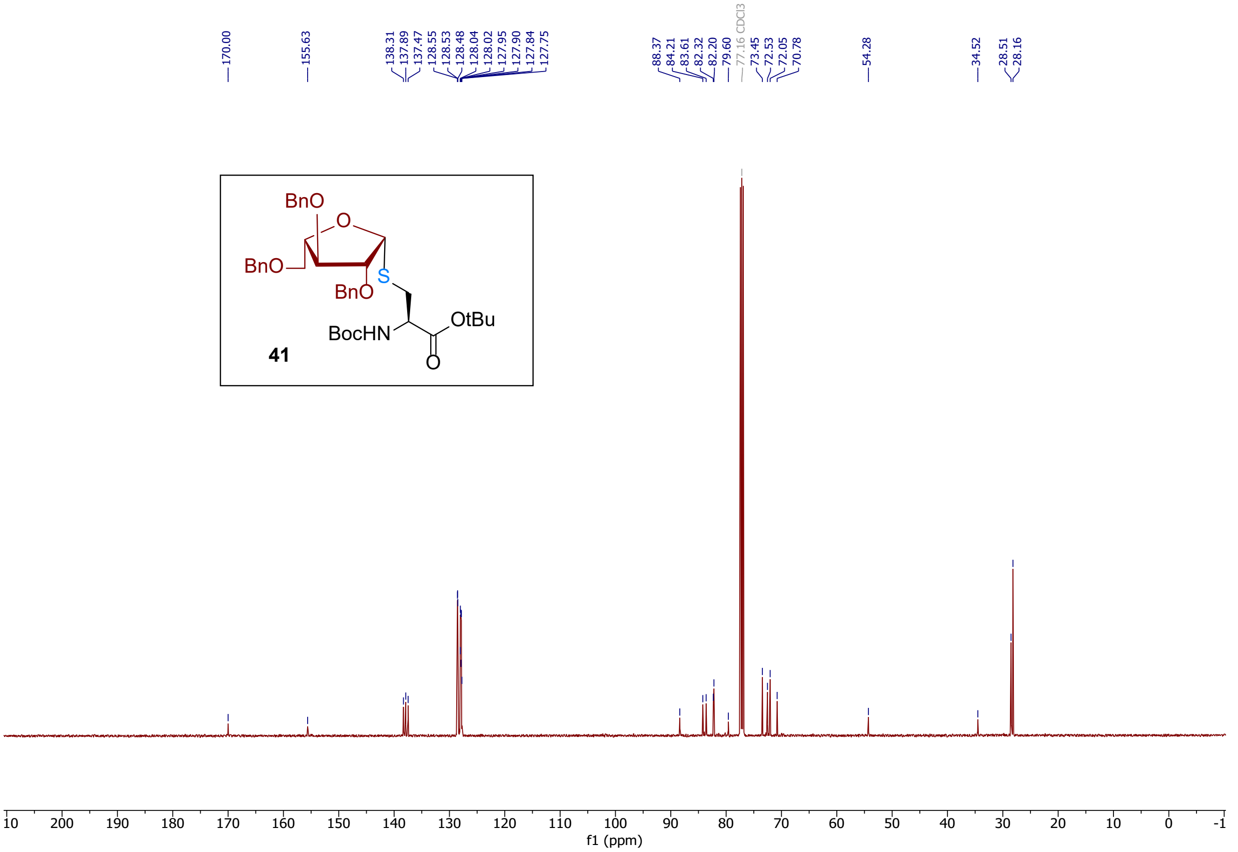
170.00
155.63
138.31 137.89 137.47 128.55 128.53 128.48 128.04 128.02 127.95 127.90 127.84 127.75
88.37 84.21 83.61 82.32 82.20 79.60 77.16 CDCl3 73.45 72.53 72.05 70.78
54.28
34.52
28.51 28.16
BnO
BnO
BnO
S
OtBu
BocHN
O
41
f1 (ppm)

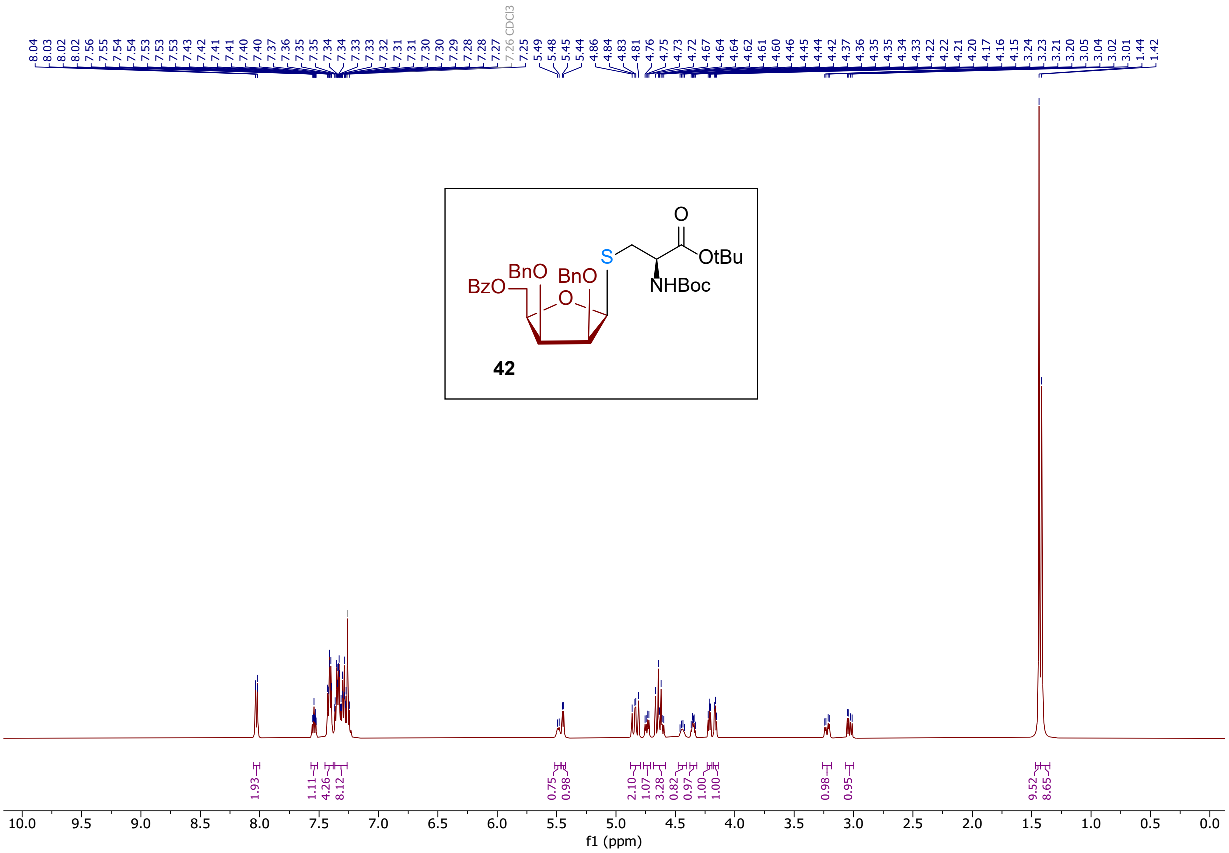
8.04 8.03 8.02 7.56 7.55 7.54 7.54 7.53 7.53 7.53 7.43 7.42 7.41 7.41 7.40 7.37 7.36 7.35 7.35 7.34 7.34 7.33 7.33 7.32 7.31 7.31 7.30 7.30 7.29 7.28 7.27 7.26 CDCl3 7.25 5.49 5.48 5.45 5.44 4.86 4.84 4.83 4.81 4.76 4.75 4.73 4.72 4.67 4.64 4.64 4.62 4.61 4.60 4.46 4.45 4.44 4.42 4.37 4.36 4.35 4.35 4.34 4.33 4.22 4.22 4.21 4.20 4.17 4.16 4.15 3.24 3.23 3.21 3.20 3.05 3.04 3.02 3.01 1.44 1.42
O
S
OtBu
BzO
BnO
BnO
O
NHBoc
42
1.93 1.11 4.26 8.12 0.75 0.98 2.10 1.07 3.28 0.82 0.97 1.00 1.00 0.98 0.95 9.52 8.65
10.0 9.5 9.0 8.5 8.0 7.5 7.0 6.5 6.0 5.5 5.0 4.5 4.0 3.5 3.0 2.5 2.0 1.5 1.0 0.5 0.0
f1 (ppm)

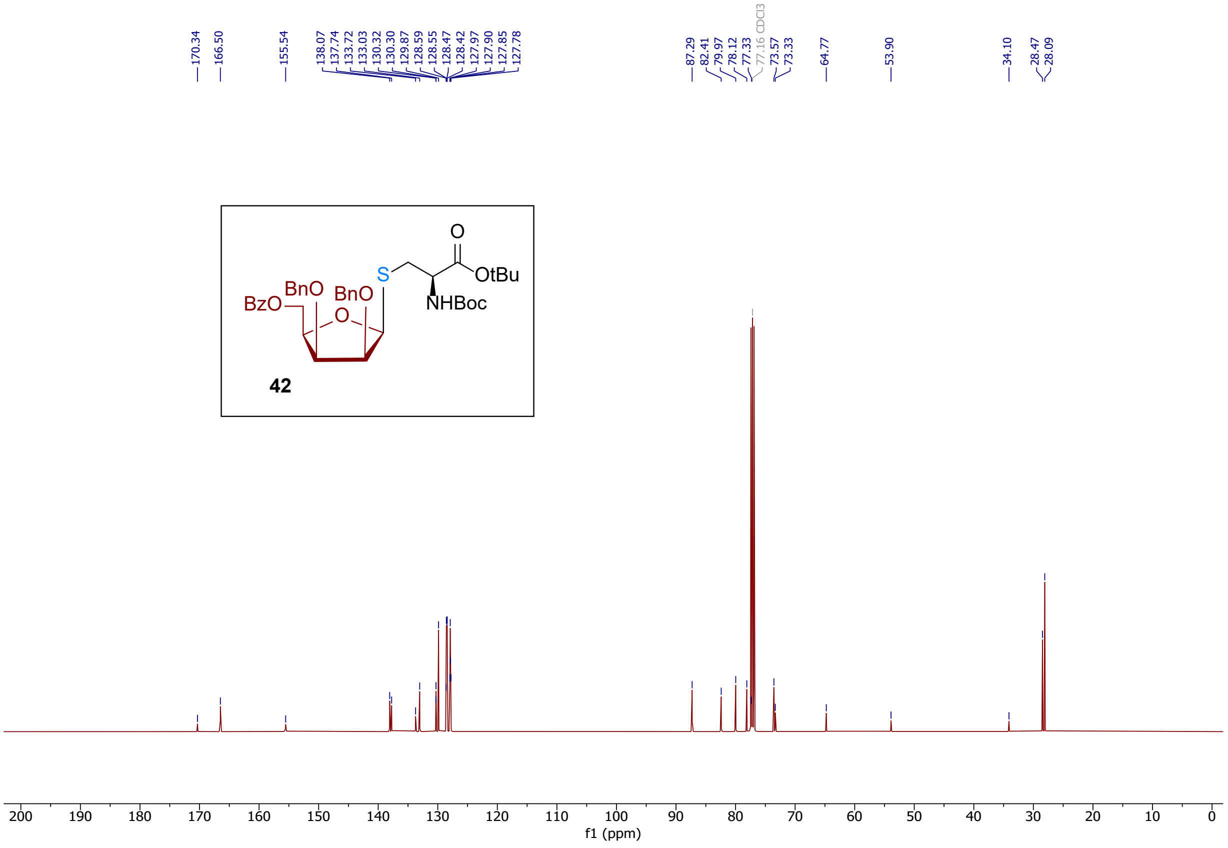
170.34 166.50 155.54 138.07 137.74 133.72 133.03 130.32 130.30 129.87 128.59 128.55 128.47 128.42 127.97 127.90 127.85 127.78 87.29 82.41 79.97 78.12 77.33 77.16 CDCl3 73.57 73.33 64.77 53.90 34.10 28.47 28.09
O
S
OtBu
BzO
BnO
BnO
O
NHBoc
42
200 190 180 170 160 150 140 130 120 110 100 90 80 70 60 50 40 30 20 10 0
f1 (ppm)

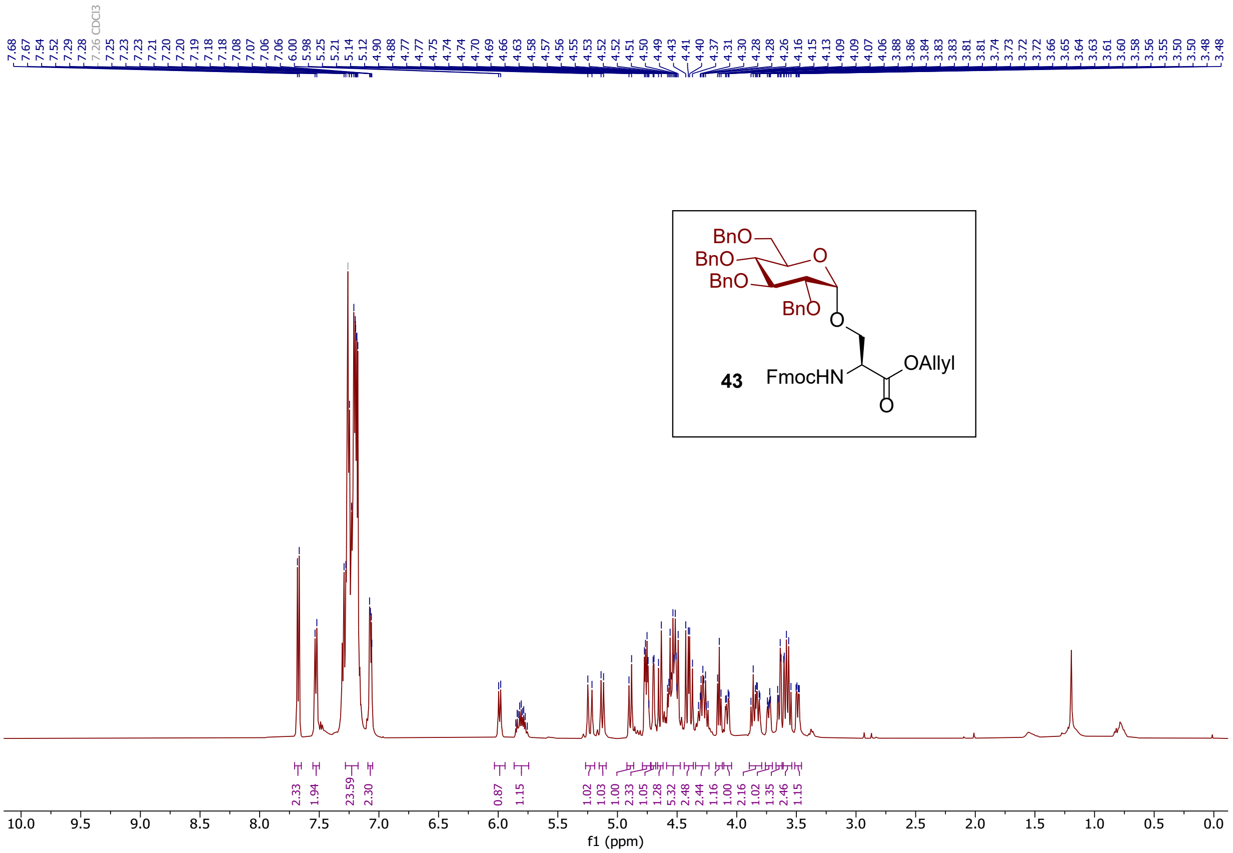
7.68 7.67 7.54 7.52 7.29 7.28 7.26 CDCl3 7.25 7.23 7.21 7.20 7.20 7.19 7.18 7.18 7.08 7.07 7.06 7.06 6.00 5.98 5.25 5.21 5.14 5.12 4.90 4.88 4.77 4.77 4.75 4.74 4.74 4.70 4.69 4.66 4.63 4.58 4.57 4.56 4.55 4.53 4.52 4.52 4.51 4.50 4.49 4.43 4.41 4.40 4.37 4.31 4.30 4.28 4.28 4.26 4.16 4.15 4.13 4.09 4.09 4.07 4.06 3.88 3.86 3.84 3.83 3.83 3.81 3.81 3.74 3.73 3.72 3.72 3.66 3.65 3.64 3.63 3.61 3.60 3.58 3.56 3.55 3.50 3.50 3.48 3.48
BnO
BnO
BnO
BnO
O
O
43
FmocHN
OAllyl
O
2.33 1.94 23.59 2.30 0.87 1.15 1.02 1.03 1.00 2.33 1.05 1.28 5.32 2.48 2.44 1.16 1.00 2.16 1.02 1.35 2.46 1.15
10.0 9.5 9.0 8.5 8.0 7.5 7.0 6.5 6.0 5.5 5.0 4.5 4.0 3.5 3.0 2.5 2.0 1.5 1.0 0.5 0.0
f1 (ppm)

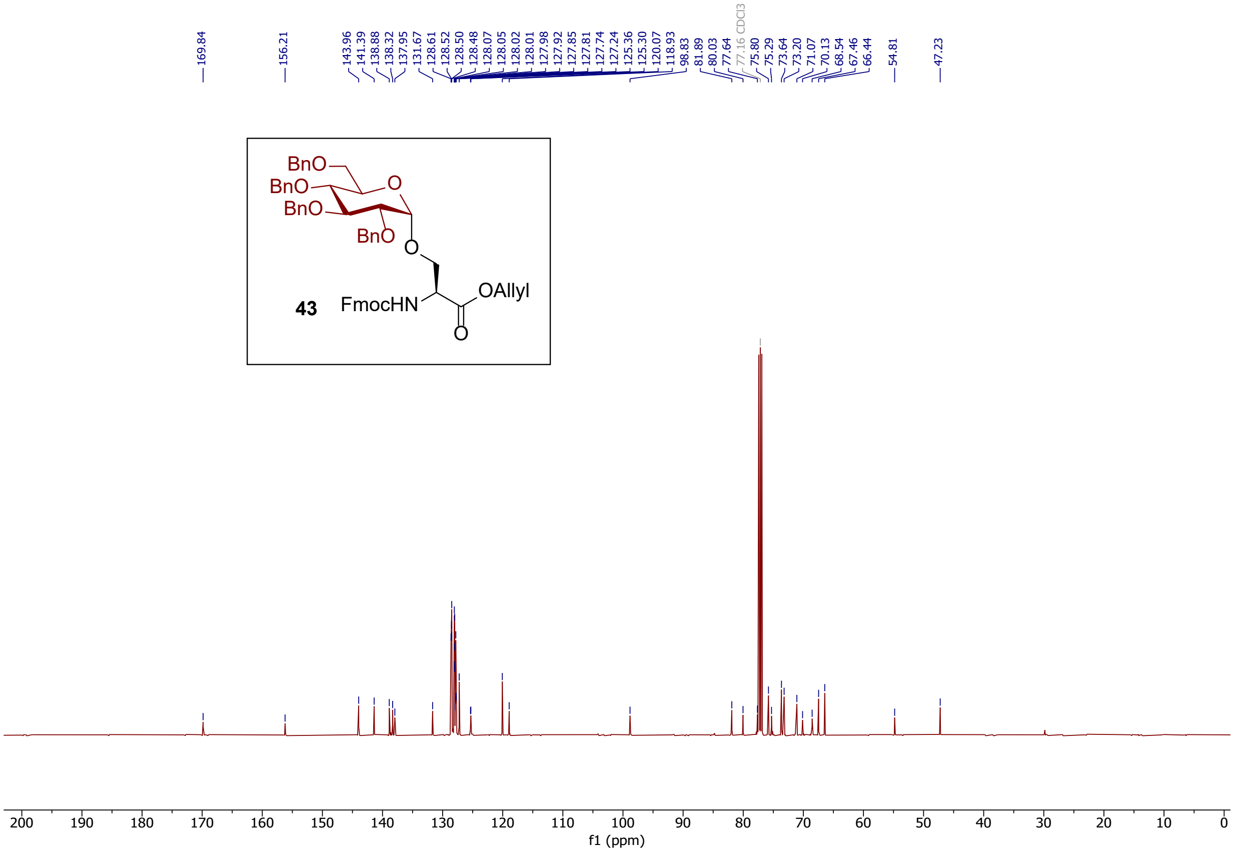
169.84 156.21 143.96 141.39 138.88 138.32 137.95 131.67 128.61 128.52 128.50 128.48 128.07 128.05 128.02 128.01 127.98 127.92 127.85 127.81 127.74 127.24 125.36 125.30 120.07 118.93 98.83 81.89 80.03 77.64 77.16 CDCl3 75.80 75.29 73.64 73.20 71.07 70.13 68.54 67.46 66.44 54.81 47.23
BnO
BnO
BnO
BnO
O
O
43
FmocHN
OAllyl
O
200 190 180 170 160 150 140 130 120 110 100 90 80 70 60 50 40 30 20 10 0
f1 (ppm)

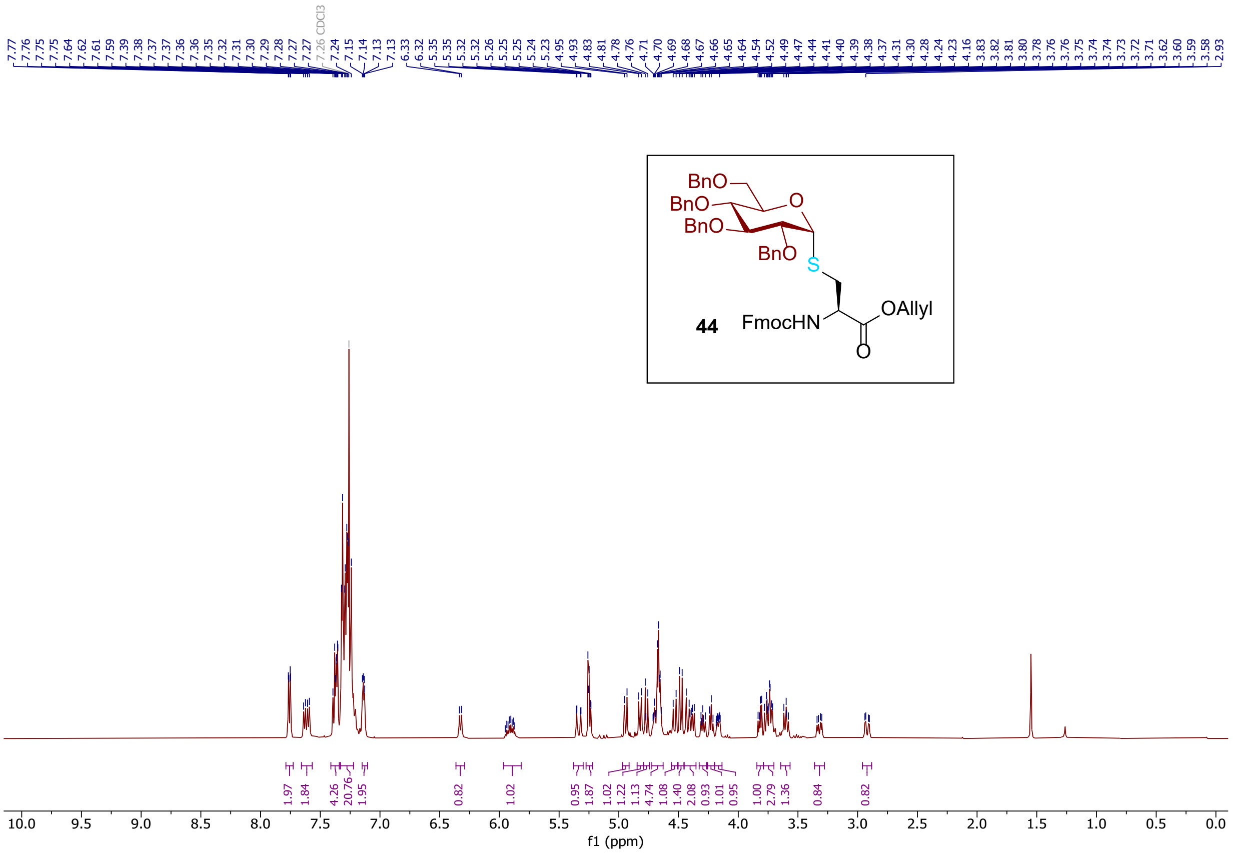
BnO
BnO
BnO
O
BnO
S
44
FmocHN
OAllyl
O
f1 (ppm)

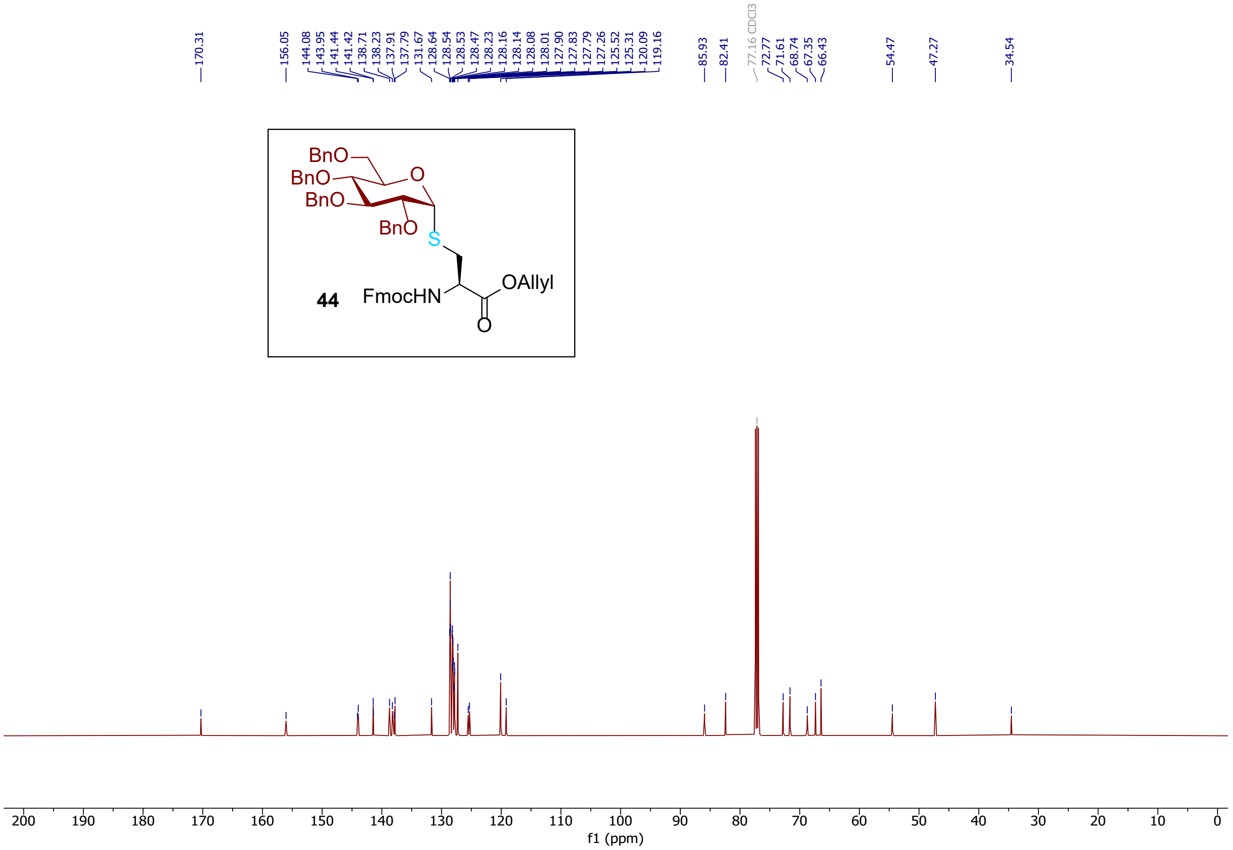
BnO
BnO
BnO
O
BnO
S
44
FmocHN
OAllyl
O
f1 (ppm)

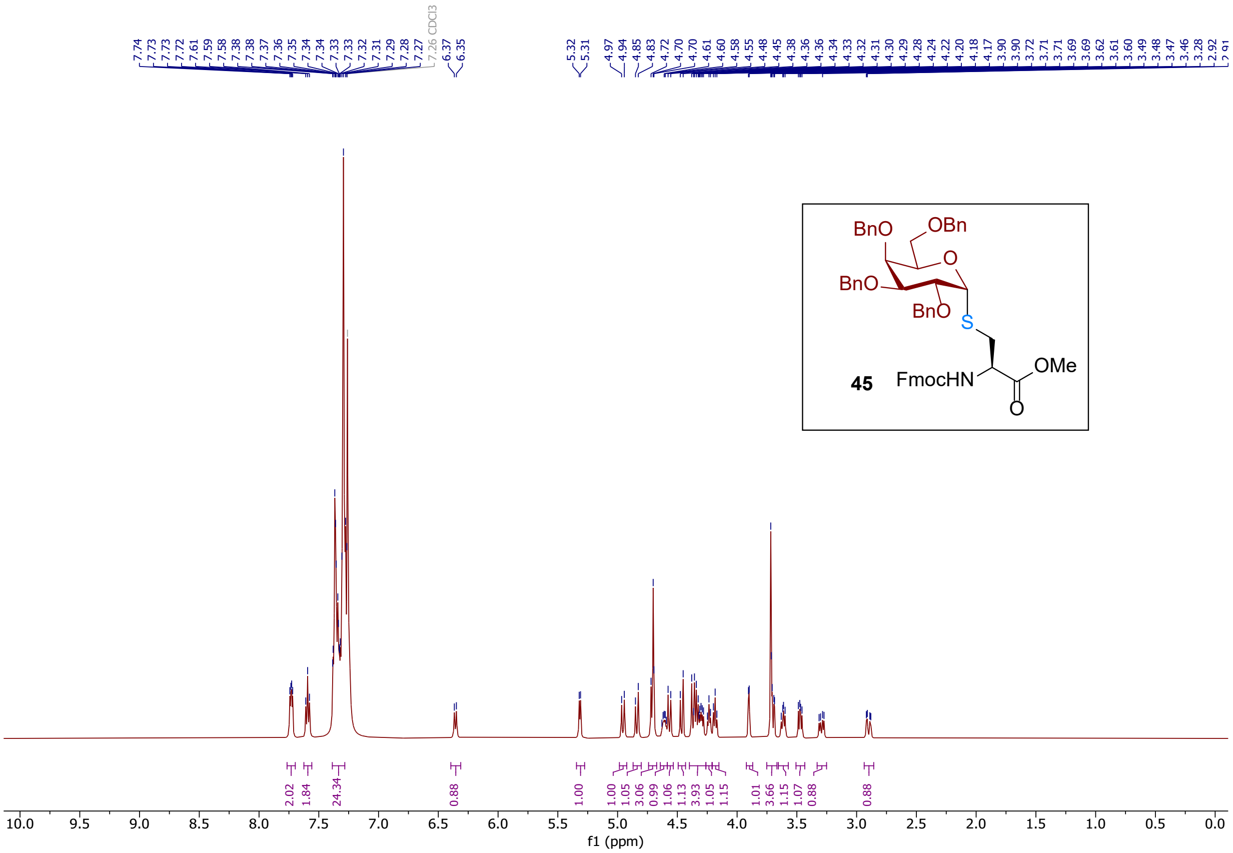

7.74 7.73 7.73 7.72 7.61 7.59 7.58 7.38 7.38 7.37 7.36 7.35 7.34 7.34 7.33 7.33 7.32 7.31 7.29 7.28 7.27
7.26 CDCl3
6.37 6.35
5.32 5.31 4.97 4.94 4.85 4.83 4.72 4.70 4.70 4.61 4.60 4.58 4.55 4.48 4.45 4.38 4.36 4.36 4.34 4.33 4.32 4.31 4.30 4.29 4.28 4.24 4.22 4.20 4.18 4.17 3.90 3.90 3.72 3.71 3.71 3.69 3.69 3.62 3.61 3.60 3.49 3.48 3.47 3.46 3.28 2.92
BnO
OBn
O
BnO
BnO
S
OMe
45
FmocHN
O
2.02 1.84 24.34 0.88 1.00 1.00 1.05 3.06 0.99 1.06 1.13 3.93 1.05 1.15 1.01 3.66 1.15 1.07 0.88 0.88
10.0 9.5 9.0 8.5 8.0 7.5 7.0 6.5 6.0 5.5 5.0 4.5 4.0 3.5 3.0 2.5 2.0 1.5 1.0 0.5 0.0
f1 (ppm)

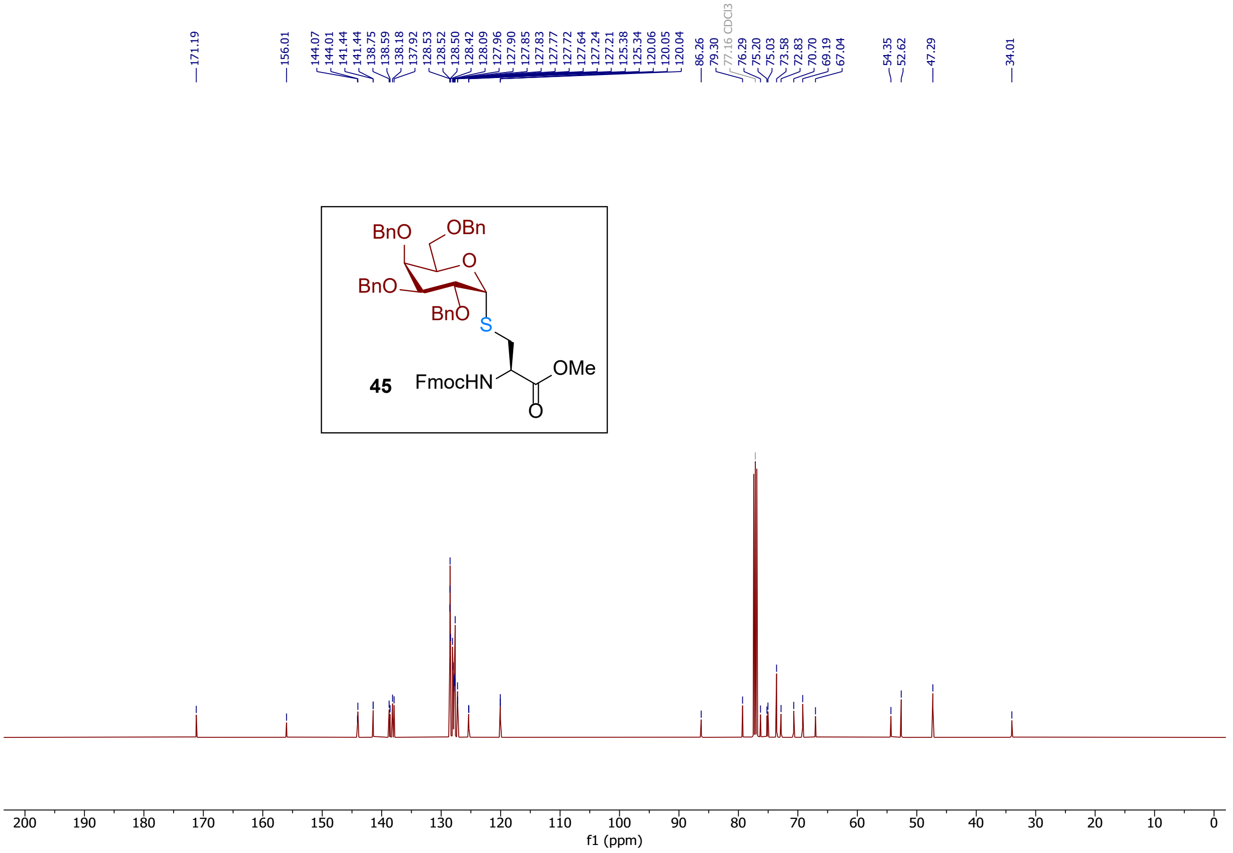

171.19
156.01
144.07 144.01 141.44 141.44 138.75 138.59 138.18 137.92 128.53 128.52 128.50 128.42 128.09 127.96 127.90 127.85 127.83 127.77 127.72 127.64 127.24 127.21 125.38 125.34 120.06 120.05 120.04
86.26 79.30
77.16 CDCl3
76.29 75.20 75.03 73.58 72.83 70.70 69.19 67.04
54.35 52.62 47.29
34.01
BnO
OBn
O
BnO
BnO
S
OMe
45
FmocHN
O
200 190 180 170 160 150 140 130 120 110 100 90 80 70 60 50 40 30 20 10 0
f1 (ppm)

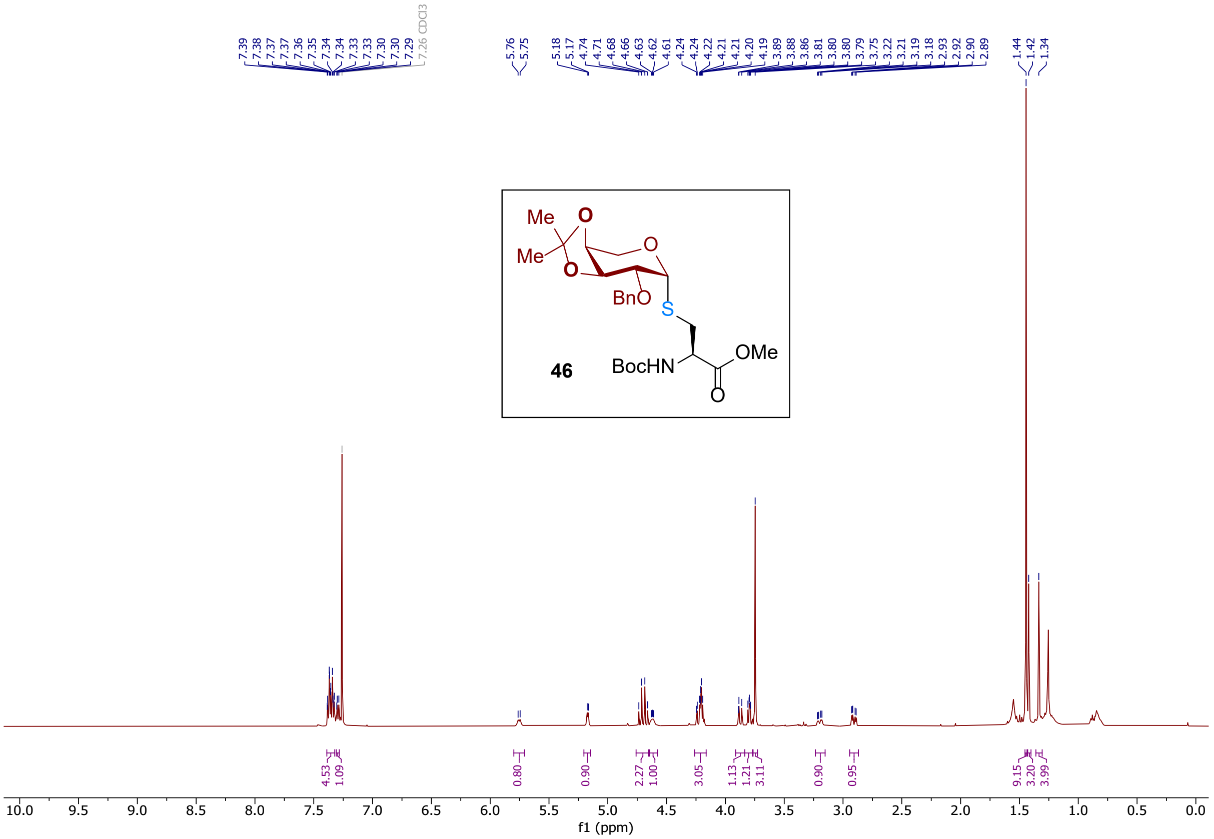
7.39 7.38 7.37 7.37 7.36 7.35 7.34 7.34 7.33 7.33 7.30 7.30 7.29 7.26 CDCl3
5.76 5.75
5.18 5.17 4.74 4.71 4.68 4.66 4.63 4.62 4.61 4.24 4.24 4.22 4.21 4.21 4.20 4.19 3.89 3.88 3.86 3.81 3.80 3.80 3.79 3.75 3.22 3.21 3.19 3.18 2.93 2.92 2.90 2.89
1.44 1.42 1.34
Me
O
Me
O
O
BnO
S
46
BocHN
OMe
O
4.53 1.09 0.80 0.90 2.27 1.00 3.05 1.13 1.21 3.11 0.90 0.95 9.15 3.20 3.99
10.0 9.5 9.0 8.5 8.0 7.5 7.0 6.5 6.0 5.5 5.0 4.5 4.0 3.5 3.0 2.5 2.0 1.5 1.0 0.5 0.0
f1 (ppm)

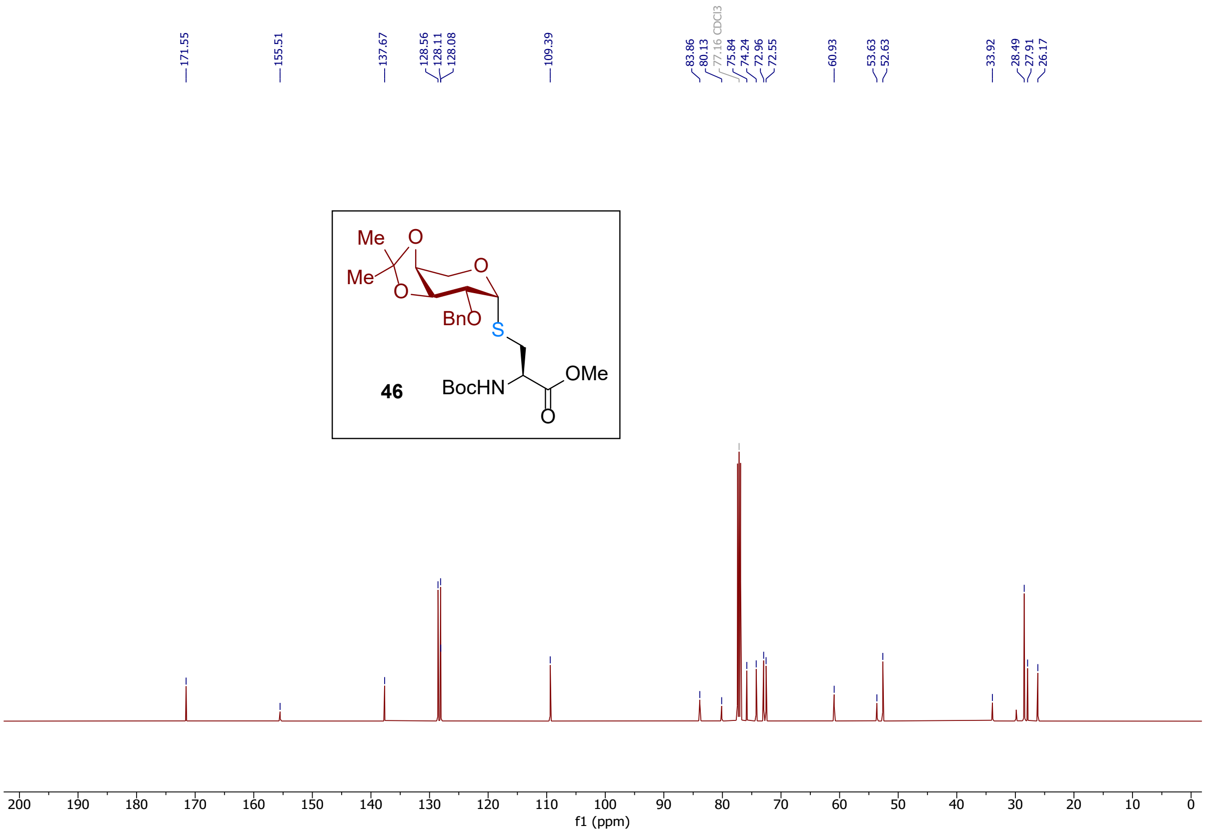
171.55
155.51
137.67
128.56 128.11 128.08
109.39
83.86 80.13 77.16 CDCl3 75.84 74.24 72.96 72.55
60.93
53.63 52.63
33.92
28.49 27.91 26.17
Me
O
Me
O
O
BnO
S
46
BocHN
OMe
O
200 190 180 170 160 150 140 130 120 110 100 90 80 70 60 50 40 30 20 10 0
f1 (ppm)

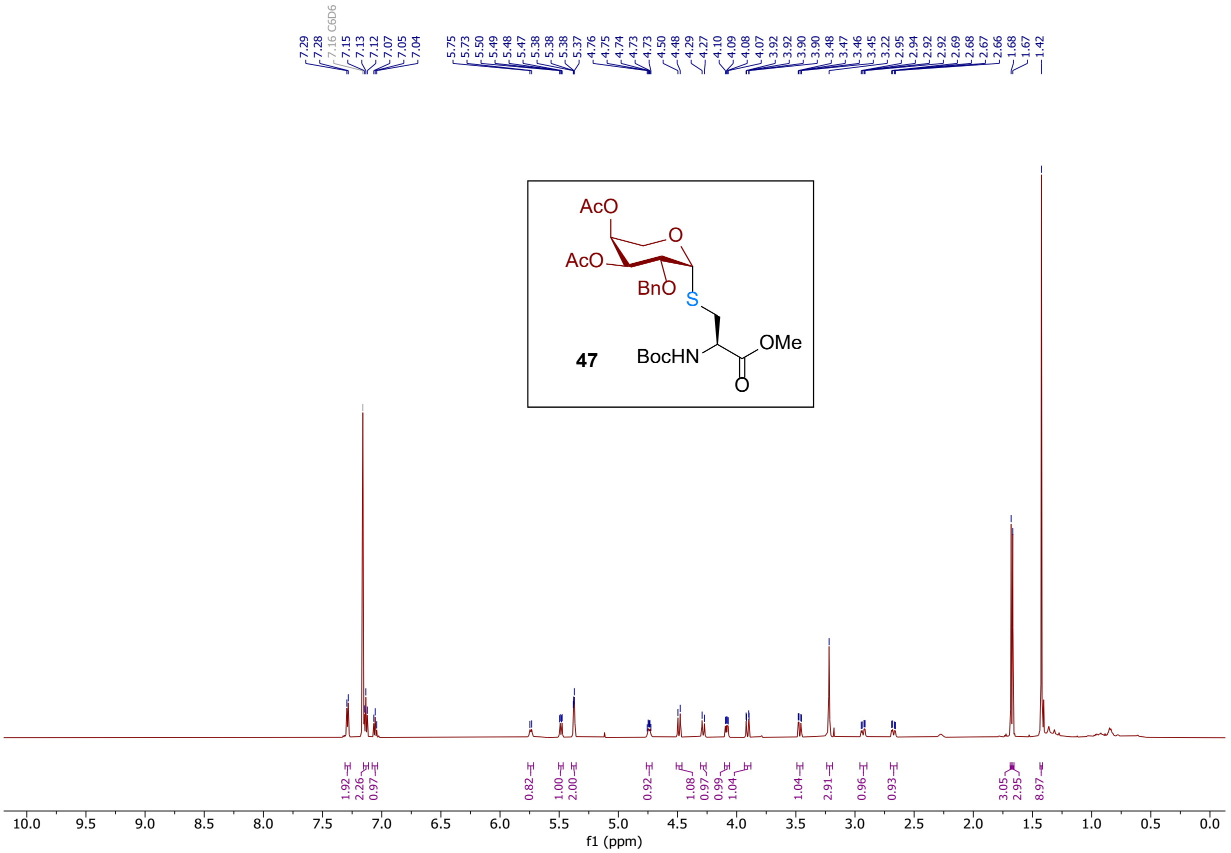
7.29 7.28 7.16 C6D6 7.15 7.13 7.12 7.07 7.05 7.04 5.75 5.73 5.50 5.49 5.48 5.47 5.38 5.38 5.38 5.37 4.76 4.75 4.74 4.73 4.73 4.50 4.48 4.29 4.27 4.10 4.09 4.08 4.07 3.92 3.92 3.90 3.90 3.48 3.47 3.46 3.45 3.22 2.95 2.94 2.92 2.92 2.69 2.68 2.67 2.66 1.68 1.67 1.42
AcO
AcO
O
BnO
S
OMe
47
BocHN
O
1.92 2.26 0.97 0.82 1.00 2.00 0.92 1.08 0.97 0.99 1.04 1.04 2.91 0.96 0.93 3.05 2.95 8.97
f1 (ppm)

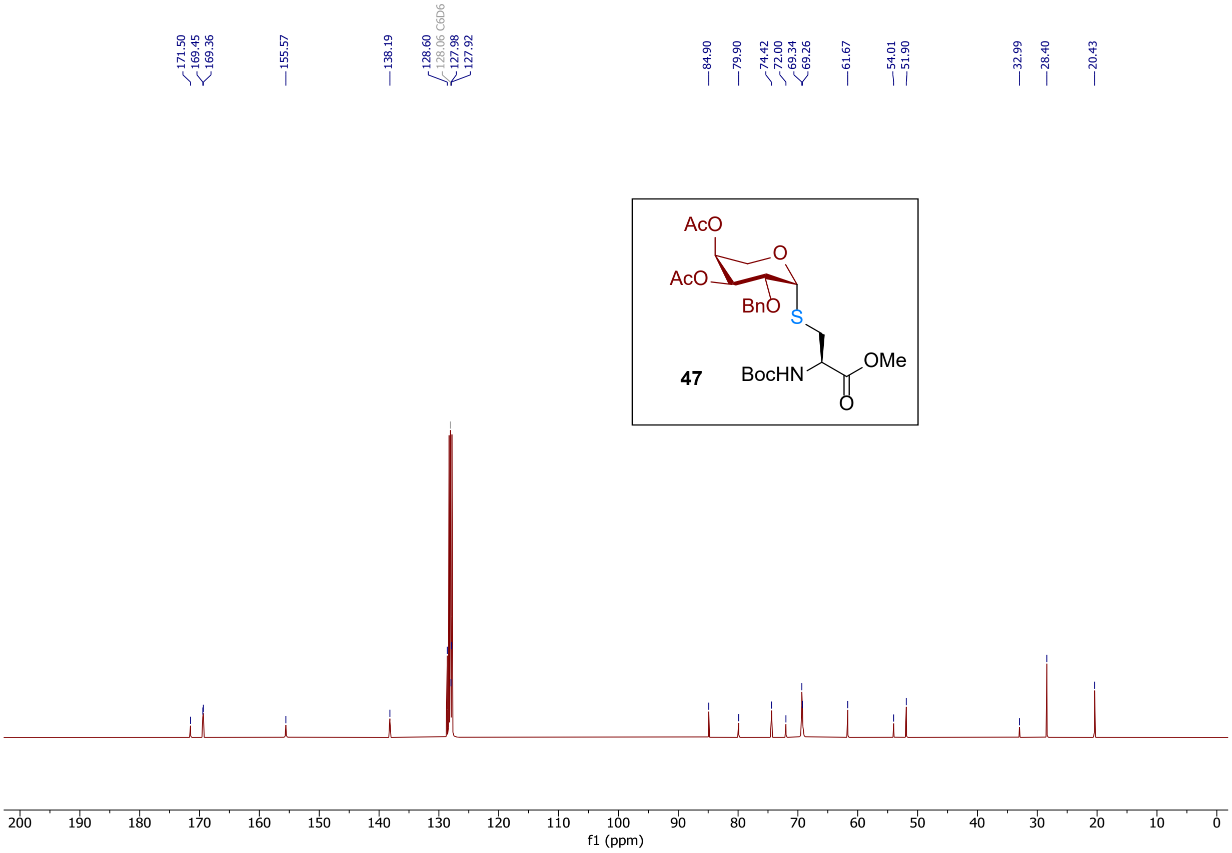
171.50 169.45 169.36 155.57 138.19 128.60 128.06 C6D6 127.98 127.92 84.90 79.90 74.42 72.00 69.34 69.26 61.67 54.01 51.90 32.99 28.40 20.43
AcO
AcO
O
BnO
S
OMe
47
BocHN
O
f1 (ppm)

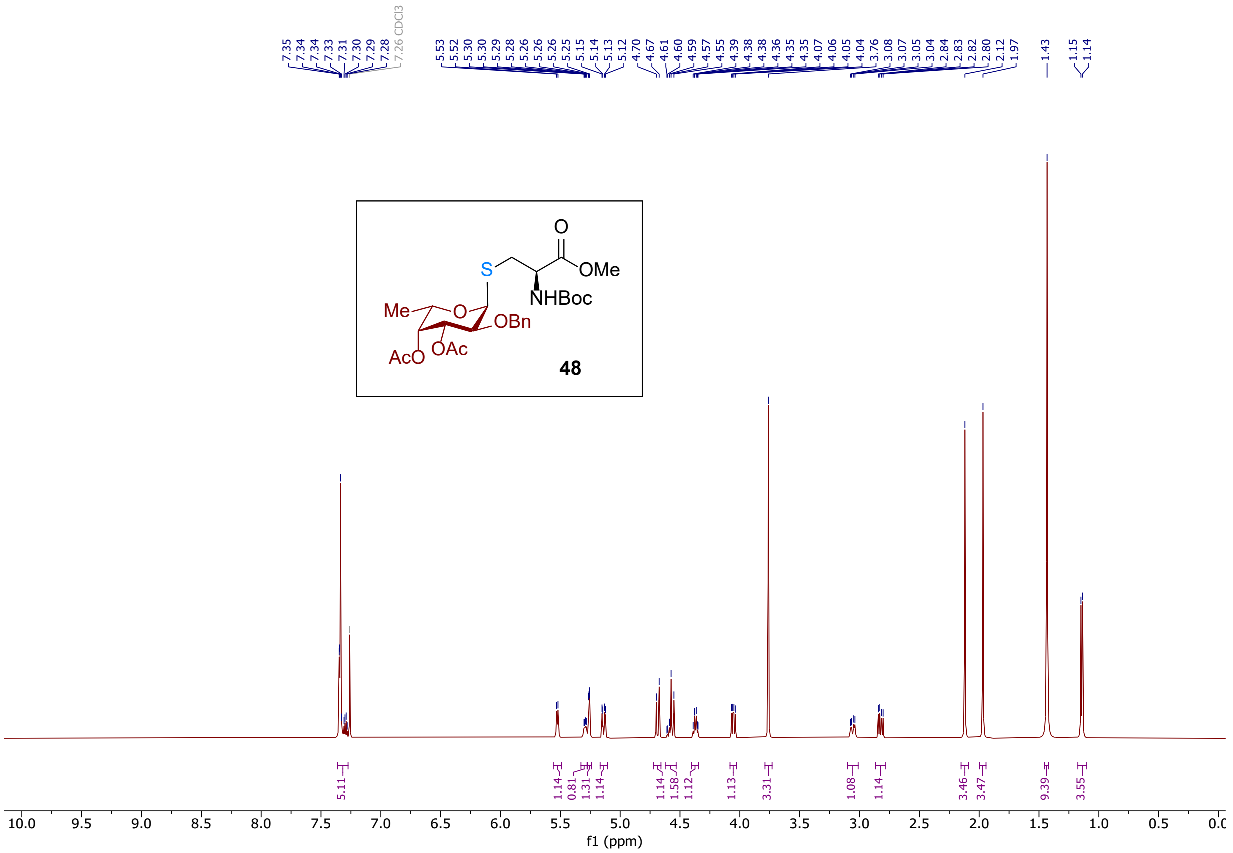
7.35
7.34
7.34
7.33
7.31
7.30
7.29
7.28
7.26 CDCl3
5.53
5.52
5.30
5.30
5.29
5.28
5.26
5.26
5.26
5.25
5.15
5.14
5.13
5.12
4.70
4.67
4.61
4.60
4.59
4.57
4.55
4.39
4.38
4.38
4.36
4.35
4.35
4.07
4.06
4.05
4.04
3.76
3.08
3.07
3.05
3.04
2.84
2.83
2.82
2.80
2.12
1.97
1.43
1.15
1.14
O
OMe
S
NHBoc
Me
O
OBn
AcO
OAc
48
5.11
1.14
0.81
1.31
1.14
1.14
1.58
1.12
1.13
3.31
1.08
1.14
3.46
3.47
9.39
3.55
f1 (ppm)

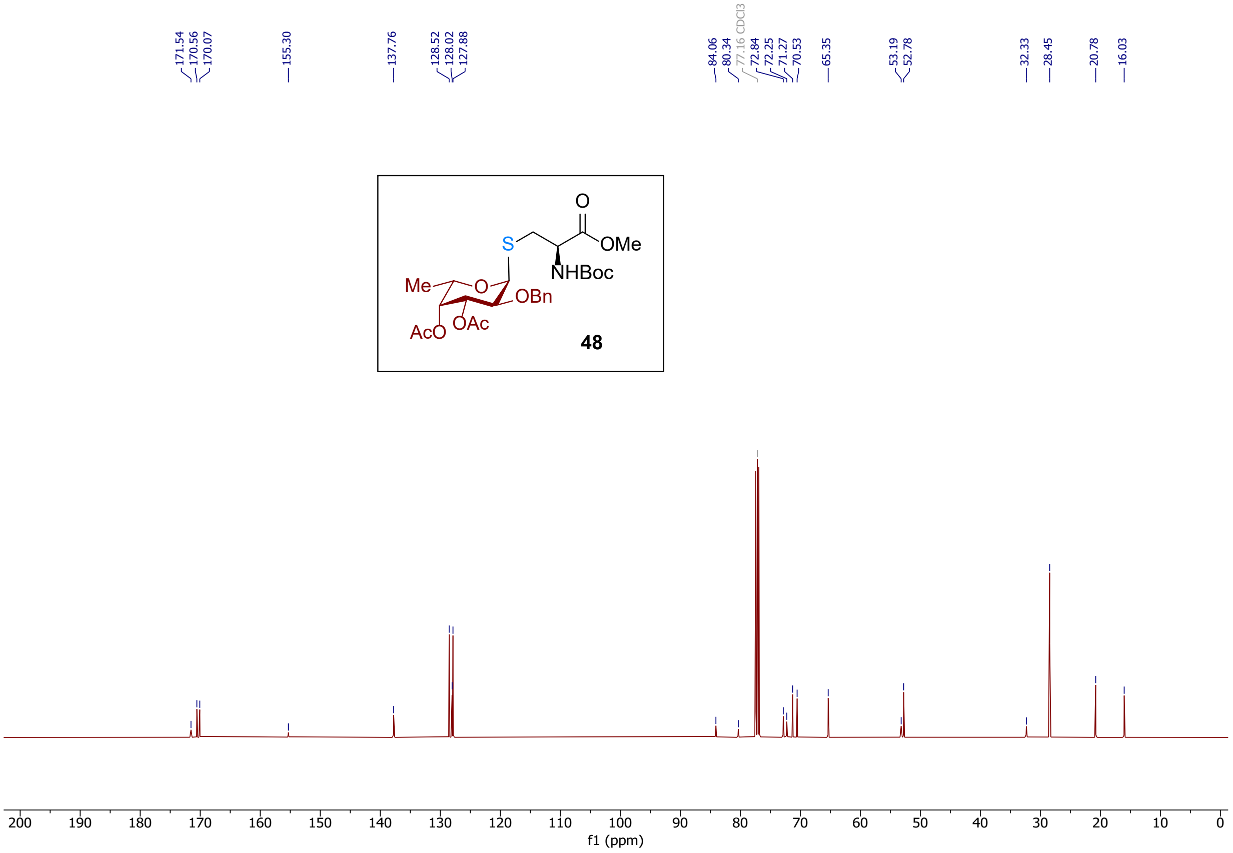
171.54
170.56
170.07
155.30
137.76
128.52
128.02
127.88
84.06
80.34
77.16 CDCl3
72.84
72.25
71.27
70.53
65.35
53.19
52.78
32.33
28.45
20.78
16.03
O
OMe
S
NHBoc
Me
O
OBn
AcO
OAc
48
f1 (ppm)

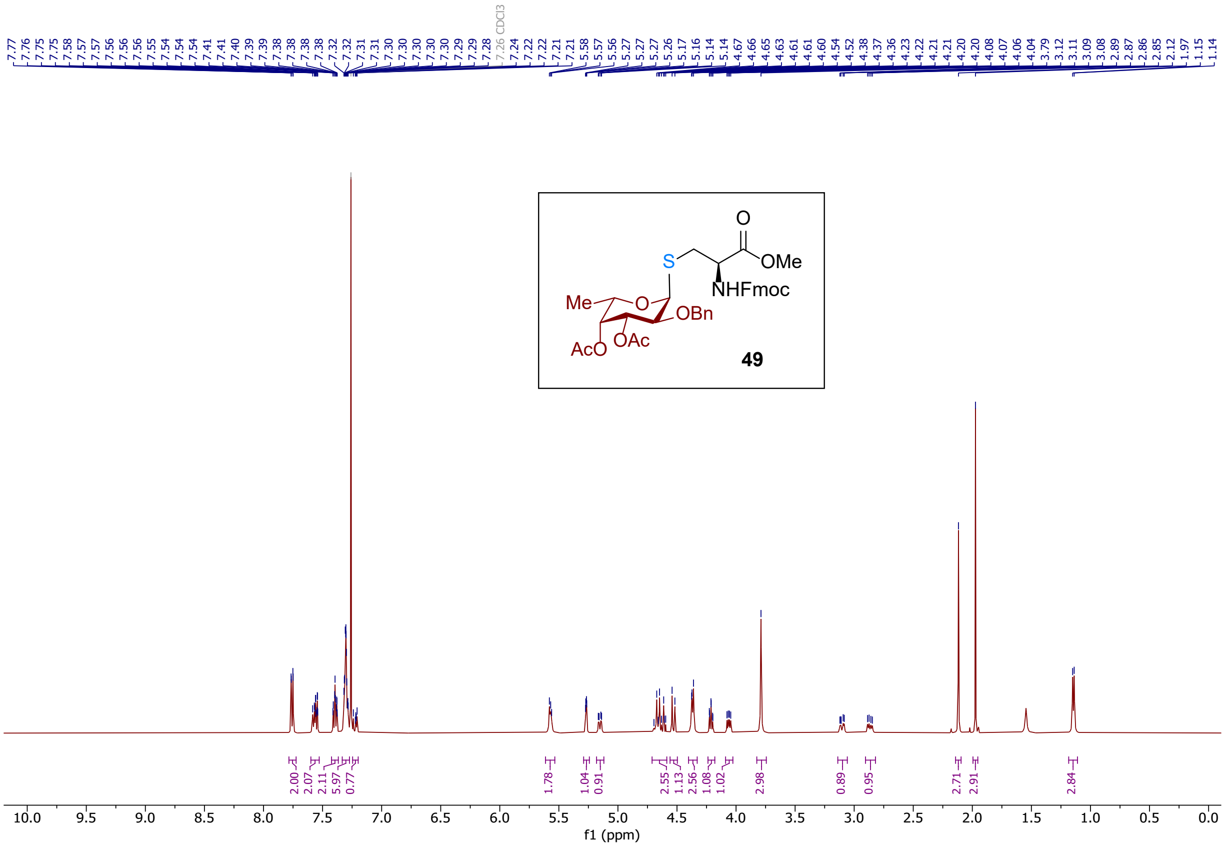
7.77 7.76 7.75 7.58 7.57 7.57 7.56 7.56 7.55 7.54 7.54 7.41 7.41 7.40 7.39 7.39 7.38 7.38 7.38 7.38 7.32 7.32 7.31 7.30 7.30 7.30 7.30 7.30 7.29 7.29 7.28 7.26 CDCl3 7.24 7.22 7.22 7.21 7.21 5.58 5.57 5.56 5.27 5.27 5.27 5.26 5.17 5.16 5.14 5.14 4.67 4.66 4.65 4.63 4.61 4.61 4.60 4.54 4.52 4.38 4.37 4.36 4.23 4.22 4.21 4.21 4.20 4.20 4.08 4.07 4.06 4.04 3.79 3.12 3.11 3.09 3.08 2.89 2.87 2.86 2.85 2.12 1.97 1.15 1.14
S
O
OMe
NHFmoc
Me
O
OBn
OAc
AcO
49
2.00 2.07 2.11 5.97 0.77 1.78 1.04 0.91 2.55 1.13 2.56 1.08 1.02 2.98 0.89 0.95 2.71 2.91 2.84
10.0 9.5 9.0 8.5 8.0 7.5 7.0 6.5 6.0 5.5 5.0 4.5 4.0 3.5 3.0 2.5 2.0 1.5 1.0 0.5 0.0
f1 (ppm)

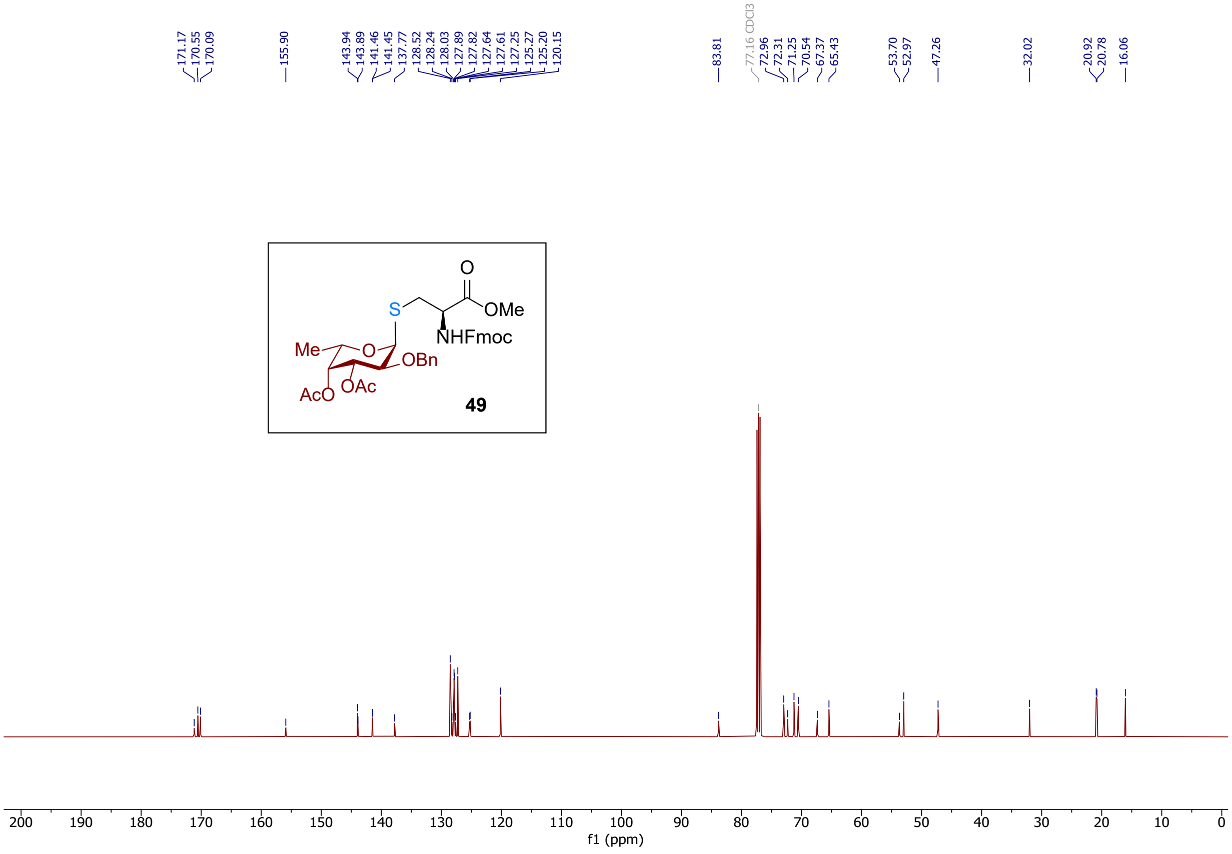
171.17 170.55 170.09 155.90 143.94 143.89 141.46 141.45 137.77 128.52 128.24 128.03 127.89 127.82 127.64 127.61 127.25 125.27 125.20 120.15 83.81 77.16 CDCl3 72.96 72.31 71.25 70.54 67.37 65.43 53.70 52.97 47.26 32.02 20.92 20.78 16.06
S
O
OMe
NHFmoc
Me
O
OBn
OAc
AcO
49
200 190 180 170 160 150 140 130 120 110 100 90 80 70 60 50 40 30 20 10 0
f1 (ppm)

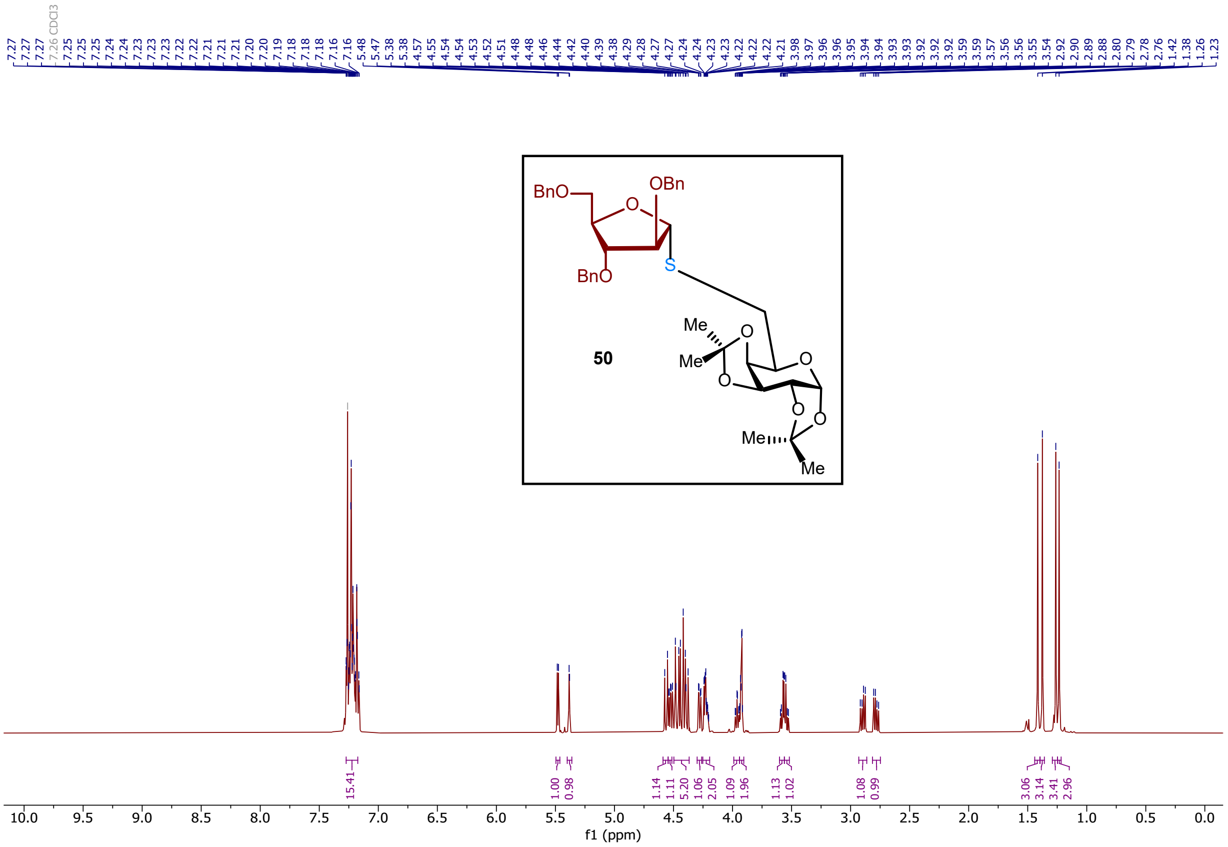

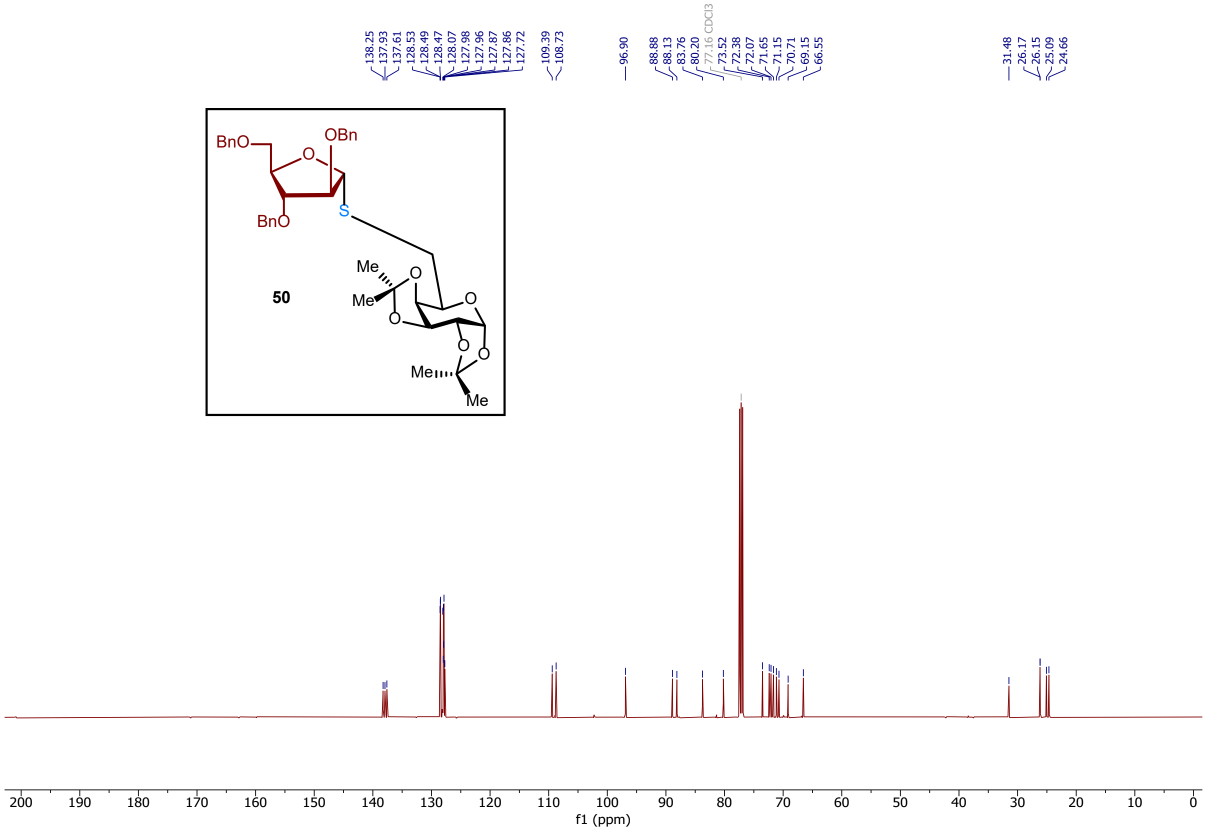

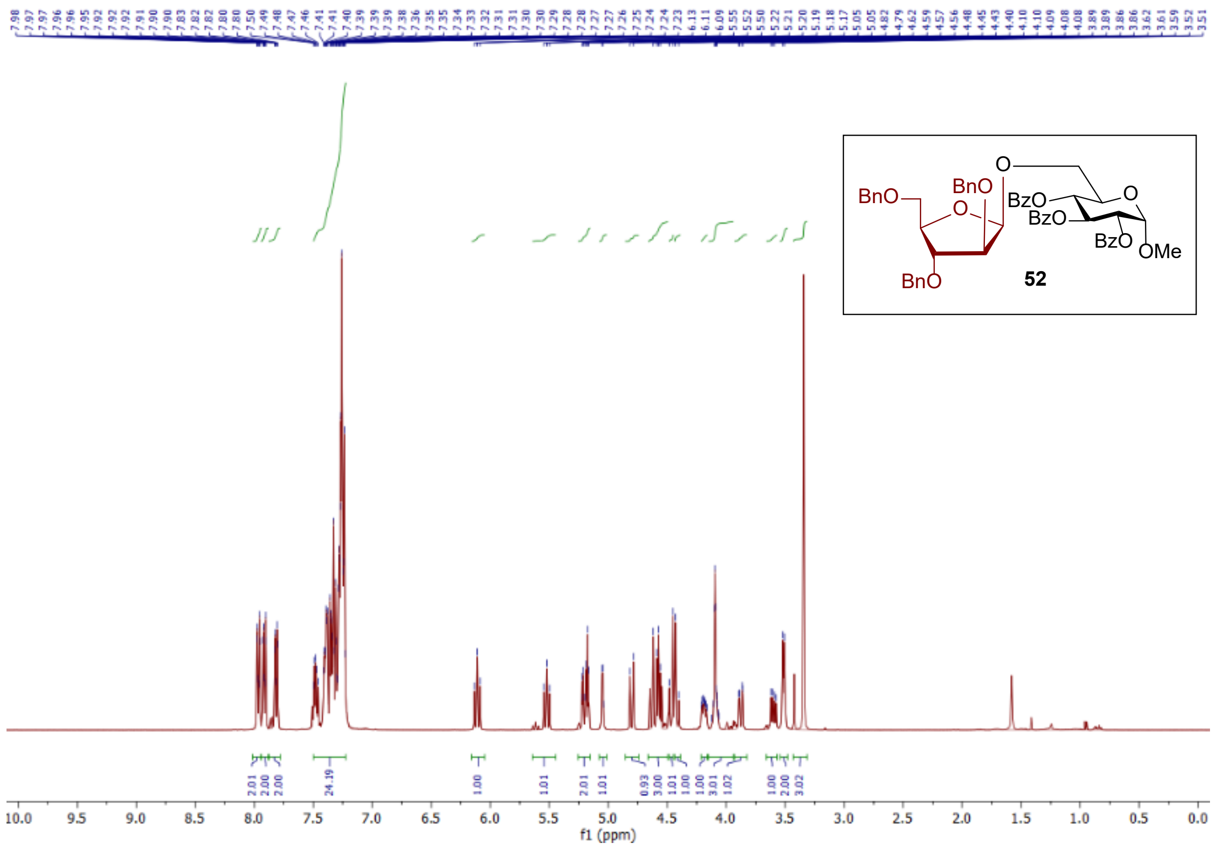
BnO
BnO
O
O
BzO
BzO
O
BzO
OMe
BnO
52
f1 (ppm)

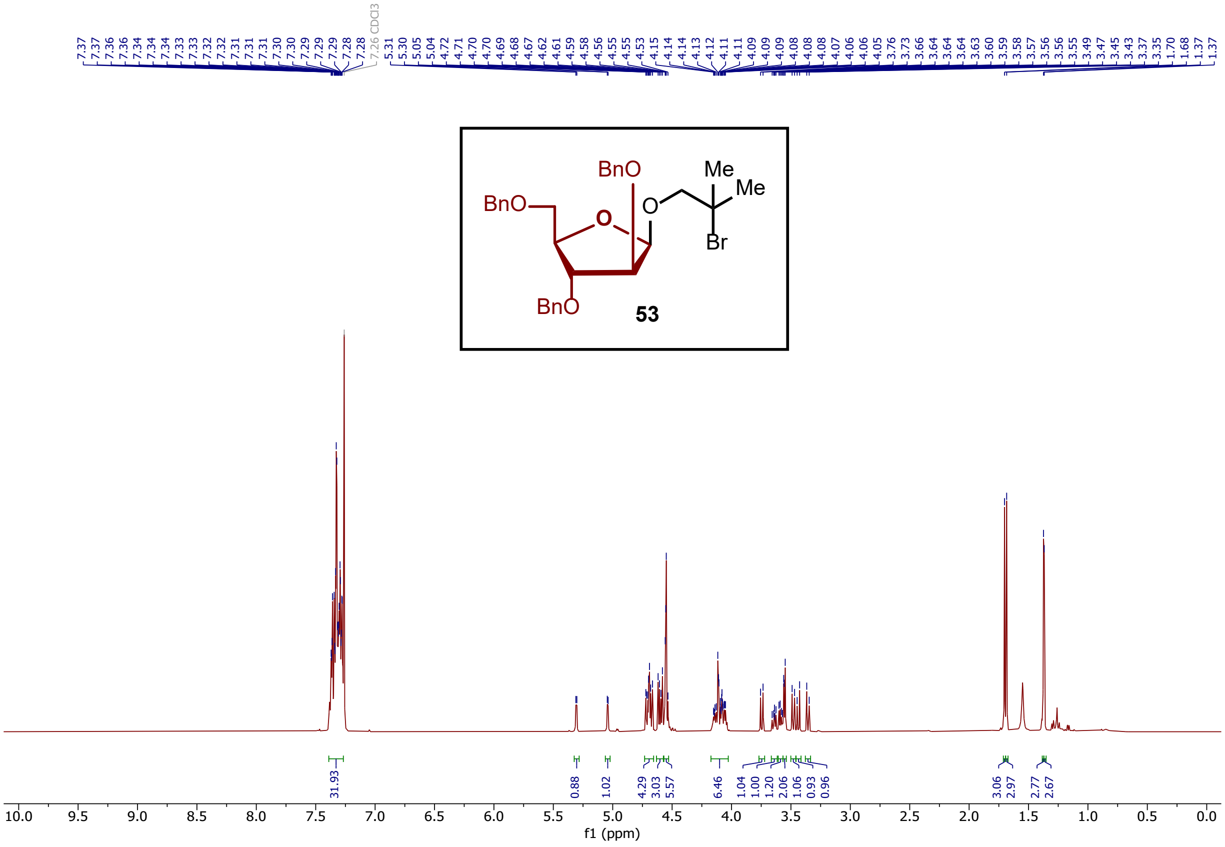
BnO
BnO
O
O
Me
Me
Br
BnO
53
f1 (ppm)

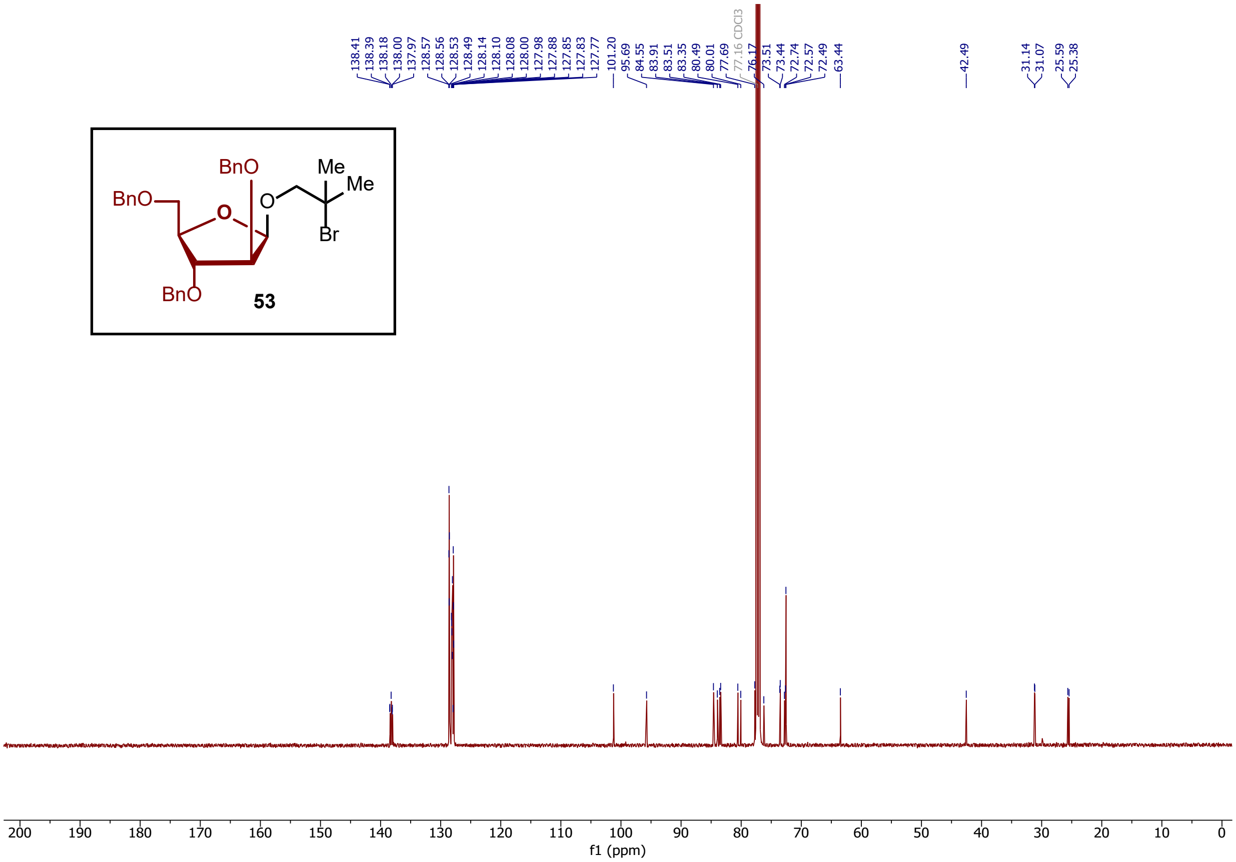
BnO
BnO
O
O
Me
Me
Br
BnO
53
138.41
138.39
138.18
138.00
137.97
128.57
128.56
128.53
128.49
128.14
128.10
128.08
128.00
127.98
127.88
127.85
127.83
127.77
101.20
95.69
84.55
83.91
83.51
83.35
80.49
80.01
77.69
77.16 CDCl3
76.17
73.51
73.44
72.74
72.57
72.49
63.44
42.49
31.14
31.07
25.59
25.38
200
190
180
170
160
150
140
130
120
110
100
90
80
70
60
50
40
30
20
10
0
f1 (ppm)
